# Supplementary material for: Effect of intensive treatment for schistosomiasis on immune responses to vaccines among rural Ugandan island adolescents: randomised controlled trial protocol A for the ‘POPulation differences in VACcine responses’ (POPVAC) programme
Source: BMJ Open. 2021 Feb 16;11(2):e040426. doi: 10.1136/bmjopen-2020-040426 (PMC7888376; doi:10.1136/bmjopen-2020-040426)
Supplement: Supplementary data [file bmjopen-2020-040426supp002.pdf]

MRC/UVRI and LSHTM Uganda Research Unit

MRC Medical Research Council | Uganda Virus Research Institute | LONDON SCHOOL of HYGIENE &TROPICAL MEDICINE

Population differences in vaccine responses: the effect of intensive treatment for schistosomiasis on response to vaccines among island adolescents

Short Title: POPVAC Project A

Information Sheet for participants 9 – 12 years

1. Why are we meeting with you?

We are inviting you to take part in a research which is being done by a team from Uganda Virus Research Institute (UVRI), working together with colleagues from the London School of Hygiene and Tropical Medicine, UK. We are meeting you because you are aged between 9 and 12 years and study in a primary school on Koome island. After we tell you about it, we will ask if you'd like to be in this study or not. Only If you agree to take part, will you sign the assent form to show us that you are happy to do so.

The people leading this research study are Professor Alison Elliott and Dr Ludoviko Zirimenya.

2. Why are we doing this research?

We want to **find out whether treating Bilharzia much more frequently (a total of 7 times in a year) can get rid of it completely and whether this will improve vaccine responses.**

In the whole study, there will be about 480 children from schools of Koome islands.

3. What is going to happen in this research study?

Only if you agree, a full health check-up that will involve taking stool, urine and blood samples will be done. If everything is okay, the following will be done:

You will be put into one of the two **Bilharzia treatment** groups by chance

- One group will be treated for Bilharzia using praziquantel (the recommended medicine for Bilharzia) seven times in the year, and an eighth time after the end of the study.
- The other group will be treated for Bilharzia using praziquantel once in the year, and a second time after the end of the study.

You will be immunised with **five (5) vaccines.** These are:

- BCG vaccine. This is intended to protect against tuberculosis.
- Yellow fever vaccine. This protects against Yellow Fever an infection carried by mosquitoes.
- HPV vaccine. Human Papilloma Virus (HPV) causes growths in private parts and cancer in girls and boys.
- Tetanus and diphtheria vaccines. Tetanus and diphtheria are bacterial infections that cause very serious disease.
- Typhoid vaccine. Typhoid is a serious form of fever which is spread through contaminated food.

These immunisations will be given on different times during the entire duration of the study.

UGANDA VIRUS RESEARCH INSTITUTE ENTEBBE
RESEARCH ETHICS COMMITTEE
APPROVED
05 SEP 2020
P. O. BOX 49, ENTEBBE
UGANDA

Most of the vaccines will be injected into your upper arm (either right or left side). The typhoid vaccine is an oral vaccine: it is in the form of capsules which are swallowed. You will be given one capsule per day for three alternating days.

You will be asked to give **blood samples**

We will ask you to give blood samples before and after you are given the vaccines. The amount of blood that we will take at each visit is completely safe and will vary between 4 and 27 millilitres (1 teaspoon and 5 and a half teaspoons) at the different visits.

You will be asked to give **stool and urine samples**

At some visits we will ask you for additional stool and urine samples for testing to check whether the worm infections have responded to treatment or (for girls) whether you have become pregnant.

Some of you will be asked to take part in a special **sugar test** of absorption from the intestines

We think that worm infections may make our intestines a little bit leaky, and this might explain some of their effects. We can test this using a special sugar drink. We will ask about 200 people to do this additional test. If you are asked to do this, it will be done about 8 weeks after your first vaccine is given. You will be asked not to eat overnight or in the morning of the test day. Then you will be given a special drink that contains sugars early in the morning. Afterwards you will also be asked to drink a litre of water (equal to two Rwenzori bottles) over the following few hours. All the urine that you pass during the next five hours will be collected. The volume (amount) of urine will be measured and the amount of sugar in it will be tested, to find out how much of the sugar your body has absorbed.

4. What will the blood, stool and urine samples be used for?

The samples will be used to test for infections, including HIV, malaria and worms, to test for anaemia (the strength of your blood) and to test for pregnancy (among girls). The blood samples will also be used to test how your body responds to vaccines. For some participants, the sugar test will be used to test absorption from the intestines.

5. Will taking part in this study harm me?

We do not expect this to harm you, though you will experience the following:

- You will need to take time off classes during each visit by the study team.
- Taking blood samples is not expected to cause any problem for you, apart from the discomfort or pain, bruising or bleeding, redness at the place where the needle goes into the skin, but this will go away in a few days.
- During the sugar test, you will be asked not to eat overnight and during the morning of the test so you may get hungry (although water and a snack will be provided). This may make you feel a little sick and stools may become a bit loose.
- If you are a girl, even if you know you could not be pregnant, we will do a pregnancy test. We have to be quite sure. You must as well promise that you can avoid getting pregnant while taking part in this study.

UGANDA VIRUS RESEARCH INSTITUTE ENTEBBE
RESEARCH ETHICS COMMITTEE
APPROVED
05 SEP 2020
P. O. BOX 49, ENTEBBE
UGANDA

MRC/UVRI and LSHTM Uganda Research Unit

MRC Medical Research Council | Uganda Virus Research Institute | LONDON SCHOOL of HYGIENE &TROPICAL MEDICINE

- Treatment of Bilharzia with praziquantel may make you feel dizzy or sick, give you abdominal pain or diarrhoea, or occasionally cause an itchy rash. The research team will have medicines available to help you if you have a strong reaction to the treatment.
- All the study vaccines are known to be safe. However, even approved vaccines may very occasionally cause a serious reaction, such as an allergic reaction. The research doctors and nurses will be available to help if this happens.

6. Will the study help me?

The treatment for Bilharzia is likely to be good for you, whichever group you are put in. The vaccines are likely to help you by protecting you from infectious diseases. The research results may help all people in the world, because in the end we may get a better understanding whether treating Bilharzia much more frequently (a total of 7 times in a year) can get rid of it completely and whether this will improve vaccine responses.

7. What is the cost of taking part in the trial?

There is no cost to participate in this trial. You will receive a soft drink and the gift of a pen or other simple school material on days when blood samples are drawn, and the gift of a T-shirt at the end of the study when everything is done, in appreciation of your contribution to the work.

8. What happens if I refuse to take part?

It is very important for you to know that you do not have to take part in the research, the choice is yours. No-one will be upset if you decide not to take part. If you agree to take part and later decide that you do not want to take part anymore, that is also okay.

9. What happens if something goes wrong?

The researchers will make every effort to ensure your safety and well-being. MRC/UVRI and LSHTM has a specialist insurance policy in place which would help to pay for treatment if any harm came to you as a result of taking part in the research.

10. Who will have access to your information and samples from this research?

Only research staff trained to keep the information confidential will have access to the records. Your name will be removed from the records and samples, so no-one will be able to find out information about you from our records.

11. Who has reviewed the trial?

This trial has been reviewed by the Uganda National Council for Science and Technology, The Uganda National Drug Authority, the Uganda Virus Research Institute Research and Ethics Committee the ethics committee of the London School of Hygiene and Tropical Medicine in the United Kingdom.

You can find out more about the study at any time by asking one of the members of the research team. You may also contact Professor Alison Elliott (telephone: 0417 704000) who is in charge of the study, or the Chairperson of the Ethics Committee for the Uganda Virus Research Institute on 0414 321962.

UGANDA VIRUS RESEARCH INSTITUTE RESEARCH ETHICS COMMITTEE APPROVED 05 SEP 2020 P.O. BOX 49, ENTEBBE UGANDA

MRC/UVRI and LSHTM Uganda Research Unit

MRC Medical Research Council | Uganda Virus Research Institute | LONDON SCHOOL of HYGIENE &TROPICAL MEDICINE

Population differences in vaccine responses: the effect of intensive treatment for schistosomiasis on response to vaccines among island adolescents

Short Title: POPVAC Project A

Participant Assent

A copy of this form will be given to you. Please keep a copy of the form because it contains the information that was discussed with you and you may want to look at it again.

AGREEMENT TO TAKE PART:

I have read and understood (or been read to and understand) the information sheet for this study. My questions have been answered. I understand that taking part in the study is voluntary. I understand that at any time I may withdraw from this study without giving a reason. I agree to participate in this study.

Name: ________________________________ **PVA ID: |A|___|___|___|___|**
(please write your name in capital letters here if you agree)

Signature: ________________________________ **Date:**________________
(please sign or write your name here if you agree; or use a thumbprint)

What if I have any questions?
If you have any questions about your participation in this study, please feel free to ask any member of the research team at any time. If you prefer, you may speak to the principal investigator for this study (Professor Alison Elliott, telephone 0417 704000).

What if we want to ask someone independent anything about this research, or have any questions about your rights as a research participant? You may speak with the Chairman of the Science and Ethics Committee at Uganda Virus Research Institute on 0414 321962.

UGANDA VIRUS RESEARCH INSTITUTE ENTEBBE
RESEARCH ETHICS COMMITTEE
APPROVED
05 SEP 2020
P. O. BOX 49, ENTEBBE
UGANDA

PVA ID: |A|___|___|___|___|

Witness:

I have read the participant information sheet and the assent statement above

to:.. (PRINT NAME OF PARTICIPANT) in a language which he/she understands. I believe that he/she gives assent to take part in the study.

....	....	....
**Witness name**	**Signature**	**Date**

Witness required only for those using a thumb print instead of the final signature, or unable to read the information and consent form, or if the person taking consent does not speak the participant's language. The witness must not be a member of the research staff or a study participant. The witness must be present for the whole consent process.

Person taking the assent:

....	....	....
**Researcher name**	**Signature**	**Date**

Comment:

UGANDA VIRUS RESEARCH INSTITUTE ENTEBBE
RESEARCH ETHICS COMMITTEE
APPROVED
VALID UNTIL
05 SEP 2020
P. O. BOX 49, ENTEBBE
UGANDA

MRC/UVRI and LSHTM Uganda Research Unit

MRC Medical Research Council | Uganda Virus Research Institute | LONDON SCHOOL of HYGIENE &TROPICAL MEDICINE

Population differences in vaccine responses: the effect of intensive treatment for schistosomiasis on response to vaccines among island adolescents

Short Title: POPVAC Project A

Information Sheet for participants aged 13 to 17 years

We are inviting you to join in some research which is being done by a team from Uganda Virus Research Institute (UVRI), working together with colleagues from the London School of Hygiene and Tropical Medicine, UK. This form will tell you about the research. If you have any questions, please ask the person who is telling you about this research. Then you can decide whether you want to take part or not. There is no need to take part unless you really wish to. If you agree to take part, you will need to sign the assent form to show us that you are happy to do so. If you decide you do not want to take part, do not sign the assent form.

The people leading this research study are Professor Alison Elliott and Dr Ludoviko Zirimenya.

1. **What this research is about, and the reason for doing this research.**

This research is about how worm infections like Bilharzia "switch off" the body's defence systems and how this affects vaccine responses. **We want to find out whether treating Bilharzia much more frequently (a total of 7 times in a year) can get rid of it completely and whether this will improve vaccine responses.**

2. **Why have I been asked to take part in this study?**

You have been asked because you are attending primary school in Koome islands.

3. **What is going to happen in this research study?**

Only if you agree, a comprehensive health check-up that will involve taking stool, urine and blood samples will be done. If everything is okay, the following will be done:

You will be put into one of the two **Bilharzia treatment** groups by luck

- One group will be treated for Bilharzia using praziquantel (the recommended medicine for Bilharzia) seven times in the year, and an eighth time after the end of the study.
- The other group will be treated for Bilharzia using praziquantel once in the year, and a second time after the end of the study.

You will be immunised with **several vaccines**. These are the vaccines:

- BCG vaccine. This is intended to protect against tuberculosis.
- Yellow fever vaccine. This protects against Yellow Fever virus carried by mosquitoes.
- HPV vaccine. Human Papilloma Virus (HPV) causes growths in private parts and cancer in girls and boys.
- Tetanus and diphtheria vaccines. Tetanus and diphtheria are bacterial infections that cause very serious disease.
- Typhoid vaccine. Typhoid is a serious form of fever which is spread through contaminated food.

These immunisations will be given on three different days.

UGANDA VIRUS RESEARCH INSTITUTE ENTEBBE RESEARCH ETHICS COMMITTEE APPROVED VALID UNTIL 05 SEP 2020 P. O. BOX 49, ENTEBBE UGANDA

MRC/UVRI and LSHTM Uganda Research Unit

Medical Research Council MRC | Uganda Virus Research Institute | LONDON SCHOOL of HYGIENE &TROPICAL MEDICINE

Most of the vaccines will be injected into your upper arm (either right or left side). This will be a bit painful, as for any injection of medicine that you may have had, and will feel a bit sore over the next week or so. The typhoid vaccine is an oral vaccine: it is in the form of capsules which are swallowed. You will be given one capsule per day for three alternating days.

You will be asked to give **blood samples**

We will ask you to give blood samples before and after you are given the vaccines. The amount of blood that we will take at each visit is completely safe and will vary between 4 and 27 millilitres (1 teaspoon and 5 and a half teaspoons) at the different visits

You will be asked to give **stool and urine samples**

At some visits we will ask you for additional stool and urine samples for testing to check whether the worm infections have responded to treatment or (for girls) whether you have become pregnant.

Some of you will be asked to take part in a special **sugar test** of absorption from the intestines

We think that worm infections may make our intestines a little bit leaky, and this might explain some of their effects. We can test this using a special sugar drink. We will ask about 200 people to do this additional test. If you are asked to do this, it will be done about 8 weeks after your first vaccine is given. You will be asked not to eat overnight or in the morning of the test day. Then you will be given a special drink that contains sugars early in the morning. Afterwards you will also be asked to drink a litre of water (equal to two Rwenzori bottles) over the following few hours. All the urine that you pass during the next five hours will be collected. The volume (amount) of urine will be measured and the amount of sugar in it will be tested, to find out how much of the sugar your body has absorbed.

4. What will the blood, stool and urine samples be used for?

The samples will be used to test for infections, including HIV, malaria and worms, to test for anaemia (the strength of your blood) and to test for pregnancy (among girls). The blood samples will also be used to test how your body responds to vaccines. For some participants, the sugar test will be used to test absorption from the intestines.

5. How many people will take part in the study, and how long will it last?

The whole study will enrol 480 people, and the study will last for about two years.

6. What are the risks of participating in this trial?

You will need to take time off classes during each visit by the study team.

Taking blood samples is not expected to cause any problem for you, apart from the discomfort or pain, bruising or bleeding, redness at the place where the needle goes into the skin, but this will go away in a few days.

During the sugar test you will be asked not to eat overnight and during the morning of the test so you may get hungry (although water and a snack will be provided). Most people have no problems with this test although a few may feel a little sick and stools may become a bit loose.

UGANDA VIRUS RESEARCH INSTITUTE ENTEBBE
RESEARCH ETHICS COMMITTEE
APPROVED
05 SEP 2020
P. O. BOX 49, ENTEBBE
UGANDA

If you are a girl, even if you know you could not be pregnant. We have to be quite sure. We will test for pregnancy at the beginning of the study and on each immunisation day. You must as well promise that you can avoid getting pregnant while taking part in this study.

Treatment of Bilharzia with praziquantel may make you feel dizzy or sick, give you abdominal pain or diarrhoea, or occasionally cause an itchy rash. This is most likely caused by your body's response to the worms as they are being killed by the medicine. The research team will have medicines available to help you if you have a strong reaction to the treatment.

All the study vaccines are known to be safe though not often, may cause a serious reaction, such as an allergic reaction. The research doctors and nurses will be available to help if this happens.

7. What are the benefits of taking part in this trial?

The treatment for Bilharzia is likely to be good for you, whichever group you are put in. The vaccines are likely to help you by protecting you from infectious diseases.

Also, you will be helping us to find out why vaccines sometimes don't work so well in countries like Uganda, and whether vaccines work better if worms are treated first. This may help other people in the future.

8. What is the cost of taking part in the trial?

There is no cost to participate in this trial. You will receive a soft drink and the gift of a pen or other simple school material on days when blood samples are drawn, and the gift of a T-shirt at the end of the study when everything is done, in appreciation of your contribution to the work.

9. What happens if I refuse to take part?

It is very important for you to know that you do not have to take part in the research, the choice is yours. No-one will be upset if you decide not to take part. If you agree to take part and later decide that you do not want to take part anymore, that is also okay.

10. What happens if something goes wrong?

You will be making an important contribution to medical research. The researchers will make every effort to ensure your safety and well-being. MRC/UVRI and LSHTM has a specialist insurance policy in place which would help to pay for treatment if any harm came to you as a result of taking part in the research.

11. Who will have access to my information and samples from this research?

All our research records are stored securely in rooms with restricted access and on password protected computers. Only research staff trained to keep the information confidential will have access to the records. Your name will be removed from the records, so no-one will be able to find out information about you from our records.

12. Who has reviewed the trial?

This trial has been reviewed by the Uganda National Council for Science and Technology, Uganda National Drug Authority the Uganda Virus Research Institute Research and Ethics Committee the ethics committee of the London School of Hygiene and Tropical Medicine in the United Kingdom.

UGANDA VIRUS RESEARCH INSTITUTE
RESEARCH ETHICS COMMITTEE
APPROVED
VALID UNTIL
05 SEP 2020
P. O. BOX 49, ENTEBBE
UGANDA

MRC/UVRI and LSHTM Uganda Research Unit

MRC Medical Research Council Uganda Virus Research Institute LONDON SCHOOL of HYGIENE &TROPICAL MEDICINE

You can find out more about the study at any time by asking one of the members of the research team. You may also contact Professor Alison Elliott (telephone: 0417 704000) who is in charge of the study, or the Chairperson of the Ethics Committee for the Uganda Virus Research Institute on 0414 321962.

UGANDA VIRUS RESEARCH INSTITUTE ENTEBBE
RESEARCH ETHICS COMMITTEE
APPROVED
05 SEP 2020
P. O. BOX 49, ENTEBBE
UGANDA

MRC/UVRI and LSHTM Uganda Research Unit

MRC Medical Research Council | Uganda Virus Research Institute | LONDON SCHOOL of HYGIENE &TROPICAL MEDICINE

Population differences in vaccine responses: the effect of intensive treatment for schistosomiasis on response to vaccines among island adolescents

Short Title: POPVAC Project A

Participant Assent

A copy of this form will be given to you. Please keep a copy of the form because it contains the information that was discussed with you and you may want to look at it again.

AGREEMENT TO TAKE PART:

I have read and understood (or been read to and understand) the information sheet for this study. My questions have been answered. I understand that taking part in the study is voluntary. I understand that at any time I may withdraw from this study without giving a reason. I agree to participate in this study.

Name: ______________________________ **PVA ID:** |**A**|___|___|___|___|
(please write your name in capital letters here if you agree)

Signature: ______________________________ **Date:**________________
(please sign or write your name here if you agree; or use a thumbprint)

What if I have any questions?
If you have any questions about your participation in this study, please feel free to ask any member of the research team at any time. If you prefer, you may speak to the principal investigator for this study (Professor Alison Elliott, telephone 0417 704000).

What if we want to ask someone independent anything about this research, or have any questions about your rights as a research participant? You may speak with the Chairman of the Science and Ethics Committee at Uganda Virus Research Institute on 0414 321962.

UGANDA VIRUS RESEARCH INSTITUTE ENTEBBE RESEARCH ETHICS COMMITTEE APPROVED 05 SEP 2020 P. O. BOX 49, ENTEBBE UGANDA

PVA ID: |A|___|___|___|___|

Witness:

I have read the participant information sheet and the assent statement above

to:... (PRINT NAME OF PARTICIPANT) in a language which he/she understands. I believe that he/she gives assent to take part in the study.

....	....	....
**Witness name**	**Signature**	**Date**

Witness required only for those using a thumb print instead of the final signature, or unable to read the information and consent form, or if the person taking consent does not speak the participant's language. The witness must not be a member of the research staff or a study participant. The witness must be present for the whole consent process.

Person taking the assent:

....	....	....
**Researcher name**	**Signature**	**Date**

Comment:

POPVAC Project A, assent age 13-17 years v1.3, 1st July 2019 |Page **6** of **6**

UGANDA VIRUS RESEARCH INSTITUTE ENTEBBE
RESEARCH ETHICS COMMITTEE
APPROVED
05 SEP 2020
P. O. BOX 49, ENTEBBE
UGANDA

MRC/UVRI and LSHTM Uganda Research Unit

MRC Medical Research Council | Uganda Virus Research Institute | LONDON SCHOOL of HYGIENE &TROPICAL MEDICINE

Population differences in vaccine responses: the effect of intensive treatment for schistosomiasis on response to vaccines among island adolescents

Short Title: POPVAC Project A

Information Sheet for emancipated or mature minors

We are inviting you to join in some research which is being done by a team from Uganda Virus Research Institute (UVRI), working together with colleagues from the London School of Hygiene and Tropical Medicine, UK. This form will tell you about the research. If you have any questions, please ask the person who is telling you about this research. Then you can decide whether you want to take part or not. If you agree to take part, you will need to sign the consent form to show us that you are happy to do so. If you decide that you do not want to take part, do not sign the consent form.

The people leading this research study are Professor Alison Elliott and Dr Ludoviko Zirimenya.

1. What is this research about?

This research is about how worm infections like Bilharzia "switch off" the body's defence systems and how this affects vaccine responses. **We want to find out whether treating Bilharzia much more frequently (a total of 7 times in a year) can get rid of it completely and whether this will improve vaccine responses.**

2. Why have I been asked to take part in this study?

You have been asked because you are attending primary school in Koome islands.

3. What is going to happen in this research study?

Only if you agree, you will have a comprehensive health check-up that will involve taking stool, urine and blood samples. If everything is okay, the following will be done:

You will be put into one of the two **Bilharzia treatment** groups by chance

One group will be treated for Bilharzia using praziquantel (the recommended medicine for Bilharzia) seven times in the year, and an eighth time after the end of the study.

The other group will be treated for Bilharzia using praziquantel once in the year, and a second time after the end of the study.

UGANDA VIRUS RESEARCH INSTITUTE ENTEBBE RESEARCH ETHICS COMMITTEE APPROVED 09 SEP 2020 P. O. BOX 49, ENTEBBE UGANDA

MRC/UVRI and LSHTM Uganda Research Unit

MRC Medical Research Council | Uganda Virus Research Institute | LONDON SCHOOL of HYGIENE &TROPICAL MEDICINE

You will be immunised with **five (5) vaccines**. These are:

- **BCG vaccine.** This is intended to protect against tuberculosis.
- **Yellow fever vaccine.** This protects against Yellow Fever an infection carried by mosquitoes.
- **HPV vaccine.** Human Papilloma Virus (HPV) causes growths in private parts and cancer in girls and boys.
- **Tetanus and diphtheria vaccines.** Tetanus and diphtheria are bacterial infections that cause very serious disease.
- **Typhoid vaccine.** Typhoid is a serious form of fever which is spread through contaminated food.

These immunisations will be given on different times during the entire duration of the study.

Most of the vaccines will be injected into your upper arm (either right or left side). The typhoid vaccine is an oral vaccine: it is in the form of capsules which are swallowed. You will be given one capsule per day for three alternating days.

You will be asked to give **blood samples**

We will ask you to give blood samples before and after you are given the vaccines. The amount of blood that we will take at each visit is completely safe and will vary between 4 and 27 millilitres (1 teaspoon and 5 and a half teaspoons) at the different visits.

You will be asked to give **stool and urine samples**

At some visits we will ask you for additional stool and urine samples for testing to check whether the worm infections have responded to treatment or (for girls) whether you have become pregnant.

Some of you will be asked to take part in a special **sugar test** of absorption from the intestines

We think that worm infections may make our intestines a little bit leaky, and this might explain some of their effects. We can test this using a special sugar drink. We will ask about 200 people to do this additional test. If you are asked to do this, it will be done about 8 weeks after your first vaccine is given. You will be asked not to eat overnight or in the morning of the test day. Then you will be given a special drink that contains sugars early in the morning. Afterwards you will also be asked to drink a litre of water (equal to two Rwenzori bottles) over the following few hours. All the urine that you pass during the next five hours will be collected. The volume (amount) of urine will be measured and the amount of sugar in it will be tested, to find out how much of the sugar your body has absorbed.

UGANDA VIRUS RESEARCH INSTITUTE ENTEBBE
RESEARCH ETHICS COMMITTEE
APPROVED
05 SEP 2020
P. O. BOX 49, ENTEBBE
UGANDA

MRC/UVRI and LSHTM Uganda Research Unit

MRC Medical Research Council | Uganda Virus Research Institute | LONDON SCHOOL of HYGIENE &TROPICAL MEDICINE

4. What will the blood, stool and urine samples be used for?

The samples will be used to test for infections, including HIV, malaria and worms, to test for anaemia (the strength of your blood) and to test for pregnancy (among girls). The blood samples will also be used to test how your body responds to vaccines.

5. How many people will take part in the study, and how long will it last?

The whole study will enrol 480 people, and the study will last for about two years.

6. What are the risks of participating in this trial?

- You will need to take time off classes during each visit by the study team.
- Taking blood samples is not expected to cause any problem for you, apart from the discomfort or pain, bruising or bleeding, redness at the place where the needle goes into the skin, but this will go away in a few days.
- During the sugar test, you will be asked not to eat overnight and during the morning of the test so you may get hungry (although water and a snack will be provided). This may make you feel a little sick and stools may become a bit loose.
- If you are a girl, even if you know you could not be pregnant, we will do a pregnancy test. We have to be quite sure. You must as well promise that you can avoid getting pregnant while taking part in this study.
- Treatment of Bilharzia with praziquantel may make you feel dizzy or sick, give you abdominal pain or diarrhoea, or occasionally cause an itchy rash. The research team will have medicines available to help you if you have a strong reaction to the treatment.
- All the study vaccines are known to be safe. However, very occasionally even approved vaccines may cause a serious reaction, such as an allergic reaction. The research doctors and nurses will be available to help if this happens.

7. What are the benefits of taking part in this trial?

The treatment for Bilharzia is likely to be good for you, whichever group you are put in. The vaccines are likely to help you by protecting you from infectious diseases.

Also, you will be helping us to find out why vaccines sometimes don't work so well in countries like Uganda, and whether vaccines work better if worms are treated first. This may help other people in the future.

UGANDA VIRUS RESEARCH INSTITUTE ENTEBBE
RESEARCH ETHICS COMMITTEE
APPROVED
05 SEP 2020
P. O. BOX 49, ENTEBBE
UGANDA

8. What is the cost of taking part in the trial?

There is no cost to participate in this trial. You will receive a soft drink and the gift of a pen or other simple school material on days when blood samples are drawn, and the gift of a T-shirt at the end of the study when everything is done, in appreciation of your contribution to the work.

9. What happens if I refuse to take part?

It is very important for you to know that you do not have to take part in the research, the choice is yours. No-one will be upset if you decide not to take part. If you agree to take part and later decide that you do not want to take part anymore, that is also okay.

10. What happens if something goes wrong?

The researchers will make every effort to ensure your safety and well-being. MRC/UVRI and LSHTM has a specialist insurance policy in place which would help to pay for treatment if any harm came to you as a result of taking part in the research.

11. Who will have access to my information and samples from this research?

All our research records and samples are stored securely in rooms with restricted access and on password protected computers. Only research staff trained to keep the information confidential will have access to the them. Your name will be removed from the records, so no-one will be able to find out information about you from our records.

12. Who has reviewed the trial?

This trial has been reviewed by the Uganda National Council for Science and Technology, The Uganda National Drug Authority, the Uganda Virus Research Institute Research and Ethics Committee the ethics committee of the London School of Hygiene and Tropical Medicine in the United Kingdom.

You can find out more about the study at any time by asking one of the members of the research team. You may also contact Professor Alison Elliott (telephone: 0417 704000) who is in charge of the study, or the Chairperson of the Ethics Committee for the Uganda Virus Research Institute on 0414 321962.

UGANDA VIRUS RESEARCH INSTITUTE ENTEBBE
RESEARCH ETHICS COMMITTEE
APPROVED
05 SEP 2020
P. O. BOX 49, ENTEBBE
UGANDA

MRC/UVRI and LSHTM Uganda Research Unit

MRC Medical Research Council | Uganda Virus Research Institute | LONDON SCHOOL of HYGIENE & TROPICAL MEDICINE

Population differences in vaccine responses: the effect of intensive treatment for schistosomiasis on response to vaccines among island adolescents

Short Title: POPVAC Project A

Participant Consent

A copy of this form will be given to you. Please keep a copy of the form because it contains the information that was discussed with you and you may want to look at it again.

AGREEMENT TO TAKE PART:

I have read and understood (or been read to and understand) the information sheet for this study. My questions have been answered. I understand that taking part in the study is voluntary. I understand that at any time I may withdraw from this study without giving a reason. I agree to participate in this study.

Name: ______________________________ **PVA ID:** |A|___|___|___|___|___|

(please write your name in capital letters here if you agree)

Signature: ______________________________ **Date:**__________________

(please sign or write your name here if you agree; or use a thumbprint)

What if I have any questions?

If you have any questions about your participation in this study, please feel free to ask any member of the research team at any time. If you prefer, you may speak to the principal investigator for this study (Professor Alison Elliott, telephone 0417 704000).

What if we want to ask someone independent anything about this research, or have any questions about your rights as a research participant? You may speak with the Chairman of the Science and Ethics Committee at Uganda Virus Research Institute on 0414-321962.

UGANDA VIRUS RESEARCH INSTITUTE ENTEBBE
RESEARCH ETHICS COMMITTEE
APPROVED
VALID UNTIL
05 SEP 2020
P. O. BOX 49, ENTEBBE
UGANDA

POPVAC Project A, consent for emancipated minors v1.1, 1st July 2019 |Page **5** of **6**

PVA ID |A| ___ | ___ | ___ | ___ |

Witness:

I have read the participant information sheet and the consent statement above

to:.. (PRINT NAME OF PARTICIPANT) in a language which he/she understands. I believe that he/she gives consent to take part in the study.

....	....	....
**Witness name**	**Signature**	**Date**

Witness required only for those using a thumb print instead of the final signature, or unable to read the information and consent form, or if the person taking consent does not speak the participant's language. The witness must not be a member of the research staff or a study participant. The witness must be present for the whole consent process.

Person taking the consent:

....	....	....
**Researcher name**	**Signature**	**Date**

Comment:

UGANDA VIRUS RESEARCH INSTITUTE ENTEBBE
RESEARCH ETHICS COMMITTEE
APPROVED
05 SEP 2020
P. O. BOX 49, ENTEBBE
UGANDA

MRC/UVRI and LSHTM Uganda Research Unit

Medical Research Council | Uganda Virus Research Institute | LONDON SCHOOL of HYGIENE &TROPICAL MEDICINE

Population differences in vaccine responses: the effect of intensive treatment for schistosomiasis on response to vaccines among island adolescents

Short Title: POPVAC Project A

Information Sheet for parents/guardians

We are inviting your child to join in some research which is being done by a team from Uganda Virus Research Institute (UVRI), working together with colleagues from the London School of Hygiene and Tropical Medicine, UK. This form will tell you about the research. If you have any questions, please ask the person who is telling you about this research. Then you can decide whether you want your child to take part or not. There is no need for your child to take part unless you really want him/her to do so. If you agree for your child to take part, you will need to sign the consent form to show us that you are happy for him/her to do so. If you decide that you do not want your child to take part, do not sign the consent form.

The people leading this research study are Professor Alison Elliott and Dr Ludoviko Zirimenya.

1. What this research is about, and the reason for doing this research?

Vaccines are a very important tool for preventing infectious diseases. They have saved many lives. Vaccines are usually made from a weakened or killed strain of the bacteria or viruses that cause infectious diseases, or from a part of the bacteria or viruses. Vaccines are designed to help our body's defence system to recognise infectious diseases before we actually meet them, so that we can defeat them more easily. **However, some vaccines seem to work less well in hot countries, near the equator (such as Uganda), than in cooler countries (such as the United Kingdom, the UK).** We want to find out why this is so.

Worm infections are much more common in warm countries (such as Uganda) than in cooler countries (such as the UK). Bilharzia (schistosomiasis) is a worm infection which is very common in Koome islands. Almost everyone in Koome has Bilharzia. Worms can live in our bodies for many years. To do this they have to be able to switch off some of our body's defence systems so that they will not be killed. **We think that this "switching off" of defence systems may also prevent some vaccines from working well.** If this is true then treating worms before giving vaccines might result in better responses to the vaccines.

UGANDA VIRUS RESEARCH INSTITUTE ENTEBBE RESEARCH ETHICS COMMITTEE
APPROVED
05 SEP 2020
P. O. BOX 49, ENTEBBE UGANDA

Light infections with Bilharzia may not be noticed but heavy infections can cause serious damage, especially to the liver. That is why the Ugandan government usually gives treatment for Bilharzia once a year in Ugandan schools. This usually reduces the number of Bilharzia worms but does not get rid of them. **We want to find out whether treating Bilharzia much more frequently (a total of 7 times in a year) can get rid of it completely and whether this will improve vaccine responses.**

2. Why has my child been asked to take part in this study?

Your child has been asked because he/she is attending primary school in Koome islands, in primary 1 to 6. Bilharzia infection is often heaviest in people of primary school age. Also, the government of Uganda offers vaccines to your child's age group that will help to protect them from infectious diseases later on. So it is important to know whether Bilharzia can affect these vaccines, and whether treating Bilharzia effectively is helpful.

3. What is going to happen in this research study?

Your child will have **a health check-up**

If you want your child to take part in this study, we will first check your child's health, and take stool samples, urine samples and some blood from a vein in your child's arm and do some tests. These will include tests for infections including HIV, malaria and worms, tests for anaemia (the strength of blood in your child's body), and tests for pregnancy if your child is a girl.

If everything is okay then we will enrol your child in the study. If something is not okay then we will either give your child the treatment that he/she needs, or tell you what to do.

Your child will be put into one of the two **Bilharzia treatment** groups

When your child is enrolled in the study we will put your child into one of two groups.

- One group will be treated for Bilharzia using praziquantel (the recommended medicine for Bilharzia) seven times in the year, and an eighth time after the end of the study.
- The other group will be treated for Bilharzia using praziquantel once in the year, and a second time after the end of the study.

The choice of groups will be done using a code generated by computer. This works so that your child is put into one group or the other by luck – this is like a lottery.

UGANDA VIRUS RESEARCH INSTITUTE ENTEBBE
RESEARCH ETHICS COMMITTEE
APPROVED
05 SEP 2020
P. O. BOX 49, ENTEBBE
UGANDA

Your child will be immunised with **several vaccines.** These are the vaccines:

- BCG vaccine. This is intended to protect against tuberculosis. Tuberculosis is very common in Uganda. Many people receive the BCG vaccine as a baby. BCG can be given again at school age and may help to protect against tuberculosis later on although the level of protection varies a lot between countries. We do not know how well it works in school children in Uganda.
- Yellow fever vaccine. This protects against Yellow Fever. Yellow fever is a virus carried by mosquitoes. Yellow fever disease affects the liver and causes fever and jaundice (yellow eyes). Outbreaks occur in Uganda and neighbouring countries from time to time – an outbreak is when a group of people falls sick around the same time.
- HPV vaccine. Human Papilloma Virus (HPV) causes warts. As well, some strains of HPV can cause cancer in the genital areas, especially on the cervix (the opening of the womb) in girls. HPV can also cause cancer of the penis in boys, and other cancers. The HPV vaccine reduces the risk of infection with dangerous HPV strains that cause cancer. In this way it reduces the risk of cancers. The Ugandan government recently started offering this vaccine to girls in primary 4. We will give this vaccine to you if you are a girl (in any class) and you have not received it already. We will also give this vaccine to boys because it can protect them against some cancers too.
- Tetanus and diphtheria vaccines. Tetanus and diphtheria are bacterial infections. The tetanus and diphtheria bacteria produce chemicals called toxins which cause diseases. Tetanus bacteria infect deep wounds and produce tetanus toxin which causes very serious muscle spasms. Tetanus can affect young babies if the wound where the umbilical cord is cut becomes infected. Immunising young women can protect their future babies too. Diphtheria causes a very serious throat infection. Tetanus and diphtheria immunisation is given to babies but booster immunisation is recommended by the Uganda government for school children also.
- Typhoid vaccine. Typhoid is a serious form of fever which is spread through contaminated food. Outbreaks occur in Uganda. Immunisation against typhoid can prevent this illness.

These immunisations will be given at three different time points. BCG will be given first. HPV vaccine, oral typhoid vaccine and yellow fever vaccine will be given four weeks later. A second dose of HPV vaccine and first dose of Tetanus/diphtheria vaccine will be given at 28 weeks after the BCG immunisation.

Most of the vaccines will be injected into your child's upper arm (either right or left). This will be a bit painful, as for any injection of medicine that you may have had, and will feel a bit sore over the next week or so. The BCG vaccine is likely to form a small swelling and then an ulcer

UGANDA VIRUS RESEARCH INSTITUTE ENTEBBE
RESEARCH ETHICS COMMITTEE
APPROVED
VALID UNTIL
05 SEP 2020
P. O. BOX 49, ENTEBBE
UGANDA

which may take quite some time (perhaps two to five months) to heal. The typhoid vaccine is an oral vaccine: it is in the form of capsules which are swallowed. Your child will be given one capsule per day for three alternating days.

Your child will be asked to give **blood samples**

To test your child's response to the vaccines we will ask your child to give blood samples before and after they are given the vaccines. The amount of blood that we will take at each visit is completely safe and will vary between 4 and 27 millilitres (1 teaspoon and 5 and a half teaspoons) at the different visits. In total we will ask your child to give blood samples at 7 different times during the study. The study will last for about two years.

Your child will be asked to give **stool and urine samples**

At some visits we will ask your child for additional stool and urine samples for testing to check whether the worm infections have responded to treatment or (for girls) whether your child has become pregnant.

Some of the children will be asked to take part in a special **sugar test** of absorption from the intestines

We think that worm infections may make our intestines a little bit leaky, and this might explain some of their effects. We can test this using a special sugar drink. We will ask about 200 children to do this additional test. If your child is asked to do this, it will be done about 8 weeks after the first vaccine is given. He/she will be asked not to eat overnight or in the morning of the test day. Then he/she will be given a special drink that contains sugars early in the morning. Afterwards he/she will also be asked to drink a litre of water (equal to two Rwenzori bottles) over the following few hours. All the urine that he/she passes during the next five hours will be collected. The volume (amount) of urine will be measured and the amount of sugar in it will be tested, to find out how much of the sugar his/her body has absorbed.

If you do not want your child to take part in the study, to have blood, urine and stool samples taken or to receive the treatment and vaccines, you can say no to your child's taking part in this study.

4. What will the blood, stool and urine samples be used for?

The samples will be used to test for infections, including HIV, malaria and worms, to test for anaemia (the strength of your child's blood) and to test for pregnancy (among girls). The blood samples will also be used to test how your child's body responds to vaccines.

UGANDA VIRUS [illegible] INSTITUTE ENTEBBE
RESEARCH ETHICS COMMITTEE
[illegible]
05 SEP 2020
P. O. BOX 49, ENTEBBE
UGANDA

5. How many people will take part in the study, and how long will it last?

The whole study will enrol 480 people, and the study will last for about two years.

6. What are the risks of participating in this trial?

Your child's time

Your child will need to take time off classes during each visit by the study team. There will be 12 visits altogether. Each visit will usually take about 30 minutes, but the first visit may take about two hours to give time for everything to be explained, and for a thorough check-up. The research team will work with teachers to avoid disturbing classes too much.

Blood samples

Taking blood samples is not expected to cause any problem for your child, apart from the discomfort or pain, bruising or bleeding, redness at the place where the needle goes into the skin, but this will go away in a few days. There is a very small chance that your child may get an infection or some swelling at this place – this almost never happens. Some people faint when their blood is taken. The person taking the blood will do all they can to prevent these things from happening.

Sugar test

During the sugar test your child will be asked not to eat overnight and during the morning of the test so he/she may get hungry (although water and a snack will be provided). Most people have no problems with this test although a few may feel a little sick and stools may become a bit loose.

Pregnancy

We do not know how some of these vaccines might affect a developing baby if they were given to someone who was pregnant. That is why we will do a pregnancy test, if your child is a girl, even if she knows she could not be pregnant. We have to be quite sure. We will test for pregnancy at the beginning of the study and on each immunisation day. We will not enrol your child in the study if she is pregnant at the start. We will not give your child the vaccines if she falls pregnant later.

Bilharzia treatment

Treatment of Bilharzia with praziquantel may make your child feel dizzy or sick, give your child abdominal pain or diarrhoea, or occasionally cause an itchy rash. This is most likely caused by

UGANDA VIRUS RESEARCH INSTITUTE ENTEBBE
RESEARCH ETHICS COMMITTEE
APPROVED
VALID UNTIL
05 SEP 2020
P. O. BOX 49, ENTEBBE
UGANDA

the body's response to the worms as they are being killed by the medicine. The research team will have medicines available to help your child if he/she has a strong reaction to the treatment.

Immunisations

All the vaccines that we will give have been used to protect very large numbers of people and are known to be safe. They are not expected to cause any major problems for your child. As mentioned, the BCG vaccine is expected to cause an ulcer and to heal slowly, leaving a scar. The injections will cause some pain at the time of injection and your child's arm will feel a bit sore for a day or two. Some people develop flu-like symptoms including headache and fever for a day or two. It is fine for your child to take painkillers like paracetamol (Panadol) for these symptoms. The typhoid vaccine, which your child will swallow as capsules, is not expected to cause your child any problem at all, although your child may experience some stomach pain, feeling sick, vomiting and (rarely) rash.

Very occasionally any vaccine can cause a serious reaction, such as an allergic reaction. The research doctors and nurses will be available to help if this happens.

7. What are the benefits of taking part in this trial?

The treatment for Bilharzia is likely to be good for your child, whichever group he or she is put in. The vaccines are likely to help your child by protecting him/her from infectious diseases.

Also, you and your child will be helping us to find out why vaccines sometimes don't work so well in countries like Uganda, and whether vaccines work better if worms are treated first. This may help other people in the future.

8. What is the cost of taking part in the trial?

There is no cost to participate in this trial. We will reimburse you (the parents) for the time you spend at meetings at school, 20,000/= (twenty thousand shillings) for each visit. Your child will receive a soft drink and the gift of a pen or other simple school material on days when blood samples are drawn, and the gift of a T-shirt at the end of the study when everything is done, in appreciation of his/her contribution to the work.

9. What happens if I refuse for my child to take part?

It is very important for you to know that your child does not have to take part in the research, the choice is yours and your child's. No-one will be upset if your child decides not to take part. The teachers will not be upset and the research team will not be upset. If you agree for your child to take part and later decide that you do not want him/her to take part anymore, that is also okay. Whatever happens, your child will still be able to receive the treatment for Bilharzia

UGANDA VIRUS RESEARCH INSTITUTE
RESEARCH ETHICS COMMITTEE
APPROVED
VALID UNTIL
05 SEP 2020
P. O. BOX 49, ENTEBBE
UGANDA

and the immunisations for HPV and tetanus and diphtheria when they are provided by the government, if you wish.

10. Who will be able to see the results of tests done on my child's samples?

The research team will keep your child's results private. Only members of the research team will be able to see results and to know that they belong to your child. Your child's samples will be given a special code so that anyone who is working on them in research laboratories will not know they came from your child.

11. What happens if something goes wrong?

You and your child will be making an important contribution to medical research. The researchers will make every effort to ensure your child's safety and well-being. MRC/UVRI and LSHTM has a specialist insurance policy in place which would help to pay for treatment if any harm came to your child as a result of taking part in the research.

12. Who will have access to information from this research?

All our research records are stored securely in rooms with restricted access and on password protected computers. Only research staff trained to keep the information confidential will have access to the records. Your child's name will be removed from the records, so no-one will be able to find out information about your child from our records. The people who may review your child's records include Research Ethics Committees (Uganda Virus Research Institute Research Ethics Committee and the London School of Hygiene & Tropical Medicine Ethics Committee) the Uganda National Council Science and Technology, Study Monitors, Sponsor and the Uganda National Drug Authority. These organisations are there to ensure that your child's rights are protected and that the research is conducted properly and safely.

13. Who has reviewed the trial?

This trial has been reviewed by the Uganda National Council for Science and Technology, the Uganda Virus Research Institute Research and Ethics Committee, the ethics committee of the London School of Hygiene and Tropical Medicine in the United Kingdom. The Uganda National Drug Authority, which regulates the use of all medicines in Uganda, has granted permission to use the medicines and vaccines needed for this clinical trial.

You can find out more about the study at any time by asking one of the members of the research team. You may also contact Professor Alison Elliott (telephone: 0417 704000) who is in charge of the study, or the Chairperson of the Ethics Committee for the Uganda Virus Research Institute on 0414 321962.

UGANDA VIRUS RESEARCH INSTITUTE ENTEBBE
RESEARCH ETHICS COMMITTEE
APPROVED
VALID UNTIL
05 SEP 2020
P. O. BOX 49, ENTEBBE
UGANDA

MRC/UVRI and LSHTM Uganda Research Unit

MRC Medical Research Council | Uganda Virus Research Institute | LONDON SCHOOL of HYGIENE &TROPICAL MEDICINE

Population differences in vaccine responses: the effect of intensive treatment for schistosomiasis on response to vaccines among island adolescents

Short Title: POPVAC Project A

Parent/guardian consent

A copy of this form will be given to you. Please keep a copy of the form because it contains the information that was discussed with you and you may want to look at it again.

Participant (child) name: ________________________________ **PVA ID|A|___|___|___|___|**
(please write your child's name in capital letters here if you agree)

AGREEMENT BY PARENT OR GUARDIAN:

I have read and/or been fully explained the information sheet concerning my child's participation in this study and I understand what will be required if he or she takes part. I understand that my child's participation is voluntary. My questions concerning this study have been answered. I understand that at any time, I may withdraw my child from this study without giving a reason and without affecting his or her entitlement to government health care. I agree for my child to take part in this study.

Name: ____
(please write your name in capital letters here if you agree)

Signature: ___________________________________ **Date:**________________
(please sign or write your name here if you agree; or use a thumbprint)

What if I have any questions?
If you have any questions about your participation in this study, please feel free to ask any member of the research team at any time. If you prefer, you may speak to the principal investigator for this study (Professor Alison Elliott, telephone 0417 704000).

What if we want to ask someone independent anything about this research, or have any questions about your rights as a research participant? You may speak with the Chairman of the Science and Ethics Committee at Uganda Virus Research Institute on 0414 321962.

UGANDA VIRUS RESEARCH INSTITUTE ENTEBBE
RESEARCH ETHICS COMMITTEE
APPROVED
05 SEP 2020
P. O. BOX 49, ENTEBBE
UGANDA

PVA ID|A|___|___|___|___|

Witness:

I have read the participant information sheet and the assent statement above

to:.. (PRINT NAME OF PARTICIPANT) in a language which he/she understands. I believe that he/she gives consent for his/her child to take part in the study.

....	....	....
**Witness name**	**Signature**	**Date**

Witness required only for those using a thumb print instead of the final signature, or unable to read the information and consent form, or if the person taking consent does not speak the participant's language. The witness must not be a member of the research staff or a study participant. The witness must be present for the whole consent process.

Person taking the consent:

....	....	....
**Researcher name**	**Signature**	**Date**

Comment:

UGANDA VIRUS RESEARCH INSTITUTE ENTEBBE
RESEARCH ETHICS COMMITTEE
APPROVED
VALID UNTIL
05 SEP 2020
P. O. BOX 49, ENTEBBE
UGANDA

UGANDA VIRUS RESEARCH INSTITUTE ENTEBBE
RESEARCH ETHICS COMMITTEE
APPROVED
05 SEP 2020
P. O. BOX 49, ENTEBBE
UGANDA
