# Supplementary material for: Effect of intensive treatment for schistosomiasis on immune responses to vaccines among rural Ugandan island adolescents: randomised controlled trial protocol A for the ‘POPulation differences in VACcine responses’ (POPVAC) programme
Source: BMJ Open. 2021 Feb 16;11(2):e040426. doi: 10.1136/bmjopen-2020-040426 (PMC7888376; doi:10.1136/bmjopen-2020-040426)
Supplement: Author's manuscript [file bmjopen-2020-040426.draft_revisions.pdf]

BMJ Open is committed to open peer review. As part of this commitment we make the peer review history of every article we publish publicly available.

When an article is published we post the peer reviewers' comments and the authors' responses online. We also post the versions of the paper that were used during peer review. These are the versions that the peer review comments apply to.

The versions of the paper that follow are the versions that were submitted during the peer review process. They are not the versions of record or the final published versions. They should not be cited or distributed as the published version of this manuscript.

BMJ Open is an open access journal and the full, final, typeset and author-corrected version of record of the manuscript is available on our site with no access controls, subscription charges or pay-per-view fees (http://bmjopen.bmj.com).

If you have any questions on BMJ Open's open peer review process please email info.bmjopen@bmj.com

BMJ Open

The effect of intensive treatment for schistosomiasis on immune responses to vaccines among rural Ugandan island adolescents: randomised controlled trial protocol A for the 'POPulation differences in VACcine responses' (POPVAC) programme

Journal:	*BMJ Open*
Manuscript ID	bmjopen-2020-040426
Article Type:	Protocol
Date Submitted by the Author:	13-May-2020
Complete List of Authors:	Nkurunungi, Gyaviira; Medical Research Council/Uganda Virus Research Institute and London School of Hygiene and Tropical Medicine (MRC/UVRI and LSHTM) Uganda Research Unit, Immunomodulation and Vaccines Programme<br>Zirimenya , Ludoviko ; Medical Research Council/Uganda Virus Research Institute and London School of Hygiene and Tropical Medicine (MRC/UVRI and LSHTM) Uganda Research Unit, Immunomodulation and Vaccines Programme<br>Nassuuna , Jacent; Medical Research Council/Uganda Virus Research Institute and London School of Hygiene and Tropical Medicine (MRC/UVRI and LSHTM) Uganda Research Unit, Immunomodulation and Vaccines Programme<br>Natukunda , Agnes ; Medical Research Council/Uganda Virus Research Institute and London School of Hygiene and Tropical Medicine (MRC/UVRI and LSHTM) Uganda Research Unit, Immunomodulation and Vaccines Programme<br>Kabuubi , Prossy ; Medical Research Council/Uganda Virus Research Institute and London School of Hygiene and Tropical Medicine (MRC/UVRI and LSHTM) Uganda Research Unit, Immunomodulation and Vaccines Programme<br>Niwagaba , Emmanuel ; Medical Research Council/Uganda Virus Research Institute and London School of Hygiene and Tropical Medicine (MRC/UVRI and LSHTM) Uganda Research Unit, Immunomodulation and Vaccines Programme<br>Oduru, Gloria; Medical Research Council/Uganda Virus Research Institute and London School of Hygiene and Tropical Medicine (MRC/UVRI and LSHTM) Uganda Research Unit, Immunomodulation and Vaccines Programme<br>Kabami , Grace ; Medical Research Council/Uganda Virus Research Institute and London School of Hygiene and Tropical Medicine (MRC/UVRI and LSHTM) Uganda Research Unit, Immunomodulation and Vaccines Programme<br>Amongin , Rebecca ; Medical Research Council/Uganda Virus Research Institute and London School of Hygiene and Tropical Medicine (MRC/UVRI and LSHTM) Uganda Research Unit, Immunomodulation and Vaccines Programme<br>Mutebe , Alex ; Medical Research Council/Uganda Virus Research

Institute and London School of Hygiene and Tropical Medicine (MRC/UVRI and LSHTM) Uganda Research Unit, Immunomodulation and Vaccines Programme
Namutebi , Milly ; Medical Research Council/Uganda Virus Research Institute and London School of Hygiene and Tropical Medicine (MRC/UVRI and LSHTM) Uganda Research Unit, Immunomodulation and Vaccines Programme
Zziwa , Christopher ; Medical Research Council/Uganda Virus Research Institute and London School of Hygiene and Tropical Medicine (MRC/UVRI and LSHTM) Uganda Research Unit, Immunomodulation and Vaccines Programme
Amongi, Susan; Medical Research Council/Uganda Virus Research Institute and London School of Hygiene and Tropical Medicine (MRC/UVRI and LSHTM) Uganda Research Unit, Immunomodulation and Vaccines Programme
Ninsiima , Caroline ; Medical Research Council/Uganda Virus Research Institute and London School of Hygiene and Tropical Medicine (MRC/UVRI and LSHTM) Uganda Research Unit, Immunomodulation and Vaccines Programme
Onen, Caroline; Medical Research Council/Uganda Virus Research Institute and London School of Hygiene and Tropical Medicine (MRC/UVRI and LSHTM) Uganda Research Unit, Immunomodulation and Vaccines Programme
Akello , Florence ; Medical Research Council/Uganda Virus Research Institute and London School of Hygiene and Tropical Medicine (MRC/UVRI and LSHTM) Uganda Research Unit, Immunomodulation and Vaccines Programme
Sewankambo, Moses; Medical Research Council/Uganda Virus Research Institute and London School of Hygiene and Tropical Medicine (MRC/UVRI and LSHTM) Uganda Research Unit, Immunomodulation and Vaccines Programme
Kiwanuka, Samuel; Medical Research Council/Uganda Virus Research Institute and London School of Hygiene and Tropical Medicine (MRC/UVRI and LSHTM) Uganda Research Unit, Immunomodulation and Vaccines Programme
Kizindo , Robert ; Medical Research Council/Uganda Virus Research Institute and London School of Hygiene and Tropical Medicine (MRC/UVRI and LSHTM) Uganda Research Unit, Immunomodulation and Vaccines Programme
Kaweesa, James; Vector Control Division, Ministry of Health
Cose , Stephen ; Medical Research Council/Uganda Virus Research Institute and London School of Hygiene and Tropical Medicine (MRC/UVRI and LSHTM) Uganda Research Unit, Immunomodulation and Vaccines Programme; London School of Hygiene and Tropical Medicine Department of Clinical Research
Webb, Emily; London School of Hygiene and Tropical Medicine, MRC Tropical Epidemiology Group, Department of Infectious Disease Epidemiology
Elliott, Alison ; Medical Research Council/Uganda Virus Research Institute and London School of Hygiene and Tropical Medicine (MRC/UVRI and LSHTM) Uganda Research Unit, Immunomodulation and Vaccines Programme; London School of Hygiene and Tropical Medicine Department of Clinical Research

Keywords: Infection control < INFECTIOUS DISEASES, PARASITOLOGY, Public health < INFECTIOUS DISEASES, Immunology < TROPICAL MEDICINE, Epidemiology < TROPICAL MEDICINE, Paediatric infectious disease & immunisation < PAEDIATRICS

SCHOLARONE™
Manuscripts

BMJ

I, the Submitting Author has the right to grant and does grant on behalf of all authors of the Work (as defined in the below author licence), an exclusive licence and/or a non-exclusive licence for contributions from authors who are: i) UK Crown employees; ii) where BMJ has agreed a CC-BY licence shall apply, and/or iii) in accordance with the terms applicable for US Federal Government officers or employees acting as part of their official duties; on a worldwide, perpetual, irrevocable, royalty-free basis to BMJ Publishing Group Ltd ("BMJ") its licensees and where the relevant Journal is co-owned by BMJ to the co-owners of the Journal, to publish the Work in this journal and any other BMJ products and to exploit all rights, as set out in our licence.

The Submitting Author accepts and understands that any supply made under these terms is made by BMJ to the Submitting Author unless you are acting as an employee on behalf of your employer or a postgraduate student of an affiliated institution which is paying any applicable article publishing charge ("APC") for Open Access articles. Where the Submitting Author wishes to make the Work available on an Open Access basis (and intends to pay the relevant APC), the terms of reuse of such Open Access shall be governed by a Creative Commons licence – details of these licences and which Creative Commons licence will apply to this Work are set out in our licence referred to above.

Other than as permitted in any relevant BMJ Author's Self Archiving Policies, I confirm this Work has not been accepted for publication elsewhere, is not being considered for publication elsewhere and does not duplicate material already published. I confirm all authors consent to publication of this Work and authorise the granting of this licence.

For peer review only

The effect of intensive treatment for schistosomiasis on immune responses to vaccines among rural Ugandan island adolescents: randomised controlled trial protocol A for the 'POPulation differences in VACcine responses' (POPVAC) programme

Gyaviira Nkurunungi[1,¶,*], Ludoviko Zirimenya[1,¶], Jacent Nassuuna[1,¶], Agnes Natukunda[1,¶], Prossy N Kabuubi[1], Emmanuel Niwagaba[1], Gloria Oduru[1], Grace Kabami[1], Rebecca Amongin[1], Alex Mutebe [1], Milly Namutebi[1], Christopher Zziwa[1], Susan Amongi[1], Caroline Ninsiima[1], Caroline Onen[1], Florence Akello[1], Moses Sewankambo[1], Samuel Kiwanuka[1], Robert Kizindo[1], James Kaweesa[2], Stephen Cose[1,3], Emily L Webb[4], Alison M Elliott[1,3] **for the POPVAC trial team**

[1]Immunomodulation and Vaccines Programme, Medical Research Council/Uganda Virus Research Institute and London School of Hygiene and Tropical Medicine (MRC/UVRI and LSHTM) Uganda Research Unit, Entebbe, Uganda

[2]Vector Control Division, Ministry of Health, Kampala, Uganda

[3]Department of Clinical Research, London School of Hygiene and Tropical Medicine, London, United Kingdom

[4]MRC Tropical Epidemiology Group, Department of Infectious Disease Epidemiology, London School of Hygiene and Tropical Medicine, London, United Kingdom

¶These authors contributed equally

***Correspondence:** Gyaviira Nkurunungi; gyaviira.nkurunungi@lshtm.ac.uk

For peer review only - http://bmjopen.bmj.com/site/about/guidelines.xhtml

Abstract

Introduction

Several licensed and investigational vaccines have lower efficacy, and induce impaired immune responses, in low-income versus high-income countries and in rural, versus urban, settings. Understanding these population differences is essential to optimising vaccine effectiveness in the tropics. We suggest that repeated exposure to and immunomodulation by chronic helminth infections is crucial.

Methods and analysis

We have designed an individually randomised, parallel group trial of intensive versus standard praziquantel (PZQ) intervention against schistosomiasis, to determine effects on vaccine response outcomes among school-going adolescents (9 to 17 years) from rural *Schistosoma mansoni* (*Sm*)-endemic Ugandan islands. Vaccines to be studied comprise BCG on day ‘zero’; yellow fever, oral typhoid and Human Papilloma Virus (HPV) vaccines at week 4; and HPV and Tetanus/diphtheria booster vaccine at week 28. The intensive arm will receive PZQ doses three times, each two weeks apart, before BCG immunisation, followed by a dose at week 8 and quarterly thereafter. The standard arm will receive PZQ at week 8 and week 52. We expect to enrol 480 participants, with 80% *Sm* infected at the outset.

Primary outcomes are BCG-specific IFN-γ ELISpot responses eight weeks after BCG immunisation and for other vaccines, antibody responses to key vaccine antigens at four weeks after immunisation. Secondary analyses will determine effects of intensive anthelminthic treatment on correlates of protective immunity, on vaccine response waning, on priming vs boosting immunisations, and on *Sm* infection status and intensity. Exploratory immunology assays using archived samples will enable assessment of mechanistic links between helminths and vaccine responses.

Ethics and dissemination

Ethics approval has been obtained from relevant Ugandan and UK ethics committees. Results will be shared with Uganda Ministry of Health, relevant district councils, community leaders and study participants. Further dissemination will be done through conference proceedings and publications.

Trial registration

Current Controlled Trials identifier: ISRCTN60517191.

For peer review only

Article summary

Strengths and limitations of this study

- This will be the first well-powered intervention study to investigate effects of schistosomiasis treatment on vaccine responses in adolescents.
- Effects on both live-attenuated and inert vaccines will be studied.
- Our strong immunoepidemiological design and nested immunological studies will address specific hypotheses regarding pathways of effects.
- The sample archives developed will provide a major asset for exploration of new leads arising from this hypothesis-driven work, or for an alternative, "systems biology" approach investigating (for example) transcriptome, microbiome and virome.
- Even with intensive anthelminthic intervention, it may be difficult to "successfully" treat *Schistosoma* infection in our endemic setting due to re-infections; however, we still expect a substantial difference in intensity between the two trial arms.

Word count

3066

Keywords

Vaccine; Schistosomiasis; Praziquantel; Immunization

For peer review only

Introduction

Vaccine-specific immune responses are often impaired, and vaccine efficacy lower, in tropical low-income countries (LICs) compared to temperate high-income countries and in rural, compared to urban, LIC settings.[1-8] This has been recognised for both live vaccines (such as BCG,[2 3 5 9] polio,[1] yellow fever[4] vaccines) and non-live vaccines (such as influenza[10] and tetanus[11]). Investigational malaria[7] and viral-vectored tuberculosis[6] and Ebola[12] vaccines are also affected. Previous exposure to the target pathogen (or related organisms) may mask the benefit of the vaccine.[13 14] However, pre-vaccination exposure does not explain UK-Senegal differences in Ebola trial vaccine-specific responses in healthy adults,[12] as the target organism is rare. Therefore, “environmental sensitisation” may play an important role.[5]

A long-held hypothesis is that parasites, particularly helminths, modulate vaccine responses through profound pre- and post-immunisation bystander effects on immunological activation and regulation.[15-17] Helminths might also impact vaccines responses through interactions with the complex ecosystem of mammalian gut bacteria, fungi, protozoa and viruses (the“trans-kingdom” concept[18] detailed elsewhere in this issue). Helminth-induced gut mucosa damage, the associated translocation of microbial products into the systemic circulation[19-21] and systemic immune activation or regulation mediated by microbial products might contribute to modulation of responses to vaccines and other infections.

Helminth-mediated modulation of vaccine responses has not been substantiated in human populations. No well-powered trials have been conducted to evaluate reversibility of their effects. In animal models, helminths generally impair priming and accelerate waning of vaccine responses, although effects vary with helminth species, vaccine type and the timing of infection and immunisation.[22] Most observational studies in humans also suggest suppressed or biased responses during helminth infection, especially during systemic infections, such as schistosomiasis and the filariases. There is modest evidence that treating geohelminths in humans improves responses to BCG[23 24] or oral cholera vaccine[25] and we found that schistosomiasis treatment improved the measles-booster response in pre-school children.[26] There is therefore a strong case for a comprehensive assessment of the effects of helminths and their treatment on vaccine responses.

The extent to which helminths and related “trans-kingdom” mediators causally and reversibly impact immunological characteristics associated with vaccine responses may best be determined by intervention studies. This trial protocol A of the ‘Population differences in Vaccine responses” programme (POPVAC A; Current Controlled Trials identifier: ISRCTN60517191) has been designed to evaluate the effect of *Schistosoma mansoni* and its treatment on vaccine responses. This study is

For peer review only - http://bmjopen.bmj.com/site/about/guidelines.xhtml

one of three parallel trials whose designs and cross-cutting analyses are described separately in this issue.

For peer review only - http://bmjopen.bmj.com/site/about/guidelines.xhtml

Hypothesis

The overarching goal of the POPVAC programme is to understand population differences in vaccine responses in Uganda, in order to identify strategies through which vaccine effectiveness can be optimised for the low-income, tropical settings where they are especially needed. For this Trial A, we focus on the hypothesis that *Schistosoma mansoni infection suppresses responses to unrelated vaccines; and that this effect can be reversed, at least in part, by intensive praziquantel treatment intervention.*

Objective

To determine whether there are reversible effects of chronic *Schistosoma mansoni* infection on vaccine response in adolescents, using an intervention study.

For peer review only

For peer review only - http://bmjopen.bmj.com/site/about/guidelines.xhtml

Methods and analysis

Setting and participants

SPIRIT reporting guidelines[27] have been used. We will conduct an individually randomised, parallel group trial of intensive versus standard intervention against schistosomiasis (described below) in the *S. mansoni*-endemic Koome islands of Lake Victoria, Mukono district, Uganda.[28] We aim to enroll 480 participants, randomising 240 to each intervention arm. The study cohort will recruit participants aged 9 to 17 years in primary school years 1 to 6. Adolescents[29] in this study setting bear a heavy parasite burden.[30] In addition, this age-group is a target group for vaccines against sexually transmitted infections (currently human papilloma virus [HPV] – in future, it is hoped, for vaccines against HIV) and for booster immunisations.

Recruitment criteria

Inclusion criteria

i. Attending the selected school and planning to continue to attend the school for the duration of the study
ii. Aged 9 to 17 years and enrolled in primary 1 to 6 (to avoid primary leaving examinations in late year 7, and loss to follow up of children leaving after primary 7)
iii. Written informed assent by participant and consent by parent or guardian
iv. Females agree to avoid pregnancy for the duration of the trial
v. Willing to provide locator information and to be contacted during the course of the trial
vi. Able and willing (in the investigator's opinion) to comply with all the study requirements

Exclusion criteria

i. Clinically significant history of immunodeficiency (including HIV), cancer, cardiovascular disease, gastrointestinal disease, liver disease, renal disease, endocrine disorder and neurological illness
ii. History of serious psychiatric condition or disorder
iii. Moderate or severe acute illness characterised by any of the following symptoms: fever, impaired consciousness, convulsions, difficulty in breathing, vomiting; or as otherwise determined by the attending project clinician.
iv. Concurrent oral or systemic steroid medication or the concurrent use of other immunosuppressive agents within 2 months prior to enrolment
v. History of allergic reaction to immunisation or any allergy likely to be exacerbated by any component of the study vaccines including egg or chicken proteins

For peer review only - http://bmjopen.bmj.com/site/about/guidelines.xhtml

vi. History of previous immunisation with Yellow Fever (YF), oral typhoid or Human Papillomavirus (HPV) vaccine; previous immunisation with BCG or Tetanus and diphtheria vaccine (Td) at age ≥5 years

vii. Tendency to develop keloid scars

viii. Haemoglobin less than 82g/L

ix. Positive HIV serology

x. Positive pregnancy test

xi. Female currently lactating, confirmed pregnancy or intention to become pregnant during the trial period

xii. Use of an investigational medicinal product or non-registered drug, live vaccine, or medical device other than the study vaccines for 30 days prior to dosing with the study vaccine, or planned use during the study period

xiii. Administration of immunoglobulins and/or any blood products within the three months preceding the planned trial immunisation date

Further information on recruitment criteria can be found in Supplementary information.

Interventions

We will individually randomise participants to intensive or standard praziquantel (PZQ) treatment, in a 1:1 ratio. The intensive arm will receive three doses of PZQ (approximately 40mg/kg, assessed by height pole) each two weeks apart prior to the first immunisation (the last of these 2-3 weeks before immunisation), followed by PZQ at 8 weeks (after primary endpoint sampling) and thereafter quarterly PZQ (approximately; timings adjusted to accommodate school terms) during follow up. The standard arm will receive their first dose of PZQ at week 8 (after immunisation and after primary endpoint sampling) and a second dose at week 52 (to conform to Uganda Ministry of Health policy, which is annual treatment) (**Figure 1**). No placebo will be used in this trial because all participants will be treated (albeit at different frequencies) and participants are unlikely to seek additional treatments outside the trial schedule: praziquantel treatment is not popular because of the recognised (although temporary) adverse effects (described in Supplementary information).

Randomisation and allocation to treatment arm

A randomisation code will be generated by an independent statistician using a randomly permuted block size. At enrolment, eligibility criteria will be checked and eligible participants will be allocated sequentially to the next randomisation number. The randomisation code will be kept securely by the trial statistician and made available only to those responsible for providing or preparing the trial

For peer review only

For peer review only - http://bmjopen.bmj.com/site/about/guidelines.xhtml

177 interventions. A second copy will be held by a data manager or statistician not otherwise involved in
178 the trial at the MRC/UVRI and LSHTM Uganda Research Unit.

179 Implementation will be done by a clinician using sequentially numbered opaque sealed envelopes. A
180 set of envelopes will be prepared, labelled with the randomisation numbers and containing a card
181 indicating the allocation (to intensive or standard treatment). After the next randomisation number
182 in the sequence has been allocated, the envelope bearing that number will be opened to reveal the
183 allocation.

184 ***Blinding***

185 Clinicians and participants will not be blinded to the treatment allocation since they will not
186 participate in outcome ascertainment; only immunology laboratory staff who are assessing trial
187 outcomes will be blinded.

188 ***Immunisations***

189 We will study a portfolio of licensed vaccines (live and inert, oral and parental, priming and boosting)
190 expected to be beneficial (in some cases, already given) to adolescents in Uganda. Our schedule
191 (**Table 1,** supplementary **Table S1**) will comprise three main immunisation days (week 0, week 4 and
192 week 28). Additional HPV immunisation will be provided for girls aged 14 years or above, and a
193 second Tetanus/diphtheria boost will be given after completion of the study, to accord with the
194 national Expanded Programme on Immunisation (EPI) routines, but the response to these will not
195 specifically be assessed. Further rationale for the selection of vaccines is detailed in the
196 Supplementary information.

197

Table 1. Immunisation schedule

	Immunisation week 0	Immunisation week 4	[Immunisation week 8]	Immunisation week 28	[Immunisation week 52]
**Live vaccines**	BCG vaccination / re-vaccination[1]	Yellow fever (YF-17D)<br>Oral typhoid (Ty21a)			
**Non-live vaccines**		HPV prime	HPV boost for girls aged ≥14 years[2,3]	HPV boost and Tetanus/ diphtheria (Td) boost	Tetanus/ diphtheria (Td) boost[3,4]

1. Prior BCG status may vary (data on history and documentation of prior BCG, and presence of a BCG scar, will be documented although these approaches have limitations for determining BCG status)
2. The National EPI programme recommends three doses of HPV vaccine for older girls
3. These doses will be given to comply with guidelines but outcomes specifically relating to these doses will not be assessed
4. Priming by immunisation in infancy is assumed

For peer review only - http://bmjopen.bmj.com/site/about/guidelines.xhtml

Schedule of immunisation and sampling

The schedule of immunisation and sampling is outlined in **Figure 1** and **Table S1**. While optimal timings for outcome measures vary between vaccines, sampling at 8 weeks post BCG and 4 weeks post YF-17D, Ty21a, HPV and Td is proposed for the primary endpoints, targeting the establishment of memory responses and approximate peak of antibody responses. A secondary endpoint at one year will assess waning. All analyses will take baseline measurements into account. Immunisation postponement criteria are detailed in Supplementary information.

Outcomes

Primary outcomes

These will be assessed in all participants.

i. **BCG:** BCG-specific IFN-γ ELISpot response eight weeks post BCG immunisation.
ii. **YF-17D:** neutralising antibody titres (plaque-reduction neutralisation test) at four weeks post YF immunisation.
iii. **Ty21a:** *Salmonella typhi* lipopolysaccharide (LPS)-specific immunoglobulin (Ig)G concentration at four weeks post Ty21a immunisation.
iv. **HPV:** IgG specific for L1-proteins of HPV-16/18 at four weeks post HPV priming immunisation.
v. **Td:** Tetanus and diphtheria toxoid-specific IgG concentration at four weeks post Td immunisation.

Secondary outcomes

These will be assessed in all participants and will further investigate estimates of protective immunity (for vaccines where these are available) and dynamics of the vaccine responses, as well as the impact of the interventions on parasite clearance.

i. **Protective immunity.** Proportions with protective neutralising antibody (YF); protective IgG levels (TT);[31] seroconversion rates (Ty21a) at four weeks post the corresponding immunisation.
ii. **Response waning.** Primary outcome measures (all vaccines) repeated at week 52, and area-under-the curve (AUC) analyses. Parasitic infection may accelerate,[32] and anti-parasitic interventions delay, waning.
iii. **Priming versus boosting.** Effects on priming versus boosting will be examined for HPV only, comparing outcomes four weeks after the first, and four weeks after the second vaccine dose.

For peer review only - http://bmjopen.bmj.com/site/about/guidelines.xhtml

iv. **Current *S. mansoni* infection status and intensity** will be determined by serum/plasma levels of circulating anodic antigen (CAA). The method is quantitative, highly specific for *Schistosoma* infection, and much more sensitive than the conventional Kato Katz method.[33] CAA will be assessed retrospectively on stored samples collected at baseline, on immunisation days, and on primary and secondary endpoint days.

Furthermore, our sample collection will offer opportunities for an array of exploratory immunological evaluations on stored samples, focusing mainly on vaccine antigen specific outcomes. Exploratory assays will provide further detail on the role of immunological profiles and trans-kingdom effects in mediating helminth modulation of vaccine-specific responses.

Additional evaluation of parasite infection exposure

i. **Prior exposure to schistosomiasis** will be evaluated by ELISA for IgG to schistosome egg antigen using stored blood samples collected at baseline.
ii. **The presence of other helminth infections** will be determined retrospectively using stool PCR of samples collected at baseline and at weeks 28 and 52.[30] In accordance with national guidelines, all participants will be treated with albendazole or mebendazole after collection of samples for primary endpoints at week 8 and 28, and after collection of samples for secondary endpoints at week 52.
iii. **Current malaria infection status and intensity** will be assessed retrospectively by PCR on stored samples collected on immunisation days and at week 52. Individuals presenting with fever will be investigated using rapid diagnostic tests for malaria and treated based on the results and according to prevailing national guidelines.
iv. **Prior malaria exposure** will be evaluated by ELISA for IgG to malaria antigen using stored samples collected at baseline.

Sample size considerations

Based on the literature[4 34 35] and preliminary data, we anticipate that standard deviations (SDs) of primary outcome measures will lie between 0.3 and 0.6 $\log_{10}$, and that effective treatment may increase responses by approximately 0.2 $\log_{10}$ (based on Tweyongyere *et al.*[26]). We have therefore powered our study to detect differences of this magnitude (0.2 $\log_{10}$) or (in some cases) smaller. We assume *S. mansoni* prevalence of $\geq$80%.

Based on these assumptions, we plan to include 480 participants in total (240 quarterly PZQ, 240 annual PZQ); of whom 384 are expected to be *S. mansoni* infected, giving 192 participants in each trial arm who are infected at baseline.

For peer review only - http://bmjopen.bmj.com/site/about/guidelines.xhtml

Table 2 shows power estimates, for 5% significance level and assuming 20% loss to follow-up.

Table 2. Power estimates (5% significance level)

**Standard deviation ($\log_{10}$)**	**Log_{10} difference**						
	**0.08**	**0.10**	**0.12**	**0.14**	**0.16**	**0.18**	**0.20**
192 intensive PZQ vs 192 standard PZQ (*S. mansoni* infected only)							
0.3	65%	83%	94%	98%	>99%	>99%	>99%
0.4	42%	59%	75%	87%	94%	98%	99%
0.5	29%	42%	56%	69%	80%	88%	94%
0.6	21%	31%	42%	53%	65%	75%	83%
Cells highlighted in grey correspond to >80% power.							

Ethical and regulatory considerations

Ethical approval has been granted from the Research Ethics Committee of the Uganda Virus Research Institute (UVRI REC, reference: GC/127/19/05/664) and the London School of Hygiene and Tropical Medicine (LSHTM,reference: 16032), and from the Uganda National Council for Science and Technology (UNCST, reference: HS2486) and the Uganda National Drug Authority (NDA, reference: CTA0093). Any protocol amendments will be submitted to ethics committees and regulatory bodies for approval before implementation.

Participants are adolescents and therefore a vulnerable human population. Care will be taken to provide adequate, age and education-status appropriate information and to ensure that it is understood; and to emphasise that participation is voluntary. Participants will be enrolled only when they have given their own assent and when consent has been given by the parent or guardian. No major risks to the participants are anticipated since all the treatments and vaccines to be given are licensed and known to be safe. The main risk to participants will be time lost from school work: we will work with teachers and parents to minimise disruption to classes, and will avoid enrolment of primary 7 students since these classes are involved in national examinations. Further risks are discussed in Supplementary information.

Patient and public involvement

Concepts involved in this work have been discussed with colleagues at the Vector Control Division and Expanded Programme on Immunisation in the Ministry of Health (Uganda), with the Mukono District Council and with community leaders and Village Health Teams from Koome subcounty. We also have held meetings to explain the proposed work to teachers, parents, participants and village members, and to address their questions about issues such as study length, the study's ethical

For peer review only

286 approval status, if boys would also receive the HPV vaccine, and why adults were excluded from the study. Study findings will be shared with these stakeholders and with participants.

288 ***Dissemination***

289 Study findings will be published through open access peer-reviewed journals, presentations at local, national and international conferences and to the local community through community meetings. Anonymised participant level datasets generated will be available upon request.

292 ***Data management and analysis***

293 Socio-demographic information and clinical and laboratory measurements will be recorded and managed using Research Electronic Data Capture (REDCap) tools,[36 37] with paper-based forms as back-up. All data will be recorded under a unique study ID number. When paper forms must be used, data will be double entered in a study-specific database, with standard checks for discrepancies. All data for analysis will be anonymised and stored on a secure and password-protected server, with access limited to essential research personnel.

299 The effect of intensive (compared to standard) praziquantel treatment on the outcomes will be analysed. Information on infection status will only be available after randomisation. The primary analysis will be done on individuals identified as infected at baseline (through randomisation, these will be balanced between treatment arms); this will test the hypothesis that treating the infection (and subsequent reinfections) removes the parasite's effects. Secondary analyses will include all randomised individuals; this will provide insight into the potential benefit of the interventions as public health measures.

For peer review only

Discussion

This will be the first well-powered intervention study to investigate the effect of schistosomiasis treatment on vaccine responses in adolescents. This study will determine whether *S. mansoni* infection has a causal, reversible, impact on the response to live or inert vaccines, including effects on vaccine replication, immune response profile, priming, boosting and waning. The results will add to understanding of population differences in vaccine responses and on interventions that may enhance responses. If treating helminths improves vaccine responses in adolescents, combined parasite-control/immunisation programmes offer an attractive, practical public health intervention for schools and communities.

There are risks associated with our approach to addressing the trial objective. **First**, there is a risk of failure to clear *S. mansoni* infections, and repeated reinfection during the trial. This issue can be challenging because of incomplete cure or maturation of immature worms after treatment, and lifestyles in endemic communities that result in repeated exposure. To mitigate this, we will administer three PZQ treatments over a six-week period before the first immunisations, and continuing quarterly treatment in the intensive arm. **Second,** there is a risk that *S. mansoni* infection has long-term effects, not removed by treatment, mediated, for example, by epigenetic change.[38] However, studies show that parasite treatment results in immunological changes,[39,40] and our data suggest at least partial recovery of the measles vaccine response among young children treated for schistosomiasis.[26] By initiating intervention six to eight weeks before the first immunisations, and providing repeated intervention in the intensive arms, we hope to achieve significant resolution of *S. mansoni* effects.

We are interested in the effects of removing *S. mansoni*. Treating parasites can induce acute immunological change due to release of previously hidden antigens. To minimise such effects, immunisations will be given at least 2 weeks after PZQ (the longest practicable interval; **Figure 1**).

Laboratory analyses will also highlight immune parameters and cellular populations that link environmental exposures to vaccine responses. Identifying processes associated with poor or good outcomes will inform strategies in vaccine design (for example, the genetic modification of vaccines, or innovative use of adjuvants, to counter any adverse immunological milieu, currently an area of intense research for cancer vaccines[41]); ultimately supporting the development of effective vaccines tailored to the low-income settings that most need them.

Study timeline

For peer review only - http://bmjopen.bmj.com/site/about/guidelines.xhtml

Applications for ethical approval were submitted in May 2018, with approval received in September 2018 (Uganda Virus Research Institute Research Ethics Committee), May 2019 (National Drug Authority and Uganda National Council for Science and Technology), June 2019 (London School of Hygiene and Tropical Medicine). Collaborator/investigator/trial steering committee meetings were also held during the initial 12-month planning period. The study began recruitment in July 2019. Intervention will be up to 12 months, with completion of the project scheduled for September 2020.

Competing interests

Alison Elliott reports a grant from the Medical research Council, UK (POPVAC programme funding). The rest of the authors declare that they have no conflicts of interest.

Author contributions

AME conceived the study. AME, GN, ELW, AN, JN, SC, LZ and JK contributed to study design. LZ, GO, PNK, EN, GK, RA, CN, CO, MN, SA and FA are site clinicians/nurses/clinical laboratory technicians providing valuable input on clinical considerations of the intervention. MS, SK and RK are field workers handling the organisational integration of the intervention. AN, AM and ELW are involved in organisation of the databases, trial randomization, treatment allocation and drawing up of analytical plans. GN, LZ, JN, AN, SC, ELW and AME drafted the manuscript. All authors reviewed the manuscript, contributed to it and approved the final version.

Acknowledgements

We thank the Uganda National Expanded Programme for Immunisation, Sanofi Pasteur and PaxVax for providing the HPV, yellow fever and oral typhoid vaccines, respectively. The BCG and tetanus-diphtheria vaccines were kind donations from the Serum Institute of India. We thank the Vector Control Division of the Ministry of Health and the Mukono district local government for their support. We also thank members of the POPVAC programme steering committee (chaired by Prof. Richard Hayes) and the Data and Safety Monitoring Board (Dr David Meya, Prof Andrew Prendergast and Dr Elizabeth George).

Funding

The POPVAC programme of work is supported by the Medical Research Council of the United Kingdom (grant number MR/R02118X/1). SC and JN are supported in part by the Makerere University – Uganda Virus Research Institute Centre of Excellence for Infection and Immunity Research and Training (MUII-plus). MUII-plus is funded under the DELTAS Africa Initiative. The DELTAS Africa Initiative is an independent funding scheme of the African Academy of Sciences (AAS),

For peer review only - http://bmjopen.bmj.com/site/about/guidelines.xhtml

Alliance for Accelerating Excellence in Science in Africa (AESA) and supported by the New Partnership for Africa's Development Planning and Coordinating Agency (NEPAD Agency) with funding from the Wellcome Trust (grant 107743) and the UK Government. The MRC/UVRI and LSHTM Uganda Research Unit is jointly funded by the UK Medical Research Council (MRC) and the UK Department for International Development (DFID) under the MRC/DFID Concordat agreement and is also part of the EDCTP2 programme supported by the European Union.

The study sponsor (London School of Hygiene and Tropical Medicine) and funders had no role in study design; collection, management, analysis, and interpretation of data; writing of the protocol; and the decision to submit the protocol for publication.

POPVAC trial team

Principal investigator: Alison Elliott; ***Project leader***: Ludoviko Zirimenya; ***laboratory staff:*** Gyaviira Nkurunungi, Stephen Cose, Rebecca Amongin, Beatrice Nassanga, Jacent Nassuuna, Irene Nambuya, Prossy Kabuubi, Emmanuel Niwagaba, Gloria Oduru, Grace Kabami; ***statisticians and data managers:*** Emily Webb, Agnes Natukunda, Helen Akurut, Alex Mutebe; ***clinicians:*** Anne Wajja, Milly Namutebi, Christopher Zziwa, Joel Serubanja; ***nurses:*** Caroline Onen, Esther Nakazibwe, Josephine Tumusiime, Caroline Ninsiima, Susan Amongi, Florence Akello; ***internal monitor:*** Mirriam Akello; ***field workers:*** Robert Kizindo, Moses Sewankambo, Denis Nsubuga, Samuel Kiwanuka, Fred Kiwudhu; ***boatman:*** David Abiriga; ***administrative management:*** Moses Kizza, Samsi Nansukusa; ***internal and external collaborators:*** Pontiano Kaleebu, Hermelijn Smits, Maria Yazdanbakhsh, Govert van Dam, Paul Corstjens, Sarah Staedke, Henry Luzze, James Kaweesa, Edridah Tukahebwa, Elly Tumushabe, Moses Muwanga

For peer review only - http://bmjopen.bmj.com/site/about/guidelines.xhtml

References

1. Taniuchi M, Platts-Mills JA, Begum S, et al. Impact of enterovirus and other enteric pathogens on oral polio and rotavirus vaccine performance in Bangladeshi infants. *Vaccine* 2016;34(27):3068-75. doi: 10.1016/j.vaccine.2016.04.080 [published Online First: 2016/05/08]

2. Fine PE. Variation in protection by BCG: implications of and for heterologous immunity. *Lancet* 1995;346(8986):1339-45.

3. Black GF, Weir RE, Floyd S, et al. BCG-induced increase in interferon-gamma response to mycobacterial antigens and efficacy of BCG vaccination in Malawi and the UK: two randomised controlled studies. *Lancet* 2002;359(9315):1393-401. doi: 10.1016/S0140-6736(02)08353-8

4. Muyanja E, Ssemaganda A, Ngauv P, et al. Immune activation alters cellular and humoral responses to yellow fever 17D vaccine. *The Journal of clinical investigation* 2014;124(7):3147-58. doi: 10.1172/jci75429 [published Online First: 2014/06/10]

5. Barreto ML, Pilger D, Pereira SM, et al. Causes of variation in BCG vaccine efficacy: examining evidence from the BCG REVAC cluster randomized trial to explore the masking and the blocking hypotheses. *Vaccine* 2014;32(30):3759-64. doi: 10.1016/j.vaccine.2014.05.042

6. Tanner R, Kakalacheva K, Miller E, et al. Serum indoleamine 2,3-dioxygenase activity is associated with reduced immunogenicity following vaccination with MVA85A. *BMC infectious diseases* 2014;14:660. doi: 10.1186/s12879-014-0660-7 [published Online First: 2014/12/04]

7. Sissoko MS, Healy SA, Katile A, et al. Safety and efficacy of PfSPZ Vaccine against Plasmodium falciparum via direct venous inoculation in healthy malaria-exposed adults in Mali: a randomised, double-blind phase 1 trial. *The Lancet Infectious diseases* 2017;17(5):498-509. doi: 10.1016/s1473-3099(17)30104-4 [published Online First: 2017/02/22]

8. Venkatraman N, Ndiaye BP, Bowyer G, et al. Safety and Immunogenicity of a Heterologous Prime-Boost Ebola Virus Vaccine Regimen in Healthy Adults in the United Kingdom and Senegal. *The Journal of infectious diseases* 2018;219(8):1187-97. doi: 10.1093/infdis/jiy639

9. Barreto ML, Pereira SM, Pilger D, et al. Evidence of an effect of BCG revaccination on incidence of tuberculosis in school-aged children in Brazil: second report of the BCG-REVAC cluster-randomised trial. *Vaccine* 2011;29(31):4875-7. doi: 10.1016/j.vaccine.2011.05.023

10. van Riet E, Adegnika AA, Retra K, et al. Cellular and humoral responses to influenza in gabonese children living in rural and semi-urban areas. *The Journal of infectious diseases* 2007;196(11):1671-8. doi: 10.1086/522010 [published Online First: 2007/11/17]

11. van Riet E, Retra K, Adegnika AA, et al. Cellular and humoral responses to tetanus vaccination in Gabonese children. *Vaccine* 2008;26(29-30):3690-5. doi: 10.1016/j.vaccine.2008.04.067 [published Online First: 2008/06/10]

12. Venkatraman N, Ndiaye BP, Bowyer G, et al. Safety and Immunogenicity of a Heterologous Prime-Boost Ebola Virus Vaccine Regimen in Healthy Adults in the United Kingdom and Senegal. *The Journal of infectious diseases* 2019;219(8):1187-97. doi: 10.1093/infdis/jiy639

13. Brandt L, Feino Cunha J, Weinreich Olsen A, et al. Failure of the Mycobacterium bovis BCG vaccine: some species of environmental mycobacteria block multiplication of BCG and induction of protective immunity to tuberculosis. *Infection and immunity* 2002;70(2):672-8. [published Online First: 2002/01/18]

14. Flaherty DK, Vesosky B, Beamer GL, et al. Exposure to Mycobacterium avium can modulate established immunity against Mycobacterium tuberculosis infection generated by Mycobacterium bovis BCG vaccination.

For peer review only

For peer review only - http://bmjopen.bmj.com/site/about/guidelines.xhtml

Journal of leukocyte biology 2006;80(6):1262-71. doi: 10.1189/jlb.0606407 [published Online First: 2006/09/14]

15. Maizels RM, McSorley HJ. Regulation of the host immune system by helminth parasites. *The Journal of allergy and clinical immunology* 2016;138(3):666-75. doi: 10.1016/j.jaci.2016.07.007 [published Online First: 2016/08/02]

16. Wammes LJ, Mpairwe H, Elliott AM, et al. Helminth therapy or elimination: epidemiological, immunological, and clinical considerations. *The Lancet Infectious diseases* 2014;14(11):1150-62. doi: 10.1016/S1473-3099(14)70771-6

17. Wammes LJ, Hamid F, Wiria AE, et al. Regulatory T cells in human geohelminth infection suppress immune responses to BCG and Plasmodium falciparum. *Eur J Immunol* 2010;40(2):437-42. doi: 10.1002/eji.200939699

18. Pfeiffer JK, Virgin HW. Viral immunity. Transkingdom control of viral infection and immunity in the mammalian intestine. *Science* 2016;351(6270) doi: 10.1126/science.aad5872

19. Onguru D, Liang Y, Griffith Q, et al. Human schistosomiasis is associated with endotoxemia and Toll-like receptor 2- and 4-bearing B cells. *The American journal of tropical medicine and hygiene* 2011;84(2):321-4. doi: 10.4269/ajtmh.2011.10-0397 [published Online First: 2011/02/05]

20. George PJ, Anuradha R, Kumar NP, et al. Evidence of microbial translocation associated with perturbations in T cell and antigen-presenting cell homeostasis in hookworm infections. *PLoS neglected tropical diseases* 2012;6(10):e1830. doi: 10.1371/journal.pntd.0001830 [published Online First: 2012/10/12]

21. Rajamanickam A, Munisankar S, Bhootra Y, et al. Microbial Translocation Associated with an Acute-Phase Response and Elevations in MMP-1, HO-1, and Proinflammatory Cytokines in Strongyloides stercoralis Infection. *Infection and immunity* 2017;85(1) doi: 10.1128/iai.00772-16 [published Online First: 2016/11/09]

22. Sanya RE, Nkurunungi G, Andia Biraro I, et al. A life without worms. *Transactions of the Royal Society of Tropical Medicine and Hygiene* 2017:1-9. doi: 10.1093/trstmh/trx010 [published Online First: 2017/03/25]

23. Elias D, Britton S, Aseffa A, et al. Poor immunogenicity of BCG in helminth infected population is associated with increased in vitro TGF-beta production. *Vaccine* 2008;26(31):3897-902. doi: S0264-410X(08)00540-9 [pii]
10.1016/j.vaccine.2008.04.083 [published Online First: 2008/06/17]

24. Elias D, Wolday D, Akuffo H, et al. Effect of deworming on human T cell responses to mycobacterial antigens in helminth-exposed individuals before and after bacille Calmette-Guerin (BCG) vaccination. *Clin Exp Immunol* 2001;123(2):219-25.

25. Cooper PJ, Chico ME, Losonsky G, et al. Albendazole treatment of children with ascariasis enhances the vibriocidal antibody response to the live attenuated oral cholera vaccine CVD 103-HgR. *The Journal of infectious diseases* 2000;182(4):1199-206. doi: 10.1086/315837 [published Online First: 2000/09/09]

26. Tweyongyere R, Nassanga BR, Muhwezi A, et al. Effect of Schistosoma mansoni infection and its treatment on antibody responses to measles catch-up immunisation in pre-school children: A randomised trial. *PLoS neglected tropical diseases* 2019;13(2):e0007157. doi: 10.1371/journal.pntd.0007157 [published Online First: 2019/02/15]

27. Chan AW, Tetzlaff JM, Altman DG, et al. SPIRIT 2013 statement: defining standard protocol items for clinical trials. *Ann Intern Med* 2013;158(3):200-7. doi: 10.7326/0003-4819-158-3-201302050-00583 [published Online First: 2013/01/09]

28. Sanya RE, Nkurunungi G, Hoek Spaans R, et al. The Impact of Intensive Versus Standard Anthelminthic Treatment on Allergy-related Outcomes, Helminth Infection Intensity, and Helminth-related Morbidity in

For peer review only

For peer review only - http://bmjopen.bmj.com/site/about/guidelines.xhtml

Lake Victoria Fishing Communities, Uganda: Results From the LaVIISWA Cluster-randomized Trial. *Clinical Infectious Diseases* 2018:ciy761-ciy61. doi: 10.1093/cid/ciy761

29. WHO. Adolescent Health Research Priorities: Report of a Technical Consultation 2015. http://apps.who.int/iris/bitstream/10665/203564/1/WHO_FWC_MCA_15_07_eng.pdf?ua=1 (accessed 17th May 2019).

30. Webb EL, Nampijja M, Kaweesa J, et al. Helminths are positively associated with atopy and wheeze in Ugandan fishing communities: results from a cross-sectional survey. *Allergy* 2016 doi: 10.1111/all.12867

31. Plotkin SA. Correlates of protection induced by vaccination. *Clinical and vaccine immunology : CVI* 2010;17(7):1055-65. doi: 10.1128/cvi.00131-10 [published Online First: 2010/05/14]

32. Riner DK, Ndombi EM, Carter JM, et al. Schistosoma mansoni Infection Can Jeopardize the Duration of Protective Levels of Antibody Responses to Immunizations against Hepatitis B and Tetanus Toxoid. *PLoS neglected tropical diseases* 2016;10(12):e0005180. doi: 10.1371/journal.pntd.0005180

33. Corstjens PL, Nyakundi RK, de Dood CJ, et al. Improved sensitivity of the urine CAA lateral-flow assay for diagnosing active Schistosoma infections by using larger sample volumes. *Parasites & vectors* 2015;8:241. doi: 10.1186/s13071-015-0857-7 [published Online First: 2015/04/22]

34. Fletcher HA, Snowden MA, Landry B, et al. T-cell activation is an immune correlate of risk in BCG vaccinated infants. *Nat Commun* 2016;7:11290. doi: 10.1038/ncomms11290

35. Safaeian M, Porras C, Pan Y, et al. Durable antibody responses following one dose of the bivalent human papillomavirus L1 virus-like particle vaccine in the Costa Rica Vaccine Trial. *Cancer prevention research (Philadelphia, Pa)* 2013;6(11):1242-50. doi: 10.1158/1940-6207.capr-13-0203 [published Online First: 2013/11/06]

36. Harris PA, Taylor R, Minor BL, et al. The REDCap Consortium: Building an International Community of Software Platform Partners. *Journal of biomedical informatics* 2019:103208. doi: 10.1016/j.jbi.2019.103208 [published Online First: 2019/05/13]

37. Harris PA, Taylor R, Thielke R, et al. Research electronic data capture (REDCap)--a metadata-driven methodology and workflow process for providing translational research informatics support. *Journal of biomedical informatics* 2009;42(2):377-81. doi: 10.1016/j.jbi.2008.08.010 [published Online First: 2008/10/22]

38. Blok BA, Arts RJ, van Crevel R, et al. Trained innate immunity as underlying mechanism for the long-term, nonspecific effects of vaccines. *J Leukoc Biol* 2015;98(3):347-56. doi: 10.1189/jlb.5RI0315-096R

39. Watanabe K, Mwinzi PN, Black CL, et al. T regulatory cell levels decrease in people infected with Schistosoma mansoni on effective treatment. *The American journal of tropical medicine and hygiene* 2007;77(4):676-82. [published Online First: 2007/11/06]

40. Schmiedel Y, Mombo-Ngoma G, Labuda LA, et al. CD4+CD25hiFOXP3+ Regulatory T Cells and Cytokine Responses in Human Schistosomiasis before and after Treatment with Praziquantel. *PLoS neglected tropical diseases* 2015;9(8):e0003995. doi: 10.1371/journal.pntd.0003995 [published Online First: 2015/08/21]

41. Seledtsov VI, Goncharov AG, Seledtsova GV. Clinically feasible approaches to potentiating cancer cell-based immunotherapies. *Human vaccines & immunotherapeutics* 2015;11(4):851-69. doi: 10.1080/21645515.2015.1009814 [published Online First: 2015/05/02]

For peer review only - http://bmjopen.bmj.com/site/about/guidelines.xhtml

FIGURE LEGENDS

Figure 1. Outline of immunisations and anthelminthic intervention

[1]Primary endpoints will be at eight weeks post BCG and four weeks post Yellow fever (YF-17D), oral typhoid (Ty21a), Human Papilloma Virus (HPV) and Tetanus/diptheria (Td) vaccination.

[2]Primary endpoint for responses to Td given at 28 weeks.

For peer review only

For peer review only - http://bmjopen.bmj.com/site/about/guidelines.xhtml

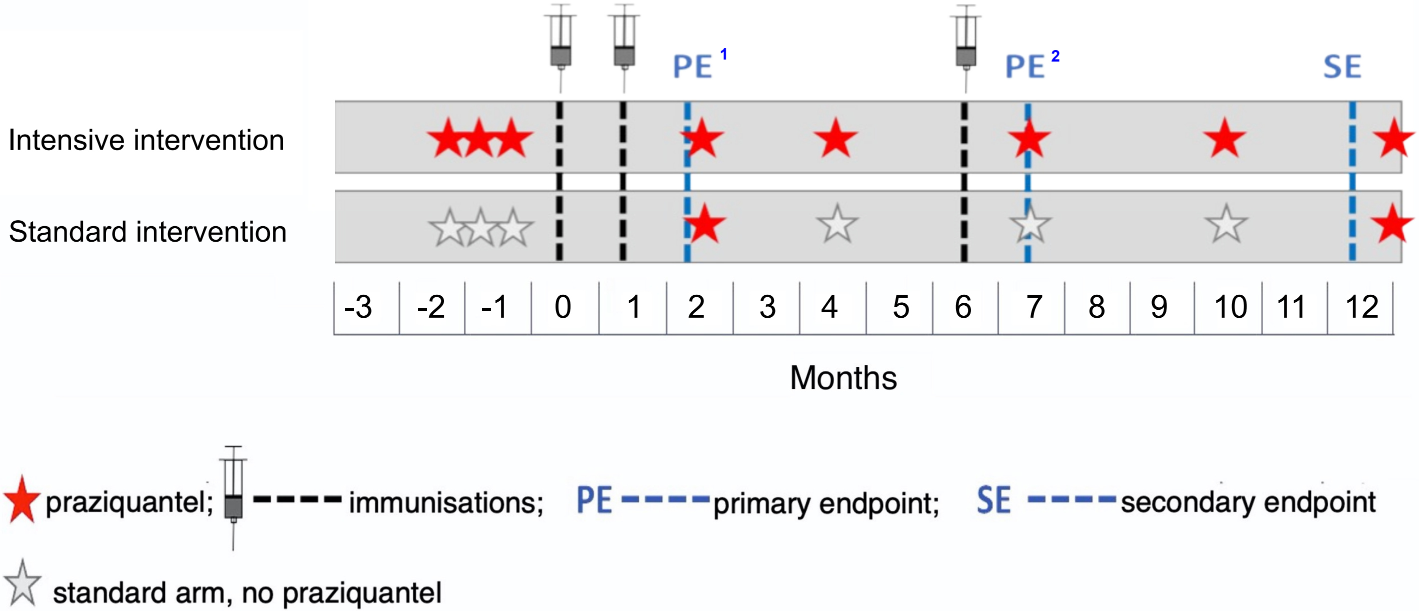
PE 1
PE 2
SE
Intensive intervention
Standard intervention
-3
-2
-1
0
1
2
3
4
5
6
7
8
9
10
11
12
Months
praziquantel;
immunisations;
PE primary endpoint;
SE secondary endpoint
standard arm, no praziquantel

SUPPLEMENTARY INFORMATION

The effect of intensive treatment for schistosomiasis on immune responses to vaccines among rural Ugandan island adolescents: randomised controlled trial protocol A for the 'POPulation differences in VACcine responses' (POPVAC) programme

Gyaviira Nkurunungi[1,¶,*], Ludoviko Zirimenya[1,¶], Jacent Nassuuna[1,¶], Agnes Natukunda[1,¶], Prossy N Kabuubi[1], Emmanuel Niwagaba[1], Gloria Oduru[1], Grace Kabami[1], Rebecca Amongin[1], Alex Mutebe [1], Milly Namutebi[1], Christopher Zziwa[1], Susan Amongi[1], Caroline Ninsiima[1], Caroline Onen[1], Florence Akello[1], Moses Sewankambo[1], Samuel Kiwanuka[1], Robert Kizindo[1], James Kaweesa[2], Stephen Cose[1,3], Emily L Webb[4], Alison M Elliott[1,3] **for the POPVAC trial team**

[1]Immunomodulation and Vaccines Programme, Medical Research Council/Uganda Virus Research Institute and London School of Hygiene and Tropical Medicine (MRC/UVRI and LSHTM) Uganda Research Unit, Entebbe, Uganda

[2]Vector Control Division, Ministry of Health, Kampala, Uganda

[3]Department of Clinical Research, London School of Hygiene and Tropical Medicine, London, United Kingdom

[4]MRC Tropical Epidemiology Group, Department of Infectious Disease Epidemiology, London School of Hygiene and Tropical Medicine, London, United Kingdom

¶These authors contributed equally

***Correspondence:** Gyaviira Nkurunungi; gyaviira.nkurunungi@lshtm.ac.uk

For peer review only

Table S1. Schedule of visits and procedures

VISIT NUMBER	1	2 & 3	4	5[9]	5.2	6	7	8	9	10	11
WEEKS FROM 1ST IMMUNISATION	-8[1]	-6[10], -4, -2	0	4	4 weeks +4 days	8	20	28	32	44	52
	Screening	Treatment (Rx) only	Immunisations	Immunisations		Primary Endpoint (PE), Immunisations	Rx only	Immunisations	PE/Secondary Endpoint (SE)[2]	Rx only	SE, Immunisations
**RANDOMISED INTENSIVE VS STANDARD PRAZIQUANTEL INTERVENTION**											
PZQ intensive arm (x)		x				x[3]	x		x[3]	x	x[3]
PZQ standard arm						x[3]					x[3]
Albendazole						x[3]			x[3]		x[3]
**VACCINES**											
BCG			x								
YF-17D				x							
Ty21a				x							
HPV				x		[x][4]		x			
Td								x			[x][5]
**INVESTIGATIONS/PROCEDURES**											
Inclusion/exclusion criteria	x										
Informed consent	x										
Questionnaire	x		x	x	x	x		x	x		x
Examination	x		(x)	(x)	(x)	(x)		(x)	(x)		(x)
Urine β-HCG test (female only) 1mL	x		x	x				x			
Urine YF viral load					x						
Stool for PCR and storage	x							x			x
Stool for coproantibody and storage	x					x					
**BLOOD SAMPLES**											
Malaria PCR (1ml)			x	x				x			x
Serology for HIV, prior malaria and *S. mansoni* (0.5ml)	x										
Mansonella perstans (1ml)	x										
Serum/plasma CAA (1ml)	x		x	x		x		x	x		x
Hb[8] / Full blood count (0.5ml)	x		x	x				x			
Assessments of pre-immunisation responses, and/or vaccine response outcomes and/or exploratory immunology; storage[7] (10 – 20 mls)			x	x		x		x	x		x
Blood for gene expression (2mls)			x	x				x			
Blood vol (mL)	4		27	17		10-20		27	10		14
Cumulative blood vol (mL)[7]	4		31	48		68		95	105		119

PE: primary endpoint; **SE:** secondary endpoint; **Rx only**: treatment only
Immunisation days highlighted in green, primary end point days in red, days for treatment only in grey
(**x**) performed if clinically indicated

1. Screening and enrolment into Project A will take place about 8 weeks before immunisation 0 to allow initiation of the praziquantel intervention.
2. Week 32 is primary endpoint for responses to Td given at 28 weeks; secondary endpoint for HPV booster
3. Treatments given after sampling when schedules coincide
4. Week 8 HPV dose will be given for previously-unvaccinated girls aged $\geq$14 years
5. Week 52 Td booster dose will be provided as a service
6. Exploratory immunology blood volume will be guided by guidelines from Harvard Mass General, where a maximum of 3ml/kg body weight is taken at any one time point and not more than 3ml/kg is taken over any 8-week period (ref http://www.drgreene.com/21_1616.html.) These guidelines have been followed in a previous study vaccinating adolescents with investigational tuberculosis vaccine MVA85A (in Uganda).[1] The total blood volume planned is 68 ml over the initial intensive sampling period of 8 weeks. Revision of sample volumes based on weight will only be required for participants who weigh less than 22 kg; the average weight of children aged 9 years is expected to be 28kg (with 21kg the 3rd centile) with greater weights for older children.[2]
7. At baseline, it will only be Hb estimation by Haemocue
8. Oral typhoid vaccine doses will be administered on three alternate days namely visit 5, 5.1 and 5.2.
9. The first PZQ treatment at week -6 will be administered at the end of the screening visit

For peer review only

For peer review only - http://bmjopen.bmj.com/site/about/guidelines.xhtml

Further information on recruitment criteria

- Participants who are excluded from the trial because they have been discovered (during screening procedures) to be suffering from a previously undiagnosed condition thought to require further medical attention will be referred appropriately for further investigation and treatment.
- Participants discovered to have severe anaemia will be excluded from the trial and treated for anaemia
- Participants discovered to be HIV-positive will be counselled and offered a CD4 T-cell count and referred to a provider of antiretroviral treatment ("Test and Treat" – i.e. initiation of treatment regardless of CD4 count is recommended for these high-risk communities).
- Participants discovered to be pregnant will be counselled and referred to an antenatal clinic of their choice.

This trial proposes to recruit all participants within a short time-frame. It will not, therefore, be possible to reconsider enrolment of potential participants with temporary exclusion criteria after treatment and resolution of the condition.

Further rationale for the selection of vaccines

Bacillus Calmette–Guérin (BCG)

BCG is a live, replicating parenteral vaccine, and the only licensed vaccine against TB. The BCG vaccine for these studies will be obtained from the Serum Institute of India either directly, or through a supplier in Uganda. The Serum Institute of India provides much of the BCG vaccine used in Uganda.

Worldwide, TB is among the top 10 causes of death; Uganda has an estimated incidence of 202/100,000 people.[3] Infectious, sputum positive, pulmonary TB classically emerges in adolescence, driving the on-going epidemic.[4] Thus adolescent booster immunisation is a key TB control strategy.[5] However, BCG vaccine response and efficacy are often impaired in tropical and rural settings[6-8] and new TB vaccines are similarly affected.[9] In the past, the WHO has been hesitant to recommend BCG re-vaccination. However, in 2017 WHO's Strategic Advisory Group of Experts (SAGE) recommended: "Further research is warranted to explore whether certain sub-groups of age, geographic or *M. tuberculosis* exposure categories would benefit from re-vaccination."[10] Recent results suggest that, despite the variability of BCG efficacy between populations, BCG vaccination in adolescence offers benefit in some tropical settings, especially for individuals who are not yet infected with *Mycobacterium tuberculosis*, and may also be cost-effective.[7 11] Also, BCG vaccine is currently being used among adolescents in South Africa as a comparator in a trial of a novel TB vaccine (trial

For peer review only - http://bmjopen.bmj.com/site/about/guidelines.xhtml

registration NCT02075203). BCG efficacy in Ugandan adolescents, and differences in BCG vaccine responses between urban and rural Ugandan populations, have not been tested. Information obtained from this study is expected to further inform the use of BCG in adolescents, and also to inform the development of new vaccines for tuberculosis.

Yellow fever vaccine

Yellow fever vaccine YF-17D is a live replicating parenteral vaccine. The vaccine (Stamaril; Sanofi Pasteur) is available for purchase in Uganda. Yellow Fever (YF) causes outbreaks in Uganda and the wider region[12] and YF-17D is a candidate for Uganda's expanded programme on immunisation (EPI). Lower vaccine replication, lower neutralising antibody induction, and greater waning, are described in Uganda compared to Switzerland.[13] YF-17D is a potential vector for novel vaccine constructs,[14] adding relevance to vaccine development.

Typhoid vaccine Ty21a

Typhoid vaccine Ty21a is a live replicating oral vaccine and also a potential vector for new vaccine constructs.[15] The Ty21a vaccine will be purchased from PaxVax, Redwood City, California. Substantial, multi-year typhoid outbreaks occur in Uganda and immunisation campaigns have been advocated as cost effective.[16] Schistosomiasis has been associated with prolonged *S. typhi* infection[17] and impaired antibody responses to killed typhoid vaccines.[18]

Ty21a was developed in the 1970s. Although not routinely used in Uganda, it has been (and is currently) registered in many countries. It was first registered in the United States and United Kingdom in the 1980s, and is recommended by the WHO for both endemic and epidemic settings.[19] It has comparable efficacy to the parenteral Vi polysaccharide typhoid vaccine, good durability and minimal adverse effects.[19] It is proposed for use in this study to model effects of study exposures and intervention on the response to a live oral vaccine.

The Ty21a vaccine is given as a three-dose regimen on alternate days.

Human Papilloma Virus (HPV) vaccine

The Human Papilloma Virus (HPV) vaccine is a protein virus-like particle. The quadrivalent HPV Vaccine Gardasil (Merck) is available for purchase in Uganda and is the vaccine used by the national EPI programme. Studies after three vaccine doses have found somewhat enhanced responses in the presence of malaria, but no effect of helminths.[20] No study has previously investigated parasite effects on the priming response, but recent results for tetanus suggest that priming may be more susceptible than boosting to adverse effects.[21] This will be important if forthcoming trials support single-dose HPV immunisation (NCT02834637). HPV immunisation is being rolled out among girls to

For peer review only

prevent cervical neoplasia, the most common cancer among Ugandan women and we will coordinate provision with the national HPV immunisation programme.[22] HPV immunisation is also beneficial for boys since HPV infection is associated with anogenital warts, anal cancer and oropharyngeal cancers in both males and females, and with penile cancer in men,[23] and we will include boys in these studies.

Tetanus and diphtheria vaccines

Tetanus and diphtheria (Td) vaccines comprise inert toxoids. Schistosomiasis is associated with a Th2 biased response to tetanus toxoid[24] and with suppressed antibody responses among those with low pre-immunisation antibody levels.[21] Booster immunisation is recommended for young women to prevent maternal and neonatal tetanus. Recent evidence emphasises the need to protect young men also.[25]

Immunisation Postponement Criteria

If any one of the following is identified at the time scheduled for immunisation, the participant may be immunised at a later date, or withdrawn, at the discretion of the Investigator. The participant must be followed until resolution of the event as with any adverse event:

- Acute disease at the time of immunisation. Acute disease is defined as the presence of a moderate or severe illness with or without fever. All vaccines can be administered to persons with a minor illness such as diarrhoea or mild upper respiratory infection with or without low-grade fever, i.e. temperature of ≤37.5°C (99.5°F)
- Temperature of >37.5°C (99.5°F) at the time of immunisation
- Taking antibiotics or antimalarials currently, or within the past 7 days, of the date of Ty21a administration (ascertained verbally)

Vaccine storage and transport

In order to maintain a reliable vaccine cold chain, the vaccines and diluents to be used will be stored and transported within the recommended temperature range of +2°C to +8°C. Care will be taken to ensure that the vaccines are not frozen. BCG, being sensitive to light, will be kept in the dark (normally within its secondary packaging) for as long as possible to protect it during storage and transportation. All vaccines will be kept in appropriate refrigeration equipment with a temperature monitoring device to ensure temperatures remain between +2°C and +8°C. Cold boxes/vaccines carriers with temperature monitors will be used to transport vaccines and the diluents from the MRC/UVRI and LSHTM Uganda Research Unit (Entebbe) to Koome island and while transporting vaccines to immunisation sessions. Designated staff will be given responsibility for managing the

For peer review only

vaccine cold chain. All cold chain equipment including the temperature monitoring devices used for this project will comply with relevant technical specifications as defined by the EPI standards. Basic routine maintenance will be regularly carried out on all cold chain equipment.

Additional laboratory measurements

Additional assays will comprise HIV serology, pregnancy testing and full blood counts. HIV testing and pregnancy testing will be accompanied by appropriate counselling by trained staff.

- HIV serology will be done on blood samples using rapid tests and according to prevailing national algorithms.[26] This will be done at baseline.
- Pregnancy testing will be done using urine samples and standard operating procedures for assessment of urine β-human chorionic gonadotropin (βhCG). This will be done at baseline and before immunisation on each immunisation day.
- Full blood counts will be conducted using a haematology analyser. Mild, moderate and severe anaemia will be defined according to WHO guidelines, by age.[27] This will be done at baseline to test for anaemia as part of the eligibility assessment, and pre-immunisation as part of the assessment of immunological profile.

Individuals found to be HIV positive or pregnant will be referred to appropriate providers for further care. Individuals with severe anaemia (haemoglobin <82g/L) will be excluded from the randomised intervention (since the intervention might be beneficial in management of anaemia). They will be treated for anaemia.

Sample handling and archive

Blood and other samples will be processed according to local laboratory standard operating procedures (SOPs). All samples will reach the laboratory in anonymised form.

A sample archive will be developed. Although our current programme of work will address specific hypotheses regarding pathways of effects of parasites and interventions, the sample archive will provide a major asset for exploration of new leads arising from this work, or for an alternative, "systems biology" approach employing (for example) proteomic, genomic, epigenetic and transcriptomic analyses, and investigating the microbiome and virome. Information provided to participants, and consent forms, will include considerations of sample storage, and the possibility of sample analysis in laboratories within and outside Uganda. Participants will be able to decide if they will permit such future use of any leftover samples. We plan to store the samples for up to 20 years. If further storage is needed after that time, permission will be requested from the Uganda Virus Research Institute and London School of Hygiene and Tropical Medicine ethical review committees.

For peer review only - http://bmjopen.bmj.com/site/about/guidelines.xhtml

If they elect not to permit this, all of those leftover samples will be discarded after the completion of the work included in the current protocol.

Operational considerations

Programme governance

A Programme Steering Committee has been set up to guide progress across all projects. This comprises the following:

- An independent chair
- Representatives from the Ministry of Health programmes for immunisation and for vector borne disease control
- Representatives of district authorities (Mukono and Jinja districts)
- Community representatives
- Principal investigator and co-investigators
- Project leader and post-doctoral immunologist
- Trial statistician
- Laboratory manager
- Medical Research Council observer

Informed consent

Both written informed assent from the participants and written informed consent from a parent or guardian will be required for participation, although these may not necessarily be obtained at the same time. Information will be provided in both English and the appropriate local language. For individuals who cannot speak the languages used, or who cannot read or write, a witness who can read the information sheet and translate the information to the participant or parent/guardian will be used. Two different types of age specific assent forms will be used for the group of participants aged 9 – 12 years and for the group aged 13 – 17 years. Informed consent by emancipated or mature minors will be obtained using a designated consent form for these categories of participants.

The aims of the study, all tests, treatments and immunisations to be carried out and potential risks will be explained. The participant will be given the opportunity to ask about details of the trial, and will then have time to consider whether or not to participate. If they do decide to participate, they and their parent/guardian will sign and date two copies of the assent and consent forms, one for them to take away and keep, and one to be stored securely by the research team. Separate information and consent forms will be provided (i) for consent for storage of samples for future

For peer review only - http://bmjopen.bmj.com/site/about/guidelines.xhtml

studies and for anonymous sharing of data from this study and (ii) for possible genetic studies; the information sheet will explain that these data may be used in analyses related to this protocol.

Screening and Eligibility Assessment

Once the informed consent process has been completed, and consent (and assent) given, a baseline medical history (including concomitant medication) will be collected. Vital signs will be checked and a physical examination will be performed. Inclusion and exclusion criteria will be checked.

Participants will undergo pre- and post-test counselling for HIV and (for girls) pregnancy testing by a trained and experienced nurse- or clinician-counsellor. Blood, urine and stool samples will be obtained, for tests as specified in the schedule of procedures. These tests are to exclude the major, immunomodulating co-infection, HIV, and conditions that might impact safety (anaemia, pregnancy).

Enrolment

Participants who consent/assent, complete the screening processes, satisfy all the inclusion criteria and meet none of the exclusion criteria will be enrolled.

Discontinuation/withdrawal criteria

In accordance with the principles of the current revision of the Declaration of Helsinki and any other applicable regulations, a participant has the right to withdraw from the study at any time and for any reason, and is not obliged to give his or her reasons for doing so. The Investigator may withdraw the participant at any time in the interests of the participant's health and well-being. In addition, the participant may withdraw/be withdrawn for any of the following reasons:

- Ineligibility (either arising during the study or retrospectively, having been overlooked at screening)
- Administrative decision by the Investigator
- Significant protocol deviation
- Participant non-compliance with study requirements
- An adverse event which requires discontinuation of the study involvement or results in inability to continue to comply with study procedures.

Any participant who becomes pregnant during the trial will be followed up until the end of the pregnancy but no further immunisations will be given unless indicated during pregnancy (as is the case for tetanus toxoid). The trial allocation for this participant will be unblinded and the participant will only be given further treatment if clinically indicated. The babies will also be followed up and

For peer review only

examined for any adverse effects. We will not routinely perform venepuncture in a pregnant participant.

The reason for withdrawal will be recorded in the case report form (CRF). If withdrawal is due to an AE, appropriate follow-up visits or medical care will be arranged, with the agreement of the participant, until the AE has resolved, stabilised or a non-trial related causality has been assigned.

If a participant withdraws from the study samples collected before their withdrawal from the trial will be used/ stored unless the participant specifically requests otherwise.

Trial discontinuation

The trial will be discontinued in the event of new scientific information that renders continuation futile or unethical, or for any other reason, at the discretion of the Programme Steering Committee.

End of study definition

The trial will be completed when the last participant enrolled into the trial has completed their final follow up visit.

Safety assessments and oversight

No new investigational drug or product will be used in the proposed trial. However, standard approaches for monitoring safety and reporting of serious adverse events will be followed.

Monitoring

The trial will be monitored by both internal and external monitors according to a pre-defined monitoring plan which will include a site initiation visit, monitoring visits at least annually, and a close-out visit. The monitors will assess patient safety, data integrity, and adherence to the protocol and to Good Clinical Research Practice procedures.

Considerations regarding standard of care

S. mansoni infection status will be determined retrospectively through assays conducted in bulk on stored samples (plasma CAA). These results will not, therefore, be useful to determine management of individual participants.

Participants in the standard treatment arm will receive lower levels of anthelminthic treatment. However, all trial arms will receive a minimum of well-implemented national standard of care.

Standard of care will comprise annual praziquantel treatment. Our own results from the Lake Victoria Island Intervention Study on Worms and Allergy-related diseases (LaVIISWA),[28] which compared annual versus quarterly intervention for schistosomiasis at community level over three

For peer review only - http://bmjopen.bmj.com/site/about/guidelines.xhtml

years, showed no advantage of quarterly treatment for morbidity outcomes attributed to schistosomiasis.

Schistosomiasis can cause anaemia. To manage the expected differential benefits of the interventions for anaemia, a full blood count will be performed at baseline, as discussed above; anaemic children will be managed appropriately and severely anaemic children excluded.

Albendazole will be provided twice a year to manage nematode infections (after collection of primary and secondary endpoint samples).

Procedures to be followed in the event of abnormal findings

Abnormal clinical findings from medical history, examination or blood tests will be assessed as to their clinical significance throughout the trials. If an abnormal test result is deemed clinically significant, it may be repeated. If a test remains clinically significant, the participant will be informed and appropriate medical care arranged as appropriate and with the permission of the participant. Specific details regarding findings, discussion with participants and resulting actions will be recorded in the clinical records. Decisions to exclude the participant from enrolling in the trial or to withdraw a participant from the trial will be at the discretion of the Investigator.

Data and Safety Monitoring Board (DSMB)

A data and safety monitoring board (DSMB) has been appointed to provide real-time safety oversight. The DSMB will be notified within 7 days of the Investigators' being aware of the occurrence of SAEs. The DSMB may recommend the Investigators to place the trial on hold if deemed necessary following an intervention-related SAE. The DSMB will be chaired by a clinician experienced in clinical trials. There will be a minimum of two other appropriately qualified committee members. In the case of events related to a blinded intervention, the DSMB can request unblinding. Membership will include a statistician, and at least one Ugandan member. All correspondence between Investigators and the DSMB will be conveyed by the Principal Investigator to the trial Sponsor. The Chair of the DSMB will be contacted for advice and independent review by the Investigator or trial Sponsor in the following situations:

- The occurrence of any SAE
- Any other situation where the Investigator or trial Sponsor feels independent advice or review is important

Ethical and regulatory considerations

Further information regarding risks

For peer review only

The immunisations to be given have recognised side effects which are usually mild and resolve spontaneously in a few days to one week. Parenteral vaccines are likely to result in pain and swelling at the site of injection and mild fever; very occasionally pain and swelling can be severe and associated with difficulty in moving the shoulder. Sometimes headache and tiredness occur. Rarely a vaccine may cause a severe allergic reaction. For most vaccines this is estimated at less than one in a million doses (but 1 in 55,000 for Yellow Fever vaccine).[29] Individuals with a history of a possible allergic reaction to drugs or vaccines, or to vaccine components including eggs or chicken proteins, will be excluded from the studies. The research team will be trained and prepared to manage severe allergic reactions.

Adverse reactions to Yellow Fever vaccine include severe nervous system reaction (about 1 person in 125,000) and severe, life-threatening illness with organ failure (about 1 person in 250,000). The mortality for this severe, life-threatening adverse effect is reported as about 50%.[29]

BCG immunisation is likely to induce a scar in many cases. This may develop over several weeks, starting as a small papule at the injection site which may become ulcerated and then heal over a period of 2 to 5 months; and lymphadenopathy may develop. Occasionally a more severe local reaction occurs (estimated at 1 per 1,000-10,000 doses): for example, an abscess develops and scars may develop into keloids. Rarely BCG can cause disseminated disease (1 per 230,000 to 640,000 doses), or disease in sites remote from the immunisation site. Disseminated BCG disease usually occurs in immunocompromised people: HIV positive people will be excluded from these studies.[30] BCG "pre-immunisation" may interfere with the response to the subsequent live vaccines; indeed our hypothesis, and published results, suggest that it may suppress replication of YF 17D vaccine.[31] However, this reduced replication has not been shown to correlate with, or result in, reduced levels of neutralising antibody titres (which are the desired protective outcome).[13 31]

Oral typhoid vaccine (Ty21a) may occasionally be associated with stomach pain, nausea, vomiting and (rarely) rash.[29]

Praziquantel has been in use for about 30 years. It has a well-recognised profile of side effects including dizziness, nausea, vomiting, abdominal pain, diarrhoea (sometimes with blood) and urticarial rash. The symptoms are considered to arise largely from the effects of killing worms and to be more severe in people with heavy infections. Symptoms are better tolerated when the drugs are given after food and we will provide treatment after a meal or snack. Simple medications, such as paracetamol and cetirizine, can alleviate symptoms and these will be available on treatment days.

For peer review only

References

1. Wajja A, Kizito D, Nassanga B, et al. The effect of current Schistosoma mansoni infection on the immunogenicity of a candidate TB vaccine, MVA85A, in BCG-vaccinated adolescents: An open-label trial. *PLoS neglected tropical diseases* 2017;11(5):e0005440. doi: 10.1371/journal.pntd.0005440 [published Online First: 2017/05/05]

2. WHO. Growth reference 5-19 years. 2007

3. WHO. Global tuberculosis report 2016 http://www.who.int/tb/publications/global_report/en/ (accessed 17 June 2017), 2016.

4. Alcais A, Fieschi C, Abel L, et al. Tuberculosis in children and adults: two distinct genetic diseases. *J Exp Med* 2005;202(12):1617-21. doi: 10.1084/jem.20052302

5. Weiner J, 3rd, Kaufmann SH. Recent advances towards tuberculosis control: vaccines and biomarkers. *J Intern Med* 2014;275(5):467-80. doi: 10.1111/joim.12212

6. Fine PE. Variation in protection by BCG: implications of and for heterologous immunity. *Lancet (London, England)* 1995;346(8986):1339-45.

7. Barreto ML, Pilger D, Pereira SM, et al. Causes of variation in BCG vaccine efficacy: examining evidence from the BCG REVAC cluster randomized trial to explore the masking and the blocking hypotheses. *Vaccine* 2014;32(30):3759-64. doi: 10.1016/j.vaccine.2014.05.042

8. Black GF, Weir RE, Floyd S, et al. BCG-induced increase in interferon-gamma response to mycobacterial antigens and efficacy of BCG vaccination in Malawi and the UK: two randomised controlled studies. *Lancet (London, England)* 2002;359(9315):1393-401. doi: 10.1016/S0140-6736(02)08353-8

9. Tanner R, Kakalacheva K, Miller E, et al. Serum indoleamine 2,3-dioxygenase activity is associated with reduced immunogenicity following vaccination with MVA85A. *BMC infectious diseases* 2014;14:660. doi: 10.1186/s12879-014-0660-7 [published Online First: 2014/12/04]

10. WHO. SAGE Evidence to recommendations framework. 2017. http://www.who.int/immunization/sage/meetings/2017/october/2_EvidencetoRecommendationFramework_BCG.pdf (accessed 16th March 2018).

11. Dye C. Making wider use of the world's most widely used vaccine: Bacille Calmette-Guerin revaccination reconsidered. *Journal of the Royal Society, Interface* 2013;10(87):20130365. doi: 10.1098/rsif.2013.0365 [published Online First: 2013/08/02]

12. Kraemer MU, Faria NR, Reiner RC, Jr., et al. Spread of yellow fever virus outbreak in Angola and the Democratic Republic of the Congo 2015-16: a modelling study. *The Lancet Infectious diseases* 2016 doi: 10.1016/s1473-3099(16)30513-8 [published Online First: 2016/12/27]

13. Muyanja E, Ssemaganda A, Ngauv P, et al. Immune activation alters cellular and humoral responses to yellow fever 17D vaccine. *The Journal of clinical investigation* 2014;124(7):3147-58. doi: 10.1172/jci75429 [published Online First: 2014/06/10]

14. Aguiar M, Stollenwerk N, Halstead SB. The Impact of the Newly Licensed Dengue Vaccine in Endemic Countries. *PLoS neglected tropical diseases* 2016;10(12):e0005179. doi: 10.1371/journal.pntd.0005179 [published Online First: 2016/12/22]

15. Dharmasena MN, Osorio M, Filipova S, et al. Stable expression of Shigella dysenteriae serotype 1 O-antigen genes integrated into the chromosome of live Salmonella oral vaccine vector Ty21a. *Pathogens and disease* 2016 doi: 10.1093/femspd/ftw098 [published Online First: 2016/09/23]

For peer review only

For peer review only - http://bmjopen.bmj.com/site/about/guidelines.xhtml

16. Carias C, Walters MS, Wefula E, et al. Economic evaluation of typhoid vaccination in a prolonged typhoid outbreak setting: the case of Kasese district in Uganda. *Vaccine* 2015;33(17):2079-85. doi: 10.1016/j.vaccine.2015.02.027 [published Online First: 2015/02/26]

17. Melhem RF, LoVerde PT. Mechanism of interaction of Salmonella and Schistosoma species. *Infection and immunity* 1984;44(2):274-81. [published Online First: 1984/05/01]

18. Muniz-Junqueira MI, Tavares-Neto J, Prata A, et al. Antibody response to Salmonella typhi in human schistosomiasis mansoni. *Revista da Sociedade Brasileira de Medicina Tropical* 1996;29(5):441-5. [published Online First: 1996/09/01]

19. WHO. Position Paper on Typhoid vaccines: WHO position paper – March 2018 2018

20. Brown J, Baisley K, Kavishe B, et al. Impact of malaria and helminth infections on immunogenicity of the human papillomavirus-16/18 AS04-adjuvanted vaccine in Tanzania. *Vaccine* 2014;32(5):611-7. doi: 10.1016/j.vaccine.2013.11.061

21. Riner DK, Ndombi EM, Carter JM, et al. Schistosoma mansoni Infection Can Jeopardize the Duration of Protective Levels of Antibody Responses to Immunizations against Hepatitis B and Tetanus Toxoid. *PLoS neglected tropical diseases* 2016;10(12):e0005180. doi: 10.1371/journal.pntd.0005180

22. Centre HI. HPV and related diseases report: Uganda. 2016. http://www.hpvcentre.net/statistics/reports/UGA.pdf (accessed 20.01.2017).

23. WHO. Human papillomavirus vaccines: WHO position paper, May 2017. *Releve epidemiologique hebdomadaire* 2017;92(19):241-68. [published Online First: 2017/05/23]

24. Sabin EA, Araujo MI, Carvalho EM, et al. Impairment of tetanus toxoid-specific Th1-like immune responses in humans infected with Schistosoma mansoni. *The Journal of infectious diseases* 1996;173(1):269-72. [published Online First: 1996/01/01]

25. Nanteza B, Galukande M, Aceng J, et al. The burden of tetanus in Uganda. *SpringerPlus* 2016;5(1):705. doi: 10.1186/s40064-016-2309-z [published Online First: 2016/06/29]

26. Ministry of Health–Uganda. Uganda Clinical Guidelines 2016. http://apps.who.int/medicinedocs/documents/s23532en/s23532en.pdf

27. WHO. Haemoglobin concentrations for the diagnosis of anaemia and assessment of severity. Vitamin and Mineral Nutrition Information System. Geneva, World Health Organization, 2011 (WHO/NMH/NHD/MNM/11.1).

28. Sanya RE, Nkurunungi G, Hoek Spaans R, et al. The Impact of Intensive Versus Standard Anthelminthic Treatment on Allergy-related Outcomes, Helminth Infection Intensity, and Helminth-related Morbidity in Lake Victoria Fishing Communities, Uganda: Results From the LaVIISWA Cluster-randomized Trial. *Clinical Infectious Diseases* 2018:ciy761-ciy61. doi: 10.1093/cid/ciy761

29. CDC. Centers for Disease Control and Prevention, vaccines and immunizations.

30. WHO. Information sheet observed rate of vaccine reactions Bacille Calmette-Guérin (BCG) vaccine. 2012

31. Arts RJW, Moorlag S, Novakovic B, et al. BCG Vaccination Protects against Experimental Viral Infection in Humans through the Induction of Cytokines Associated with Trained Immunity. *Cell host & microbe* 2018;23(1):89-100.e5. doi: 10.1016/j.chom.2017.12.010 [published Online First: 2018/01/13]

For peer review only

Reporting checklist for protocol of a clinical trial.

Based on the SPIRIT guidelines.

Instructions to authors

Complete this checklist by entering the page numbers from your manuscript where readers will find each of the items listed below.

Your article may not currently address all the items on the checklist. Please modify your text to include the missing information. If you are certain that an item does not apply, please write "n/a" and provide a short explanation.

Upload your completed checklist as an extra file when you submit to a journal.

In your methods section, say that you used the SPIRITreporting guidelines, and cite them as:

Chan A-W, Tetzlaff JM, Altman DG, Laupacis A, Gøtzsche PC, Krleža-Jerić K, Hróbjartsson A, Mann H, Dickersin K, Berlin J, Doré C, Parulekar W, Summerskill W, Groves T, Schulz K, Sox H, Rockhold FW, Rennie D, Moher D. SPIRIT 2013 Statement: Defining standard protocol items for clinical trials. Ann Intern Med. 2013;158(3):200-207

		Reporting Item	Page Number
**Administrative information**			
Title	#1	Descriptive title identifying the study design, population, interventions, and, if applicable, trial acronym	1
Trial registration	#2a	Trial identifier and registry name. If not yet registered, name of intended registry	2
Trial registration: data set	#2b	All items from the World Health Organization Trial Registration Data Set	n/a
Protocol version	#3	Date and version identifier	Information available at ISRCTN60517191
Funding	#4	Sources and types of financial, material, and other support	15
Roles and	#5a	Names, affiliations, and roles of protocol	15

For peer review only

responsibilities: contributorship		contributors	
Roles and responsibilities: sponsor contact information	#5b	Name and contact information for the trial sponsor	Information available at ISRCTN60517191
Roles and responsibilities: sponsor and funder	#5c	Role of study sponsor and funders, if any, in study design; collection, management, analysis, and interpretation of data; writing of the report; and the decision to submit the report for publication, including whether they will have ultimate authority over any of these activities	16
Roles and responsibilities: committees	#5d	Composition, roles, and responsibilities of the coordinating centre, steering committee, endpoint adjudication committee, data management team, and other individuals or groups overseeing the trial, if applicable (see Item 21a for data monitoring committee)	Information detailed in supplementary information file
**Introduction**			
Background and rationale	#6a	Description of research question and justification for undertaking the trial, including summary of relevant studies (published and unpublished) examining benefits and harms for each intervention	4
Background and rationale: choice of comparators	#6b	Explanation for choice of comparators	8
Objectives	#7	Specific objectives or hypotheses	6
Trial design	#8	Description of trial design including type of trial (eg, parallel group, crossover, factorial, single group), allocation ratio, and framework (eg, superiority, equivalence, non-inferiority, exploratory)	7
**Methods: Participants, interventions, and outcomes**			

For peer review only

Study setting	#9	Description of study settings (eg, community clinic, academic hospital) and list of countries where data will be collected. Reference to where list of study sites can be obtained	7
Eligibility criteria	#10	Inclusion and exclusion criteria for participants. If applicable, eligibility criteria for study centres and individuals who will perform the interventions (eg, surgeons, psychotherapists)	7
Interventions: description	#11a	Interventions for each group with sufficient detail to allow replication, including how and when they will be administered	8
Interventions: modifications	#11b	Criteria for discontinuing or modifying allocated interventions for a given trial participant (eg, drug dose change in response to harms, participant request, or improving / worsening disease)	Detailed in supplementary information file
Interventions: adherance	#11c	Strategies to improve adherence to intervention protocols, and any procedures for monitoring adherence (eg, drug tablet return; laboratory tests)	Detailed in supplementary information file
Interventions: concomitant care	#11d	Relevant concomitant care and interventions that are permitted or prohibited during the trial	Detailed in supplementary information file
Outcomes	#12	Primary, secondary, and other outcomes, including the specific measurement variable (eg, systolic blood pressure), analysis metric (eg, change from baseline, final value, time to event), method of aggregation (eg, median, proportion), and time point for each outcome. Explanation of the clinical relevance of chosen efficacy and harm outcomes is strongly recommended	10
Participant timeline	#13	Time schedule of enrolment, interventions (including any run-ins and washouts), assessments, and visits for participants. A schematic diagram is highly recommended (see Figure)	14<br>Also detailed in supplementary information file, Table S1

For peer review only

Sample size	#14	Estimated number of participants needed to achieve study objectives and how it was determined, including clinical and statistical assumptions supporting any sample size calculations	11
Recruitment	#15	Strategies for achieving adequate participant enrolment to reach target sample size	Detailed in supplementary information file
**Methods: Assignment of interventions (for controlled trials)**			
Allocation: sequence generation	#16a	Method of generating the allocation sequence (eg, computer-generated random numbers), and list of any factors for stratification. To reduce predictability of a random sequence, details of any planned restriction (eg, blocking) should be provided in a separate document that is unavailable to those who enrol participants or assign interventions	8
Allocation concealment mechanism	#16b	Mechanism of implementing the allocation sequence (eg, central telephone; sequentially numbered, opaque, sealed envelopes), describing any steps to conceal the sequence until interventions are assigned	9
Allocation: implementation	#16c	Who will generate the allocation sequence, who will enrol participants, and who will assign participants to interventions	8, 9
Blinding (masking)	#17a	Who will be blinded after assignment to interventions (eg, trial participants, care providers, outcome assessors, data analysts), and how	9
Blinding (masking): emergency unblinding	#17b	If blinded, circumstances under which unblinding is permissible, and procedure for revealing a participant's allocated intervention during the trial	Detailed in supplementary information file
**Methods: Data collection, management, and analysis**			

For peer review only

Data collection plan	#18a	Plans for assessment and collection of outcome, baseline, and other trial data, including any related processes to promote data quality (eg, duplicate measurements, training of assessors) and a description of study instruments (eg, questionnaires, laboratory tests) along with their reliability and validity, if known. Reference to where data collection forms can be found, if not in the protocol	10.<br><br>More details in supplementary information file
Data collection plan: retention	#18b	Plans to promote participant retention and complete follow-up, including list of any outcome data to be collected for participants who discontinue or deviate from intervention protocols	Detailed in supplementary information file
Data management	#19	Plans for data entry, coding, security, and storage, including any related processes to promote data quality (eg, double data entry; range checks for data values). Reference to where details of data management procedures can be found, if not in the protocol	13.<br><br>More information in the statistical analysis plan found at ISRCTN60517191
Statistics: outcomes	#20a	Statistical methods for analysing primary and secondary outcomes. Reference to where other details of the statistical analysis plan can be found, if not in the protocol	13.<br><br>More information in the statistical analysis plan found at ISRCTN60517191
Statistics: additional analyses	#20b	Methods for any additional analyses (eg, subgroup and adjusted analyses)	11.<br><br>More information in the statistical analysis plan found at ISRCTN60517191
Statistics: analysis population and missing data	#20c	Definition of analysis population relating to protocol non-adherence (eg, as randomised analysis), and any statistical methods to handle missing data (eg, multiple imputation)	Information in the statistical analysis plan found at ISRCTN60517191
**Methods: Monitoring**			

For peer review only

Data monitoring: formal committee	#21a	Composition of data monitoring committee (DMC); summary of its role and reporting structure; statement of whether it is independent from the sponsor and competing interests; and reference to where further details about its charter can be found, if not in the protocol. Alternatively, an explanation of why a DMC is not needed	Detailed in supplementary information file
Data monitoring: interim analysis	#21b	Description of any interim analyses and stopping guidelines, including who will have access to these interim results and make the final decision to terminate the trial	Detailed in supplementary information file
Harms	#22	Plans for collecting, assessing, reporting, and managing solicited and spontaneously reported adverse events and other unintended effects of trial interventions or trial conduct	Detailed in supplementary information file
Auditing	#23	Frequency and procedures for auditing trial conduct, if any, and whether the process will be independent from investigators and the sponsor	Detailed in supplementary information file
**Ethics and dissemination**			
Research ethics approval	#24	Plans for seeking research ethics committee / institutional review board (REC / IRB) approval	12
Protocol amendments	#25	Plans for communicating important protocol modifications (eg, changes to eligibility criteria, outcomes, analyses) to relevant parties (eg, investigators, REC / IRBs, trial participants, trial registries, journals, regulators)	11
Consent or assent	#26a	Who will obtain informed consent or assent from potential trial participants or authorised surrogates, and how (see Item 32)	12.<br>Also detailed in supplementary information file
Consent or assent: ancillary studies	#26b	Additional consent provisions for collection and use of participant data and biological specimens in ancillary studies, if applicable	Detailed in supplementary information file

For peer review only

Confidentiality	#27	How personal information about potential and enrolled participants will be collected, shared, and maintained in order to protect confidentiality before, during, and after the trial	Detailed in supplementary information file
Declaration of interests	#28	Financial and other competing interests for principal investigators for the overall trial and each study site	15
Data access	#29	Statement of who will have access to the final trial dataset, and disclosure of contractual agreements that limit such access for investigators	n/a
Ancillary and post trial care	#30	Provisions, if any, for ancillary and post-trial care, and for compensation to those who suffer harm from trial participation	Detailed in supplementary information file
Dissemination policy: trial results	#31a	Plans for investigators and sponsor to communicate trial results to participants, healthcare professionals, the public, and other relevant groups (eg, via publication, reporting in results databases, or other data sharing arrangements), including any publication restrictions	2, 13
Dissemination policy: authorship	#31b	Authorship eligibility guidelines and any intended use of professional writers	n/a
Dissemination policy: reproducible research	#31c	Plans, if any, for granting public access to the full protocol, participant-level dataset, and statistical code	13
**Appendices**			
Informed consent materials	#32	Model consent form and other related documentation given to participants and authorised surrogates	n/a
Biological specimens	#33	Plans for collection, laboratory evaluation, and storage of biological specimens for genetic or molecular analysis in the current trial and for future use in ancillary studies, if applicable	n/a

None The SPIRIT checklist is distributed under the terms of the Creative Commons Attribution License CC-BY-ND 3.0. This checklist can be completed online using https://www.goodreports.org/, a tool made by the EQUATOR Network in collaboration with Penelope.ai

For peer review only

BMJ Open

The effect of intensive treatment for schistosomiasis on immune responses to vaccines among rural Ugandan island adolescents: randomised controlled trial protocol A for the 'POPulation differences in VACcine responses' (POPVAC) programme

Journal:	*BMJ Open*
Manuscript ID	bmjopen-2020-040426.R1
Article Type:	Protocol
Date Submitted by the Author:	05-Oct-2020
Complete List of Authors:	Nkurunungi, Gyaviira; MRC/UVRI and LSHTM Uganda Research Unit, Immunomodulation and Vaccines Programme<br>Zirimenya , Ludoviko ; MRC/UVRI and LSHTM Uganda Research Unit, Immunomodulation and Vaccines Programme<br>Nassuuna , Jacent; MRC/UVRI and LSHTM Uganda Research Unit, Immunomodulation and Vaccines Programme<br>Natukunda , Agnes ; MRC/UVRI and LSHTM Uganda Research Unit, Immunomodulation and Vaccines Programme<br>Kabuubi , Prossy ; MRC/UVRI and LSHTM Uganda Research Unit, Immunomodulation and Vaccines Programme<br>Niwagaba , Emmanuel ; MRC/UVRI and LSHTM Uganda Research Unit, Immunomodulation and Vaccines Programme<br>Oduru, Gloria; MRC/UVRI and LSHTM Uganda Research Unit, Immunomodulation and Vaccines Programme<br>Kabami , Grace ; MRC/UVRI and LSHTM Uganda Research Unit, Immunomodulation and Vaccines Programme<br>Amongin , Rebecca ; MRC/UVRI and LSHTM Uganda Research Unit, Immunomodulation and Vaccines Programme<br>Mutebe , Alex ; MRC/UVRI and LSHTM Uganda Research Unit, Immunomodulation and Vaccines Programme<br>Namutebi , Milly ; MRC/UVRI and LSHTM Uganda Research Unit, Immunomodulation and Vaccines Programme<br>Zziwa , Christopher ; MRC/UVRI and LSHTM Uganda Research Unit, Immunomodulation and Vaccines Programme<br>Amongi, Susan; MRC/UVRI and LSHTM Uganda Research Unit, Immunomodulation and Vaccines Programme<br>Ninsiima , Caroline ; MRC/UVRI and LSHTM Uganda Research Unit, Immunomodulation and Vaccines Programme<br>Onen, Caroline; MRC/UVRI and LSHTM Uganda Research Unit, Immunomodulation and Vaccines Programme<br>Akello , Florence ; MRC/UVRI and LSHTM Uganda Research Unit, Immunomodulation and Vaccines Programme<br>Sewankambo, Moses; MRC/UVRI and LSHTM Uganda Research Unit, Immunomodulation and Vaccines Programme<br>Kiwanuka, Samuel; MRC/UVRI and LSHTM Uganda Research Unit, Immunomodulation and Vaccines Programme<br>Kizindo , Robert ; MRC/UVRI and LSHTM Uganda Research Unit,

	Immunomodulation and Vaccines Programme<br>Kaweesa, James; Republic of Uganda Ministry of Health, Vector Control Division<br>Cose , Stephen ; MRC/UVRI and LSHTM Uganda Research Unit, Immunomodulation and Vaccines Programme; London School of Hygiene and Tropical Medicine Department of Clinical Research<br>Webb, Emily; London School of Hygiene & Tropical Medicine, MRC Tropical Epidemiology Group, Department of Infectious Disease Epidemiology<br>Elliott, Alison ; MRC/UVRI and LSHTM Uganda Research Unit, Immunomodulation and Vaccines Programme; London School of Hygiene and Tropical Medicine Department of Clinical Research
**Primary Subject Heading**:	Infectious diseases
Secondary Subject Heading:	Epidemiology, Immunology (including allergy), Public health
Keywords:	Infection control < INFECTIOUS DISEASES, PARASITOLOGY, Public health < INFECTIOUS DISEASES, Immunology < TROPICAL MEDICINE, Epidemiology < TROPICAL MEDICINE, Paediatric infectious disease & immunisation < PAEDIATRICS

SCHOLARONE™
Manuscripts

I, the Submitting Author has the right to grant and does grant on behalf of all authors of the Work (as defined in the below author licence), an exclusive licence and/or a non-exclusive licence for contributions from authors who are: i) UK Crown employees; ii) where BMJ has agreed a CC-BY licence shall apply, and/or iii) in accordance with the terms applicable for US Federal Government officers or employees acting as part of their official duties; on a worldwide, perpetual, irrevocable, royalty-free basis to BMJ Publishing Group Ltd ("BMJ") its licensees and where the relevant Journal is co-owned by BMJ to the co-owners of the Journal, to publish the Work in this journal and any other BMJ products and to exploit all rights, as set out in our licence.

The Submitting Author accepts and understands that any supply made under these terms is made by BMJ to the Submitting Author unless you are acting as an employee on behalf of your employer or a postgraduate student of an affiliated institution which is paying any applicable article publishing charge ("APC") for Open Access articles. Where the Submitting Author wishes to make the Work available on an Open Access basis (and intends to pay the relevant APC), the terms of reuse of such Open Access shall be governed by a Creative Commons licence – details of these licences and which Creative Commons licence will apply to this Work are set out in our licence referred to above.

Other than as permitted in any relevant BMJ Author's Self Archiving Policies, I confirm this Work has not been accepted for publication elsewhere, is not being considered for publication elsewhere and does not duplicate material already published. I confirm all authors consent to publication of this Work and authorise the granting of this licence.

For peer review only

The effect of intensive treatment for schistosomiasis on immune responses to vaccines among rural Ugandan island adolescents: randomised controlled trial protocol A for the 'POPulation differences in VACcine responses' (POPVAC) programme

Gyaviira Nkurunungi[1,¶,*], Ludoviko Zirimenya[1,¶], Jacent Nassuuna[1,¶], Agnes Natukunda[1,¶], Prossy N Kabuubi[1], Emmanuel Niwagaba[1], Gloria Oduru[1], Grace Kabami[1], Rebecca Amongin[1], Alex Mutebe [1], Milly Namutebi[1], Christopher Zziwa[1], Susan Amongi[1], Caroline Ninsiima[1], Caroline Onen[1], Florence Akello[1], Moses Sewankambo[1], Samuel Kiwanuka[1], Robert Kizindo[1], James Kaweesa[2], Stephen Cose[1,3], Emily L Webb[4], Alison M Elliott[1,3] **for the POPVAC trial team**

[1]Immunomodulation and Vaccines Programme, Medical Research Council/Uganda Virus Research Institute and London School of Hygiene and Tropical Medicine (MRC/UVRI and LSHTM) Uganda Research Unit, Entebbe, Uganda

[2]Vector Control Division, Ministry of Health, Kampala, Uganda

[3]Department of Clinical Research, London School of Hygiene and Tropical Medicine, London, United Kingdom

[4]MRC Tropical Epidemiology Group, Department of Infectious Disease Epidemiology, London School of Hygiene and Tropical Medicine, London, United Kingdom

[¶]These authors contributed equally

***Correspondence:** Gyaviira Nkurunungi; Gyaviira.Nkurunungi@mrcuganda.org

For peer review only

For peer review only - http://bmjopen.bmj.com/site/about/guidelines.xhtml

Abstract

Introduction

Several licensed and investigational vaccines have lower efficacy, and induce impaired immune responses, in low-income versus high-income countries and in rural, versus urban, settings. Understanding these population differences is essential to optimising vaccine effectiveness in the tropics. We suggest that repeated exposure to and immunomodulation by chronic helminth infections partly explains population differences in vaccine response.

Methods and analysis

We have designed an individually randomised, parallel group trial of intensive versus standard praziquantel (PZQ) intervention against schistosomiasis, to determine effects on vaccine response outcomes among school-going adolescents (9 to 17 years) from rural *Schistosoma mansoni* (*Sm*)-endemic Ugandan islands. Vaccines to be studied comprise BCG on day 'zero'; yellow fever, oral typhoid and Human Papilloma Virus (HPV) vaccines at week 4; and HPV and Tetanus/diphtheria booster vaccine at week 28. The intensive arm will receive PZQ doses three times, each two weeks apart, before BCG immunisation, followed by a dose at week 8 and quarterly thereafter. The standard arm will receive PZQ at week 8 and week 52. We expect to enrol 480 participants, with 80% *Sm* infected at the outset.

Primary outcomes are BCG-specific IFN-γ ELISpot responses eight weeks after BCG immunisation and for other vaccines, antibody responses to key vaccine antigens at four weeks after immunisation. Secondary analyses will determine effects of intensive anthelminthic treatment on correlates of protective immunity, on vaccine response waning, on priming vs boosting immunisations, and on *Sm* infection status and intensity. Exploratory immunology assays using archived samples will enable assessment of mechanistic links between helminths and vaccine responses.

Ethics and dissemination

Ethics approval has been obtained from relevant Ugandan and UK ethics committees. Results will be shared with Uganda Ministry of Health, relevant district councils, community leaders and study participants. Further dissemination will be done through conference proceedings and publications.

Trial registration

Current Controlled Trials identifier: ISRCTN60517191.

For peer review only

Article summary

Strengths and limitations of this study

- This will be the first adequately powered intervention study to investigate effects of schistosomiasis treatment on vaccine responses in adolescents.
- Effects on both live-attenuated and inert vaccines will be studied.
- Our strong immunoepidemiological design and nested immunological studies will address specific hypotheses regarding pathways of effects.
- The sample archives developed will provide a major asset for exploration of new leads arising from this hypothesis-driven work, or for an alternative, “systems biology” approach investigating (for example) transcriptome, microbiome and virome.
- Even with intensive anthelminthic intervention, it may be difficult to “successfully” treat *Schistosoma* infection in our endemic setting due to re-infections; however, we still expect a substantial difference in intensity between the two trial arms.

Word count

3250

Keywords

Vaccine; Schistosomiasis; Praziquantel; Immunization

For peer review only

For peer review only - http://bmjopen.bmj.com/site/about/guidelines.xhtml

Introduction

Vaccine-specific immune responses are often impaired, and vaccine efficacy and effectiveness lower, in tropical low-income countries (LICs) compared to temperate high-income countries and in rural, compared to urban, LIC settings.[1-8] This has been recognised for both live vaccines (such as BCG,[2 3 5 9] polio,[1] yellow fever[4] vaccines) and non-live vaccines (such as influenza[10] and tetanus[11]). Investigational malaria[7] and viral-vectored tuberculosis[6] and Ebola[12] vaccines are also affected. Previous exposure to the target pathogen (or related organisms) may mask the benefit of the vaccine.[13 14] However, pre-vaccination exposure does not explain why Ebola trial vaccine-specific responses differ between healthy UK and Senegalese adults,[12] as the target organism is rare. Therefore, environmentally-dependent mechanisms may play an important role.[5]

A long-held hypothesis is that parasites, particularly helminths, modulate vaccine responses through profound pre- and post-immunisation bystander effects on immunological activation and regulation.[15-17] Helminths might also impact vaccines responses through interactions with the complex ecosystem of mammalian gut bacteria, fungi, protozoa and viruses (the"trans-kingdom" concept[18] detailed elsewhere in this journal [bmjopen-2020-040425]). Helminth-induced gut mucosa damage, the associated translocation of microbial products into the systemic circulation[19-21] and systemic immune activation or regulation mediated by microbial products might contribute to modulation of responses to vaccines and other infections.

Helminth-mediated modulation of vaccine responses has not been substantiated in human populations. No appropriately powered trials have been conducted to evaluate reversibility of their effects. In animal models, helminths generally impair priming and accelerate waning of vaccine responses, although effects vary with helminth species, vaccine type and the timing of infection and immunisation.[22] Most observational studies in humans also suggest suppressed or biased responses during helminth infection, especially during systemic infections, such as schistosomiasis and the filariases. There is modest evidence that treating geohelminths in humans improves responses to BCG[23 24] or oral cholera vaccine[25] and we found that schistosomiasis treatment improved the measles-booster response in pre-school children.[26] There is therefore a strong case for a comprehensive assessment of the effects of helminths and their treatment on vaccine responses.

The extent to which helminths and related "trans-kingdom" mediators causally and reversibly impact immunological characteristics associated with vaccine responses may best be determined by intervention studies. This trial protocol A of the 'Population differences in Vaccine responses" programme (POPVAC A; Current Controlled Trials identifier: ISRCTN60517191) has been designed to evaluate the effect of *Schistosoma mansoni* and its treatment on vaccine responses. This study is

For peer review only - http://bmjopen.bmj.com/site/about/guidelines.xhtml

one of three parallel trials whose designs and cross-cutting analyses are described separately in this journal (bmjopen-2020-040425, bmjopen-2020-040427 and bmjopen-2020-040430).

For peer review only

For peer review only - http://bmjopen.bmj.com/site/about/guidelines.xhtml

Hypothesis

The overarching goal of the POPVAC programme is to understand population differences in vaccine responses in Uganda, in order to identify strategies through which vaccine effectiveness can be optimised for the low-income, tropical settings where they are especially needed. For this Trial A, we focus on the hypothesis that *Schistosoma mansoni infection suppresses responses to unrelated vaccines; and that this effect can be reversed, at least in part, by intensive praziquantel treatment intervention.*

Objective

To determine whether there are reversible effects of chronic *Schistosoma mansoni* infection on vaccine response in adolescents, using an intervention study.

For peer review only

For peer review only - http://bmjopen.bmj.com/site/about/guidelines.xhtml

Methods and analysis

Setting and participants

SPIRIT reporting guidelines[27] have been used. We will conduct an individually randomised, parallel group trial of intensive versus standard intervention against schistosomiasis (described below) in the *S. mansoni*-endemic Koome islands of Lake Victoria, Mukono district, Uganda.[28] We aim to enroll 480 participants, randomising 240 to each intervention arm. The study cohort will recruit participants aged 9 to 17 years in primary school years 1 to 6. Adolescents[29] in this study setting bear a heavy parasite burden.[30] In addition, this age-group is a target group for vaccines against sexually transmitted infections (currently human papilloma virus [HPV] – in future, it is hoped, for vaccines against HIV) and for booster immunisations.

Recruitment criteria

Inclusion criteria

i. Attending the selected school and planning to continue to attend the school for the duration of the study
ii. Aged 9 to 17 years and enrolled in primary 1 to 6 (to avoid primary leaving examinations in late year 7, and loss to follow up of children leaving after primary 7)
iii. Written informed assent by participant and consent by parent or guardian
iv. Females agree to avoid pregnancy for the duration of the trial
v. Willing to provide locator information and to be contacted during the course of the trial
vi. Able and willing (in the investigator's opinion) to comply with all the study requirements

Exclusion criteria

i. Clinically significant history of immunodeficiency (including HIV), cancer, cardiovascular disease, gastrointestinal disease, liver disease, renal disease, endocrine disorder and neurological illness
ii. History of serious psychiatric condition or disorder
iii. Moderate or severe acute illness characterised by any of the following symptoms: fever, impaired consciousness, convulsions, difficulty in breathing, vomiting; or as otherwise determined by the attending project clinician.
iv. Concurrent oral or systemic steroid medication or the concurrent use of other immunosuppressive agents within 2 months prior to enrolment
v. History of allergic reaction to immunisation or any allergy likely to be exacerbated by any component of the study vaccines including egg or chicken proteins

For peer review only - http://bmjopen.bmj.com/site/about/guidelines.xhtml

vi. History of previous immunisation with Yellow Fever (YF), oral typhoid or Human Papillomavirus (HPV) vaccine; previous immunisation with BCG or Tetanus and diphtheria vaccine (Td) at age ≥5 years

vii. Tendency to develop keloid scars

viii. Haemoglobin less than 82g/L

ix. Positive HIV serology

x. Positive pregnancy test

xi. Female currently lactating, confirmed pregnancy or intention to become pregnant during the trial period

xii. Use of an investigational medicinal product or non-registered drug, live vaccine, or medical device other than the study vaccines for 30 days prior to dosing with the study vaccine, or planned use during the study period

xiii. Administration of immunoglobulins and/or any blood products within the three months preceding the planned trial immunisation date

Further information on recruitment criteria can be found in Supplementary information.

Interventions

We will individually randomise participants to intensive or standard praziquantel (PZQ) treatment, in a 1:1 ratio. The intensive arm will receive three doses of PZQ (approximately 40mg/kg, assessed by height pole[31]) each two weeks apart prior to the first immunisation (the last of these 2-3 weeks before immunisation), followed by PZQ at 8 weeks (after primary endpoint sampling) and thereafter quarterly PZQ (approximately; timings adjusted to accommodate school terms) during follow up. The standard arm will receive their first dose of PZQ at week 8 (after immunisation and after primary endpoint sampling) and a second dose at week 52 (to conform to Uganda Ministry of Health policy, which is annual treatment) (**Figure 1**). No placebo will be used in this trial because all participants will be treated (albeit at different frequencies) and participants are unlikely to seek additional treatments outside the trial schedule: praziquantel treatment is not popular because of the recognised (although temporary) adverse effects (described in Supplementary information).

Randomisation and allocation to treatment arm

A randomisation code will be generated by an independent statistician using a randomly permuted block size (sizes 4, 6, 8 and 10) and used to allocate participants to either receive quarterly PZQ (intensive arm) or annual PZQ (standard arm). A set of envelopes will be prepared, labelled sequentially with the randomisation numbers and containing a card indicating the corresponding allocation (to intensive or standard treatment). The randomisation code will be kept securely by the

For peer review only - http://bmjopen.bmj.com/site/about/guidelines.xhtml

For peer review only

trial statistician and made available only to those responsible for providing or preparing the trial interventions. A second copy will be held by a data manager or statistician not otherwise involved in the trial at the MRC/UVRI and LSHTM Uganda Research Unit. At enrolment, eligibility criteria will be checked and eligible participants will be allocated sequentially to the next randomisation number until the required sample size is achieved. Randomisation implementation will be done by a clinician using the sequentially numbered opaque sealed envelopes. When the next randomisation number in the sequence is allocated, the envelope bearing that number will be opened to reveal the allocation.

Blinding

Clinicians and participants will not be blinded to the treatment allocation since they will not participate in outcome ascertainment; only immunology laboratory staff who are assessing trial outcomes will be blinded.

Immunisations

We will study a portfolio of licensed vaccines (live and inert, oral and parental, priming and boosting) expected to be beneficial (in some cases, already given) to adolescents in Uganda. Our schedule (**Table 1,** supplementary **Table S1**) will comprise three main immunisation days (week 0, week 4 and week 28). Additional HPV immunisation will be provided for girls aged 14 years or above, and a second Tetanus/diphtheria boost will be given after completion of the study, to accord with the national Expanded Programme on Immunisation (EPI) routines, but the response to these will not specifically be assessed. Further rationale for the selection of vaccines is detailed in the Supplementary information.

Table 1. Immunisation schedule

	**Immunisation week 0**	**Immunisation week 4**	**[Immunisation week 8]**	**Immunisation week 28**	**[Immunisation week 52]**
**Live vaccines**	BCG vaccination / re-vaccination[1]	Yellow fever (YF-17D)<br>Oral typhoid (Ty21a)			
**Non-live vaccines**		HPV prime[2]	HPV boost for girls aged ≥14 years[3,4]	HPV boost[2] and Tetanus/ diphtheria (Td) boost	Tetanus/ diphtheria (Td) boost[4,5]

1. Prior BCG status may vary (data on history and documentation of prior BCG, and presence of a BCG scar, will be documented although these approaches have limitations for determining BCG status)
2. Both girls and boys will receive the HPV vaccine
3. The National EPI programme recommends three doses of HPV vaccine for older girls
4. These doses will be given to comply with guidelines but outcomes specifically relating to these doses will not be assessed
5. Priming by immunisation in infancy is assumed

For peer review only

For peer review only - http://bmjopen.bmj.com/site/about/guidelines.xhtml

Schedule of immunisation and sampling

The schedule of immunisation and sampling is outlined in **Figure 1** and **Table S1**. While optimal timings for outcome measures vary between vaccines, sampling at 8 weeks post BCG and 4 weeks post YF-17D, Ty21a, HPV and Td is proposed for the primary endpoints, targeting the establishment of memory responses and approximate peak of antibody responses. A secondary endpoint at one year will assess waning. All analyses will take baseline measurements into account. Immunisation postponement criteria are detailed in Supplementary information.

Outcomes

Primary outcomes

These will be assessed in all participants.

i. **BCG:** BCG-specific IFN-γ ELISpot response eight weeks post BCG immunisation.
ii. **YF-17D:** neutralising antibody titres (plaque-reduction neutralisation test) at four weeks post YF immunisation.
iii. **Ty21a:** *Salmonella typhi* lipopolysaccharide (LPS)-specific immunoglobulin (Ig)G concentration at four weeks post Ty21a immunisation.
iv. **HPV:** IgG specific for L1-proteins of HPV-16/18 at four weeks post HPV priming immunisation.
v. **Td:** Tetanus and diphtheria toxoid-specific IgG concentration at four weeks post Td immunisation.

Secondary outcomes

These will be assessed in all participants and will further investigate estimates of protective immunity (for vaccines where these are available) and dynamics of the vaccine responses, as well as the impact of the interventions on parasite clearance.

i. **Protective immunity.** Proportions with protective neutralising antibody (YF); protective IgG levels (TT);[32] seroconversion rates (Ty21a) at four weeks post the corresponding immunisation.
ii. **Response waning.** Primary outcome measures (all vaccines) repeated at week 52, and area-under-the curve (AUC) analyses. Parasitic infection may accelerate,[33] and anti-parasitic interventions delay, waning.
iii. **Priming versus boosting.** Effects on priming versus boosting will be examined for HPV only, comparing outcomes four weeks after the first, and four weeks after the second vaccine dose.

For peer review only - http://bmjopen.bmj.com/site/about/guidelines.xhtml

iv. **Current *S. mansoni* infection status and intensity** will be determined by serum/plasma levels of circulating anodic antigen (CAA). The method is quantitative, highly specific for *Schistosoma* infection, and much more sensitive than the conventional Kato Katz method.[34] CAA will be assessed retrospectively on stored samples collected at baseline, on immunisation days, and on primary and secondary endpoint days.

Furthermore, our sample collection will offer opportunities for an array of exploratory immunological evaluations on stored samples, focusing mainly on vaccine antigen specific outcomes. Exploratory assays will provide further detail on the role of immunological profiles and trans-kingdom effects in mediating helminth modulation of vaccine-specific responses.

Additional evaluation of parasite infection exposure

i. **Prior exposure to schistosomiasis** will be evaluated by ELISA for IgG to schistosome egg antigen using stored blood samples collected at baseline.

ii. **The presence of other helminth infections** will be determined retrospectively using stool PCR of samples collected at baseline and at weeks 28 and 52.[30] In accordance with national guidelines, all participants will be treated with albendazole or mebendazole after collection of samples for primary endpoints at week 8 and 28, and after collection of samples for secondary endpoints at week 52.

iii. **Current malaria infection status and intensity** will be assessed retrospectively by PCR on stored samples collected on immunisation days and at week 52. Individuals presenting with fever will be investigated using rapid diagnostic tests for malaria and treated based on the results and according to prevailing national guidelines.

iv. **Prior malaria exposure** will be evaluated by ELISA for IgG to malaria antigen using stored samples collected at baseline.

Sample size considerations

Based on the literature[4 35 36] and preliminary data, we anticipate that, following log to base 10 transformations that will be applied to normalise primary outcome measures, standard deviations (SDs) of primary outcome measures will lie between 0.3 and 0.6 on this log scale, and that effective treatment may increase responses by approximately 0.2 on the log scale (based on Tweyongyere *et al.*[26]). We have therefore powered our study to detect differences of this magnitude (0.2 on the log scale) or (in some cases) smaller (**Table 2**). We assume *S. mansoni* prevalence of ≥80%.

For peer review only

For peer review only - http://bmjopen.bmj.com/site/about/guidelines.xhtml

260 Based on these assumptions, we plan to include 480 participants in total (240 quarterly PZQ, 240
261 annual PZQ); of whom 384 are expected to be *S. mansoni* infected,[28] giving 192 participants in each
262 trial arm who are infected at baseline.

263 **Table 2** shows power estimates, for 5% significance level and assuming 20% loss to follow-up.

264

Table 2. Power estimates (5% significance level)

Standard deviation (log_{10})	Difference in mean log_{10} transformed outcome, between trial arms						
	**0.08**	**0.10**	**0.12**	**0.14**	**0.16**	**0.18**	**0.20**
192 intensive PZQ vs 192 standard PZQ (*S. mansoni* infected only)							
0.3	65%	83%	94%	98%	>99%	>99%	>99%
0.4	42%	59%	75%	87%	94%	98%	99%
0.5	29%	42%	56%	69%	80%	88%	94%
0.6	21%	31%	42%	53%	65%	75%	83%

Cells highlighted in grey correspond to >80% power; differences in mean log10 transformed outcome of 0.08, 0.10, 0.12, 0.14, 0.16, 0.18 and 0.20 are equivalent to geometric mean ratios for untransformed outcomes of 1.20, 1.26, 1.32, 1.38, 1.45, 1.51 and 1.59, respectively.

265

266 ***Ethics and dissemination***

267 Ethical approval has been granted from the Research Ethics Committee of the Uganda Virus
268 Research Institute (UVRI REC, reference: GC/127/19/05/664) and the London School of Hygiene and
269 Tropical Medicine (LSHTM, reference: 16032), and from the Uganda National Council for Science and
270 Technology (UNCST, reference: HS2486) and the Uganda National Drug Authority (NDA, reference:
271 CTA0093). Any protocol amendments will be submitted to ethics committees and regulatory bodies
272 for approval before implementation.

273 Participants are adolescents and therefore a vulnerable human population. Care will be taken to
274 provide adequate, age and education-status appropriate information and to ensure that it is
275 understood; and to emphasise that participation is voluntary. Participants will be enrolled only when
276 they have given their own assent and when consent has been given by the parent or guardian. No
277 major risks to the participants are anticipated since all the treatments and vaccines to be given are
278 licensed and known to be safe. The main risk to participants will be time lost from school work: we
279 will work with teachers and parents to minimise disruption to classes, and will avoid enrolment of
280 primary 7 students since these classes are involved in national examinations. Further risks are
281 discussed in Supplementary information.

282 Study findings will be published through open access peer-reviewed journals, presentations at local,
283 national and international conferences and to the local community through community meetings.
284 Anonymised participant level datasets generated will be available upon request.

For peer review only - http://bmjopen.bmj.com/site/about/guidelines.xhtml

Patient and public involvement

Concepts involved in this work have been discussed with colleagues at the Vector Control Division and Expanded Programme on Immunisation in the Ministry of Health (Uganda), with the Mukono District Council and with community leaders and Village Health Teams from Koome subcounty. We also have held meetings to explain the proposed work to teachers, parents, participants and village members, and to address their questions about issues such as study length, the study's ethical approval status, why adults were excluded from the study, and to explain to them why boys will also receive the HPV vaccine. Study findings will be shared with these stakeholders and with participants.

Data management and analysis

Socio-demographic information and clinical and laboratory measurements will be recorded and managed using Research Electronic Data Capture (REDCap) tools,[37 38] with paper-based forms as back-up. All data will be recorded under a unique study ID number. When paper forms must be used, data will be double entered in a study-specific database, with standard checks for discrepancies. All data for analysis will be anonymised and stored on a secure and password-protected server, with access limited to essential research personnel.

Baseline characteristics will be summarised by trial arm, and the effect of intensive (compared to standard) praziquantel treatment on the outcomes will be analysed. Information on infection status will only be available after randomisation. The primary analysis will be done on individuals identified as infected at baseline (through randomisation, these will be balanced between treatment arms); this will test the hypothesis that treating the infection (and subsequent reinfections) reverses the parasite's effects on vaccine responses. If treating *S. mansoni* reverses adverse parasite effects on vaccine responses, this may be a beneficial public health intervention. However, routine screening for parasite infection before immunisation would be laborious. Secondary analyses will include all randomised individuals; this will provide insight into the broader benefit of the interventions as public health measures. The effect of intensive versus standard praziquantel treatment on primary outcomes will be assessed using unpaired t-tests, with results presented as a mean difference in vaccine response measure together with 95% confidence interval and p-value. We anticipate that outcomes will be positively skewed, and will apply log transformations to normalise distributions before analysis if required. The detailed analytical plan is available on the online trial registration site (http://www.isrctn.com/ISRCTN60517191).

For peer review only - http://bmjopen.bmj.com/site/about/guidelines.xhtml

Discussion

This will be the first adequately powered intervention study to investigate the effect of schistosomiasis treatment on vaccine responses in adolescents. This study will determine whether *S. mansoni* infection has a causal, reversible, impact on the response to live or inert vaccines, including effects on vaccine replication, immune response profile, priming, boosting and waning. The results will add to understanding of population differences in vaccine responses and on interventions that may enhance responses. If treating helminths improves vaccine responses in adolescents, combined parasite-control/immunisation programmes offer an attractive, practical public health intervention for schools and communities.

There are risks associated with our approach to addressing the trial objective. **First**, there is a risk of failure to clear *S. mansoni* infections, and repeated reinfection during the trial. This issue can be challenging because of incomplete cure or maturation of immature worms after treatment, and lifestyles in endemic communities that result in repeated exposure. To mitigate this, we will administer three PZQ treatments over a six-week period before the first immunisations, and continuing quarterly treatment in the intensive arm. **Second,** there is a risk that *S. mansoni* infection has long-term effects, not removed by treatment, mediated, for example, by epigenetic change.[39] However, studies show that parasite treatment results in immunological changes,[40 41] and our data suggest at least partial recovery of the measles vaccine response among young children treated for schistosomiasis.[26] By initiating intervention six to eight weeks before the first immunisations, and providing repeated intervention in the intensive arms, we hope to achieve significant resolution of *S. mansoni* effects.

We are interested in the effects of removing *S. mansoni*. Treating parasites can induce acute immunological change due to release of previously hidden antigens.[42 43] To minimise such effects, immunisations will be given at least 2 weeks after PZQ (the longest practicable interval; **Figure 1**).

Laboratory analyses will also highlight immune parameters and cellular populations that link environmental exposures to vaccine responses. Identifying processes associated with poor or good outcomes will inform strategies in vaccine design (for example, the genetic modification of vaccines, or innovative use of adjuvants, to counter any adverse immunological milieu, currently an area of intense research for cancer vaccines[44]); ultimately supporting the development of effective vaccines tailored to the low-income settings that most need them.

Study timeline

For peer review only

Applications for ethical approval were submitted in May 2018, with approval received in September 2018 (Uganda Virus Research Institute Research Ethics Committee), May 2019 (National Drug Authority and Uganda National Council for Science and Technology), June 2019 (London School of Hygiene and Tropical Medicine). Collaborator/investigator/trial steering committee meetings were also held during the initial 12-month planning period. The study began recruitment in July 2019. Intervention will be up to 12 months, with completion of the project scheduled for September 2020.

Competing interests

Alison Elliott reports a grant from the Medical research Council, UK (POPVAC programme funding). The rest of the authors declare that they have no conflicts of interest.

Author contributions

AME conceived the study. AME, GN, ELW, AN, JN, SC, LZ and JK contributed to study design. LZ, GO, PNK, EN, GK, RA, CN, CO, MN, CZ, SA and FA are site clinicians/nurses/clinical laboratory technicians providing valuable input on clinical considerations of the intervention. MS, SK and RK are field workers handling the organisational integration of the intervention. AN, AM and ELW are involved in organisation of the databases, trial randomization, treatment allocation and drawing up of analytical plans. GN, LZ, JN, AN, SC, ELW and AME drafted the manuscript. All authors reviewed the manuscript, contributed to it and approved the final version.

Acknowledgements

We thank the Uganda National Expanded Programme for Immunisation, Sanofi Pasteur and PaxVax for providing the HPV, yellow fever and oral typhoid vaccines, respectively. The BCG and tetanus-diphtheria vaccines were kind donations from the Serum Institute of India. We thank the Vector Control Division of the Ministry of Health and the Mukono district local government for their support. We also thank members of the POPVAC programme steering committee (chaired by Prof. Richard Hayes) and the Data and Safety Monitoring Board (Dr David Meya, Prof Andrew Prendergast and Dr Elizabeth George).

Funding

The POPVAC programme of work is supported by the Medical Research Council of the United Kingdom (grant number MR/R02118X/1). SC and JN are supported in part by the Makerere University – Uganda Virus Research Institute Centre of Excellence for Infection and Immunity Research and Training (MUII-plus). MUII-plus is funded under the DELTAS Africa Initiative. The DELTAS Africa Initiative is an independent funding scheme of the African Academy of Sciences (AAS),

For peer review only

For peer review only - http://bmjopen.bmj.com/site/about/guidelines.xhtml

Alliance for Accelerating Excellence in Science in Africa (AESA) and supported by the New Partnership for Africa's Development Planning and Coordinating Agency (NEPAD Agency) with funding from the Wellcome Trust (grant 107743) and the UK Government. The MRC/UVRI and LSHTM Uganda Research Unit is jointly funded by the UK Medical Research Council (MRC) and the UK Department for International Development (DFID) under the MRC/DFID Concordat agreement and is also part of the EDCTP2 programme supported by the European Union.

The study sponsor (London School of Hygiene and Tropical Medicine) and funders had no role in study design; collection, management, analysis, and interpretation of data; writing of the protocol; and the decision to submit the protocol for publication.

POPVAC trial team

Principal investigator: Alison Elliott; ***Project leader***: Ludoviko Zirimenya; ***laboratory staff:*** Gyaviira Nkurunungi, Stephen Cose, Rebecca Amongin, Beatrice Nassanga, Jacent Nassuuna, Irene Nambuya, Prossy Kabuubi, Emmanuel Niwagaba, Gloria Oduru, Grace Kabami; ***statisticians and data managers:*** Emily Webb, Agnes Natukunda, Helen Akurut, Alex Mutebe; ***clinicians:*** Anne Wajja, Milly Namutebi, Christopher Zziwa, Joel Serubanja; ***nurses:*** Caroline Onen, Esther Nakazibwe, Josephine Tumusiime, Caroline Ninsiima, Susan Amongi, Florence Akello; ***internal monitor:*** Mirriam Akello; ***field workers:*** Robert Kizindo, Moses Sewankambo, Denis Nsubuga, Samuel Kiwanuka, Fred Kiwudhu; ***boatman:*** David Abiriga; ***administrative management:*** Moses Kizza, Samsi Nansukusa; ***internal and external collaborators:*** Pontiano Kaleebu, Hermelijn Smits, Maria Yazdanbakhsh, Govert van Dam, Paul Corstjens, Sarah Staedke, Henry Luzze, James Kaweesa, Edridah Tukahebwa, Elly Tumushabe, Moses Muwanga

For peer review only

References

1. Taniuchi M, Platts-Mills JA, Begum S, et al. Impact of enterovirus and other enteric pathogens on oral polio and rotavirus vaccine performance in Bangladeshi infants. *Vaccine* 2016;34(27):3068-75. doi: 10.1016/j.vaccine.2016.04.080 [published Online First: 2016/05/08]

2. Fine PE. Variation in protection by BCG: implications of and for heterologous immunity. *Lancet* 1995;346(8986):1339-45.

3. Black GF, Weir RE, Floyd S, et al. BCG-induced increase in interferon-gamma response to mycobacterial antigens and efficacy of BCG vaccination in Malawi and the UK: two randomised controlled studies. *Lancet* 2002;359(9315):1393-401. doi: 10.1016/S0140-6736(02)08353-8

4. Muyanja E, Ssemaganda A, Ngauv P, et al. Immune activation alters cellular and humoral responses to yellow fever 17D vaccine. *The Journal of clinical investigation* 2014;124(7):3147-58. doi: 10.1172/jci75429 [published Online First: 2014/06/10]

5. Barreto ML, Pilger D, Pereira SM, et al. Causes of variation in BCG vaccine efficacy: examining evidence from the BCG REVAC cluster randomized trial to explore the masking and the blocking hypotheses. *Vaccine* 2014;32(30):3759-64. doi: 10.1016/j.vaccine.2014.05.042

6. Tanner R, Kakalacheva K, Miller E, et al. Serum indoleamine 2,3-dioxygenase activity is associated with reduced immunogenicity following vaccination with MVA85A. *BMC infectious diseases* 2014;14:660. doi: 10.1186/s12879-014-0660-7 [published Online First: 2014/12/04]

7. Sissoko MS, Healy SA, Katile A, et al. Safety and efficacy of PfSPZ Vaccine against Plasmodium falciparum via direct venous inoculation in healthy malaria-exposed adults in Mali: a randomised, double-blind phase 1 trial. *The Lancet Infectious diseases* 2017;17(5):498-509. doi: 10.1016/s1473-3099(17)30104-4 [published Online First: 2017/02/22]

8. Venkatraman N, Ndiaye BP, Bowyer G, et al. Safety and Immunogenicity of a Heterologous Prime-Boost Ebola Virus Vaccine Regimen in Healthy Adults in the United Kingdom and Senegal. *The Journal of infectious diseases* 2018;219(8):1187-97. doi: 10.1093/infdis/jiy639

9. Barreto ML, Pereira SM, Pilger D, et al. Evidence of an effect of BCG revaccination on incidence of tuberculosis in school-aged children in Brazil: second report of the BCG-REVAC cluster-randomised trial. *Vaccine* 2011;29(31):4875-7. doi: 10.1016/j.vaccine.2011.05.023

10. van Riet E, Adegnika AA, Retra K, et al. Cellular and humoral responses to influenza in gabonese children living in rural and semi-urban areas. *The Journal of infectious diseases* 2007;196(11):1671-8. doi: 10.1086/522010 [published Online First: 2007/11/17]

11. van Riet E, Retra K, Adegnika AA, et al. Cellular and humoral responses to tetanus vaccination in Gabonese children. *Vaccine* 2008;26(29-30):3690-5. doi: 10.1016/j.vaccine.2008.04.067 [published Online First: 2008/06/10]

12. Venkatraman N, Ndiaye BP, Bowyer G, et al. Safety and Immunogenicity of a Heterologous Prime-Boost Ebola Virus Vaccine Regimen in Healthy Adults in the United Kingdom and Senegal. *The Journal of infectious diseases* 2019;219(8):1187-97. doi: 10.1093/infdis/jiy639

13. Brandt L, Feino Cunha J, Weinreich Olsen A, et al. Failure of the Mycobacterium bovis BCG vaccine: some species of environmental mycobacteria block multiplication of BCG and induction of protective immunity to tuberculosis. *Infection and immunity* 2002;70(2):672-8. [published Online First: 2002/01/18]

14. Flaherty DK, Vesosky B, Beamer GL, et al. Exposure to Mycobacterium avium can modulate established immunity against Mycobacterium tuberculosis infection generated by Mycobacterium bovis BCG vaccination.

For peer review only

For peer review only - http://bmjopen.bmj.com/site/about/guidelines.xhtml

Journal of leukocyte biology 2006;80(6):1262-71. doi: 10.1189/jlb.0606407 [published Online First: 2006/09/14]

15. Maizels RM, McSorley HJ. Regulation of the host immune system by helminth parasites. *The Journal of allergy and clinical immunology* 2016;138(3):666-75. doi: 10.1016/j.jaci.2016.07.007 [published Online First: 2016/08/02]

16. Wammes LJ, Mpairwe H, Elliott AM, et al. Helminth therapy or elimination: epidemiological, immunological, and clinical considerations. *The Lancet Infectious diseases* 2014;14(11):1150-62. doi: 10.1016/S1473-3099(14)70771-6

17. Wammes LJ, Hamid F, Wiria AE, et al. Regulatory T cells in human geohelminth infection suppress immune responses to BCG and Plasmodium falciparum. *Eur J Immunol* 2010;40(2):437-42. doi: 10.1002/eji.200939699

18. Pfeiffer JK, Virgin HW. Viral immunity. Transkingdom control of viral infection and immunity in the mammalian intestine. *Science* 2016;351(6270) doi: 10.1126/science.aad5872

19. Onguru D, Liang Y, Griffith Q, et al. Human schistosomiasis is associated with endotoxemia and Toll-like receptor 2- and 4-bearing B cells. *The American journal of tropical medicine and hygiene* 2011;84(2):321-4. doi: 10.4269/ajtmh.2011.10-0397 [published Online First: 2011/02/05]

20. George PJ, Anuradha R, Kumar NP, et al. Evidence of microbial translocation associated with perturbations in T cell and antigen-presenting cell homeostasis in hookworm infections. *PLoS neglected tropical diseases* 2012;6(10):e1830. doi: 10.1371/journal.pntd.0001830 [published Online First: 2012/10/12]

21. Rajamanickam A, Munisankar S, Bhootra Y, et al. Microbial Translocation Associated with an Acute-Phase Response and Elevations in MMP-1, HO-1, and Proinflammatory Cytokines in Strongyloides stercoralis Infection. *Infection and immunity* 2017;85(1) doi: 10.1128/iai.00772-16 [published Online First: 2016/11/09]

22. Sanya RE, Nkurunungi G, Andia Biraro I, et al. A life without worms. *Transactions of the Royal Society of Tropical Medicine and Hygiene* 2017:1-9. doi: 10.1093/trstmh/trx010 [published Online First: 2017/03/25]

23. Elias D, Britton S, Aseffa A, et al. Poor immunogenicity of BCG in helminth infected population is associated with increased in vitro TGF-beta production. *Vaccine* 2008;26(31):3897-902. doi: S0264-410X(08)00540-9 [pii]
10.1016/j.vaccine.2008.04.083 [published Online First: 2008/06/17]

24. Elias D, Wolday D, Akuffo H, et al. Effect of deworming on human T cell responses to mycobacterial antigens in helminth-exposed individuals before and after bacille Calmette-Guerin (BCG) vaccination. *Clin Exp Immunol* 2001;123(2):219-25.

25. Cooper PJ, Chico ME, Losonsky G, et al. Albendazole treatment of children with ascariasis enhances the vibriocidal antibody response to the live attenuated oral cholera vaccine CVD 103-HgR. *The Journal of infectious diseases* 2000;182(4):1199-206. doi: 10.1086/315837 [published Online First: 2000/09/09]

26. Tweyongyere R, Nassanga BR, Muhwezi A, et al. Effect of Schistosoma mansoni infection and its treatment on antibody responses to measles catch-up immunisation in pre-school children: A randomised trial. *PLoS neglected tropical diseases* 2019;13(2):e0007157. doi: 10.1371/journal.pntd.0007157 [published Online First: 2019/02/15]

27. Chan AW, Tetzlaff JM, Altman DG, et al. SPIRIT 2013 statement: defining standard protocol items for clinical trials. *Ann Intern Med* 2013;158(3):200-7. doi: 10.7326/0003-4819-158-3-201302050-00583 [published Online First: 2013/01/09]

28. Sanya RE, Nkurunungi G, Hoek Spaans R, et al. The Impact of Intensive Versus Standard Anthelminthic Treatment on Allergy-related Outcomes, Helminth Infection Intensity, and Helminth-related Morbidity in Lake Victoria Fishing Communities, Uganda: Results From the LaVIISWA Cluster-randomized Trial. *Clinical*

For peer review only

For peer review only - http://bmjopen.bmj.com/site/about/guidelines.xhtml

infectious diseases : an official publication of the Infectious Diseases Society of America 2019;68(10):1665-74. doi: 10.1093/cid/ciy761 [published Online First: 2018/09/12]

29. WHO. Adolescent Health Research Priorities: Report of a Technical Consultation 2015. http://apps.who.int/iris/bitstream/10665/203564/1/WHO_FWC_MCA_15_07_eng.pdf?ua=1 (accessed 17th May 2019).

30. Webb EL, Nampijja M, Kaweesa J, et al. Helminths are positively associated with atopy and wheeze in Ugandan fishing communities: results from a cross-sectional survey. *Allergy* 2016 doi: 10.1111/all.12867

31. Montresor A, Odermatt P, Muth S, et al. The WHO dose pole for the administration of praziquantel is also accurate in non-African populations. *Transactions of the Royal Society of Tropical Medicine and Hygiene* 2005;99(1):78-81. doi: 10.1016/j.trstmh.2004.06.006

32. Plotkin SA. Correlates of protection induced by vaccination. *Clinical and vaccine immunology : CVI* 2010;17(7):1055-65. doi: 10.1128/cvi.00131-10 [published Online First: 2010/05/14]

33. Riner DK, Ndombi EM, Carter JM, et al. Schistosoma mansoni Infection Can Jeopardize the Duration of Protective Levels of Antibody Responses to Immunizations against Hepatitis B and Tetanus Toxoid. *PLoS neglected tropical diseases* 2016;10(12):e0005180. doi: 10.1371/journal.pntd.0005180

34. Corstjens PL, Nyakundi RK, de Dood CJ, et al. Improved sensitivity of the urine CAA lateral-flow assay for diagnosing active Schistosoma infections by using larger sample volumes. *Parasites & vectors* 2015;8:241. doi: 10.1186/s13071-015-0857-7 [published Online First: 2015/04/22]

35. Fletcher HA, Snowden MA, Landry B, et al. T-cell activation is an immune correlate of risk in BCG vaccinated infants. *Nat Commun* 2016;7:11290. doi: 10.1038/ncomms11290

36. Safaeian M, Porras C, Pan Y, et al. Durable antibody responses following one dose of the bivalent human papillomavirus L1 virus-like particle vaccine in the Costa Rica Vaccine Trial. *Cancer prevention research (Philadelphia, Pa)* 2013;6(11):1242-50. doi: 10.1158/1940-6207.capr-13-0203 [published Online First: 2013/11/06]

37. Harris PA, Taylor R, Minor BL, et al. The REDCap Consortium: Building an International Community of Software Platform Partners. *Journal of biomedical informatics* 2019:103208. doi: 10.1016/j.jbi.2019.103208 [published Online First: 2019/05/13]

38. Harris PA, Taylor R, Thielke R, et al. Research electronic data capture (REDCap)--a metadata-driven methodology and workflow process for providing translational research informatics support. *Journal of biomedical informatics* 2009;42(2):377-81. doi: 10.1016/j.jbi.2008.08.010 [published Online First: 2008/10/22]

39. Blok BA, Arts RJ, van Crevel R, et al. Trained innate immunity as underlying mechanism for the long-term, nonspecific effects of vaccines. *J Leukoc Biol* 2015;98(3):347-56. doi: 10.1189/jlb.5RI0315-096R

40. Watanabe K, Mwinzi PN, Black CL, et al. T regulatory cell levels decrease in people infected with Schistosoma mansoni on effective treatment. *The American journal of tropical medicine and hygiene* 2007;77(4):676-82. [published Online First: 2007/11/06]

41. Schmiedel Y, Mombo-Ngoma G, Labuda LA, et al. CD4+CD25hiFOXP3+ Regulatory T Cells and Cytokine Responses in Human Schistosomiasis before and after Treatment with Praziquantel. *PLoS neglected tropical diseases* 2015;9(8):e0003995. doi: 10.1371/journal.pntd.0003995 [published Online First: 2015/08/21]

42. van den Biggelaar AHJ, Borrmann S, Kremsner P, et al. Immune Responses Induced by Repeated Treatment Do Not Result in Protective Immunity to Schistosoma haematobium: Interleukin (IL)–5 and IL-10 Responses. *The Journal of infectious diseases* 2002;186(10):1474-82. doi: 10.1086/344352

For peer review only

For peer review only - http://bmjopen.bmj.com/site/about/guidelines.xhtml

43. Woolhouse MEJ, Hagan P. Seeking the ghost of worms past. *Nature Medicine* 1999;5(11):1225-27. doi: 10.1038/15169

44. Seledtsov VI, Goncharov AG, Seledtsova GV. Clinically feasible approaches to potentiating cancer cell-based immunotherapies. *Human vaccines & immunotherapeutics* 2015;11(4):851-69. doi: 10.1080/21645515.2015.1009814 [published Online First: 2015/05/02]

FIGURE LEGENDS

Figure 1. Outline of immunisations and anthelminthic intervention

[1]Primary endpoints will be at eight weeks post BCG and four weeks post Yellow fever (YF-17D), oral typhoid (Ty21a), Human Papilloma Virus (HPV) and Tetanus/diptheria (Td) vaccination.

[2]Primary endpoint for responses to Td given at 28 weeks.

For peer review only

For peer review only - http://bmjopen.bmj.com/site/about/guidelines.xhtml

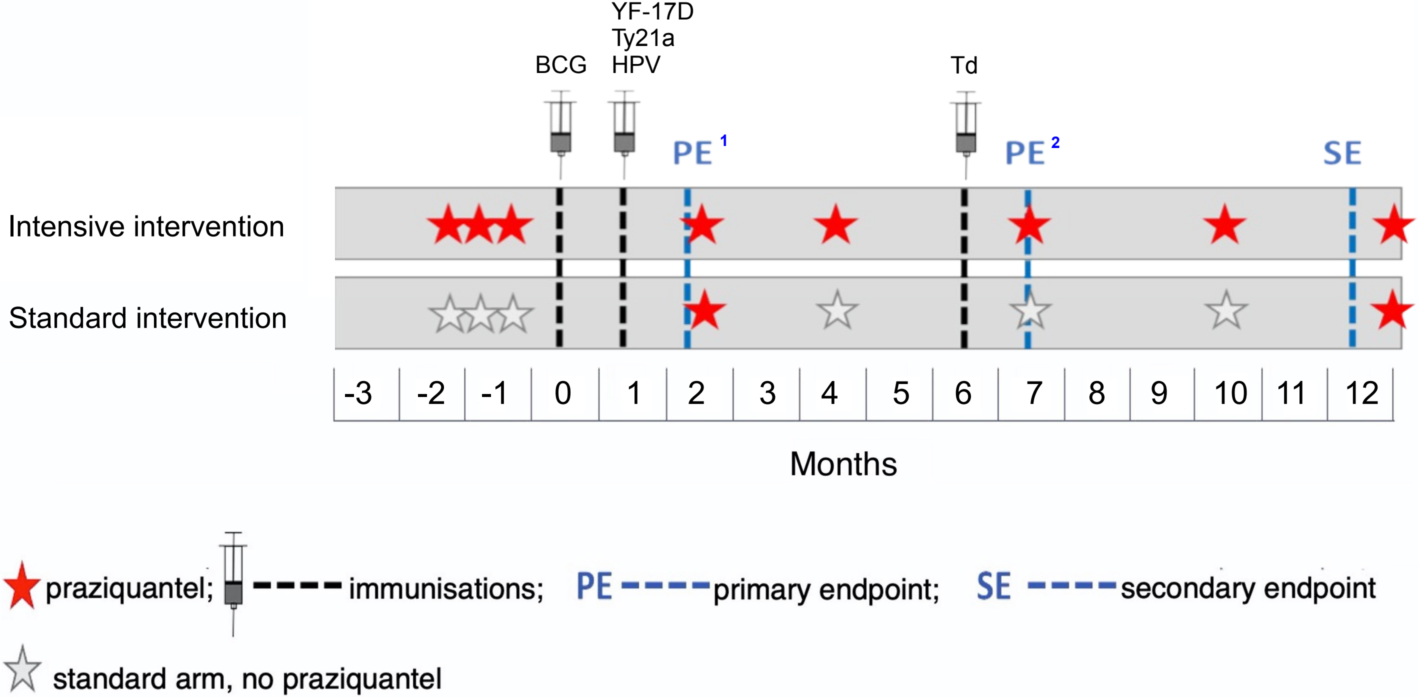

For peer review only - http://bmjopen.bmj.com/site/about/guidelines.xhtml

SUPPLEMENTARY INFORMATION

The effect of intensive treatment for schistosomiasis on immune responses to vaccines among rural Ugandan island adolescents: randomised controlled trial protocol A for the 'POPulation differences in VACcine responses' (POPVAC) programme

Gyaviira Nkurunungi[1,¶,*], Ludoviko Zirimenya[1,¶], Jacent Nassuuna[1,¶], Agnes Natukunda[1,¶], Prossy N Kabuubi[1], Emmanuel Niwagaba[1], Gloria Oduru[1], Grace Kabami[1], Rebecca Amongin[1], Alex Mutebe[1], Milly Namutebi[1], Christopher Zziwa[1], Susan Amongi[1], Caroline Ninsiima[1], Caroline Onen[1], Florence Akello[1], Moses Sewankambo[1], Samuel Kiwanuka[1], Robert Kizindo[1], James Kaweesa[2], Stephen Cose[1,3], Emily L Webb[4], Alison M Elliott[1,3] **for the POPVAC trial team**

[1]Immunomodulation and Vaccines Programme, Medical Research Council/Uganda Virus Research Institute and London School of Hygiene and Tropical Medicine (MRC/UVRI and LSHTM) Uganda Research Unit, Entebbe, Uganda

[2]Vector Control Division, Ministry of Health, Kampala, Uganda

[3]Department of Clinical Research, London School of Hygiene and Tropical Medicine, London, United Kingdom

[4]MRC Tropical Epidemiology Group, Department of Infectious Disease Epidemiology, London School of Hygiene and Tropical Medicine, London, United Kingdom

[¶]These authors contributed equally

***Correspondence:** Gyaviira Nkurunungi; Gyaviira.Nkurunungi@mrcuganda.org

For peer review only

Table S1. Schedule of visits and procedures

VISIT NUMBER	1	2 & 3	4	5[9]	5.2	6	7	8	9	10	11
WEEKS FROM 1ST IMMUNISATION	-8[1]	-6[10], -4, -2	0	4	4 weeks +4 days	8	20	28	32	44	52
	Screening	Treatment (Rx) only	Immunisations	Immunisations		Primary Endpoint (PE), Immunisations	Rx only	Immunisations	PE/Secondary Endpoint (SE)[2]	Rx only	SE, Immunisations
**RANDOMISED INTENSIVE VS STANDARD PRAZIQUANTEL INTERVENTION**											
PZQ intensive arm (x)		x				x[3]	x		x[3]	x	x[3]
PZQ standard arm						x[3]					x[3]
Albendazole						x[3]			x[3]		x[3]
**VACCINES**											
BCG			x								
YF-17D				x							
Ty21a				x							
HPV				x		[x][4]		x			
Td								x			[x][5]
**INVESTIGATIONS/PROCEDURES**											
Inclusion/exclusion criteria	x										
Informed consent	x										
Questionnaire	x		x	x	x	x		x	x		x
Examination	x		(x)	(x)	(x)	(x)		(x)	(x)		(x)
Urine β-HCG test (female only) 1mL	x		x	x				x			
Urine YF viral load					x						
Stool for PCR and storage	x							x			x
Stool for coproantibody and storage	x					x					
**BLOOD SAMPLES**											
Malaria PCR (1ml)			x	x				x			x
Serology for HIV, prior malaria and *S. mansoni* (0.5ml)	x										
Mansonella perstans (1ml)	x										
Serum/plasma CAA (1ml)	x		x	x		x		x	x		x
Hb[8] / Full blood count (0.5ml)	x		x	x				x			
Assessments of pre-immunisation responses, and/or vaccine response outcomes and/or exploratory immunology; storage[7] (10 – 20 mls)			x	x		x		x	x		x
Blood for gene expression (2mls)			x	x				x			
Blood vol (mL)	4		27	17		10-20		27	10		14
Cumulative blood vol (mL)[7]	4		31	48		68		95	105		119

For peer review only - http://bmjopen.bmj.com/site/about/guidelines.xhtml

PE: primary endpoint; **SE:** secondary endpoint; **Rx only**: treatment only
Immunisation days highlighted in green, primary end point days in red, days for treatment only in grey
(**x**) performed if clinically indicated

1. Screening and enrolment into Project A will take place about 8 weeks before immunisation 0 to allow initiation of the praziquantel intervention.
2. Week 32 is primary endpoint for responses to Td given at 28 weeks; secondary endpoint for HPV booster
3. Treatments given after sampling when schedules coincide
4. Week 8 HPV dose will be given for previously-unvaccinated girls aged ≥14 years
5. Week 52 Td booster dose will be provided as a service
6. Exploratory immunology blood volume will be guided by guidelines from Harvard Mass General, where a maximum of 3ml/kg body weight is taken at any one time point and not more than 3ml/kg is taken over any 8-week period (ref http://www.drgreene.com/21_1616.html.) These guidelines have been followed in a previous study vaccinating adolescents with investigational tuberculosis vaccine MVA85A (in Uganda).[1] The total blood volume planned is 68 ml over the initial intensive sampling period of 8 weeks. Revision of sample volumes based on weight will only be required for participants who weigh less than 22 kg; the average weight of children aged 9 years is expected to be 28kg (with 21kg the 3rd centile) with greater weights for older children.[2]
7. At baseline, it will only be Hb estimation by Haemocue
8. Oral typhoid vaccine doses will be administered on three alternate days namely visit 5, 5.1 and 5.2.
9. The first PZQ treatment at week -6 will be administered at the end of the screening visit

22

For peer review only

Further information on recruitment criteria

- Participants who are excluded from the trial because they have been discovered (during screening procedures) to be suffering from a previously undiagnosed condition thought to require further medical attention will be referred appropriately for further investigation and treatment.
- Participants discovered to have severe anaemia will be excluded from the trial and treated for anaemia
- Participants discovered to be HIV-positive will be counselled and offered a CD4 T-cell count and referred to a provider of antiretroviral treatment ("Test and Treat" – i.e. initiation of treatment regardless of CD4 count is recommended for these high-risk communities).
- Participants discovered to be pregnant will be counselled and referred to an antenatal clinic of their choice.

This trial proposes to recruit all participants within a short time-frame. It will not, therefore, be possible to reconsider enrolment of potential participants with temporary exclusion criteria after treatment and resolution of the condition.

Further rationale for the selection of vaccines

Bacillus Calmette–Guérin (BCG)

BCG is a live, replicating parenteral vaccine, and the only licensed vaccine against TB. The BCG vaccine for these studies will be obtained from the Serum Institute of India either directly, or through a supplier in Uganda. The Serum Institute of India provides much of the BCG vaccine used in Uganda.

Worldwide, TB is among the top 10 causes of death; Uganda has an estimated incidence of 202/100,000 people.[3] Infectious, sputum positive, pulmonary TB classically emerges in adolescence, driving the on-going epidemic.[4] Thus adolescent booster immunisation is a key TB control strategy.[5] However, BCG vaccine response and efficacy are often impaired in tropical and rural settings[6-8] and new TB vaccines are similarly affected.[9] In the past, the WHO has been hesitant to recommend BCG re-vaccination. However, in 2017 WHO's Strategic Advisory Group of Experts (SAGE) recommended: "Further research is warranted to explore whether certain sub-groups of age, geographic or *M. tuberculosis* exposure categories would benefit from re-vaccination."[10] Recent results suggest that, despite the variability of BCG efficacy between populations, BCG vaccination in adolescence offers benefit in some tropical settings, especially for individuals who are not yet infected with *Mycobacterium tuberculosis*, and may also be cost-effective.[7 11] Also, BCG vaccine is currently being used among adolescents in South Africa as a comparator in a trial of a novel TB vaccine (trial

For peer review only

registration NCT02075203). BCG efficacy in Ugandan adolescents, and differences in BCG vaccine responses between urban and rural Ugandan populations, have not been tested. Information obtained from this study is expected to further inform the use of BCG in adolescents, and also to inform the development of new vaccines for tuberculosis.

Yellow fever vaccine

Yellow fever vaccine YF-17D is a live replicating parenteral vaccine. The vaccine (Stamaril; Sanofi Pasteur) is available for purchase in Uganda. Yellow Fever (YF) causes outbreaks in Uganda and the wider region[12] and YF-17D is a candidate for Uganda's expanded programme on immunisation (EPI). Lower vaccine replication, lower neutralising antibody induction, and greater waning, are described in Uganda compared to Switzerland.[13] YF-17D is a potential vector for novel vaccine constructs,[14] adding relevance to vaccine development.

Typhoid vaccine Ty21a

Typhoid vaccine Ty21a is a live replicating oral vaccine and also a potential vector for new vaccine constructs.[15] The Ty21a vaccine will be purchased from PaxVax, Redwood City, California. Substantial, multi-year typhoid outbreaks occur in Uganda and immunisation campaigns have been advocated as cost effective.[16] Schistosomiasis has been associated with prolonged *S. typhi* infection[17] and impaired antibody responses to killed typhoid vaccines.[18]

Ty21a was developed in the 1970s. Although not routinely used in Uganda, it has been (and is currently) registered in many countries. It was first registered in the United States and United Kingdom in the 1980s, and is recommended by the WHO for both endemic and epidemic settings.[19] It has comparable efficacy to the parenteral Vi polysaccharide typhoid vaccine, good durability and minimal adverse effects.[19] It is proposed for use in this study to model effects of study exposures and intervention on the response to a live oral vaccine.

The Ty21a vaccine is given as a three-dose regimen on alternate days.

Human Papilloma Virus (HPV) vaccine

The Human Papilloma Virus (HPV) vaccine is a protein virus-like particle. The quadrivalent HPV Vaccine Gardasil (Merck) is available for purchase in Uganda and is the vaccine used by the national EPI programme. Studies after three vaccine doses have found somewhat enhanced responses in the presence of malaria, but no effect of helminths.[20] No study has previously investigated parasite effects on the priming response, but recent results for tetanus suggest that priming may be more susceptible than boosting to adverse effects.[21] This will be important if forthcoming trials support single-dose HPV immunisation (NCT02834637). HPV immunisation is being rolled out among girls to

For peer review only - http://bmjopen.bmj.com/site/about/guidelines.xhtml

prevent cervical neoplasia, the most common cancer among Ugandan women and we will coordinate provision with the national HPV immunisation programme.[22] HPV immunisation is also beneficial for boys since HPV infection is associated with anogenital warts, anal cancer and oropharyngeal cancers in both males and females, and with penile cancer in men,[23] and we will include boys in these studies.

Tetanus and diphtheria vaccines

Tetanus and diphtheria (Td) vaccines comprise inert toxoids. Schistosomiasis is associated with a Th2 biased response to tetanus toxoid[24] and with suppressed antibody responses among those with low pre-immunisation antibody levels.[21] Booster immunisation is recommended for young women to prevent maternal and neonatal tetanus. Recent evidence emphasises the need to protect young men also.[25]

Immunisation Postponement Criteria

If any one of the following is identified at the time scheduled for immunisation, the participant may be immunised at a later date, or withdrawn, at the discretion of the Investigator. The participant must be followed until resolution of the event as with any adverse event:

- Acute disease at the time of immunisation. Acute disease is defined as the presence of a moderate or severe illness with or without fever. All vaccines can be administered to persons with a minor illness such as diarrhoea or mild upper respiratory infection with or without low-grade fever, i.e. temperature of ≤37.5°C (99.5°F)
- Temperature of >37.5°C (99.5°F) at the time of immunisation
- Taking antibiotics or antimalarials currently, or within the past 7 days, of the date of Ty21a administration (ascertained verbally)

Vaccine storage and transport

In order to maintain a reliable vaccine cold chain, the vaccines and diluents to be used will be stored and transported within the recommended temperature range of +2ºC to +8ºC. Care will be taken to ensure that the vaccines are not frozen. BCG, being sensitive to light, will be kept in the dark (normally within its secondary packaging) for as long as possible to protect it during storage and transportation. All vaccines will be kept in appropriate refrigeration equipment with a temperature monitoring device to ensure temperatures remain between +2ºC and +8ºC. Cold boxes/vaccines carriers with temperature monitors will be used to transport vaccines and the diluents from the MRC/UVRI and LSHTM Uganda Research Unit (Entebbe) to Koome island and while transporting vaccines to immunisation sessions. Designated staff will be given responsibility for managing the

For peer review only

For peer review only - http://bmjopen.bmj.com/site/about/guidelines.xhtml

vaccine cold chain. All cold chain equipment including the temperature monitoring devices used for this project will comply with relevant technical specifications as defined by the EPI standards. Basic routine maintenance will be regularly carried out on all cold chain equipment.

Additional laboratory measurements

Additional assays will comprise HIV serology, pregnancy testing and full blood counts. HIV testing and pregnancy testing will be accompanied by appropriate counselling by trained staff.

- HIV serology will be done on blood samples using rapid tests and according to prevailing national algorithms.[26] This will be done at baseline.
- Pregnancy testing will be done using urine samples and standard operating procedures for assessment of urine β-human chorionic gonadotropin (βhCG). This will be done at baseline and before immunisation on each immunisation day.
- Full blood counts will be conducted using a haematology analyser. Mild, moderate and severe anaemia will be defined according to WHO guidelines, by age.[27] This will be done at baseline to test for anaemia as part of the eligibility assessment, and pre-immunisation as part of the assessment of immunological profile.

Individuals found to be HIV positive or pregnant will be referred to appropriate providers for further care. Individuals with severe anaemia (haemoglobin <82g/L) will be excluded from the randomised intervention (since the intervention might be beneficial in management of anaemia). They will be treated for anaemia.

Sample handling and archive

Blood and other samples will be processed according to local laboratory standard operating procedures (SOPs). All samples will reach the laboratory in anonymised form.

A sample archive will be developed. Although our current programme of work will address specific hypotheses regarding pathways of effects of parasites and interventions, the sample archive will provide a major asset for exploration of new leads arising from this work, or for an alternative, "systems biology" approach employing (for example) proteomic, genomic, epigenetic and transcriptomic analyses, and investigating the microbiome and virome. Information provided to participants, and consent forms, will include considerations of sample storage, and the possibility of sample analysis in laboratories within and outside Uganda. Participants will be able to decide if they will permit such future use of any leftover samples. We plan to store the samples for up to 20 years. If further storage is needed after that time, permission will be requested from the Uganda Virus Research Institute and London School of Hygiene and Tropical Medicine ethical review committees.

For peer review only

If they elect not to permit this, all of those leftover samples will be discarded after the completion of the work included in the current protocol.

Operational considerations

Programme governance

A Programme Steering Committee has been set up to guide progress across all projects. This comprises the following:

- An independent chair
- Representatives from the Ministry of Health programmes for immunisation and for vector borne disease control
- Representatives of district authorities (Mukono and Jinja districts)
- Community representatives
- Principal investigator and co-investigators
- Project leader and post-doctoral immunologist
- Trial statistician
- Laboratory manager
- Medical Research Council observer

Informed consent

Both written informed assent from the participants and written informed consent from a parent or guardian will be required for participation, although these may not necessarily be obtained at the same time. Information will be provided in both English and the appropriate local language. For individuals who cannot speak the languages used, or who cannot read or write, a witness who can read the information sheet and translate the information to the participant or parent/guardian will be used. Two different types of age specific assent forms will be used for the group of participants aged 9 – 12 years and for the group aged 13 – 17 years. Informed consent by emancipated or mature minors will be obtained using a designated consent form for these categories of participants.

The aims of the study, all tests, treatments and immunisations to be carried out and potential risks will be explained. The participant will be given the opportunity to ask about details of the trial, and will then have time to consider whether or not to participate. If they do decide to participate, they and their parent/guardian will sign and date two copies of the assent and consent forms, one for them to take away and keep, and one to be stored securely by the research team. Separate information and consent forms will be provided (i) for consent for storage of samples for future

For peer review only

For peer review only - http://bmjopen.bmj.com/site/about/guidelines.xhtml

studies and for anonymous sharing of data from this study and (ii) for possible genetic studies; the information sheet will explain that these data may be used in analyses related to this protocol.

Screening and Eligibility Assessment

Once the informed consent process has been completed, and consent (and assent) given, a baseline medical history (including concomitant medication) will be collected. Vital signs will be checked and a physical examination will be performed. Inclusion and exclusion criteria will be checked.

Participants will undergo pre- and post-test counselling for HIV and (for girls) pregnancy testing by a trained and experienced nurse- or clinician-counsellor. Blood, urine and stool samples will be obtained, for tests as specified in the schedule of procedures. These tests are to exclude the major, immunomodulating co-infection, HIV, and conditions that might impact safety (anaemia, pregnancy).

Enrolment

Participants who consent/assent, complete the screening processes, satisfy all the inclusion criteria and meet none of the exclusion criteria will be enrolled.

Discontinuation/withdrawal criteria

In accordance with the principles of the current revision of the Declaration of Helsinki and any other applicable regulations, a participant has the right to withdraw from the study at any time and for any reason, and is not obliged to give his or her reasons for doing so. The Investigator may withdraw the participant at any time in the interests of the participant's health and well-being. In addition, the participant may withdraw/be withdrawn for any of the following reasons:

- Ineligibility (either arising during the study or retrospectively, having been overlooked at screening)
- Administrative decision by the Investigator
- Significant protocol deviation
- Participant non-compliance with study requirements
- An adverse event which requires discontinuation of the study involvement or results in inability to continue to comply with study procedures.

Any participant who becomes pregnant during the trial will be followed up until the end of the pregnancy but no further immunisations will be given unless indicated during pregnancy (as is the case for tetanus toxoid). The trial allocation for this participant will be unblinded and the participant will only be given further treatment if clinically indicated. The babies will also be followed up and

For peer review only

214 examined for any adverse effects. We will not routinely perform venepuncture in a pregnant
215 participant.

216 The reason for withdrawal will be recorded in the case report form (CRF). If withdrawal is due to an
217 AE, appropriate follow-up visits or medical care will be arranged, with the agreement of the
218 participant, until the AE has resolved, stabilised or a non-trial related causality has been assigned.

219 If a participant withdraws from the study samples collected before their withdrawal from the trial
220 will be used/ stored unless the participant specifically requests otherwise.

221 *Trial discontinuation*

222 The trial will be discontinued in the event of new scientific information that renders continuation
223 futile or unethical, or for any other reason, at the discretion of the Programme Steering Committee.

224 *End of study definition*

225 The trial will be completed when the last participant enrolled into the trial has completed their final
226 follow up visit.

227 *Safety assessments and oversight*

228 No new investigational drug or product will be used in the proposed trial. However, standard
229 approaches for monitoring safety and reporting of serious adverse events will be followed.

230 *Monitoring*

231 The trial will be monitored by both internal and external monitors according to a pre-defined
232 monitoring plan which will include a site initiation visit, monitoring visits at least annually, and a
233 close-out visit. The monitors will assess patient safety, data integrity, and adherence to the protocol
234 and to Good Clinical Research Practice procedures.

235 ***Considerations regarding standard of care***

236 *S. mansoni* infection status will be determined retrospectively through assays conducted in bulk on
237 stored samples (plasma CAA). These results will not, therefore, be useful to determine management
238 of individual participants.

239 Participants in the standard treatment arm will receive lower levels of anthelminthic treatment.
240 However, all trial arms will receive a minimum of well-implemented national standard of care.

241 Standard of care will comprise annual praziquantel treatment. Our own results from the Lake
242 Victoria Island Intervention Study on Worms and Allergy-related diseases (LaVIISWA),[28] which
243 compared annual versus quarterly intervention for schistosomiasis at community level over three

For peer review only

years, showed no advantage of quarterly treatment for morbidity outcomes attributed to schistosomiasis.

Schistosomiasis can cause anaemia. To manage the expected differential benefits of the interventions for anaemia, a full blood count will be performed at baseline, as discussed above; anaemic children will be managed appropriately and severely anaemic children excluded.

Albendazole will be provided twice a year to manage nematode infections (after collection of primary and secondary endpoint samples).

Procedures to be followed in the event of abnormal findings

Abnormal clinical findings from medical history, examination or blood tests will be assessed as to their clinical significance throughout the trials. If an abnormal test result is deemed clinically significant, it may be repeated. If a test remains clinically significant, the participant will be informed and appropriate medical care arranged as appropriate and with the permission of the participant. Specific details regarding findings, discussion with participants and resulting actions will be recorded in the clinical records. Decisions to exclude the participant from enrolling in the trial or to withdraw a participant from the trial will be at the discretion of the Investigator.

Data and Safety Monitoring Board (DSMB)

A data and safety monitoring board (DSMB) has been appointed to provide real-time safety oversight. The DSMB will be notified within 7 days of the Investigators' being aware of the occurrence of SAEs. The DSMB may recommend the Investigators to place the trial on hold if deemed necessary following an intervention-related SAE. The DSMB will be chaired by a clinician experienced in clinical trials. There will be a minimum of two other appropriately qualified committee members. In the case of events related to a blinded intervention, the DSMB can request unblinding. Membership will include a statistician, and at least one Ugandan member. All correspondence between Investigators and the DSMB will be conveyed by the Principal Investigator to the trial Sponsor. The Chair of the DSMB will be contacted for advice and independent review by the Investigator or trial Sponsor in the following situations:

- The occurrence of any SAE
- Any other situation where the Investigator or trial Sponsor feels independent advice or review is important

Ethical and regulatory considerations

Further information regarding risks

For peer review only - http://bmjopen.bmj.com/site/about/guidelines.xhtml

The immunisations to be given have recognised side effects which are usually mild and resolve spontaneously in a few days to one week. Parenteral vaccines are likely to result in pain and swelling at the site of injection and mild fever; very occasionally pain and swelling can be severe and associated with difficulty in moving the shoulder. Sometimes headache and tiredness occur. Rarely a vaccine may cause a severe allergic reaction. For most vaccines this is estimated at less than one in a million doses (but 1 in 55,000 for Yellow Fever vaccine).[29] Individuals with a history of a possible allergic reaction to drugs or vaccines, or to vaccine components including eggs or chicken proteins, will be excluded from the studies. The research team will be trained and prepared to manage severe allergic reactions.

Adverse reactions to Yellow Fever vaccine include severe nervous system reaction (about 1 person in 125,000) and severe, life-threatening illness with organ failure (about 1 person in 250,000). The mortality for this severe, life-threatening adverse effect is reported as about 50%.[29]

BCG immunisation is likely to induce a scar in many cases. This may develop over several weeks, starting as a small papule at the injection site which may become ulcerated and then heal over a period of 2 to 5 months; and lymphadenopathy may develop. Occasionally a more severe local reaction occurs (estimated at 1 per 1,000-10,000 doses): for example, an abscess develops and scars may develop into keloids. Rarely BCG can cause disseminated disease (1 per 230,000 to 640,000 doses), or disease in sites remote from the immunisation site. Disseminated BCG disease usually occurs in immunocompromised people: HIV positive people will be excluded from these studies.[30] BCG "pre-immunisation" may interfere with the response to the subsequent live vaccines; indeed our hypothesis, and published results, suggest that it may suppress replication of YF 17D vaccine.[31] However, this reduced replication has not been shown to correlate with, or result in, reduced levels of neutralising antibody titres (which are the desired protective outcome).[13 31]

Oral typhoid vaccine (Ty21a) may occasionally be associated with stomach pain, nausea, vomiting and (rarely) rash.[29]

Praziquantel has been in use for about 30 years. It has a well-recognised profile of side effects including dizziness, nausea, vomiting, abdominal pain, diarrhoea (sometimes with blood) and urticarial rash. The symptoms are considered to arise largely from the effects of killing worms and to be more severe in people with heavy infections. Symptoms are better tolerated when the drugs are given after food and we will provide treatment after a meal or snack. Simple medications, such as paracetamol and cetirizine, can alleviate symptoms and these will be available on treatment days.

For peer review only - http://bmjopen.bmj.com/site/about/guidelines.xhtml

References

1. Wajja A, Kizito D, Nassanga B, et al. The effect of current Schistosoma mansoni infection on the immunogenicity of a candidate TB vaccine, MVA85A, in BCG-vaccinated adolescents: An open-label trial. *PLoS neglected tropical diseases* 2017;11(5):e0005440. doi: 10.1371/journal.pntd.0005440 [published Online First: 2017/05/05]

2. WHO. Growth reference 5-19 years. 2007

3. WHO. Global tuberculosis report 2016 http://www.who.int/tb/publications/global_report/en/ (accessed 17 June 2017), 2016.

4. Alcais A, Fieschi C, Abel L, et al. Tuberculosis in children and adults: two distinct genetic diseases. *J Exp Med* 2005;202(12):1617-21. doi: 10.1084/jem.20052302

5. Weiner J, 3rd, Kaufmann SH. Recent advances towards tuberculosis control: vaccines and biomarkers. *J Intern Med* 2014;275(5):467-80. doi: 10.1111/joim.12212

6. Fine PE. Variation in protection by BCG: implications of and for heterologous immunity. *Lancet (London, England)* 1995;346(8986):1339-45.

7. Barreto ML, Pilger D, Pereira SM, et al. Causes of variation in BCG vaccine efficacy: examining evidence from the BCG REVAC cluster randomized trial to explore the masking and the blocking hypotheses. *Vaccine* 2014;32(30):3759-64. doi: 10.1016/j.vaccine.2014.05.042

8. Black GF, Weir RE, Floyd S, et al. BCG-induced increase in interferon-gamma response to mycobacterial antigens and efficacy of BCG vaccination in Malawi and the UK: two randomised controlled studies. *Lancet (London, England)* 2002;359(9315):1393-401. doi: 10.1016/S0140-6736(02)08353-8

9. Tanner R, Kakalacheva K, Miller E, et al. Serum indoleamine 2,3-dioxygenase activity is associated with reduced immunogenicity following vaccination with MVA85A. *BMC infectious diseases* 2014;14:660. doi: 10.1186/s12879-014-0660-7 [published Online First: 2014/12/04]

10. WHO. SAGE Evidence to recommendations framework. 2017. http://www.who.int/immunization/sage/meetings/2017/october/2_EvidencetoRecommendationFramework_BCG.pdf (accessed 16th March 2018).

11. Dye C. Making wider use of the world's most widely used vaccine: Bacille Calmette-Guerin revaccination reconsidered. *Journal of the Royal Society, Interface* 2013;10(87):20130365. doi: 10.1098/rsif.2013.0365 [published Online First: 2013/08/02]

12. Kraemer MU, Faria NR, Reiner RC, Jr., et al. Spread of yellow fever virus outbreak in Angola and the Democratic Republic of the Congo 2015-16: a modelling study. *The Lancet Infectious diseases* 2016 doi: 10.1016/s1473-3099(16)30513-8 [published Online First: 2016/12/27]

13. Muyanja E, Ssemaganda A, Ngauv P, et al. Immune activation alters cellular and humoral responses to yellow fever 17D vaccine. *The Journal of clinical investigation* 2014;124(7):3147-58. doi: 10.1172/jci75429 [published Online First: 2014/06/10]

14. Aguiar M, Stollenwerk N, Halstead SB. The Impact of the Newly Licensed Dengue Vaccine in Endemic Countries. *PLoS neglected tropical diseases* 2016;10(12):e0005179. doi: 10.1371/journal.pntd.0005179 [published Online First: 2016/12/22]

15. Dharmasena MN, Osorio M, Filipova S, et al. Stable expression of Shigella dysenteriae serotype 1 O-antigen genes integrated into the chromosome of live Salmonella oral vaccine vector Ty21a. *Pathogens and disease* 2016 doi: 10.1093/femspd/ftw098 [published Online First: 2016/09/23]

For peer review only - http://bmjopen.bmj.com/site/about/guidelines.xhtml

16. Carias C, Walters MS, Wefula E, et al. Economic evaluation of typhoid vaccination in a prolonged typhoid outbreak setting: the case of Kasese district in Uganda. *Vaccine* 2015;33(17):2079-85. doi: 10.1016/j.vaccine.2015.02.027 [published Online First: 2015/02/26]

17. Melhem RF, LoVerde PT. Mechanism of interaction of Salmonella and Schistosoma species. *Infection and immunity* 1984;44(2):274-81. [published Online First: 1984/05/01]

18. Muniz-Junqueira MI, Tavares-Neto J, Prata A, et al. Antibody response to Salmonella typhi in human schistosomiasis mansoni. *Revista da Sociedade Brasileira de Medicina Tropical* 1996;29(5):441-5. [published Online First: 1996/09/01]

19. WHO. Position Paper on Typhoid vaccines: WHO position paper – March 2018 2018

20. Brown J, Baisley K, Kavishe B, et al. Impact of malaria and helminth infections on immunogenicity of the human papillomavirus-16/18 AS04-adjuvanted vaccine in Tanzania. *Vaccine* 2014;32(5):611-7. doi: 10.1016/j.vaccine.2013.11.061

21. Riner DK, Ndombi EM, Carter JM, et al. Schistosoma mansoni Infection Can Jeopardize the Duration of Protective Levels of Antibody Responses to Immunizations against Hepatitis B and Tetanus Toxoid. *PLoS neglected tropical diseases* 2016;10(12):e0005180. doi: 10.1371/journal.pntd.0005180

22. Centre HI. HPV and related diseases report: Uganda. 2016. http://www.hpvcentre.net/statistics/reports/UGA.pdf (accessed 20.01.2017).

23. WHO. Human papillomavirus vaccines: WHO position paper, May 2017. *Releve epidemiologique hebdomadaire* 2017;92(19):241-68. [published Online First: 2017/05/23]

24. Sabin EA, Araujo MI, Carvalho EM, et al. Impairment of tetanus toxoid-specific Th1-like immune responses in humans infected with Schistosoma mansoni. *The Journal of infectious diseases* 1996;173(1):269-72. [published Online First: 1996/01/01]

25. Nanteza B, Galukande M, Aceng J, et al. The burden of tetanus in Uganda. *SpringerPlus* 2016;5(1):705. doi: 10.1186/s40064-016-2309-z [published Online First: 2016/06/29]

26. Ministry of Health–Uganda. Uganda Clinical Guidelines 2016. http://apps.who.int/medicinedocs/documents/s23532en/s23532en.pdf

27. WHO. Haemoglobin concentrations for the diagnosis of anaemia and assessment of severity. Vitamin and Mineral Nutrition Information System. Geneva, World Health Organization, 2011 (WHO/NMH/NHD/MNM/11.1).

28. Sanya RE, Nkurunungi G, Hoek Spaans R, et al. The Impact of Intensive Versus Standard Anthelminthic Treatment on Allergy-related Outcomes, Helminth Infection Intensity, and Helminth-related Morbidity in Lake Victoria Fishing Communities, Uganda: Results From the LaVIISWA Cluster-randomized Trial. *Clinical Infectious Diseases* 2018:ciy761-ciy61. doi: 10.1093/cid/ciy761

29. CDC. Centers for Disease Control and Prevention, vaccines and immunizations.

30. WHO. Information sheet observed rate of vaccine reactions Bacille Calmette-Guérin (BCG) vaccine. 2012

31. Arts RJW, Moorlag S, Novakovic B, et al. BCG Vaccination Protects against Experimental Viral Infection in Humans through the Induction of Cytokines Associated with Trained Immunity. *Cell host & microbe* 2018;23(1):89-100.e5. doi: 10.1016/j.chom.2017.12.010 [published Online First: 2018/01/13]

For peer review only

For peer review only - http://bmjopen.bmj.com/site/about/guidelines.xhtml

Reporting checklist for protocol of a clinical trial.

Based on the SPIRIT guidelines.

Instructions to authors

Complete this checklist by entering the page numbers from your manuscript where readers will find each of the items listed below.

Your article may not currently address all the items on the checklist. Please modify your text to include the missing information. If you are certain that an item does not apply, please write "n/a" and provide a short explanation.

Upload your completed checklist as an extra file when you submit to a journal.

In your methods section, say that you used the SPIRITreporting guidelines, and cite them as:

Chan A-W, Tetzlaff JM, Altman DG, Laupacis A, Gøtzsche PC, Krleža-Jerić K, Hróbjartsson A, Mann H, Dickersin K, Berlin J, Doré C, Parulekar W, Summerskill W, Groves T, Schulz K, Sox H, Rockhold FW, Rennie D, Moher D. SPIRIT 2013 Statement: Defining standard protocol items for clinical trials. Ann Intern Med. 2013;158(3):200-207

		Reporting Item	Page Number
**Administrative information**			
Title	#1	Descriptive title identifying the study design, population, interventions, and, if applicable, trial acronym	1
Trial registration	#2a	Trial identifier and registry name. If not yet registered, name of intended registry	2
Trial registration: data set	#2b	All items from the World Health Organization Trial Registration Data Set	n/a
Protocol version	#3	Date and version identifier	Information available at ISRCTN60517191
Funding	#4	Sources and types of financial, material, and other support	15
Roles and	#5a	Names, affiliations, and roles of protocol	15

For peer review only

responsibilities: contributorship		contributors	
Roles and responsibilities: sponsor contact information	#5b	Name and contact information for the trial sponsor	Information available at ISRCTN60517191
Roles and responsibilities: sponsor and funder	#5c	Role of study sponsor and funders, if any, in study design; collection, management, analysis, and interpretation of data; writing of the report; and the decision to submit the report for publication, including whether they will have ultimate authority over any of these activities	16
Roles and responsibilities: committees	#5d	Composition, roles, and responsibilities of the coordinating centre, steering committee, endpoint adjudication committee, data management team, and other individuals or groups overseeing the trial, if applicable (see Item 21a for data monitoring committee)	Information detailed in supplementary information file
**Introduction**			
Background and rationale	#6a	Description of research question and justification for undertaking the trial, including summary of relevant studies (published and unpublished) examining benefits and harms for each intervention	4
Background and rationale: choice of comparators	#6b	Explanation for choice of comparators	8
Objectives	#7	Specific objectives or hypotheses	6
Trial design	#8	Description of trial design including type of trial (eg, parallel group, crossover, factorial, single group), allocation ratio, and framework (eg, superiority, equivalence, non-inferiority, exploratory)	7
**Methods: Participants, interventions, and outcomes**			

For peer review only

Study setting	#9	Description of study settings (eg, community clinic, academic hospital) and list of countries where data will be collected. Reference to where list of study sites can be obtained	7
Eligibility criteria	#10	Inclusion and exclusion criteria for participants. If applicable, eligibility criteria for study centres and individuals who will perform the interventions (eg, surgeons, psychotherapists)	7
Interventions: description	#11a	Interventions for each group with sufficient detail to allow replication, including how and when they will be administered	8
Interventions: modifications	#11b	Criteria for discontinuing or modifying allocated interventions for a given trial participant (eg, drug dose change in response to harms, participant request, or improving / worsening disease)	Detailed in supplementary information file
Interventions: adherance	#11c	Strategies to improve adherence to intervention protocols, and any procedures for monitoring adherence (eg, drug tablet return; laboratory tests)	Detailed in supplementary information file
Interventions: concomitant care	#11d	Relevant concomitant care and interventions that are permitted or prohibited during the trial	Detailed in supplementary information file
Outcomes	#12	Primary, secondary, and other outcomes, including the specific measurement variable (eg, systolic blood pressure), analysis metric (eg, change from baseline, final value, time to event), method of aggregation (eg, median, proportion), and time point for each outcome. Explanation of the clinical relevance of chosen efficacy and harm outcomes is strongly recommended	10
Participant timeline	#13	Time schedule of enrolment, interventions (including any run-ins and washouts), assessments, and visits for participants. A schematic diagram is highly recommended (see Figure)	14<br>Also detailed in supplementary information file, Table S1

For peer review only

Sample size	#14	Estimated number of participants needed to achieve study objectives and how it was determined, including clinical and statistical assumptions supporting any sample size calculations	11
Recruitment	#15	Strategies for achieving adequate participant enrolment to reach target sample size	Detailed in supplementary information file
**Methods: Assignment of interventions (for controlled trials)**			
Allocation: sequence generation	#16a	Method of generating the allocation sequence (eg, computer-generated random numbers), and list of any factors for stratification. To reduce predictability of a random sequence, details of any planned restriction (eg, blocking) should be provided in a separate document that is unavailable to those who enrol participants or assign interventions	8
Allocation concealment mechanism	#16b	Mechanism of implementing the allocation sequence (eg, central telephone; sequentially numbered, opaque, sealed envelopes), describing any steps to conceal the sequence until interventions are assigned	9
Allocation: implementation	#16c	Who will generate the allocation sequence, who will enrol participants, and who will assign participants to interventions	8, 9
Blinding (masking)	#17a	Who will be blinded after assignment to interventions (eg, trial participants, care providers, outcome assessors, data analysts), and how	9
Blinding (masking): emergency unblinding	#17b	If blinded, circumstances under which unblinding is permissible, and procedure for revealing a participant's allocated intervention during the trial	Detailed in supplementary information file
**Methods: Data collection, management, and analysis**			

For peer review only

Data collection plan	#18a	Plans for assessment and collection of outcome, baseline, and other trial data, including any related processes to promote data quality (eg, duplicate measurements, training of assessors) and a description of study instruments (eg, questionnaires, laboratory tests) along with their reliability and validity, if known. Reference to where data collection forms can be found, if not in the protocol	10. More details in supplementary information file
Data collection plan: retention	#18b	Plans to promote participant retention and complete follow-up, including list of any outcome data to be collected for participants who discontinue or deviate from intervention protocols	Detailed in supplementary information file
Data management	#19	Plans for data entry, coding, security, and storage, including any related processes to promote data quality (eg, double data entry; range checks for data values). Reference to where details of data management procedures can be found, if not in the protocol	13. More information in the statistical analysis plan found at ISRCTN60517191
Statistics: outcomes	#20a	Statistical methods for analysing primary and secondary outcomes. Reference to where other details of the statistical analysis plan can be found, if not in the protocol	13. More information in the statistical analysis plan found at ISRCTN60517191
Statistics: additional analyses	#20b	Methods for any additional analyses (eg, subgroup and adjusted analyses)	11. More information in the statistical analysis plan found at ISRCTN60517191
Statistics: analysis population and missing data	#20c	Definition of analysis population relating to protocol non-adherence (eg, as randomised analysis), and any statistical methods to handle missing data (eg, multiple imputation)	Information in the statistical analysis plan found at ISRCTN60517191
**Methods: Monitoring**			

For peer review only

Data monitoring: formal committee	#21a	Composition of data monitoring committee (DMC); summary of its role and reporting structure; statement of whether it is independent from the sponsor and competing interests; and reference to where further details about its charter can be found, if not in the protocol. Alternatively, an explanation of why a DMC is not needed	Detailed in supplementary information file
Data monitoring: interim analysis	#21b	Description of any interim analyses and stopping guidelines, including who will have access to these interim results and make the final decision to terminate the trial	Detailed in supplementary information file
Harms	#22	Plans for collecting, assessing, reporting, and managing solicited and spontaneously reported adverse events and other unintended effects of trial interventions or trial conduct	Detailed in supplementary information file
Auditing	#23	Frequency and procedures for auditing trial conduct, if any, and whether the process will be independent from investigators and the sponsor	Detailed in supplementary information file
**Ethics and dissemination**			
Research ethics approval	#24	Plans for seeking research ethics committee / institutional review board (REC / IRB) approval	12
Protocol amendments	#25	Plans for communicating important protocol modifications (eg, changes to eligibility criteria, outcomes, analyses) to relevant parties (eg, investigators, REC / IRBs, trial participants, trial registries, journals, regulators)	11
Consent or assent	#26a	Who will obtain informed consent or assent from potential trial participants or authorised surrogates, and how (see Item 32)	12.<br>Also detailed in supplementary information file
Consent or assent: ancillary studies	#26b	Additional consent provisions for collection and use of participant data and biological specimens in ancillary studies, if applicable	Detailed in supplementary information file

For peer review only

Confidentiality	#27	How personal information about potential and enrolled participants will be collected, shared, and maintained in order to protect confidentiality before, during, and after the trial	Detailed in supplementary information file
Declaration of interests	#28	Financial and other competing interests for principal investigators for the overall trial and each study site	15
Data access	#29	Statement of who will have access to the final trial dataset, and disclosure of contractual agreements that limit such access for investigators	n/a
Ancillary and post trial care	#30	Provisions, if any, for ancillary and post-trial care, and for compensation to those who suffer harm from trial participation	Detailed in supplementary information file
Dissemination policy: trial results	#31a	Plans for investigators and sponsor to communicate trial results to participants, healthcare professionals, the public, and other relevant groups (eg, via publication, reporting in results databases, or other data sharing arrangements), including any publication restrictions	2, 13
Dissemination policy: authorship	#31b	Authorship eligibility guidelines and any intended use of professional writers	n/a
Dissemination policy: reproducible research	#31c	Plans, if any, for granting public access to the full protocol, participant-level dataset, and statistical code	13
**Appendices**			
Informed consent materials	#32	Model consent form and other related documentation given to participants and authorised surrogates	Supplementary files provided
Biological specimens	#33	Plans for collection, laboratory evaluation, and storage of biological specimens for genetic or molecular analysis in the current trial and for future use in ancillary studies, if applicable	n/a

None The SPIRIT checklist is distributed under the terms of the Creative Commons Attribution License CC-BY-ND 3.0. This checklist can be completed online using https://www.goodreports.org/, a tool made by the EQUATOR Network in collaboration with Penelope.ai

For peer review only

BMJ Open

The effect of intensive treatment for schistosomiasis on immune responses to vaccines among rural Ugandan island adolescents: randomised controlled trial protocol A for the 'POPulation differences in VACcine responses' (POPVAC) programme

Journal:	*BMJ Open*
Manuscript ID	bmjopen-2020-040426.R1
Article Type:	Protocol
Date Submitted by the Author:	05-Oct-2020
Complete List of Authors:	Nkurunungi, Gyaviira; MRC/UVRI and LSHTM Uganda Research Unit, Immunomodulation and Vaccines Programme<br>Zirimenya , Ludoviko ; MRC/UVRI and LSHTM Uganda Research Unit, Immunomodulation and Vaccines Programme<br>Nassuuna , Jacent; MRC/UVRI and LSHTM Uganda Research Unit, Immunomodulation and Vaccines Programme<br>Natukunda , Agnes ; MRC/UVRI and LSHTM Uganda Research Unit, Immunomodulation and Vaccines Programme<br>Kabuubi , Prossy ; MRC/UVRI and LSHTM Uganda Research Unit, Immunomodulation and Vaccines Programme<br>Niwagaba , Emmanuel ; MRC/UVRI and LSHTM Uganda Research Unit, Immunomodulation and Vaccines Programme<br>Oduru, Gloria; MRC/UVRI and LSHTM Uganda Research Unit, Immunomodulation and Vaccines Programme<br>Kabami , Grace ; MRC/UVRI and LSHTM Uganda Research Unit, Immunomodulation and Vaccines Programme<br>Amongin , Rebecca ; MRC/UVRI and LSHTM Uganda Research Unit, Immunomodulation and Vaccines Programme<br>Mutebe , Alex ; MRC/UVRI and LSHTM Uganda Research Unit, Immunomodulation and Vaccines Programme<br>Namutebi , Milly ; MRC/UVRI and LSHTM Uganda Research Unit, Immunomodulation and Vaccines Programme<br>Zziwa , Christopher ; MRC/UVRI and LSHTM Uganda Research Unit, Immunomodulation and Vaccines Programme<br>Amongi, Susan; MRC/UVRI and LSHTM Uganda Research Unit, Immunomodulation and Vaccines Programme<br>Ninsiima , Caroline ; MRC/UVRI and LSHTM Uganda Research Unit, Immunomodulation and Vaccines Programme<br>Onen, Caroline; MRC/UVRI and LSHTM Uganda Research Unit, Immunomodulation and Vaccines Programme<br>Akello , Florence ; MRC/UVRI and LSHTM Uganda Research Unit, Immunomodulation and Vaccines Programme<br>Sewankambo, Moses; MRC/UVRI and LSHTM Uganda Research Unit, Immunomodulation and Vaccines Programme<br>Kiwanuka, Samuel; MRC/UVRI and LSHTM Uganda Research Unit, Immunomodulation and Vaccines Programme<br>Kizindo , Robert ; MRC/UVRI and LSHTM Uganda Research Unit,

	Immunomodulation and Vaccines Programme<br>Kaweesa, James; Republic of Uganda Ministry of Health, Vector Control Division<br>Cose , Stephen ; MRC/UVRI and LSHTM Uganda Research Unit, Immunomodulation and Vaccines Programme; London School of Hygiene and Tropical Medicine Department of Clinical Research<br>Webb, Emily; London School of Hygiene & Tropical Medicine, MRC Tropical Epidemiology Group, Department of Infectious Disease Epidemiology<br>Elliott, Alison ; MRC/UVRI and LSHTM Uganda Research Unit, Immunomodulation and Vaccines Programme; London School of Hygiene and Tropical Medicine Department of Clinical Research
**Primary Subject Heading**:	Infectious diseases
Secondary Subject Heading:	Epidemiology, Immunology (including allergy), Public health
Keywords:	Infection control < INFECTIOUS DISEASES, PARASITOLOGY, Public health < INFECTIOUS DISEASES, Immunology < TROPICAL MEDICINE, Epidemiology < TROPICAL MEDICINE, Paediatric infectious disease & immunisation < PAEDIATRICS

SCHOLARONE™
Manuscripts

I, the Submitting Author has the right to grant and does grant on behalf of all authors of the Work (as defined in the below author licence), an exclusive licence and/or a non-exclusive licence for contributions from authors who are: i) UK Crown employees; ii) where BMJ has agreed a CC-BY licence shall apply, and/or iii) in accordance with the terms applicable for US Federal Government officers or employees acting as part of their official duties; on a worldwide, perpetual, irrevocable, royalty-free basis to BMJ Publishing Group Ltd ("BMJ") its licensees and where the relevant Journal is co-owned by BMJ to the co-owners of the Journal, to publish the Work in this journal and any other BMJ products and to exploit all rights, as set out in our licence.

The Submitting Author accepts and understands that any supply made under these terms is made by BMJ to the Submitting Author unless you are acting as an employee on behalf of your employer or a postgraduate student of an affiliated institution which is paying any applicable article publishing charge ("APC") for Open Access articles. Where the Submitting Author wishes to make the Work available on an Open Access basis (and intends to pay the relevant APC), the terms of reuse of such Open Access shall be governed by a Creative Commons licence – details of these licences and which Creative Commons licence will apply to this Work are set out in our licence referred to above.

Other than as permitted in any relevant BMJ Author's Self Archiving Policies, I confirm this Work has not been accepted for publication elsewhere, is not being considered for publication elsewhere and does not duplicate material already published. I confirm all authors consent to publication of this Work and authorise the granting of this licence.

For peer review only

The effect of intensive treatment for schistosomiasis on immune responses to vaccines among rural Ugandan island adolescents: randomised controlled trial protocol A for the 'POPulation differences in VACcine responses' (POPVAC) programme

Gyaviira Nkurunungi[1,¶,*], Ludoviko Zirimenya[1,¶], Jacent Nassuuna[1,¶], Agnes Natukunda[1,¶], Prossy N Kabuubi[1], Emmanuel Niwagaba[1], Gloria Oduru[1], Grace Kabami[1], Rebecca Amongin[1], Alex Mutebe [1], Milly Namutebi[1], Christopher Zziwa[1], Susan Amongi[1], Caroline Ninsiima[1], Caroline Onen[1], Florence Akello[1], Moses Sewankambo[1], Samuel Kiwanuka[1], Robert Kizindo[1], James Kaweesa[2], Stephen Cose[1,3], Emily L Webb[4], Alison M Elliott[1,3] **for the POPVAC trial team**

[1]Immunomodulation and Vaccines Programme, Medical Research Council/Uganda Virus Research Institute and London School of Hygiene and Tropical Medicine (MRC/UVRI and LSHTM) Uganda Research Unit, Entebbe, Uganda

[2]Vector Control Division, Ministry of Health, Kampala, Uganda

[3]Department of Clinical Research, London School of Hygiene and Tropical Medicine, London, United Kingdom

[4]MRC Tropical Epidemiology Group, Department of Infectious Disease Epidemiology, London School of Hygiene and Tropical Medicine, London, United Kingdom

¶These authors contributed equally

***Correspondence:** Gyaviira Nkurunungi; Gyaviira.Nkurunungi@mrcuganda.org

For peer review only

For peer review only - http://bmjopen.bmj.com/site/about/guidelines.xhtml

Abstract

Introduction

Several licensed and investigational vaccines have lower efficacy, and induce impaired immune responses, in low-income versus high-income countries and in rural, versus urban, settings. Understanding these population differences is essential to optimising vaccine effectiveness in the tropics. We suggest that repeated exposure to and immunomodulation by chronic helminth infections partly explains population differences in vaccine response.

Methods and analysis

We have designed an individually randomised, parallel group trial of intensive versus standard praziquantel (PZQ) intervention against schistosomiasis, to determine effects on vaccine response outcomes among school-going adolescents (9 to 17 years) from rural *Schistosoma mansoni* (*Sm*)-endemic Ugandan islands. Vaccines to be studied comprise BCG on day 'zero'; yellow fever, oral typhoid and Human Papilloma Virus (HPV) vaccines at week 4; and HPV and Tetanus/diphtheria booster vaccine at week 28. The intensive arm will receive PZQ doses three times, each two weeks apart, before BCG immunisation, followed by a dose at week 8 and quarterly thereafter. The standard arm will receive PZQ at week 8 and week 52. We expect to enrol 480 participants, with 80% *Sm* infected at the outset.

Primary outcomes are BCG-specific IFN-γ ELISpot responses eight weeks after BCG immunisation and for other vaccines, antibody responses to key vaccine antigens at four weeks after immunisation. Secondary analyses will determine effects of intensive anthelminthic treatment on correlates of protective immunity, on vaccine response waning, on priming vs boosting immunisations, and on *Sm* infection status and intensity. Exploratory immunology assays using archived samples will enable assessment of mechanistic links between helminths and vaccine responses.

Ethics and dissemination

Ethics approval has been obtained from relevant Ugandan and UK ethics committees. Results will be shared with Uganda Ministry of Health, relevant district councils, community leaders and study participants. Further dissemination will be done through conference proceedings and publications.

Trial registration

Current Controlled Trials identifier: ISRCTN60517191.

For peer review only - http://bmjopen.bmj.com/site/about/guidelines.xhtml

Article summary

Strengths and limitations of this study

- This will be the first adequately powered intervention study to investigate effects of schistosomiasis treatment on vaccine responses in adolescents.
- Effects on both live-attenuated and inert vaccines will be studied.
- Our strong immunoepidemiological design and nested immunological studies will address specific hypotheses regarding pathways of effects.
- The sample archives developed will provide a major asset for exploration of new leads arising from this hypothesis-driven work, or for an alternative, “systems biology” approach investigating (for example) transcriptome, microbiome and virome.
- Even with intensive anthelminthic intervention, it may be difficult to “successfully” treat *Schistosoma* infection in our endemic setting due to re-infections; however, we still expect a substantial difference in intensity between the two trial arms.

Word count

3250

Keywords

Vaccine; Schistosomiasis; Praziquantel; Immunization

For peer review only

Introduction

Vaccine-specific immune responses are often impaired, and vaccine efficacy and effectiveness lower, in tropical low-income countries (LICs) compared to temperate high-income countries and in rural, compared to urban, LIC settings.[1-8] This has been recognised for both live vaccines (such as BCG,[2 3 5 9] polio,[1] yellow fever[4] vaccines) and non-live vaccines (such as influenza[10] and tetanus[11]). Investigational malaria[7] and viral-vectored tuberculosis[6] and Ebola[12] vaccines are also affected. Previous exposure to the target pathogen (or related organisms) may mask the benefit of the vaccine.[13 14] However, pre-vaccination exposure does not explain why Ebola trial vaccine-specific responses differ between healthy UK and Senegalese adults,[12] as the target organism is rare. Therefore, environmentally-dependent mechanisms may play an important role.[5]

A long-held hypothesis is that parasites, particularly helminths, modulate vaccine responses through profound pre- and post-immunisation bystander effects on immunological activation and regulation.[15-17] Helminths might also impact vaccines responses through interactions with the complex ecosystem of mammalian gut bacteria, fungi, protozoa and viruses (the"trans-kingdom" concept[18] detailed elsewhere in this journal [bmjopen-2020-040425]). Helminth-induced gut mucosa damage, the associated translocation of microbial products into the systemic circulation[19-21] and systemic immune activation or regulation mediated by microbial products might contribute to modulation of responses to vaccines and other infections.

Helminth-mediated modulation of vaccine responses has not been substantiated in human populations. No appropriately powered trials have been conducted to evaluate reversibility of their effects. In animal models, helminths generally impair priming and accelerate waning of vaccine responses, although effects vary with helminth species, vaccine type and the timing of infection and immunisation.[22] Most observational studies in humans also suggest suppressed or biased responses during helminth infection, especially during systemic infections, such as schistosomiasis and the filariases. There is modest evidence that treating geohelminths in humans improves responses to BCG[23 24] or oral cholera vaccine[25] and we found that schistosomiasis treatment improved the measles-booster response in pre-school children.[26] There is therefore a strong case for a comprehensive assessment of the effects of helminths and their treatment on vaccine responses.

The extent to which helminths and related "trans-kingdom" mediators causally and reversibly impact immunological characteristics associated with vaccine responses may best be determined by intervention studies. This trial protocol A of the 'Population differences in Vaccine responses" programme (POPVAC A; Current Controlled Trials identifier: ISRCTN60517191) has been designed to evaluate the effect of *Schistosoma mansoni* and its treatment on vaccine responses. This study is

For peer review only - http://bmjopen.bmj.com/site/about/guidelines.xhtml

101 one of three parallel trials whose designs and cross-cutting analyses are described separately in this
102 journal (bmjopen-2020-040425, bmjopen-2020-040427 and bmjopen-2020-040430).

For peer review only

For peer review only - http://bmjopen.bmj.com/site/about/guidelines.xhtml

Hypothesis

The overarching goal of the POPVAC programme is to understand population differences in vaccine responses in Uganda, in order to identify strategies through which vaccine effectiveness can be optimised for the low-income, tropical settings where they are especially needed. For this Trial A, we focus on the hypothesis that *Schistosoma mansoni infection suppresses responses to unrelated vaccines; and that this effect can be reversed, at least in part, by intensive praziquantel treatment intervention.*

Objective

To determine whether there are reversible effects of chronic *Schistosoma mansoni* infection on vaccine response in adolescents, using an intervention study.

For peer review only

For peer review only - http://bmjopen.bmj.com/site/about/guidelines.xhtml

Methods and analysis

Setting and participants

SPIRIT reporting guidelines[27] have been used. We will conduct an individually randomised, parallel group trial of intensive versus standard intervention against schistosomiasis (described below) in the *S. mansoni*-endemic Koome islands of Lake Victoria, Mukono district, Uganda.[28] We aim to enroll 480 participants, randomising 240 to each intervention arm. The study cohort will recruit participants aged 9 to 17 years in primary school years 1 to 6. Adolescents[29] in this study setting bear a heavy parasite burden.[30] In addition, this age-group is a target group for vaccines against sexually transmitted infections (currently human papilloma virus [HPV] – in future, it is hoped, for vaccines against HIV) and for booster immunisations.

Recruitment criteria

Inclusion criteria

i. Attending the selected school and planning to continue to attend the school for the duration of the study
ii. Aged 9 to 17 years and enrolled in primary 1 to 6 (to avoid primary leaving examinations in late year 7, and loss to follow up of children leaving after primary 7)
iii. Written informed assent by participant and consent by parent or guardian
iv. Females agree to avoid pregnancy for the duration of the trial
v. Willing to provide locator information and to be contacted during the course of the trial
vi. Able and willing (in the investigator's opinion) to comply with all the study requirements

Exclusion criteria

i. Clinically significant history of immunodeficiency (including HIV), cancer, cardiovascular disease, gastrointestinal disease, liver disease, renal disease, endocrine disorder and neurological illness
ii. History of serious psychiatric condition or disorder
iii. Moderate or severe acute illness characterised by any of the following symptoms: fever, impaired consciousness, convulsions, difficulty in breathing, vomiting; or as otherwise determined by the attending project clinician.
iv. Concurrent oral or systemic steroid medication or the concurrent use of other immunosuppressive agents within 2 months prior to enrolment
v. History of allergic reaction to immunisation or any allergy likely to be exacerbated by any component of the study vaccines including egg or chicken proteins

For peer review only - http://bmjopen.bmj.com/site/about/guidelines.xhtml

vi. History of previous immunisation with Yellow Fever (YF), oral typhoid or Human Papillomavirus (HPV) vaccine; previous immunisation with BCG or Tetanus and diphtheria vaccine (Td) at age $\geq$5 years

vii. Tendency to develop keloid scars

viii. Haemoglobin less than 82g/L

ix. Positive HIV serology

x. Positive pregnancy test

xi. Female currently lactating, confirmed pregnancy or intention to become pregnant during the trial period

xii. Use of an investigational medicinal product or non-registered drug, live vaccine, or medical device other than the study vaccines for 30 days prior to dosing with the study vaccine, or planned use during the study period

xiii. Administration of immunoglobulins and/or any blood products within the three months preceding the planned trial immunisation date

Further information on recruitment criteria can be found in Supplementary information.

Interventions

We will individually randomise participants to intensive or standard praziquantel (PZQ) treatment, in a 1:1 ratio. The intensive arm will receive three doses of PZQ (approximately 40mg/kg, assessed by height pole[31]) each two weeks apart prior to the first immunisation (the last of these 2-3 weeks before immunisation), followed by PZQ at 8 weeks (after primary endpoint sampling) and thereafter quarterly PZQ (approximately; timings adjusted to accommodate school terms) during follow up. The standard arm will receive their first dose of PZQ at week 8 (after immunisation and after primary endpoint sampling) and a second dose at week 52 (to conform to Uganda Ministry of Health policy, which is annual treatment) (**Figure 1**). No placebo will be used in this trial because all participants will be treated (albeit at different frequencies) and participants are unlikely to seek additional treatments outside the trial schedule: praziquantel treatment is not popular because of the recognised (although temporary) adverse effects (described in Supplementary information).

Randomisation and allocation to treatment arm

A randomisation code will be generated by an independent statistician using a randomly permuted block size (sizes 4, 6, 8 and 10) and used to allocate participants to either receive quarterly PZQ (intensive arm) or annual PZQ (standard arm). A set of envelopes will be prepared, labelled sequentially with the randomisation numbers and containing a card indicating the corresponding allocation (to intensive or standard treatment). The randomisation code will be kept securely by the

For peer review only - http://bmjopen.bmj.com/site/about/guidelines.xhtml

For peer review only

trial statistician and made available only to those responsible for providing or preparing the trial interventions. A second copy will be held by a data manager or statistician not otherwise involved in the trial at the MRC/UVRI and LSHTM Uganda Research Unit. At enrolment, eligibility criteria will be checked and eligible participants will be allocated sequentially to the next randomisation number until the required sample size is achieved. Randomisation implementation will be done by a clinician using the sequentially numbered opaque sealed envelopes. When the next randomisation number in the sequence is allocated, the envelope bearing that number will be opened to reveal the allocation.

Blinding

Clinicians and participants will not be blinded to the treatment allocation since they will not participate in outcome ascertainment; only immunology laboratory staff who are assessing trial outcomes will be blinded.

Immunisations

We will study a portfolio of licensed vaccines (live and inert, oral and parental, priming and boosting) expected to be beneficial (in some cases, already given) to adolescents in Uganda. Our schedule (**Table 1,** supplementary **Table S1**) will comprise three main immunisation days (week 0, week 4 and week 28). Additional HPV immunisation will be provided for girls aged 14 years or above, and a second Tetanus/diphtheria boost will be given after completion of the study, to accord with the national Expanded Programme on Immunisation (EPI) routines, but the response to these will not specifically be assessed. Further rationale for the selection of vaccines is detailed in the Supplementary information.

For peer review only

Table 1. Immunisation schedule

	Immunisation week 0	Immunisation week 4	[Immunisation week 8]	Immunisation week 28	[Immunisation week 52]
**Live vaccines**	BCG vaccination / re-vaccination[1]	Yellow fever (YF-17D)<br>Oral typhoid (Ty21a)			
**Non-live vaccines**		HPV prime[2]	HPV boost for girls aged $\geq$14 years[3,4]	HPV boost[2] and Tetanus/ diphtheria (Td) boost	Tetanus/ diphtheria (Td) boost[4,5]

1. Prior BCG status may vary (data on history and documentation of prior BCG, and presence of a BCG scar, will be documented although these approaches have limitations for determining BCG status)
2. Both girls and boys will receive the HPV vaccine
3. The National EPI programme recommends three doses of HPV vaccine for older girls
4. These doses will be given to comply with guidelines but outcomes specifically relating to these doses will not be assessed
5. Priming by immunisation in infancy is assumed

For peer review only - http://bmjopen.bmj.com/site/about/guidelines.xhtml

Schedule of immunisation and sampling

The schedule of immunisation and sampling is outlined in **Figure 1** and **Table S1**. While optimal timings for outcome measures vary between vaccines, sampling at 8 weeks post BCG and 4 weeks post YF-17D, Ty21a, HPV and Td is proposed for the primary endpoints, targeting the establishment of memory responses and approximate peak of antibody responses. A secondary endpoint at one year will assess waning. All analyses will take baseline measurements into account. Immunisation postponement criteria are detailed in Supplementary information.

Outcomes

Primary outcomes

These will be assessed in all participants.

i. **BCG:** BCG-specific IFN-γ ELISpot response eight weeks post BCG immunisation.
ii. **YF-17D:** neutralising antibody titres (plaque-reduction neutralisation test) at four weeks post YF immunisation.
iii. **Ty21a:** *Salmonella typhi* lipopolysaccharide (LPS)-specific immunoglobulin (Ig)G concentration at four weeks post Ty21a immunisation.
iv. **HPV:** IgG specific for L1-proteins of HPV-16/18 at four weeks post HPV priming immunisation.
v. **Td:** Tetanus and diphtheria toxoid-specific IgG concentration at four weeks post Td immunisation.

Secondary outcomes

These will be assessed in all participants and will further investigate estimates of protective immunity (for vaccines where these are available) and dynamics of the vaccine responses, as well as the impact of the interventions on parasite clearance.

i. **Protective immunity.** Proportions with protective neutralising antibody (YF); protective IgG levels (TT);[32] seroconversion rates (Ty21a) at four weeks post the corresponding immunisation.
ii. **Response waning.** Primary outcome measures (all vaccines) repeated at week 52, and area-under-the curve (AUC) analyses. Parasitic infection may accelerate,[33] and anti-parasitic interventions delay, waning.
iii. **Priming versus boosting.** Effects on priming versus boosting will be examined for HPV only, comparing outcomes four weeks after the first, and four weeks after the second vaccine dose.

For peer review only

iv. **Current *S. mansoni* infection status and intensity** will be determined by serum/plasma levels of circulating anodic antigen (CAA). The method is quantitative, highly specific for *Schistosoma* infection, and much more sensitive than the conventional Kato Katz method.[34] CAA will be assessed retrospectively on stored samples collected at baseline, on immunisation days, and on primary and secondary endpoint days.

Furthermore, our sample collection will offer opportunities for an array of exploratory immunological evaluations on stored samples, focusing mainly on vaccine antigen specific outcomes. Exploratory assays will provide further detail on the role of immunological profiles and trans-kingdom effects in mediating helminth modulation of vaccine-specific responses.

Additional evaluation of parasite infection exposure

i. **Prior exposure to schistosomiasis** will be evaluated by ELISA for IgG to schistosome egg antigen using stored blood samples collected at baseline.

ii. **The presence of other helminth infections** will be determined retrospectively using stool PCR of samples collected at baseline and at weeks 28 and 52.[30] In accordance with national guidelines, all participants will be treated with albendazole or mebendazole after collection of samples for primary endpoints at week 8 and 28, and after collection of samples for secondary endpoints at week 52.

iii. **Current malaria infection status and intensity** will be assessed retrospectively by PCR on stored samples collected on immunisation days and at week 52. Individuals presenting with fever will be investigated using rapid diagnostic tests for malaria and treated based on the results and according to prevailing national guidelines.

iv. **Prior malaria exposure** will be evaluated by ELISA for IgG to malaria antigen using stored samples collected at baseline.

Sample size considerations

Based on the literature[4 35 36] and preliminary data, we anticipate that, following log to base 10 transformations that will be applied to normalise primary outcome measures, standard deviations (SDs) of primary outcome measures will lie between 0.3 and 0.6 on this log scale, and that effective treatment may increase responses by approximately 0.2 on the log scale (based on Tweyongyere *et al.*[26]). We have therefore powered our study to detect differences of this magnitude (0.2 on the log scale) or (in some cases) smaller (**Table 2**). We assume *S. mansoni* prevalence of $\geq$80%.

For peer review only - http://bmjopen.bmj.com/site/about/guidelines.xhtml

Based on these assumptions, we plan to include 480 participants in total (240 quarterly PZQ, 240 annual PZQ); of whom 384 are expected to be *S. mansoni* infected,[28] giving 192 participants in each trial arm who are infected at baseline.

Table 2 shows power estimates, for 5% significance level and assuming 20% loss to follow-up.

Table 2. Power estimates (5% significance level)

Standard deviation (log_{10})	Difference in mean log_{10} transformed outcome, between trial arms						
	**0.08**	**0.10**	**0.12**	**0.14**	**0.16**	**0.18**	**0.20**
192 intensive PZQ vs 192 standard PZQ (*S. mansoni* infected only)							
0.3	65%	83%	94%	98%	>99%	>99%	>99%
0.4	42%	59%	75%	87%	94%	98%	99%
0.5	29%	42%	56%	69%	80%	88%	94%
0.6	21%	31%	42%	53%	65%	75%	83%

Cells highlighted in grey correspond to >80% power; differences in mean log10 transformed outcome of 0.08, 0.10, 0.12, 0.14, 0.16, 0.18 and 0.20 are equivalent to geometric mean ratios for untransformed outcomes of 1.20, 1.26, 1.32, 1.38, 1.45, 1.51 and 1.59, respectively.

Ethics and dissemination

Ethical approval has been granted from the Research Ethics Committee of the Uganda Virus Research Institute (UVRI REC, reference: GC/127/19/05/664) and the London School of Hygiene and Tropical Medicine (LSHTM, reference: 16032), and from the Uganda National Council for Science and Technology (UNCST, reference: HS2486) and the Uganda National Drug Authority (NDA, reference: CTA0093). Any protocol amendments will be submitted to ethics committees and regulatory bodies for approval before implementation.

Participants are adolescents and therefore a vulnerable human population. Care will be taken to provide adequate, age and education-status appropriate information and to ensure that it is understood; and to emphasise that participation is voluntary. Participants will be enrolled only when they have given their own assent and when consent has been given by the parent or guardian. No major risks to the participants are anticipated since all the treatments and vaccines to be given are licensed and known to be safe. The main risk to participants will be time lost from school work: we will work with teachers and parents to minimise disruption to classes, and will avoid enrolment of primary 7 students since these classes are involved in national examinations. Further risks are discussed in Supplementary information.

Study findings will be published through open access peer-reviewed journals, presentations at local, national and international conferences and to the local community through community meetings. Anonymised participant level datasets generated will be available upon request.

For peer review only

Patient and public involvement

Concepts involved in this work have been discussed with colleagues at the Vector Control Division and Expanded Programme on Immunisation in the Ministry of Health (Uganda), with the Mukono District Council and with community leaders and Village Health Teams from Koome subcounty. We also have held meetings to explain the proposed work to teachers, parents, participants and village members, and to address their questions about issues such as study length, the study's ethical approval status, why adults were excluded from the study, and to explain to them why boys will also receive the HPV vaccine. Study findings will be shared with these stakeholders and with participants.

Data management and analysis

Socio-demographic information and clinical and laboratory measurements will be recorded and managed using Research Electronic Data Capture (REDCap) tools,[37 38] with paper-based forms as back-up. All data will be recorded under a unique study ID number. When paper forms must be used, data will be double entered in a study-specific database, with standard checks for discrepancies. All data for analysis will be anonymised and stored on a secure and password-protected server, with access limited to essential research personnel.

Baseline characteristics will be summarised by trial arm, and the effect of intensive (compared to standard) praziquantel treatment on the outcomes will be analysed. Information on infection status will only be available after randomisation. The primary analysis will be done on individuals identified as infected at baseline (through randomisation, these will be balanced between treatment arms); this will test the hypothesis that treating the infection (and subsequent reinfections) reverses the parasite's effects on vaccine responses. If treating *S. mansoni* reverses adverse parasite effects on vaccine responses, this may be a beneficial public health intervention. However, routine screening for parasite infection before immunisation would be laborious. Secondary analyses will include all randomised individuals; this will provide insight into the broader benefit of the interventions as public health measures. The effect of intensive versus standard praziquantel treatment on primary outcomes will be assessed using unpaired t-tests, with results presented as a mean difference in vaccine response measure together with 95% confidence interval and p-value. We anticipate that outcomes will be positively skewed, and will apply log transformations to normalise distributions before analysis if required. The detailed analytical plan is available on the online trial registration site (http://www.isrctn.com/ISRCTN60517191).

For peer review only

Discussion

This will be the first adequately powered intervention study to investigate the effect of schistosomiasis treatment on vaccine responses in adolescents. This study will determine whether *S. mansoni* infection has a causal, reversible, impact on the response to live or inert vaccines, including effects on vaccine replication, immune response profile, priming, boosting and waning. The results will add to understanding of population differences in vaccine responses and on interventions that may enhance responses. If treating helminths improves vaccine responses in adolescents, combined parasite-control/immunisation programmes offer an attractive, practical public health intervention for schools and communities.

There are risks associated with our approach to addressing the trial objective. **First**, there is a risk of failure to clear *S. mansoni* infections, and repeated reinfection during the trial. This issue can be challenging because of incomplete cure or maturation of immature worms after treatment, and lifestyles in endemic communities that result in repeated exposure. To mitigate this, we will administer three PZQ treatments over a six-week period before the first immunisations, and continuing quarterly treatment in the intensive arm. **Second,** there is a risk that *S. mansoni* infection has long-term effects, not removed by treatment, mediated, for example, by epigenetic change.[39] However, studies show that parasite treatment results in immunological changes,[40 41] and our data suggest at least partial recovery of the measles vaccine response among young children treated for schistosomiasis.[26] By initiating intervention six to eight weeks before the first immunisations, and providing repeated intervention in the intensive arms, we hope to achieve significant resolution of *S. mansoni* effects.

We are interested in the effects of removing *S. mansoni*. Treating parasites can induce acute immunological change due to release of previously hidden antigens.[42 43] To minimise such effects, immunisations will be given at least 2 weeks after PZQ (the longest practicable interval; **Figure 1**).

Laboratory analyses will also highlight immune parameters and cellular populations that link environmental exposures to vaccine responses. Identifying processes associated with poor or good outcomes will inform strategies in vaccine design (for example, the genetic modification of vaccines, or innovative use of adjuvants, to counter any adverse immunological milieu, currently an area of intense research for cancer vaccines[44]); ultimately supporting the development of effective vaccines tailored to the low-income settings that most need them.

Study timeline

For peer review only - http://bmjopen.bmj.com/site/about/guidelines.xhtml

Applications for ethical approval were submitted in May 2018, with approval received in September 2018 (Uganda Virus Research Institute Research Ethics Committee), May 2019 (National Drug Authority and Uganda National Council for Science and Technology), June 2019 (London School of Hygiene and Tropical Medicine). Collaborator/investigator/trial steering committee meetings were also held during the initial 12-month planning period. The study began recruitment in July 2019. Intervention will be up to 12 months, with completion of the project scheduled for September 2020.

Competing interests

Alison Elliott reports a grant from the Medical research Council, UK (POPVAC programme funding). The rest of the authors declare that they have no conflicts of interest.

Author contributions

AME conceived the study. AME, GN, ELW, AN, JN, SC, LZ and JK contributed to study design. LZ, GO, PNK, EN, GK, RA, CN, CO, MN, CZ, SA and FA are site clinicians/nurses/clinical laboratory technicians providing valuable input on clinical considerations of the intervention. MS, SK and RK are field workers handling the organisational integration of the intervention. AN, AM and ELW are involved in organisation of the databases, trial randomization, treatment allocation and drawing up of analytical plans. GN, LZ, JN, AN, SC, ELW and AME drafted the manuscript. All authors reviewed the manuscript, contributed to it and approved the final version.

Acknowledgements

We thank the Uganda National Expanded Programme for Immunisation, Sanofi Pasteur and PaxVax for providing the HPV, yellow fever and oral typhoid vaccines, respectively. The BCG and tetanus-diphtheria vaccines were kind donations from the Serum Institute of India. We thank the Vector Control Division of the Ministry of Health and the Mukono district local government for their support. We also thank members of the POPVAC programme steering committee (chaired by Prof. Richard Hayes) and the Data and Safety Monitoring Board (Dr David Meya, Prof Andrew Prendergast and Dr Elizabeth George).

Funding

The POPVAC programme of work is supported by the Medical Research Council of the United Kingdom (grant number MR/R02118X/1). SC and JN are supported in part by the Makerere University – Uganda Virus Research Institute Centre of Excellence for Infection and Immunity Research and Training (MUII-plus). MUII-plus is funded under the DELTAS Africa Initiative. The DELTAS Africa Initiative is an independent funding scheme of the African Academy of Sciences (AAS),

For peer review only - http://bmjopen.bmj.com/site/about/guidelines.xhtml

Alliance for Accelerating Excellence in Science in Africa (AESA) and supported by the New Partnership for Africa’s Development Planning and Coordinating Agency (NEPAD Agency) with funding from the Wellcome Trust (grant 107743) and the UK Government. The MRC/UVRI and LSHTM Uganda Research Unit is jointly funded by the UK Medical Research Council (MRC) and the UK Department for International Development (DFID) under the MRC/DFID Concordat agreement and is also part of the EDCTP2 programme supported by the European Union.

The study sponsor (London School of Hygiene and Tropical Medicine) and funders had no role in study design; collection, management, analysis, and interpretation of data; writing of the protocol; and the decision to submit the protocol for publication.

POPVAC trial team

Principal investigator: Alison Elliott; ***Project leader***: Ludoviko Zirimenya; ***laboratory staff:*** Gyaviira Nkurunungi, Stephen Cose, Rebecca Amongin, Beatrice Nassanga, Jacent Nassuuna, Irene Nambuya, Prossy Kabuubi, Emmanuel Niwagaba, Gloria Oduru, Grace Kabami; ***statisticians and data managers:*** Emily Webb, Agnes Natukunda, Helen Akurut, Alex Mutebe; ***clinicians:*** Anne Wajja, Milly Namutebi, Christopher Zziwa, Joel Serubanja; ***nurses:*** Caroline Onen, Esther Nakazibwe, Josephine Tumusiime, Caroline Ninsiima, Susan Amongi, Florence Akello; ***internal monitor:*** Mirriam Akello; ***field workers:*** Robert Kizindo, Moses Sewankambo, Denis Nsubuga, Samuel Kiwanuka, Fred Kiwudhu; ***boatman:*** David Abiriga; ***administrative management:*** Moses Kizza, Samsi Nansukusa; ***internal and external collaborators:*** Pontiano Kaleebu, Hermelijn Smits, Maria Yazdanbakhsh, Govert van Dam, Paul Corstjens, Sarah Staedke, Henry Luzze, James Kaweesa, Edridah Tukahebwa, Elly Tumushabe, Moses Muwanga

For peer review only

References

1. Taniuchi M, Platts-Mills JA, Begum S, et al. Impact of enterovirus and other enteric pathogens on oral polio and rotavirus vaccine performance in Bangladeshi infants. *Vaccine* 2016;34(27):3068-75. doi: 10.1016/j.vaccine.2016.04.080 [published Online First: 2016/05/08]

2. Fine PE. Variation in protection by BCG: implications of and for heterologous immunity. *Lancet* 1995;346(8986):1339-45.

3. Black GF, Weir RE, Floyd S, et al. BCG-induced increase in interferon-gamma response to mycobacterial antigens and efficacy of BCG vaccination in Malawi and the UK: two randomised controlled studies. *Lancet* 2002;359(9315):1393-401. doi: 10.1016/S0140-6736(02)08353-8

4. Muyanja E, Ssemaganda A, Ngauv P, et al. Immune activation alters cellular and humoral responses to yellow fever 17D vaccine. *The Journal of clinical investigation* 2014;124(7):3147-58. doi: 10.1172/jci75429 [published Online First: 2014/06/10]

5. Barreto ML, Pilger D, Pereira SM, et al. Causes of variation in BCG vaccine efficacy: examining evidence from the BCG REVAC cluster randomized trial to explore the masking and the blocking hypotheses. *Vaccine* 2014;32(30):3759-64. doi: 10.1016/j.vaccine.2014.05.042

6. Tanner R, Kakalacheva K, Miller E, et al. Serum indoleamine 2,3-dioxygenase activity is associated with reduced immunogenicity following vaccination with MVA85A. *BMC infectious diseases* 2014;14:660. doi: 10.1186/s12879-014-0660-7 [published Online First: 2014/12/04]

7. Sissoko MS, Healy SA, Katile A, et al. Safety and efficacy of PfSPZ Vaccine against Plasmodium falciparum via direct venous inoculation in healthy malaria-exposed adults in Mali: a randomised, double-blind phase 1 trial. *The Lancet Infectious diseases* 2017;17(5):498-509. doi: 10.1016/s1473-3099(17)30104-4 [published Online First: 2017/02/22]

8. Venkatraman N, Ndiaye BP, Bowyer G, et al. Safety and Immunogenicity of a Heterologous Prime-Boost Ebola Virus Vaccine Regimen in Healthy Adults in the United Kingdom and Senegal. *The Journal of infectious diseases* 2018;219(8):1187-97. doi: 10.1093/infdis/jiy639

9. Barreto ML, Pereira SM, Pilger D, et al. Evidence of an effect of BCG revaccination on incidence of tuberculosis in school-aged children in Brazil: second report of the BCG-REVAC cluster-randomised trial. *Vaccine* 2011;29(31):4875-7. doi: 10.1016/j.vaccine.2011.05.023

10. van Riet E, Adegnika AA, Retra K, et al. Cellular and humoral responses to influenza in gabonese children living in rural and semi-urban areas. *The Journal of infectious diseases* 2007;196(11):1671-8. doi: 10.1086/522010 [published Online First: 2007/11/17]

11. van Riet E, Retra K, Adegnika AA, et al. Cellular and humoral responses to tetanus vaccination in Gabonese children. *Vaccine* 2008;26(29-30):3690-5. doi: 10.1016/j.vaccine.2008.04.067 [published Online First: 2008/06/10]

12. Venkatraman N, Ndiaye BP, Bowyer G, et al. Safety and Immunogenicity of a Heterologous Prime-Boost Ebola Virus Vaccine Regimen in Healthy Adults in the United Kingdom and Senegal. *The Journal of infectious diseases* 2019;219(8):1187-97. doi: 10.1093/infdis/jiy639

13. Brandt L, Feino Cunha J, Weinreich Olsen A, et al. Failure of the Mycobacterium bovis BCG vaccine: some species of environmental mycobacteria block multiplication of BCG and induction of protective immunity to tuberculosis. *Infection and immunity* 2002;70(2):672-8. [published Online First: 2002/01/18]

14. Flaherty DK, Vesosky B, Beamer GL, et al. Exposure to Mycobacterium avium can modulate established immunity against Mycobacterium tuberculosis infection generated by Mycobacterium bovis BCG vaccination.

For peer review only

For peer review only - http://bmjopen.bmj.com/site/about/guidelines.xhtml

442 *Journal of leukocyte biology* 2006;80(6):1262-71. doi: 10.1189/jlb.0606407 [published Online First:
443 2006/09/14]

444 15. Maizels RM, McSorley HJ. Regulation of the host immune system by helminth parasites. *The Journal of*
445 *allergy and clinical immunology* 2016;138(3):666-75. doi: 10.1016/j.jaci.2016.07.007 [published Online First:
446 2016/08/02]

447 16. Wammes LJ, Mpairwe H, Elliott AM, et al. Helminth therapy or elimination: epidemiological,
448 immunological, and clinical considerations. *The Lancet Infectious diseases* 2014;14(11):1150-62. doi:
449 10.1016/S1473-3099(14)70771-6

450 17. Wammes LJ, Hamid F, Wiria AE, et al. Regulatory T cells in human geohelminth infection suppress immune
451 responses to BCG and Plasmodium falciparum. *Eur J Immunol* 2010;40(2):437-42. doi: 10.1002/eji.200939699

452 18. Pfeiffer JK, Virgin HW. Viral immunity. Transkingdom control of viral infection and immunity in the
453 mammalian intestine. *Science* 2016;351(6270) doi: 10.1126/science.aad5872

454 19. Onguru D, Liang Y, Griffith Q, et al. Human schistosomiasis is associated with endotoxemia and Toll-like
455 receptor 2- and 4-bearing B cells. *The American journal of tropical medicine and hygiene* 2011;84(2):321-4.
456 doi: 10.4269/ajtmh.2011.10-0397 [published Online First: 2011/02/05]

457 20. George PJ, Anuradha R, Kumar NP, et al. Evidence of microbial translocation associated with perturbations
458 in T cell and antigen-presenting cell homeostasis in hookworm infections. *PLoS neglected tropical diseases*
459 2012;6(10):e1830. doi: 10.1371/journal.pntd.0001830 [published Online First: 2012/10/12]

460 21. Rajamanickam A, Munisankar S, Bhootra Y, et al. Microbial Translocation Associated with an Acute-Phase
461 Response and Elevations in MMP-1, HO-1, and Proinflammatory Cytokines in Strongyloides stercoralis
462 Infection. *Infection and immunity* 2017;85(1) doi: 10.1128/iai.00772-16 [published Online First: 2016/11/09]

463 22. Sanya RE, Nkurunungi G, Andia Biraro I, et al. A life without worms. *Transactions of the Royal Society of*
464 *Tropical Medicine and Hygiene* 2017:1-9. doi: 10.1093/trstmh/trx010 [published Online First: 2017/03/25]

465 23. Elias D, Britton S, Aseffa A, et al. Poor immunogenicity of BCG in helminth infected population is associated
466 with increased in vitro TGF-beta production. *Vaccine* 2008;26(31):3897-902. doi: S0264-410X(08)00540-9
467 [pii]
468 10.1016/j.vaccine.2008.04.083 [published Online First: 2008/06/17]

469 24. Elias D, Wolday D, Akuffo H, et al. Effect of deworming on human T cell responses to mycobacterial
470 antigens in helminth-exposed individuals before and after bacille Calmette-Guerin (BCG) vaccination. *Clin Exp*
471 *Immunol* 2001;123(2):219-25.

472 25. Cooper PJ, Chico ME, Losonsky G, et al. Albendazole treatment of children with ascariasis enhances the
473 vibriocidal antibody response to the live attenuated oral cholera vaccine CVD 103-HgR. *The Journal of*
474 *infectious diseases* 2000;182(4):1199-206. doi: 10.1086/315837 [published Online First: 2000/09/09]

475 26. Tweyongyere R, Nassanga BR, Muhwezi A, et al. Effect of Schistosoma mansoni infection and its treatment
476 on antibody responses to measles catch-up immunisation in pre-school children: A randomised trial. *PLoS*
477 *neglected tropical diseases* 2019;13(2):e0007157. doi: 10.1371/journal.pntd.0007157 [published Online First:
478 2019/02/15]

479 27. Chan AW, Tetzlaff JM, Altman DG, et al. SPIRIT 2013 statement: defining standard protocol items for
480 clinical trials. *Ann Intern Med* 2013;158(3):200-7. doi: 10.7326/0003-4819-158-3-201302050-00583
481 [published Online First: 2013/01/09]

482 28. Sanya RE, Nkurunungi G, Hoek Spaans R, et al. The Impact of Intensive Versus Standard Anthelminthic
483 Treatment on Allergy-related Outcomes, Helminth Infection Intensity, and Helminth-related Morbidity in
484 Lake Victoria Fishing Communities, Uganda: Results From the LaVIISWA Cluster-randomized Trial. *Clinical*

For peer review only - http://bmjopen.bmj.com/site/about/guidelines.xhtml

infectious diseases : an official publication of the Infectious Diseases Society of America 2019;68(10):1665-74. doi: 10.1093/cid/ciy761 [published Online First: 2018/09/12]

29. WHO. Adolescent Health Research Priorities: Report of a Technical Consultation 2015. http://apps.who.int/iris/bitstream/10665/203564/1/WHO_FWC_MCA_15_07_eng.pdf?ua=1 (accessed 17th May 2019).

30. Webb EL, Nampijja M, Kaweesa J, et al. Helminths are positively associated with atopy and wheeze in Ugandan fishing communities: results from a cross-sectional survey. *Allergy* 2016 doi: 10.1111/all.12867

31. Montresor A, Odermatt P, Muth S, et al. The WHO dose pole for the administration of praziquantel is also accurate in non-African populations. *Transactions of the Royal Society of Tropical Medicine and Hygiene* 2005;99(1):78-81. doi: 10.1016/j.trstmh.2004.06.006

32. Plotkin SA. Correlates of protection induced by vaccination. *Clinical and vaccine immunology : CVI* 2010;17(7):1055-65. doi: 10.1128/cvi.00131-10 [published Online First: 2010/05/14]

33. Riner DK, Ndombi EM, Carter JM, et al. Schistosoma mansoni Infection Can Jeopardize the Duration of Protective Levels of Antibody Responses to Immunizations against Hepatitis B and Tetanus Toxoid. *PLoS neglected tropical diseases* 2016;10(12):e0005180. doi: 10.1371/journal.pntd.0005180

34. Corstjens PL, Nyakundi RK, de Dood CJ, et al. Improved sensitivity of the urine CAA lateral-flow assay for diagnosing active Schistosoma infections by using larger sample volumes. *Parasites & vectors* 2015;8:241. doi: 10.1186/s13071-015-0857-7 [published Online First: 2015/04/22]

35. Fletcher HA, Snowden MA, Landry B, et al. T-cell activation is an immune correlate of risk in BCG vaccinated infants. *Nat Commun* 2016;7:11290. doi: 10.1038/ncomms11290

36. Safaeian M, Porras C, Pan Y, et al. Durable antibody responses following one dose of the bivalent human papillomavirus L1 virus-like particle vaccine in the Costa Rica Vaccine Trial. *Cancer prevention research (Philadelphia, Pa)* 2013;6(11):1242-50. doi: 10.1158/1940-6207.capr-13-0203 [published Online First: 2013/11/06]

37. Harris PA, Taylor R, Minor BL, et al. The REDCap Consortium: Building an International Community of Software Platform Partners. *Journal of biomedical informatics* 2019:103208. doi: 10.1016/j.jbi.2019.103208 [published Online First: 2019/05/13]

38. Harris PA, Taylor R, Thielke R, et al. Research electronic data capture (REDCap)--a metadata-driven methodology and workflow process for providing translational research informatics support. *Journal of biomedical informatics* 2009;42(2):377-81. doi: 10.1016/j.jbi.2008.08.010 [published Online First: 2008/10/22]

39. Blok BA, Arts RJ, van Crevel R, et al. Trained innate immunity as underlying mechanism for the long-term, nonspecific effects of vaccines. *J Leukoc Biol* 2015;98(3):347-56. doi: 10.1189/jlb.5RI0315-096R

40. Watanabe K, Mwinzi PN, Black CL, et al. T regulatory cell levels decrease in people infected with Schistosoma mansoni on effective treatment. *The American journal of tropical medicine and hygiene* 2007;77(4):676-82. [published Online First: 2007/11/06]

41. Schmiedel Y, Mombo-Ngoma G, Labuda LA, et al. CD4+CD25hiFOXP3+ Regulatory T Cells and Cytokine Responses in Human Schistosomiasis before and after Treatment with Praziquantel. *PLoS neglected tropical diseases* 2015;9(8):e0003995. doi: 10.1371/journal.pntd.0003995 [published Online First: 2015/08/21]

42. van den Biggelaar AHJ, Borrmann S, Kremsner P, et al. Immune Responses Induced by Repeated Treatment Do Not Result in Protective Immunity to Schistosoma haematobium: Interleukin (IL)–5 and IL-10 Responses. *The Journal of infectious diseases* 2002;186(10):1474-82. doi: 10.1086/344352

For peer review only

For peer review only - http://bmjopen.bmj.com/site/about/guidelines.xhtml

43. Woolhouse MEJ, Hagan P. Seeking the ghost of worms past. *Nature Medicine* 1999;5(11):1225-27. doi: 10.1038/15169

44. Seledtsov VI, Goncharov AG, Seledtsova GV. Clinically feasible approaches to potentiating cancer cell-based immunotherapies. *Human vaccines & immunotherapeutics* 2015;11(4):851-69. doi: 10.1080/21645515.2015.1009814 [published Online First: 2015/05/02]

FIGURE LEGENDS

Figure 1. Outline of immunisations and anthelminthic intervention

[1]Primary endpoints will be at eight weeks post BCG and four weeks post Yellow fever (YF-17D), oral typhoid (Ty21a), Human Papilloma Virus (HPV) and Tetanus/diptheria (Td) vaccination.

[2]Primary endpoint for responses to Td given at 28 weeks.

For peer review only

For peer review only - http://bmjopen.bmj.com/site/about/guidelines.xhtml

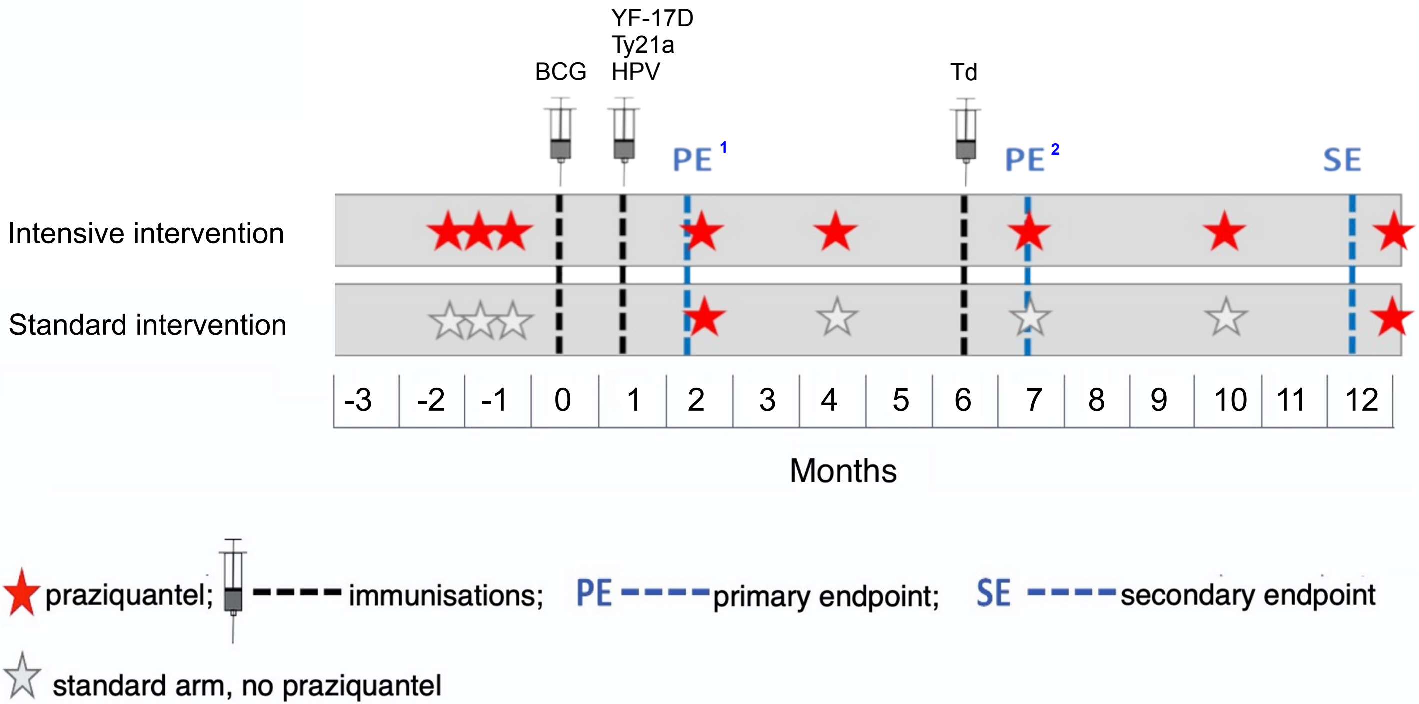

For peer review only - http://bmjopen.bmj.com/site/about/guidelines.xhtml

SUPPLEMENTARY INFORMATION

The effect of intensive treatment for schistosomiasis on immune responses to vaccines among rural Ugandan island adolescents: randomised controlled trial protocol A for the 'POPulation differences in VACcine responses' (POPVAC) programme

Gyaviira Nkurunungi[1,¶,*], Ludoviko Zirimenya[1,¶], Jacent Nassuuna[1,¶], Agnes Natukunda[1,¶], Prossy N Kabuubi[1], Emmanuel Niwagaba[1], Gloria Oduru[1], Grace Kabami[1], Rebecca Amongin[1], Alex Mutebe[1], Milly Namutebi[1], Christopher Zziwa[1], Susan Amongi[1], Caroline Ninsiima[1], Caroline Onen[1], Florence Akello[1], Moses Sewankambo[1], Samuel Kiwanuka[1], Robert Kizindo[1], James Kaweesa[2], Stephen Cose[1,3], Emily L Webb[4], Alison M Elliott[1,3] **for the POPVAC trial team**

[1]Immunomodulation and Vaccines Programme, Medical Research Council/Uganda Virus Research Institute and London School of Hygiene and Tropical Medicine (MRC/UVRI and LSHTM) Uganda Research Unit, Entebbe, Uganda

[2]Vector Control Division, Ministry of Health, Kampala, Uganda

[3]Department of Clinical Research, London School of Hygiene and Tropical Medicine, London, United Kingdom

[4]MRC Tropical Epidemiology Group, Department of Infectious Disease Epidemiology, London School of Hygiene and Tropical Medicine, London, United Kingdom

¶These authors contributed equally

***Correspondence:** Gyaviira Nkurunungi; Gyaviira.Nkurunungi@mrcuganda.org

For peer review only

Table S1. Schedule of visits and procedures

VISIT NUMBER	1	2 & 3	4	5[9]	5.2	6	7	8	9	10	11
WEEKS FROM 1ST IMMUNISATION	-8[1]	-6[10], -4, -2	0	4	4 weeks +4 days	8	20	28	32	44	52
	Screening	Treatment (Rx) only	Immunisations	Immunisations		Primary Endpoint (PE), Immunisations	Rx only	Immunisations	PE/Secondary Endpoint (SE)[2]	Rx only	SE, Immunisations
**RANDOMISED INTENSIVE VS STANDARD PRAZIQUANTEL INTERVENTION**											
PZQ intensive arm (x)		x				x[3]	x		x[3]	x	x[3]
PZQ standard arm						x[3]					x[3]
Albendazole						x[3]			x[3]		x[3]
**VACCINES**											
BCG			x								
YF-17D				x							
Ty21a				x							
HPV				x		[x][4]		x			
Td								x			[x][5]
**INVESTIGATIONS/PROCEDURES**											
Inclusion/exclusion criteria	x										
Informed consent	x										
Questionnaire	x		x	x	x	x		x	x		x
Examination	x		(x)	(x)	(x)	(x)		(x)	(x)		(x)
Urine β-HCG test (female only) 1mL	x		x	x				x			
Urine YF viral load					x						
Stool for PCR and storage	x							x			x
Stool for coproantibody and storage	x					x					
**BLOOD SAMPLES**											
Malaria PCR (1ml)			x	x				x			x
Serology for HIV, prior malaria and *S. mansoni* (0.5ml)	x										
Mansonella perstans (1ml)	x										
Serum/plasma CAA (1ml)	x		x	x		x		x	x		x
Hb[8] / Full blood count (0.5ml)	x		x	x				x			
Assessments of pre-immunisation responses, and/or vaccine response outcomes and/or exploratory immunology; storage[7] (10 – 20 mls)			x	x		x		x	x		x
Blood for gene expression (2mls)			x	x				x			
Blood vol (mL)	4		27	17		10-20		27	10		14
Cumulative blood vol (mL)[7]	4		31	48		68		95	105		119

For peer review only - http://bmjopen.bmj.com/site/about/guidelines.xhtml

PE: primary endpoint; **SE:** secondary endpoint; **Rx only**: treatment only
Immunisation days highlighted in green, primary end point days in red, days for treatment only in grey
(**x**) performed if clinically indicated

1. Screening and enrolment into Project A will take place about 8 weeks before immunisation 0 to allow initiation of the praziquantel intervention.
2. Week 32 is primary endpoint for responses to Td given at 28 weeks; secondary endpoint for HPV booster
3. Treatments given after sampling when schedules coincide
4. Week 8 HPV dose will be given for previously-unvaccinated girls aged $\geq$14 years
5. Week 52 Td booster dose will be provided as a service
6. Exploratory immunology blood volume will be guided by guidelines from Harvard Mass General, where a maximum of 3ml/kg body weight is taken at any one time point and not more than 3ml/kg is taken over any 8-week period (ref http://www.drgreene.com/21_1616.html.) These guidelines have been followed in a previous study vaccinating adolescents with investigational tuberculosis vaccine MVA85A (in Uganda).[1] The total blood volume planned is 68 ml over the initial intensive sampling period of 8 weeks. Revision of sample volumes based on weight will only be required for participants who weigh less than 22 kg; the average weight of children aged 9 years is expected to be 28kg (with 21kg the 3rd centile) with greater weights for older children.[2]
7. At baseline, it will only be Hb estimation by Haemocue
8. Oral typhoid vaccine doses will be administered on three alternate days namely visit 5, 5.1 and 5.2.
9. The first PZQ treatment at week -6 will be administered at the end of the screening visit

22

For peer review only

For peer review only - http://bmjopen.bmj.com/site/about/guidelines.xhtml

Further information on recruitment criteria

- Participants who are excluded from the trial because they have been discovered (during screening procedures) to be suffering from a previously undiagnosed condition thought to require further medical attention will be referred appropriately for further investigation and treatment.
- Participants discovered to have severe anaemia will be excluded from the trial and treated for anaemia
- Participants discovered to be HIV-positive will be counselled and offered a CD4 T-cell count and referred to a provider of antiretroviral treatment ("Test and Treat" – i.e. initiation of treatment regardless of CD4 count is recommended for these high-risk communities).
- Participants discovered to be pregnant will be counselled and referred to an antenatal clinic of their choice.

This trial proposes to recruit all participants within a short time-frame. It will not, therefore, be possible to reconsider enrolment of potential participants with temporary exclusion criteria after treatment and resolution of the condition.

Further rationale for the selection of vaccines

Bacillus Calmette–Guérin (BCG)

BCG is a live, replicating parenteral vaccine, and the only licensed vaccine against TB. The BCG vaccine for these studies will be obtained from the Serum Institute of India either directly, or through a supplier in Uganda. The Serum Institute of India provides much of the BCG vaccine used in Uganda.

Worldwide, TB is among the top 10 causes of death; Uganda has an estimated incidence of 202/100,000 people.[3] Infectious, sputum positive, pulmonary TB classically emerges in adolescence, driving the on-going epidemic.[4] Thus adolescent booster immunisation is a key TB control strategy.[5] However, BCG vaccine response and efficacy are often impaired in tropical and rural settings[6-8] and new TB vaccines are similarly affected.[9] In the past, the WHO has been hesitant to recommend BCG re-vaccination. However, in 2017 WHO's Strategic Advisory Group of Experts (SAGE) recommended: "Further research is warranted to explore whether certain sub-groups of age, geographic or *M. tuberculosis* exposure categories would benefit from re-vaccination."[10] Recent results suggest that, despite the variability of BCG efficacy between populations, BCG vaccination in adolescence offers benefit in some tropical settings, especially for individuals who are not yet infected with *Mycobacterium tuberculosis*, and may also be cost-effective.[7 11] Also, BCG vaccine is currently being used among adolescents in South Africa as a comparator in a trial of a novel TB vaccine (trial

For peer review only

registration NCT02075203). BCG efficacy in Ugandan adolescents, and differences in BCG vaccine responses between urban and rural Ugandan populations, have not been tested. Information obtained from this study is expected to further inform the use of BCG in adolescents, and also to inform the development of new vaccines for tuberculosis.

Yellow fever vaccine

Yellow fever vaccine YF-17D is a live replicating parenteral vaccine. The vaccine (Stamaril; Sanofi Pasteur) is available for purchase in Uganda. Yellow Fever (YF) causes outbreaks in Uganda and the wider region[12] and YF-17D is a candidate for Uganda's expanded programme on immunisation (EPI). Lower vaccine replication, lower neutralising antibody induction, and greater waning, are described in Uganda compared to Switzerland.[13] YF-17D is a potential vector for novel vaccine constructs,[14] adding relevance to vaccine development.

Typhoid vaccine Ty21a

Typhoid vaccine Ty21a is a live replicating oral vaccine and also a potential vector for new vaccine constructs.[15] The Ty21a vaccine will be purchased from PaxVax, Redwood City, California. Substantial, multi-year typhoid outbreaks occur in Uganda and immunisation campaigns have been advocated as cost effective.[16] Schistosomiasis has been associated with prolonged *S. typhi* infection[17] and impaired antibody responses to killed typhoid vaccines.[18]

Ty21a was developed in the 1970s. Although not routinely used in Uganda, it has been (and is currently) registered in many countries. It was first registered in the United States and United Kingdom in the 1980s, and is recommended by the WHO for both endemic and epidemic settings.[19] It has comparable efficacy to the parenteral Vi polysaccharide typhoid vaccine, good durability and minimal adverse effects.[19] It is proposed for use in this study to model effects of study exposures and intervention on the response to a live oral vaccine.

The Ty21a vaccine is given as a three-dose regimen on alternate days.

Human Papilloma Virus (HPV) vaccine

The Human Papilloma Virus (HPV) vaccine is a protein virus-like particle. The quadrivalent HPV Vaccine Gardasil (Merck) is available for purchase in Uganda and is the vaccine used by the national EPI programme. Studies after three vaccine doses have found somewhat enhanced responses in the presence of malaria, but no effect of helminths.[20] No study has previously investigated parasite effects on the priming response, but recent results for tetanus suggest that priming may be more susceptible than boosting to adverse effects.[21] This will be important if forthcoming trials support single-dose HPV immunisation (NCT02834637). HPV immunisation is being rolled out among girls to

For peer review only - http://bmjopen.bmj.com/site/about/guidelines.xhtml

prevent cervical neoplasia, the most common cancer among Ugandan women and we will coordinate provision with the national HPV immunisation programme.[22] HPV immunisation is also beneficial for boys since HPV infection is associated with anogenital warts, anal cancer and oropharyngeal cancers in both males and females, and with penile cancer in men,[23] and we will include boys in these studies.

Tetanus and diphtheria vaccines

Tetanus and diphtheria (Td) vaccines comprise inert toxoids. Schistosomiasis is associated with a Th2 biased response to tetanus toxoid[24] and with suppressed antibody responses among those with low pre-immunisation antibody levels.[21] Booster immunisation is recommended for young women to prevent maternal and neonatal tetanus. Recent evidence emphasises the need to protect young men also.[25]

Immunisation Postponement Criteria

If any one of the following is identified at the time scheduled for immunisation, the participant may be immunised at a later date, or withdrawn, at the discretion of the Investigator. The participant must be followed until resolution of the event as with any adverse event:

- Acute disease at the time of immunisation. Acute disease is defined as the presence of a moderate or severe illness with or without fever. All vaccines can be administered to persons with a minor illness such as diarrhoea or mild upper respiratory infection with or without low-grade fever, i.e. temperature of ≤37.5°C (99.5°F)
- Temperature of >37.5°C (99.5°F) at the time of immunisation
- Taking antibiotics or antimalarials currently, or within the past 7 days, of the date of Ty21a administration (ascertained verbally)

Vaccine storage and transport

In order to maintain a reliable vaccine cold chain, the vaccines and diluents to be used will be stored and transported within the recommended temperature range of +2°C to +8°C. Care will be taken to ensure that the vaccines are not frozen. BCG, being sensitive to light, will be kept in the dark (normally within its secondary packaging) for as long as possible to protect it during storage and transportation. All vaccines will be kept in appropriate refrigeration equipment with a temperature monitoring device to ensure temperatures remain between +2°C and +8°C. Cold boxes/vaccines carriers with temperature monitors will be used to transport vaccines and the diluents from the MRC/UVRI and LSHTM Uganda Research Unit (Entebbe) to Koome island and while transporting vaccines to immunisation sessions. Designated staff will be given responsibility for managing the

For peer review only - http://bmjopen.bmj.com/site/about/guidelines.xhtml

vaccine cold chain. All cold chain equipment including the temperature monitoring devices used for this project will comply with relevant technical specifications as defined by the EPI standards. Basic routine maintenance will be regularly carried out on all cold chain equipment.

Additional laboratory measurements

Additional assays will comprise HIV serology, pregnancy testing and full blood counts. HIV testing and pregnancy testing will be accompanied by appropriate counselling by trained staff.

- HIV serology will be done on blood samples using rapid tests and according to prevailing national algorithms.[26] This will be done at baseline.
- Pregnancy testing will be done using urine samples and standard operating procedures for assessment of urine β-human chorionic gonadotropin (βhCG). This will be done at baseline and before immunisation on each immunisation day.
- Full blood counts will be conducted using a haematology analyser. Mild, moderate and severe anaemia will be defined according to WHO guidelines, by age.[27] This will be done at baseline to test for anaemia as part of the eligibility assessment, and pre-immunisation as part of the assessment of immunological profile.

Individuals found to be HIV positive or pregnant will be referred to appropriate providers for further care. Individuals with severe anaemia (haemoglobin <82g/L) will be excluded from the randomised intervention (since the intervention might be beneficial in management of anaemia). They will be treated for anaemia.

Sample handling and archive

Blood and other samples will be processed according to local laboratory standard operating procedures (SOPs). All samples will reach the laboratory in anonymised form.

A sample archive will be developed. Although our current programme of work will address specific hypotheses regarding pathways of effects of parasites and interventions, the sample archive will provide a major asset for exploration of new leads arising from this work, or for an alternative, "systems biology" approach employing (for example) proteomic, genomic, epigenetic and transcriptomic analyses, and investigating the microbiome and virome. Information provided to participants, and consent forms, will include considerations of sample storage, and the possibility of sample analysis in laboratories within and outside Uganda. Participants will be able to decide if they will permit such future use of any leftover samples. We plan to store the samples for up to 20 years. If further storage is needed after that time, permission will be requested from the Uganda Virus Research Institute and London School of Hygiene and Tropical Medicine ethical review committees.

For peer review only - http://bmjopen.bmj.com/site/about/guidelines.xhtml

If they elect not to permit this, all of those leftover samples will be discarded after the completion of the work included in the current protocol.

Operational considerations

Programme governance

A Programme Steering Committee has been set up to guide progress across all projects. This comprises the following:

- An independent chair
- Representatives from the Ministry of Health programmes for immunisation and for vector borne disease control
- Representatives of district authorities (Mukono and Jinja districts)
- Community representatives
- Principal investigator and co-investigators
- Project leader and post-doctoral immunologist
- Trial statistician
- Laboratory manager
- Medical Research Council observer

Informed consent

Both written informed assent from the participants and written informed consent from a parent or guardian will be required for participation, although these may not necessarily be obtained at the same time. Information will be provided in both English and the appropriate local language. For individuals who cannot speak the languages used, or who cannot read or write, a witness who can read the information sheet and translate the information to the participant or parent/guardian will be used. Two different types of age specific assent forms will be used for the group of participants aged 9 – 12 years and for the group aged 13 – 17 years. Informed consent by emancipated or mature minors will be obtained using a designated consent form for these categories of participants.

The aims of the study, all tests, treatments and immunisations to be carried out and potential risks will be explained. The participant will be given the opportunity to ask about details of the trial, and will then have time to consider whether or not to participate. If they do decide to participate, they and their parent/guardian will sign and date two copies of the assent and consent forms, one for them to take away and keep, and one to be stored securely by the research team. Separate information and consent forms will be provided (i) for consent for storage of samples for future

For peer review only

For peer review only - http://bmjopen.bmj.com/site/about/guidelines.xhtml

studies and for anonymous sharing of data from this study and (ii) for possible genetic studies; the information sheet will explain that these data may be used in analyses related to this protocol.

Screening and Eligibility Assessment

Once the informed consent process has been completed, and consent (and assent) given, a baseline medical history (including concomitant medication) will be collected. Vital signs will be checked and a physical examination will be performed. Inclusion and exclusion criteria will be checked.

Participants will undergo pre- and post-test counselling for HIV and (for girls) pregnancy testing by a trained and experienced nurse- or clinician-counsellor. Blood, urine and stool samples will be obtained, for tests as specified in the schedule of procedures. These tests are to exclude the major, immunomodulating co-infection, HIV, and conditions that might impact safety (anaemia, pregnancy).

Enrolment

Participants who consent/assent, complete the screening processes, satisfy all the inclusion criteria and meet none of the exclusion criteria will be enrolled.

Discontinuation/withdrawal criteria

In accordance with the principles of the current revision of the Declaration of Helsinki and any other applicable regulations, a participant has the right to withdraw from the study at any time and for any reason, and is not obliged to give his or her reasons for doing so. The Investigator may withdraw the participant at any time in the interests of the participant's health and well-being. In addition, the participant may withdraw/be withdrawn for any of the following reasons:

- Ineligibility (either arising during the study or retrospectively, having been overlooked at screening)
- Administrative decision by the Investigator
- Significant protocol deviation
- Participant non-compliance with study requirements
- An adverse event which requires discontinuation of the study involvement or results in inability to continue to comply with study procedures.

Any participant who becomes pregnant during the trial will be followed up until the end of the pregnancy but no further immunisations will be given unless indicated during pregnancy (as is the case for tetanus toxoid). The trial allocation for this participant will be unblinded and the participant will only be given further treatment if clinically indicated. The babies will also be followed up and

For peer review only

214 examined for any adverse effects. We will not routinely perform venepuncture in a pregnant
215 participant.

216 The reason for withdrawal will be recorded in the case report form (CRF). If withdrawal is due to an
217 AE, appropriate follow-up visits or medical care will be arranged, with the agreement of the
218 participant, until the AE has resolved, stabilised or a non-trial related causality has been assigned.

219 If a participant withdraws from the study samples collected before their withdrawal from the trial
220 will be used/ stored unless the participant specifically requests otherwise.

221 *Trial discontinuation*

222 The trial will be discontinued in the event of new scientific information that renders continuation
223 futile or unethical, or for any other reason, at the discretion of the Programme Steering Committee.

224 *End of study definition*

225 The trial will be completed when the last participant enrolled into the trial has completed their final
226 follow up visit.

227 *Safety assessments and oversight*

228 No new investigational drug or product will be used in the proposed trial. However, standard
229 approaches for monitoring safety and reporting of serious adverse events will be followed.

230 *Monitoring*

231 The trial will be monitored by both internal and external monitors according to a pre-defined
232 monitoring plan which will include a site initiation visit, monitoring visits at least annually, and a
233 close-out visit. The monitors will assess patient safety, data integrity, and adherence to the protocol
234 and to Good Clinical Research Practice procedures.

235 ***Considerations regarding standard of care***

236 *S. mansoni* infection status will be determined retrospectively through assays conducted in bulk on
237 stored samples (plasma CAA). These results will not, therefore, be useful to determine management
238 of individual participants.

239 Participants in the standard treatment arm will receive lower levels of anthelminthic treatment.
240 However, all trial arms will receive a minimum of well-implemented national standard of care.

241 Standard of care will comprise annual praziquantel treatment. Our own results from the Lake
242 Victoria Island Intervention Study on Worms and Allergy-related diseases (LaVIISWA),[28] which
243 compared annual versus quarterly intervention for schistosomiasis at community level over three

For peer review only

years, showed no advantage of quarterly treatment for morbidity outcomes attributed to schistosomiasis.

Schistosomiasis can cause anaemia. To manage the expected differential benefits of the interventions for anaemia, a full blood count will be performed at baseline, as discussed above; anaemic children will be managed appropriately and severely anaemic children excluded.

Albendazole will be provided twice a year to manage nematode infections (after collection of primary and secondary endpoint samples).

Procedures to be followed in the event of abnormal findings

Abnormal clinical findings from medical history, examination or blood tests will be assessed as to their clinical significance throughout the trials. If an abnormal test result is deemed clinically significant, it may be repeated. If a test remains clinically significant, the participant will be informed and appropriate medical care arranged as appropriate and with the permission of the participant. Specific details regarding findings, discussion with participants and resulting actions will be recorded in the clinical records. Decisions to exclude the participant from enrolling in the trial or to withdraw a participant from the trial will be at the discretion of the Investigator.

Data and Safety Monitoring Board (DSMB)

A data and safety monitoring board (DSMB) has been appointed to provide real-time safety oversight. The DSMB will be notified within 7 days of the Investigators' being aware of the occurrence of SAEs. The DSMB may recommend the Investigators to place the trial on hold if deemed necessary following an intervention-related SAE. The DSMB will be chaired by a clinician experienced in clinical trials. There will be a minimum of two other appropriately qualified committee members. In the case of events related to a blinded intervention, the DSMB can request unblinding. Membership will include a statistician, and at least one Ugandan member. All correspondence between Investigators and the DSMB will be conveyed by the Principal Investigator to the trial Sponsor. The Chair of the DSMB will be contacted for advice and independent review by the Investigator or trial Sponsor in the following situations:

- The occurrence of any SAE
- Any other situation where the Investigator or trial Sponsor feels independent advice or review is important

Ethical and regulatory considerations

Further information regarding risks

For peer review only

The immunisations to be given have recognised side effects which are usually mild and resolve spontaneously in a few days to one week. Parenteral vaccines are likely to result in pain and swelling at the site of injection and mild fever; very occasionally pain and swelling can be severe and associated with difficulty in moving the shoulder. Sometimes headache and tiredness occur. Rarely a vaccine may cause a severe allergic reaction. For most vaccines this is estimated at less than one in a million doses (but 1 in 55,000 for Yellow Fever vaccine).[29] Individuals with a history of a possible allergic reaction to drugs or vaccines, or to vaccine components including eggs or chicken proteins, will be excluded from the studies. The research team will be trained and prepared to manage severe allergic reactions.

Adverse reactions to Yellow Fever vaccine include severe nervous system reaction (about 1 person in 125,000) and severe, life-threatening illness with organ failure (about 1 person in 250,000). The mortality for this severe, life-threatening adverse effect is reported as about 50%.[29]

BCG immunisation is likely to induce a scar in many cases. This may develop over several weeks, starting as a small papule at the injection site which may become ulcerated and then heal over a period of 2 to 5 months; and lymphadenopathy may develop. Occasionally a more severe local reaction occurs (estimated at 1 per 1,000-10,000 doses): for example, an abscess develops and scars may develop into keloids. Rarely BCG can cause disseminated disease (1 per 230,000 to 640,000 doses), or disease in sites remote from the immunisation site. Disseminated BCG disease usually occurs in immunocompromised people: HIV positive people will be excluded from these studies.[30] BCG “pre-immunisation” may interfere with the response to the subsequent live vaccines; indeed our hypothesis, and published results, suggest that it may suppress replication of YF 17D vaccine.[31] However, this reduced replication has not been shown to correlate with, or result in, reduced levels of neutralising antibody titres (which are the desired protective outcome).[13 31]

Oral typhoid vaccine (Ty21a) may occasionally be associated with stomach pain, nausea, vomiting and (rarely) rash.[29]

Praziquantel has been in use for about 30 years. It has a well-recognised profile of side effects including dizziness, nausea, vomiting, abdominal pain, diarrhoea (sometimes with blood) and urticarial rash. The symptoms are considered to arise largely from the effects of killing worms and to be more severe in people with heavy infections. Symptoms are better tolerated when the drugs are given after food and we will provide treatment after a meal or snack. Simple medications, such as paracetamol and cetirizine, can alleviate symptoms and these will be available on treatment days.

For peer review only - http://bmjopen.bmj.com/site/about/guidelines.xhtml

References

1. Wajja A, Kizito D, Nassanga B, et al. The effect of current Schistosoma mansoni infection on the immunogenicity of a candidate TB vaccine, MVA85A, in BCG-vaccinated adolescents: An open-label trial. *PLoS neglected tropical diseases* 2017;11(5):e0005440. doi: 10.1371/journal.pntd.0005440 [published Online First: 2017/05/05]

2. WHO. Growth reference 5-19 years. 2007

3. WHO. Global tuberculosis report 2016 http://www.who.int/tb/publications/global_report/en/ (accessed 17 June 2017), 2016.

4. Alcais A, Fieschi C, Abel L, et al. Tuberculosis in children and adults: two distinct genetic diseases. *J Exp Med* 2005;202(12):1617-21. doi: 10.1084/jem.20052302

5. Weiner J, 3rd, Kaufmann SH. Recent advances towards tuberculosis control: vaccines and biomarkers. *J Intern Med* 2014;275(5):467-80. doi: 10.1111/joim.12212

6. Fine PE. Variation in protection by BCG: implications of and for heterologous immunity. *Lancet (London, England)* 1995;346(8986):1339-45.

7. Barreto ML, Pilger D, Pereira SM, et al. Causes of variation in BCG vaccine efficacy: examining evidence from the BCG REVAC cluster randomized trial to explore the masking and the blocking hypotheses. *Vaccine* 2014;32(30):3759-64. doi: 10.1016/j.vaccine.2014.05.042

8. Black GF, Weir RE, Floyd S, et al. BCG-induced increase in interferon-gamma response to mycobacterial antigens and efficacy of BCG vaccination in Malawi and the UK: two randomised controlled studies. *Lancet (London, England)* 2002;359(9315):1393-401. doi: 10.1016/S0140-6736(02)08353-8

9. Tanner R, Kakalacheva K, Miller E, et al. Serum indoleamine 2,3-dioxygenase activity is associated with reduced immunogenicity following vaccination with MVA85A. *BMC infectious diseases* 2014;14:660. doi: 10.1186/s12879-014-0660-7 [published Online First: 2014/12/04]

10. WHO. SAGE Evidence to recommendations framework. 2017. http://www.who.int/immunization/sage/meetings/2017/october/2_EvidencetoRecommendationFramework_BCG.pdf (accessed 16th March 2018).

11. Dye C. Making wider use of the world's most widely used vaccine: Bacille Calmette-Guerin revaccination reconsidered. *Journal of the Royal Society, Interface* 2013;10(87):20130365. doi: 10.1098/rsif.2013.0365 [published Online First: 2013/08/02]

12. Kraemer MU, Faria NR, Reiner RC, Jr., et al. Spread of yellow fever virus outbreak in Angola and the Democratic Republic of the Congo 2015-16: a modelling study. *The Lancet Infectious diseases* 2016 doi: 10.1016/s1473-3099(16)30513-8 [published Online First: 2016/12/27]

13. Muyanja E, Ssemaganda A, Ngauv P, et al. Immune activation alters cellular and humoral responses to yellow fever 17D vaccine. *The Journal of clinical investigation* 2014;124(7):3147-58. doi: 10.1172/jci75429 [published Online First: 2014/06/10]

14. Aguiar M, Stollenwerk N, Halstead SB. The Impact of the Newly Licensed Dengue Vaccine in Endemic Countries. *PLoS neglected tropical diseases* 2016;10(12):e0005179. doi: 10.1371/journal.pntd.0005179 [published Online First: 2016/12/22]

15. Dharmasena MN, Osorio M, Filipova S, et al. Stable expression of Shigella dysenteriae serotype 1 O-antigen genes integrated into the chromosome of live Salmonella oral vaccine vector Ty21a. *Pathogens and disease* 2016 doi: 10.1093/femspd/ftw098 [published Online First: 2016/09/23]

For peer review only - http://bmjopen.bmj.com/site/about/guidelines.xhtml

16. Carias C, Walters MS, Wefula E, et al. Economic evaluation of typhoid vaccination in a prolonged typhoid outbreak setting: the case of Kasese district in Uganda. *Vaccine* 2015;33(17):2079-85. doi: 10.1016/j.vaccine.2015.02.027 [published Online First: 2015/02/26]

17. Melhem RF, LoVerde PT. Mechanism of interaction of Salmonella and Schistosoma species. *Infection and immunity* 1984;44(2):274-81. [published Online First: 1984/05/01]

18. Muniz-Junqueira MI, Tavares-Neto J, Prata A, et al. Antibody response to Salmonella typhi in human schistosomiasis mansoni. *Revista da Sociedade Brasileira de Medicina Tropical* 1996;29(5):441-5. [published Online First: 1996/09/01]

19. WHO. Position Paper on Typhoid vaccines: WHO position paper – March 2018 2018

20. Brown J, Baisley K, Kavishe B, et al. Impact of malaria and helminth infections on immunogenicity of the human papillomavirus-16/18 AS04-adjuvanted vaccine in Tanzania. *Vaccine* 2014;32(5):611-7. doi: 10.1016/j.vaccine.2013.11.061

21. Riner DK, Ndombi EM, Carter JM, et al. Schistosoma mansoni Infection Can Jeopardize the Duration of Protective Levels of Antibody Responses to Immunizations against Hepatitis B and Tetanus Toxoid. *PLoS neglected tropical diseases* 2016;10(12):e0005180. doi: 10.1371/journal.pntd.0005180

22. Centre HI. HPV and related diseases report: Uganda. 2016. http://www.hpvcentre.net/statistics/reports/UGA.pdf (accessed 20.01.2017).

23. WHO. Human papillomavirus vaccines: WHO position paper, May 2017. *Releve epidemiologique hebdomadaire* 2017;92(19):241-68. [published Online First: 2017/05/23]

24. Sabin EA, Araujo MI, Carvalho EM, et al. Impairment of tetanus toxoid-specific Th1-like immune responses in humans infected with Schistosoma mansoni. *The Journal of infectious diseases* 1996;173(1):269-72. [published Online First: 1996/01/01]

25. Nanteza B, Galukande M, Aceng J, et al. The burden of tetanus in Uganda. *SpringerPlus* 2016;5(1):705. doi: 10.1186/s40064-016-2309-z [published Online First: 2016/06/29]

26. Ministry of Health–Uganda. Uganda Clinical Guidelines 2016. http://apps.who.int/medicinedocs/documents/s23532en/s23532en.pdf

27. WHO. Haemoglobin concentrations for the diagnosis of anaemia and assessment of severity. Vitamin and Mineral Nutrition Information System. Geneva, World Health Organization, 2011 (WHO/NMH/NHD/MNM/11.1).

28. Sanya RE, Nkurunungi G, Hoek Spaans R, et al. The Impact of Intensive Versus Standard Anthelminthic Treatment on Allergy-related Outcomes, Helminth Infection Intensity, and Helminth-related Morbidity in Lake Victoria Fishing Communities, Uganda: Results From the LaVIISWA Cluster-randomized Trial. *Clinical Infectious Diseases* 2018:ciy761-ciy61. doi: 10.1093/cid/ciy761

29. CDC. Centers for Disease Control and Prevention, vaccines and immunizations.

30. WHO. Information sheet observed rate of vaccine reactions Bacille Calmette-Guérin (BCG) vaccine. 2012

31. Arts RJW, Moorlag S, Novakovic B, et al. BCG Vaccination Protects against Experimental Viral Infection in Humans through the Induction of Cytokines Associated with Trained Immunity. *Cell host & microbe* 2018;23(1):89-100.e5. doi: 10.1016/j.chom.2017.12.010 [published Online First: 2018/01/13]

For peer review only

Reporting checklist for protocol of a clinical trial.

Based on the SPIRIT guidelines.

Instructions to authors

Complete this checklist by entering the page numbers from your manuscript where readers will find each of the items listed below.

Your article may not currently address all the items on the checklist. Please modify your text to include the missing information. If you are certain that an item does not apply, please write "n/a" and provide a short explanation.

Upload your completed checklist as an extra file when you submit to a journal.

In your methods section, say that you used the SPIRITreporting guidelines, and cite them as:

Chan A-W, Tetzlaff JM, Altman DG, Laupacis A, Gøtzsche PC, Krleža-Jerić K, Hróbjartsson A, Mann H, Dickersin K, Berlin J, Doré C, Parulekar W, Summerskill W, Groves T, Schulz K, Sox H, Rockhold FW, Rennie D, Moher D. SPIRIT 2013 Statement: Defining standard protocol items for clinical trials. Ann Intern Med. 2013;158(3):200-207

		Reporting Item	Page Number
**Administrative information**			
Title	#1	Descriptive title identifying the study design, population, interventions, and, if applicable, trial acronym	1
Trial registration	#2a	Trial identifier and registry name. If not yet registered, name of intended registry	2
Trial registration: data set	#2b	All items from the World Health Organization Trial Registration Data Set	n/a
Protocol version	#3	Date and version identifier	Information available at ISRCTN60517191
Funding	#4	Sources and types of financial, material, and other support	15
Roles and	#5a	Names, affiliations, and roles of protocol	15

For peer review only

responsibilities: contributorship		contributors	
Roles and responsibilities: sponsor contact information	#5b	Name and contact information for the trial sponsor	Information available at ISRCTN60517191
Roles and responsibilities: sponsor and funder	#5c	Role of study sponsor and funders, if any, in study design; collection, management, analysis, and interpretation of data; writing of the report; and the decision to submit the report for publication, including whether they will have ultimate authority over any of these activities	16
Roles and responsibilities: committees	#5d	Composition, roles, and responsibilities of the coordinating centre, steering committee, endpoint adjudication committee, data management team, and other individuals or groups overseeing the trial, if applicable (see Item 21a for data monitoring committee)	Information detailed in supplementary information file
**Introduction**			
Background and rationale	#6a	Description of research question and justification for undertaking the trial, including summary of relevant studies (published and unpublished) examining benefits and harms for each intervention	4
Background and rationale: choice of comparators	#6b	Explanation for choice of comparators	8
Objectives	#7	Specific objectives or hypotheses	6
Trial design	#8	Description of trial design including type of trial (eg, parallel group, crossover, factorial, single group), allocation ratio, and framework (eg, superiority, equivalence, non-inferiority, exploratory)	7
**Methods: Participants, interventions, and outcomes**			

For peer review only

Study setting	#9	Description of study settings (eg, community clinic, academic hospital) and list of countries where data will be collected. Reference to where list of study sites can be obtained	7
Eligibility criteria	#10	Inclusion and exclusion criteria for participants. If applicable, eligibility criteria for study centres and individuals who will perform the interventions (eg, surgeons, psychotherapists)	7
Interventions: description	#11a	Interventions for each group with sufficient detail to allow replication, including how and when they will be administered	8
Interventions: modifications	#11b	Criteria for discontinuing or modifying allocated interventions for a given trial participant (eg, drug dose change in response to harms, participant request, or improving / worsening disease)	Detailed in supplementary information file
Interventions: adherance	#11c	Strategies to improve adherence to intervention protocols, and any procedures for monitoring adherence (eg, drug tablet return; laboratory tests)	Detailed in supplementary information file
Interventions: concomitant care	#11d	Relevant concomitant care and interventions that are permitted or prohibited during the trial	Detailed in supplementary information file
Outcomes	#12	Primary, secondary, and other outcomes, including the specific measurement variable (eg, systolic blood pressure), analysis metric (eg, change from baseline, final value, time to event), method of aggregation (eg, median, proportion), and time point for each outcome. Explanation of the clinical relevance of chosen efficacy and harm outcomes is strongly recommended	10
Participant timeline	#13	Time schedule of enrolment, interventions (including any run-ins and washouts), assessments, and visits for participants. A schematic diagram is highly recommended (see Figure)	14<br>Also detailed in supplementary information file, Table S1

For peer review only

Sample size	#14	Estimated number of participants needed to achieve study objectives and how it was determined, including clinical and statistical assumptions supporting any sample size calculations	11
Recruitment	#15	Strategies for achieving adequate participant enrolment to reach target sample size	Detailed in supplementary information file
**Methods: Assignment of interventions (for controlled trials)**			
Allocation: sequence generation	#16a	Method of generating the allocation sequence (eg, computer-generated random numbers), and list of any factors for stratification. To reduce predictability of a random sequence, details of any planned restriction (eg, blocking) should be provided in a separate document that is unavailable to those who enrol participants or assign interventions	8
Allocation concealment mechanism	#16b	Mechanism of implementing the allocation sequence (eg, central telephone; sequentially numbered, opaque, sealed envelopes), describing any steps to conceal the sequence until interventions are assigned	9
Allocation: implementation	#16c	Who will generate the allocation sequence, who will enrol participants, and who will assign participants to interventions	8, 9
Blinding (masking)	#17a	Who will be blinded after assignment to interventions (eg, trial participants, care providers, outcome assessors, data analysts), and how	9
Blinding (masking): emergency unblinding	#17b	If blinded, circumstances under which unblinding is permissible, and procedure for revealing a participant's allocated intervention during the trial	Detailed in supplementary information file
**Methods: Data collection, management, and analysis**			

For peer review only

Data collection plan	#18a	Plans for assessment and collection of outcome, baseline, and other trial data, including any related processes to promote data quality (eg, duplicate measurements, training of assessors) and a description of study instruments (eg, questionnaires, laboratory tests) along with their reliability and validity, if known. Reference to where data collection forms can be found, if not in the protocol	10.<br><br>More details in supplementary information file
Data collection plan: retention	#18b	Plans to promote participant retention and complete follow-up, including list of any outcome data to be collected for participants who discontinue or deviate from intervention protocols	Detailed in supplementary information file
Data management	#19	Plans for data entry, coding, security, and storage, including any related processes to promote data quality (eg, double data entry; range checks for data values). Reference to where details of data management procedures can be found, if not in the protocol	13.<br><br>More information in the statistical analysis plan found at ISRCTN60517191
Statistics: outcomes	#20a	Statistical methods for analysing primary and secondary outcomes. Reference to where other details of the statistical analysis plan can be found, if not in the protocol	13.<br><br>More information in the statistical analysis plan found at ISRCTN60517191
Statistics: additional analyses	#20b	Methods for any additional analyses (eg, subgroup and adjusted analyses)	11.<br><br>More information in the statistical analysis plan found at ISRCTN60517191
Statistics: analysis population and missing data	#20c	Definition of analysis population relating to protocol non-adherence (eg, as randomised analysis), and any statistical methods to handle missing data (eg, multiple imputation)	Information in the statistical analysis plan found at ISRCTN60517191

Methods: Monitoring

For peer review only

Data monitoring: formal committee	#21a	Composition of data monitoring committee (DMC); summary of its role and reporting structure; statement of whether it is independent from the sponsor and competing interests; and reference to where further details about its charter can be found, if not in the protocol. Alternatively, an explanation of why a DMC is not needed	Detailed in supplementary information file
Data monitoring: interim analysis	#21b	Description of any interim analyses and stopping guidelines, including who will have access to these interim results and make the final decision to terminate the trial	Detailed in supplementary information file
Harms	#22	Plans for collecting, assessing, reporting, and managing solicited and spontaneously reported adverse events and other unintended effects of trial interventions or trial conduct	Detailed in supplementary information file
Auditing	#23	Frequency and procedures for auditing trial conduct, if any, and whether the process will be independent from investigators and the sponsor	Detailed in supplementary information file
**Ethics and dissemination**			
Research ethics approval	#24	Plans for seeking research ethics committee / institutional review board (REC / IRB) approval	12
Protocol amendments	#25	Plans for communicating important protocol modifications (eg, changes to eligibility criteria, outcomes, analyses) to relevant parties (eg, investigators, REC / IRBs, trial participants, trial registries, journals, regulators)	11
Consent or assent	#26a	Who will obtain informed consent or assent from potential trial participants or authorised surrogates, and how (see Item 32)	12.<br>Also detailed in supplementary information file
Consent or assent: ancillary studies	#26b	Additional consent provisions for collection and use of participant data and biological specimens in ancillary studies, if applicable	Detailed in supplementary information file

For peer review only

Confidentiality	#27	How personal information about potential and enrolled participants will be collected, shared, and maintained in order to protect confidentiality before, during, and after the trial	Detailed in supplementary information file
Declaration of interests	#28	Financial and other competing interests for principal investigators for the overall trial and each study site	15
Data access	#29	Statement of who will have access to the final trial dataset, and disclosure of contractual agreements that limit such access for investigators	n/a
Ancillary and post trial care	#30	Provisions, if any, for ancillary and post-trial care, and for compensation to those who suffer harm from trial participation	Detailed in supplementary information file
Dissemination policy: trial results	#31a	Plans for investigators and sponsor to communicate trial results to participants, healthcare professionals, the public, and other relevant groups (eg, via publication, reporting in results databases, or other data sharing arrangements), including any publication restrictions	2, 13
Dissemination policy: authorship	#31b	Authorship eligibility guidelines and any intended use of professional writers	n/a
Dissemination policy: reproducible research	#31c	Plans, if any, for granting public access to the full protocol, participant-level dataset, and statistical code	13
**Appendices**			
Informed consent materials	#32	Model consent form and other related documentation given to participants and authorised surrogates	Supplementary files provided
Biological specimens	#33	Plans for collection, laboratory evaluation, and storage of biological specimens for genetic or molecular analysis in the current trial and for future use in ancillary studies, if applicable	n/a

None The SPIRIT checklist is distributed under the terms of the Creative Commons Attribution License CC-BY-ND 3.0. This checklist can be completed online using https://www.goodreports.org/, a tool made by the EQUATOR Network in collaboration with Penelope.ai

For peer review only

BMJ Open

The effect of intensive treatment for schistosomiasis on immune responses to vaccines among rural Ugandan island adolescents: randomised controlled trial protocol A for the 'POPulation differences in VACcine responses' (POPVAC) programme

Journal:	*BMJ Open*
Manuscript ID	bmjopen-2020-040426.R2
Article Type:	Protocol
Date Submitted by the Author:	19-Nov-2020
Complete List of Authors:	Nkurunungi, Gyaviira; MRC/UVRI and LSHTM Uganda Research Unit, Immunomodulation and Vaccines Programme<br>Zirimenya , Ludoviko ; MRC/UVRI and LSHTM Uganda Research Unit, Immunomodulation and Vaccines Programme<br>Nassuuna , Jacent; MRC/UVRI and LSHTM Uganda Research Unit, Immunomodulation and Vaccines Programme<br>Natukunda , Agnes ; MRC/UVRI and LSHTM Uganda Research Unit, Immunomodulation and Vaccines Programme<br>Kabuubi , Prossy ; MRC/UVRI and LSHTM Uganda Research Unit, Immunomodulation and Vaccines Programme<br>Niwagaba , Emmanuel ; MRC/UVRI and LSHTM Uganda Research Unit, Immunomodulation and Vaccines Programme<br>Oduru, Gloria; MRC/UVRI and LSHTM Uganda Research Unit, Immunomodulation and Vaccines Programme<br>Kabami , Grace ; MRC/UVRI and LSHTM Uganda Research Unit, Immunomodulation and Vaccines Programme<br>Amongin , Rebecca ; MRC/UVRI and LSHTM Uganda Research Unit, Immunomodulation and Vaccines Programme<br>Mutebe , Alex ; MRC/UVRI and LSHTM Uganda Research Unit, Immunomodulation and Vaccines Programme<br>Namutebi , Milly ; MRC/UVRI and LSHTM Uganda Research Unit, Immunomodulation and Vaccines Programme<br>Zziwa , Christopher ; MRC/UVRI and LSHTM Uganda Research Unit, Immunomodulation and Vaccines Programme<br>Amongi, Susan; MRC/UVRI and LSHTM Uganda Research Unit, Immunomodulation and Vaccines Programme<br>Ninsiima , Caroline ; MRC/UVRI and LSHTM Uganda Research Unit, Immunomodulation and Vaccines Programme<br>Onen, Caroline; MRC/UVRI and LSHTM Uganda Research Unit, Immunomodulation and Vaccines Programme<br>Akello , Florence ; MRC/UVRI and LSHTM Uganda Research Unit, Immunomodulation and Vaccines Programme<br>Sewankambo, Moses; MRC/UVRI and LSHTM Uganda Research Unit, Immunomodulation and Vaccines Programme<br>Kiwanuka, Samuel; MRC/UVRI and LSHTM Uganda Research Unit, Immunomodulation and Vaccines Programme<br>Kizindo , Robert ; MRC/UVRI and LSHTM Uganda Research Unit,

	Immunomodulation and Vaccines Programme<br>Kaweesa, James; Republic of Uganda Ministry of Health, Vector Control Division<br>Cose , Stephen ; MRC/UVRI and LSHTM Uganda Research Unit, Immunomodulation and Vaccines Programme; London School of Hygiene and Tropical Medicine Department of Clinical Research<br>Webb, Emily; London School of Hygiene & Tropical Medicine, MRC Tropical Epidemiology Group, Department of Infectious Disease Epidemiology<br>Elliott, Alison ; MRC/UVRI and LSHTM Uganda Research Unit, Immunomodulation and Vaccines Programme; London School of Hygiene and Tropical Medicine Department of Clinical Research
**Primary Subject Heading**:	Infectious diseases
Secondary Subject Heading:	Epidemiology, Immunology (including allergy), Public health
Keywords:	Infection control < INFECTIOUS DISEASES, PARASITOLOGY, Public health < INFECTIOUS DISEASES, Immunology < TROPICAL MEDICINE, Epidemiology < TROPICAL MEDICINE, Paediatric infectious disease & immunisation < PAEDIATRICS

SCHOLARONE™
Manuscripts

I, the Submitting Author has the right to grant and does grant on behalf of all authors of the Work (as defined in the below author licence), an exclusive licence and/or a non-exclusive licence for contributions from authors who are: i) UK Crown employees; ii) where BMJ has agreed a CC-BY licence shall apply, and/or iii) in accordance with the terms applicable for US Federal Government officers or employees acting as part of their official duties; on a worldwide, perpetual, irrevocable, royalty-free basis to BMJ Publishing Group Ltd ("BMJ") its licensees and where the relevant Journal is co-owned by BMJ to the co-owners of the Journal, to publish the Work in this journal and any other BMJ products and to exploit all rights, as set out in our licence.

The Submitting Author accepts and understands that any supply made under these terms is made by BMJ to the Submitting Author unless you are acting as an employee on behalf of your employer or a postgraduate student of an affiliated institution which is paying any applicable article publishing charge ("APC") for Open Access articles. Where the Submitting Author wishes to make the Work available on an Open Access basis (and intends to pay the relevant APC), the terms of reuse of such Open Access shall be governed by a Creative Commons licence – details of these licences and which Creative Commons licence will apply to this Work are set out in our licence referred to above.

Other than as permitted in any relevant BMJ Author's Self Archiving Policies, I confirm this Work has not been accepted for publication elsewhere, is not being considered for publication elsewhere and does not duplicate material already published. I confirm all authors consent to publication of this Work and authorise the granting of this licence.

For peer review only

The effect of intensive treatment for schistosomiasis on immune responses to vaccines among rural Ugandan island adolescents: randomised controlled trial protocol A for the 'POPulation differences in VACcine responses' (POPVAC) programme

Gyaviira Nkurunungi[1,¶,*], Ludoviko Zirimenya[1,¶], Jacent Nassuuna[1,¶], Agnes Natukunda[1,¶], Prossy N Kabuubi[1], Emmanuel Niwagaba[1], Gloria Oduru[1], Grace Kabami[1], Rebecca Amongin[1], Alex Mutebe [1], Milly Namutebi[1], Christopher Zziwa[1], Susan Amongi[1], Caroline Ninsiima[1], Caroline Onen[1], Florence Akello[1], Moses Sewankambo[1], Samuel Kiwanuka[1], Robert Kizindo[1], James Kaweesa[2], Stephen Cose[1,3], Emily L Webb[4], Alison M Elliott[1,3] **for the POPVAC trial team**

[1]Immunomodulation and Vaccines Programme, Medical Research Council/Uganda Virus Research Institute and London School of Hygiene and Tropical Medicine (MRC/UVRI and LSHTM) Uganda Research Unit, Entebbe, Uganda

[2]Vector Control Division, Ministry of Health, Kampala, Uganda

[3]Department of Clinical Research, London School of Hygiene and Tropical Medicine, London, United Kingdom

[4]MRC Tropical Epidemiology Group, Department of Infectious Disease Epidemiology, London School of Hygiene and Tropical Medicine, London, United Kingdom

¶These authors contributed equally

***Correspondence:** Gyaviira Nkurunungi; Gyaviira.Nkurunungi@mrcuganda.org

For peer review only

Abstract

Introduction

Several licensed and investigational vaccines have lower efficacy, and induce impaired immune responses, in low-income versus high-income countries and in rural, versus urban, settings. Understanding these population differences is essential to optimising vaccine effectiveness in the tropics. We suggest that repeated exposure to and immunomodulation by chronic helminth infections partly explains population differences in vaccine response.

Methods and analysis

We have designed an individually randomised, parallel group trial of intensive versus standard praziquantel (PZQ) intervention against schistosomiasis, to determine effects on vaccine response outcomes among school-going adolescents (9 to 17 years) from rural *Schistosoma mansoni* (*Sm*)-endemic Ugandan islands. Vaccines to be studied comprise BCG on day 'zero'; yellow fever, oral typhoid and Human Papilloma Virus (HPV) vaccines at week 4; and HPV and Tetanus/diphtheria booster vaccine at week 28. The intensive arm will receive PZQ doses three times, each two weeks apart, before BCG immunisation, followed by a dose at week 8 and quarterly thereafter. The standard arm will receive PZQ at week 8 and week 52. We expect to enrol 480 participants, with 80% *Sm* infected at the outset.

Primary outcomes are BCG-specific IFN-γ ELISpot responses eight weeks after BCG immunisation and for other vaccines, antibody responses to key vaccine antigens at four weeks after immunisation. Secondary analyses will determine effects of intensive anthelminthic treatment on correlates of protective immunity, on vaccine response waning, on priming vs boosting immunisations, and on *Sm* infection status and intensity. Exploratory immunology assays using archived samples will enable assessment of mechanistic links between helminths and vaccine responses.

Ethics and dissemination

Ethics approval has been obtained from relevant Ugandan and UK ethics committees. Results will be shared with Uganda Ministry of Health, relevant district councils, community leaders and study participants. Further dissemination will be done through conference proceedings and publications.

Trial registration

Current Controlled Trials identifier: ISRCTN60517191.

For peer review only

For peer review only - http://bmjopen.bmj.com/site/about/guidelines.xhtml

Article summary

Strengths and limitations of this study

- This will be the first adequately powered intervention study to investigate effects of schistosomiasis treatment on vaccine responses in adolescents.
- Effects on both live-attenuated and inert vaccines will be studied.
- Our strong immunoepidemiological design and nested immunological studies will address specific hypotheses regarding pathways of effects.
- The sample archives developed will provide a major asset for exploration of new leads arising from this hypothesis-driven work, or for an alternative, "systems biology" approach investigating (for example) transcriptome, microbiome and virome.
- Even with intensive anthelminthic intervention, it may be difficult to "successfully" treat *Schistosoma* infection in our endemic setting due to re-infections; however, we still expect a substantial difference in intensity between the two trial arms.

Word count

3250

Keywords

Vaccine; Schistosomiasis; Praziquantel; Immunization

For peer review only

Introduction

Vaccine-specific immune responses are often impaired, and vaccine efficacy and effectiveness lower, in tropical low-income countries (LICs) compared to temperate high-income countries and in rural, compared to urban, LIC settings.[1-8] This has been recognised for both live vaccines (such as BCG,[2 3 5 9] polio,[1] yellow fever[4] vaccines) and non-live vaccines (such as influenza[10] and tetanus[11]). Investigational malaria[7] and viral-vectored tuberculosis[6] and Ebola[12] vaccines are also affected. Previous exposure to the target pathogen (or related organisms) may mask the benefit of the vaccine.[13 14] However, pre-vaccination exposure does not explain why Ebola trial vaccine-specific responses differ between healthy UK and Senegalese adults,[12] as the target organism is rare. Therefore, environmentally-dependent mechanisms may play an important role.[5]

A long-held hypothesis is that parasites, particularly helminths, modulate vaccine responses through profound pre- and post-immunisation bystander effects on immunological activation and regulation.[15-17] Helminths might also impact vaccines responses through interactions with the complex ecosystem of mammalian gut bacteria, fungi, protozoa and viruses (the"trans-kingdom" concept[18] detailed elsewhere in this journal [bmjopen-2020-040425]). Helminth-induced gut mucosa damage, the associated translocation of microbial products into the systemic circulation[19-21] and systemic immune activation or regulation mediated by microbial products might contribute to modulation of responses to vaccines and other infections.

Helminth-mediated modulation of vaccine responses has not been substantiated in human populations. No appropriately powered trials have been conducted to evaluate reversibility of their effects. In animal models, helminths generally impair priming and accelerate waning of vaccine responses, although effects vary with helminth species, vaccine type and the timing of infection and immunisation.[22] Most observational studies in humans also suggest suppressed or biased responses during helminth infection, especially during systemic infections, such as schistosomiasis and the filariases. There is modest evidence that treating geohelminths in humans improves responses to BCG[23 24] or oral cholera vaccine[25] and we found that schistosomiasis treatment improved the measles-booster response in pre-school children.[26] There is therefore a strong case for a comprehensive assessment of the effects of helminths and their treatment on vaccine responses.

The extent to which helminths and related "trans-kingdom" mediators causally and reversibly impact immunological characteristics associated with vaccine responses may best be determined by intervention studies. This trial protocol A of the 'Population differences in Vaccine responses" programme (POPVAC A; Current Controlled Trials identifier: ISRCTN60517191) has been designed to evaluate the effect of *Schistosoma mansoni* and its treatment on vaccine responses. This study is

For peer review only

For peer review only - http://bmjopen.bmj.com/site/about/guidelines.xhtml

101 one of three parallel trials whose designs and cross-cutting analyses are described separately in this
102 journal (bmjopen-2020-040425, bmjopen-2020-040427 and bmjopen-2020-040430).

For peer review only

For peer review only - http://bmjopen.bmj.com/site/about/guidelines.xhtml

Hypothesis

The overarching goal of the POPVAC programme is to understand population differences in vaccine responses in Uganda, in order to identify strategies through which vaccine effectiveness can be optimised for the low-income, tropical settings where they are especially needed. For this Trial A, we focus on the hypothesis that *Schistosoma mansoni infection suppresses responses to unrelated vaccines; and that this effect can be reversed, at least in part, by intensive praziquantel treatment intervention.*

Objective

To determine whether there are reversible effects of chronic *Schistosoma mansoni* infection on vaccine response in adolescents, using an intervention study.

For peer review only

For peer review only - http://bmjopen.bmj.com/site/about/guidelines.xhtml

Methods and analysis

Setting and participants

SPIRIT reporting guidelines[27] have been used. We will conduct an individually randomised, parallel group trial of intensive versus standard intervention against schistosomiasis (described below) in the *S. mansoni*-endemic Koome islands of Lake Victoria, Mukono district, Uganda.[28] We aim to enroll 480 participants, randomising 240 to each intervention arm. The study cohort will recruit participants aged 9 to 17 years in primary school years 1 to 6. Adolescents[29] in this study setting bear a heavy parasite burden.[30] In addition, this age-group is a target group for vaccines against sexually transmitted infections (currently human papilloma virus [HPV] – in future, it is hoped, for vaccines against HIV) and for booster immunisations.

Recruitment criteria

Inclusion criteria

i. Attending the selected school and planning to continue to attend the school for the duration of the study
ii. Aged 9 to 17 years and enrolled in primary 1 to 6 (to avoid primary leaving examinations in late year 7, and loss to follow up of children leaving after primary 7)
iii. Written informed assent by participant and consent by parent or guardian
iv. Females agree to avoid pregnancy for the duration of the trial
v. Willing to provide locator information and to be contacted during the course of the trial
vi. Able and willing (in the investigator's opinion) to comply with all the study requirements

Exclusion criteria

i. Clinically significant history of immunodeficiency (including HIV), cancer, cardiovascular disease, gastrointestinal disease, liver disease, renal disease, endocrine disorder and neurological illness
ii. History of serious psychiatric condition or disorder
iii. Moderate or severe acute illness characterised by any of the following symptoms: fever, impaired consciousness, convulsions, difficulty in breathing, vomiting; or as otherwise determined by the attending project clinician.
iv. Concurrent oral or systemic steroid medication or the concurrent use of other immunosuppressive agents within 2 months prior to enrolment
v. History of allergic reaction to immunisation or any allergy likely to be exacerbated by any component of the study vaccines including egg or chicken proteins

For peer review only - http://bmjopen.bmj.com/site/about/guidelines.xhtml

vi. History of previous immunisation with Yellow Fever (YF), oral typhoid or Human Papillomavirus (HPV) vaccine; previous immunisation with BCG or Tetanus and diphtheria vaccine (Td) at age ≥5 years

vii. Tendency to develop keloid scars

viii. Haemoglobin less than 82g/L

ix. Positive HIV serology

x. Positive pregnancy test

xi. Female currently lactating, confirmed pregnancy or intention to become pregnant during the trial period

xii. Use of an investigational medicinal product or non-registered drug, live vaccine, or medical device other than the study vaccines for 30 days prior to dosing with the study vaccine, or planned use during the study period

xiii. Administration of immunoglobulins and/or any blood products within the three months preceding the planned trial immunisation date

Further information on recruitment criteria can be found in Supplementary information.

Interventions

We will individually randomise participants to intensive or standard praziquantel (PZQ) treatment, in a 1:1 ratio. The intensive arm will receive three doses of PZQ (approximately 40mg/kg, assessed by height pole[31]) each two weeks apart prior to the first immunisation (the last of these 2-3 weeks before immunisation), followed by PZQ at 8 weeks (after primary endpoint sampling) and thereafter quarterly PZQ (approximately; timings adjusted to accommodate school terms) during follow up. The standard arm will receive their first dose of PZQ at week 8 (after immunisation and after primary endpoint sampling) and a second dose at week 52 (to conform to Uganda Ministry of Health policy, which is annual treatment) (**Figure 1**). No placebo will be used in this trial because all participants will be treated (albeit at different frequencies) and participants are unlikely to seek additional treatments outside the trial schedule: praziquantel treatment is not popular because of the recognised (although temporary) adverse effects (described in Supplementary information).

Randomisation and allocation to treatment arm

A randomisation code will be generated by an independent statistician using a randomly permuted block size (sizes 4, 6, 8 and 10) and used to allocate participants to either receive quarterly PZQ (intensive arm) or annual PZQ (standard arm). A set of envelopes will be prepared, labelled sequentially with the randomisation numbers and containing a card indicating the corresponding allocation (to intensive or standard treatment). The randomisation code will be kept securely by the

For peer review only

For peer review only - http://bmjopen.bmj.com/site/about/guidelines.xhtml

trial statistician and made available only to those responsible for providing or preparing the trial interventions. A second copy will be held by a data manager or statistician not otherwise involved in the trial at the MRC/UVRI and LSHTM Uganda Research Unit. At enrolment, eligibility criteria will be checked and eligible participants will be allocated sequentially to the next randomisation number until the required sample size is achieved. Randomisation implementation will be done by a clinician using the sequentially numbered opaque sealed envelopes. When the next randomisation number in the sequence is allocated, the envelope bearing that number will be opened to reveal the allocation.

Blinding

Clinicians and participants will not be blinded to the treatment allocation since they will not participate in outcome ascertainment; only immunology laboratory staff who are assessing trial outcomes will be blinded.

Immunisations

We will study a portfolio of licensed vaccines (live and inert, oral and parental, priming and boosting) expected to be beneficial (in some cases, already given) to adolescents in Uganda. Our schedule (**Table 1,** supplementary **Table S1**) will comprise three main immunisation days (week 0, week 4 and week 28). Additional HPV immunisation will be provided for girls aged 14 years or above, and a second Tetanus/diphtheria boost will be given after completion of the study, to accord with the national Expanded Programme on Immunisation (EPI) routines, but the response to these will not specifically be assessed. Further rationale for the selection of vaccines is detailed in the Supplementary information.

Table 1. Immunisation schedule

	**Immunisation week 0**	**Immunisation week 4**	**[Immunisation week 8]**	**Immunisation week 28**	**[Immunisation week 52]**
**Live vaccines**	BCG vaccination / re-vaccination[1]	Yellow fever (YF-17D)<br>Oral typhoid (Ty21a)			
**Non-live vaccines**		HPV prime[2]	HPV boost for girls aged ≥14 years[3,4]	HPV boost[2] and Tetanus/ diphtheria (Td) boost	Tetanus/ diphtheria (Td) boost[4,5]

1. Prior BCG status may vary (data on history and documentation of prior BCG, and presence of a BCG scar, will be documented although these approaches have limitations for determining BCG status)
2. Both girls and boys will receive the HPV vaccine
3. The National EPI programme recommends three doses of HPV vaccine for older girls
4. These doses will be given to comply with guidelines but outcomes specifically relating to these doses will not be assessed
5. Priming by immunisation in infancy is assumed

For peer review only - http://bmjopen.bmj.com/site/about/guidelines.xhtml

Schedule of immunisation and sampling

The schedule of immunisation and sampling is outlined in **Figure 1** and **Table S1**. Pre-immunisation vaccine responses will be assessed in baseline samples. While optimal timings for outcome measures vary between vaccines, sampling at 8 weeks post BCG and 4 weeks post YF-17D, Ty21a, HPV and Td is proposed for the primary endpoints, targeting the establishment of memory responses and approximate peak of antibody responses. A secondary endpoint at one year will assess waning. Immunisation postponement criteria are detailed in Supplementary information.

Outcomes

Primary outcomes

These will be assessed in all participants.

i. **BCG:** BCG-specific IFN-γ ELISpot response eight weeks post BCG immunisation.
ii. **YF-17D:** neutralising antibody titres (plaque-reduction neutralisation test) at four weeks post YF immunisation.
iii. **Ty21a:** *Salmonella typhi* lipopolysaccharide (LPS)-specific immunoglobulin (Ig)G concentration at four weeks post Ty21a immunisation.
iv. **HPV:** IgG specific for L1-proteins of HPV-16/18 at four weeks post HPV priming immunisation.
v. **Td:** Tetanus and diphtheria toxoid-specific IgG concentration at four weeks post Td immunisation.

Secondary outcomes

These will be assessed in all participants and will further investigate estimates of protective immunity (for vaccines where these are available) and dynamics of the vaccine responses, as well as the impact of the interventions on parasite clearance.

i. **Protective immunity.** Proportions with protective neutralising antibody (YF); protective IgG levels (TT);[32] seroconversion rates (Ty21a) at four weeks post the corresponding immunisation.
ii. **Response waning.** Primary outcome measures (all vaccines) repeated at week 52, and area-under-the curve (AUC) analyses. Parasitic infection may accelerate,[33] and anti-parasitic interventions delay, waning.
iii. **Priming versus boosting.** Effects on priming versus boosting will be examined for HPV only, comparing outcomes four weeks after the first, and four weeks after the second vaccine dose.

For peer review only - http://bmjopen.bmj.com/site/about/guidelines.xhtml

iv. **Current *S. mansoni* infection status and intensity** will be determined by serum/plasma levels of circulating anodic antigen (CAA). The method is quantitative, highly specific for *Schistosoma* infection, and much more sensitive than the conventional Kato Katz method.[34] CAA will be assessed retrospectively on stored samples collected at baseline, on immunisation days, and on primary and secondary endpoint days.

Furthermore, our sample collection will offer opportunities for an array of exploratory immunological evaluations on stored samples, focusing mainly on vaccine antigen specific outcomes. Exploratory assays will provide further detail on the role of immunological profiles and trans-kingdom effects in mediating helminth modulation of vaccine-specific responses.

Evaluation of parasite infection exposure

The following measures will also be assessed in all participants, and will be used to describe the general infection-exposure experience of the study participants.

i. **Prior exposure to schistosomiasis** will be evaluated by ELISA for IgG to schistosome egg antigen using stored blood samples collected at baseline.

ii. **The presence of other helminth infections** will be determined retrospectively using stool PCR of samples collected at baseline and at weeks 28 and 52.[30] In accordance with national guidelines, all participants will be treated with albendazole or mebendazole after collection of samples for primary endpoints at week 8 and 28, and after collection of samples for secondary endpoints at week 52.

iii. **Current malaria infection status and intensity** will be assessed retrospectively by PCR on stored samples collected on immunisation days and at week 52. Individuals presenting with fever will be investigated using rapid diagnostic tests for malaria and treated based on the results and according to prevailing national guidelines.

iv. **Prior malaria exposure** will be evaluated by ELISA for IgG to malaria antigen using stored samples collected at baseline.

Sample size considerations

Based on the literature[4 35 36] and preliminary data, we anticipate that, following log to base 10 transformations that will be applied to normalise primary outcome measures, standard deviations (SDs) of primary outcome measures will lie between 0.3 and 0.6 on this log scale, and that effective treatment may increase responses by approximately 0.2 on the log scale (based on Tweyongyere *et al.*[26]). We have therefore powered our study to detect differences of this magnitude (0.2 on the log scale) or (in some cases) smaller (**Table 2**). We assume *S. mansoni* prevalence of $\geq$80%.

For peer review only - http://bmjopen.bmj.com/site/about/guidelines.xhtml

Based on these assumptions and a two independent samples t-test, we plan to include 480 participants in total (240 quarterly PZQ, 240 annual PZQ); of whom 384 are expected to be *S. mansoni* infected,[28] giving 192 participants in each trial arm who are infected at baseline.

Table 2 shows power estimates, for 5% type-1 error rate and assuming 20% loss to follow-up.

Table 2. Power estimates (5% type-1 error rate)

**Standard deviation (log_{10})**	**Difference in mean log_{10} transformed outcome, between trial arms**						
	**0.08**	**0.10**	**0.12**	**0.14**	**0.16**	**0.18**	**0.20**
192 intensive PZQ vs 192 standard PZQ (*S. mansoni* infected only)							
0.3	65%	83%	94%	98%	>99%	>99%	>99%
0.4	42%	59%	75%	87%	94%	98%	99%
0.5	29%	42%	56%	69%	80%	88%	94%
0.6	21%	31%	42%	53%	65%	75%	83%

Cells highlighted in grey correspond to >80% power; differences in mean log10 transformed outcome of 0.08, 0.10, 0.12, 0.14, 0.16, 0.18 and 0.20 are equivalent to geometric mean ratios for untransformed outcomes of 1.20, 1.26, 1.32, 1.38, 1.45, 1.51 and 1.59, respectively.

Ethics and dissemination

Ethical approval has been granted from the Research Ethics Committee of the Uganda Virus Research Institute (UVRI REC, reference: GC/127/19/05/664) and the London School of Hygiene and Tropical Medicine (LSHTM, reference: 16032), and from the Uganda National Council for Science and Technology (UNCST, reference: HS2486) and the Uganda National Drug Authority (NDA, reference: CTA0093). Any protocol amendments will be submitted to ethics committees and regulatory bodies for approval before implementation.

Participants are adolescents and therefore a vulnerable human population. Care will be taken to provide adequate, age and education-status appropriate information and to ensure that it is understood; and to emphasise that participation is voluntary. Participants will be enrolled only when they have given their own assent and when consent has been given by the parent or guardian. Model consent and assent forms are shown in Supplementary file 2. No major risks to the participants are anticipated since all the treatments and vaccines to be given are licensed and known to be safe. The main risk to participants will be time lost from school work: we will work with teachers and parents to minimise disruption to classes, and will avoid enrolment of primary 7 students since these classes are involved in national examinations. Further risks are discussed in Supplementary information.

For peer review only

For peer review only - http://bmjopen.bmj.com/site/about/guidelines.xhtml

Study findings will be published through open access peer-reviewed journals, presentations at local, national and international conferences and to the local community through community meetings. Anonymised participant level datasets generated will be available upon request.

Patient and public involvement

Concepts involved in this work have been discussed with colleagues at the Vector Control Division and Expanded Programme on Immunisation in the Ministry of Health (Uganda), with the Mukono District Council and with community leaders and Village Health Teams from Koome subcounty. We also have held meetings to explain the proposed work to teachers, parents, participants and village members, and to address their questions about issues such as study length, the study's ethical approval status, why adults were excluded from the study, and to explain to them why boys will also receive the HPV vaccine. Study findings will be shared with these stakeholders and with participants.

Data management and analysis

Socio-demographic information and clinical and laboratory measurements will be recorded and managed using Research Electronic Data Capture (REDCap) tools,[37 38] with paper-based forms as back-up. All data will be recorded under a unique study ID number. When paper forms must be used, data will be double entered in a study-specific database, with standard checks for discrepancies. All data for analysis will be anonymised and stored on a secure and password-protected server, with access limited to essential research personnel.

Baseline characteristics including age, sex, school, location of birth, prior vaccination status, helminth infection and prior exposure status and malaria infection and prior exposure status will be summarised by trial arm. The effect of intensive (compared to standard) praziquantel treatment on the outcomes will be analysed. Information on infection status will only be available after randomisation. The primary analysis will be done on individuals identified as infected at baseline (through randomisation, these will be balanced between treatment arms); this will test the hypothesis that treating the infection (and subsequent reinfections) reverses the parasite's effects on vaccine responses. If treating *S. mansoni* reverses adverse parasite effects on vaccine responses, this may be a beneficial public health intervention. However, routine screening for parasite infection before immunisation would be laborious. Secondary analyses will include all randomised individuals; this will provide insight into the broader benefit of the interventions as public health measures. The effect of intensive versus standard praziquantel treatment on primary outcomes will be assessed using unpaired t-tests, with results presented as a mean difference in vaccine response measure together with 95% confidence interval and p-value. For all outcomes, we will investigate adjusting for corresponding baseline vaccine responses as this may improve the precision of effect estimates;

For peer review only

For peer review only - http://bmjopen.bmj.com/site/about/guidelines.xhtml

this will be done using multivariable regression. We anticipate that outcomes will be positively skewed, and will apply log transformations to normalise distributions before analysis if required. The detailed analytical plan is available on the online trial registration site (http://www.isrctn.com/ISRCTN60517191).

For peer review only

For peer review only - http://bmjopen.bmj.com/site/about/guidelines.xhtml

Discussion

This will be the first adequately powered intervention study to investigate the effect of schistosomiasis treatment on vaccine responses in adolescents. This study will determine whether *S. mansoni* infection has a causal, reversible, impact on the response to live or inert vaccines, including effects on vaccine replication, immune response profile, priming, boosting and waning. The results will add to understanding of population differences in vaccine responses and on interventions that may enhance responses. If treating helminths improves vaccine responses in adolescents, combined parasite-control/immunisation programmes offer an attractive, practical public health intervention for schools and communities.

There are risks associated with our approach to addressing the trial objective. **First**, there is a risk of failure to clear *S. mansoni* infections, and repeated reinfection during the trial. This issue can be challenging because of incomplete cure or maturation of immature worms after treatment, and lifestyles in endemic communities that result in repeated exposure. To mitigate this, we will administer three PZQ treatments over a six-week period before the first immunisations, and continuing quarterly treatment in the intensive arm. **Second,** there is a risk that *S. mansoni* infection has long-term effects, not removed by treatment, mediated, for example, by epigenetic change.[39] However, studies show that parasite treatment results in immunological changes,[40 41] and our data suggest at least partial recovery of the measles vaccine response among young children treated for schistosomiasis.[26] By initiating intervention six to eight weeks before the first immunisations, and providing repeated intervention in the intensive arms, we hope to achieve significant resolution of *S. mansoni* effects.

We are interested in the effects of removing *S. mansoni*. Treating parasites can induce acute immunological change due to release of previously hidden antigens.[42 43] To minimise such effects, immunisations will be given at least 2 weeks after PZQ (the longest practicable interval; **Figure 1**).

Laboratory analyses will also highlight immune parameters and cellular populations that link environmental exposures to vaccine responses. Identifying processes associated with poor or good outcomes will inform strategies in vaccine design (for example, the genetic modification of vaccines, or innovative use of adjuvants, to counter any adverse immunological milieu, currently an area of intense research for cancer vaccines[44]); ultimately supporting the development of effective vaccines tailored to the low-income settings that most need them.

Study timeline

For peer review only - http://bmjopen.bmj.com/site/about/guidelines.xhtml

Applications for ethical approval were submitted in May 2018, with approval received in September 2018 (Uganda Virus Research Institute Research Ethics Committee), May 2019 (National Drug Authority and Uganda National Council for Science and Technology), June 2019 (London School of Hygiene and Tropical Medicine). Collaborator/investigator/trial steering committee meetings were also held during the initial 12-month planning period. The study began recruitment in July 2019. Intervention will be up to 12 months, with completion of the project scheduled for September 2020.

Competing interests

Alison Elliott reports a grant from the Medical research Council, UK (POPVAC programme funding). The rest of the authors declare that they have no conflicts of interest.

Author contributions

AME conceived the study. AME, GN, ELW, AN, JN, SC, LZ and JK contributed to study design. LZ, GO, PNK, EN, GK, RA, CN, CO, MN, CZ, SA and FA are site clinicians/nurses/clinical laboratory technicians providing valuable input on clinical considerations of the intervention. MS, SK and RK are field workers handling the organisational integration of the intervention. AN, AM and ELW are involved in organisation of the databases, trial randomization, treatment allocation and drawing up of analytical plans. GN, LZ, JN, AN, SC, ELW and AME drafted the manuscript. All authors reviewed the manuscript, contributed to it and approved the final version.

Acknowledgements

We thank the Uganda National Expanded Programme for Immunisation, Sanofi Pasteur and PaxVax for providing the HPV, yellow fever and oral typhoid vaccines, respectively. The BCG and tetanus-diphtheria vaccines were kind donations from the Serum Institute of India. We thank the Vector Control Division of the Ministry of Health and the Mukono district local government for their support. We also thank members of the POPVAC programme steering committee (chaired by Prof. Richard Hayes) and the Data and Safety Monitoring Board (Dr David Meya, Prof Andrew Prendergast and Dr Elizabeth George).

Funding

The POPVAC programme of work is supported by the Medical Research Council of the United Kingdom (grant number MR/R02118X/1). SC and JN are supported in part by the Makerere University – Uganda Virus Research Institute Centre of Excellence for Infection and Immunity Research and Training (MUII-plus). MUII-plus is funded under the DELTAS Africa Initiative. The DELTAS Africa Initiative is an independent funding scheme of the African Academy of Sciences (AAS),

For peer review only

Alliance for Accelerating Excellence in Science in Africa (AESA) and supported by the New Partnership for Africa's Development Planning and Coordinating Agency (NEPAD Agency) with funding from the Wellcome Trust (grant 107743) and the UK Government. The MRC/UVRI and LSHTM Uganda Research Unit is jointly funded by the UK Medical Research Council (MRC) and the UK Department for International Development (DFID) under the MRC/DFID Concordat agreement and is also part of the EDCTP2 programme supported by the European Union.

The study sponsor (London School of Hygiene and Tropical Medicine) and funders had no role in study design; collection, management, analysis, and interpretation of data; writing of the protocol; and the decision to submit the protocol for publication.

POPVAC trial team

Principal investigator*:* Alison Elliott; ***Project leader***: Ludoviko Zirimenya; ***laboratory staff****:* Gyaviira Nkurunungi, Stephen Cose, Rebecca Amongin, Beatrice Nassanga, Jacent Nassuuna, Irene Nambuya, Prossy Kabuubi, Emmanuel Niwagaba, Gloria Oduru, Grace Kabami; ***statisticians and data managers****:* Emily Webb, Agnes Natukunda, Helen Akurut, Alex Mutebe; ***clinicians****:* Anne Wajja, Milly Namutebi, Christopher Zziwa, Joel Serubanja; ***nurses****:* Caroline Onen, Esther Nakazibwe, Josephine Tumusiime, Caroline Ninsiima, Susan Amongi, Florence Akello; ***internal monitor****:* Mirriam Akello; ***field workers****:* Robert Kizindo, Moses Sewankambo, Denis Nsubuga, Samuel Kiwanuka, Fred Kiwudhu; ***boatman****:* David Abiriga; ***administrative management****:* Moses Kizza, Samsi Nansukusa; ***internal and external collaborators:*** Pontiano Kaleebu, Hermelijn Smits, Maria Yazdanbakhsh, Govert van Dam, Paul Corstjens, Sarah Staedke, Henry Luzze, James Kaweesa, Edridah Tukahebwa, Elly Tumushabe, Moses Muwanga

For peer review only

407 **References**

408 1. Taniuchi M, Platts-Mills JA, Begum S, et al. Impact of enterovirus and other enteric pathogens on oral polio
409 and rotavirus vaccine performance in Bangladeshi infants. *Vaccine* 2016;34(27):3068-75. doi:
410 10.1016/j.vaccine.2016.04.080 [published Online First: 2016/05/08]

411 2. Fine PE. Variation in protection by BCG: implications of and for heterologous immunity. *Lancet*
412 1995;346(8986):1339-45.

413 3. Black GF, Weir RE, Floyd S, et al. BCG-induced increase in interferon-gamma response to mycobacterial
414 antigens and efficacy of BCG vaccination in Malawi and the UK: two randomised controlled studies. *Lancet*
415 2002;359(9315):1393-401. doi: 10.1016/S0140-6736(02)08353-8

416 4. Muyanja E, Ssemaganda A, Ngauv P, et al. Immune activation alters cellular and humoral responses to
417 yellow fever 17D vaccine. *The Journal of clinical investigation* 2014;124(7):3147-58. doi: 10.1172/jci75429
418 [published Online First: 2014/06/10]

419 5. Barreto ML, Pilger D, Pereira SM, et al. Causes of variation in BCG vaccine efficacy: examining evidence from
420 the BCG REVAC cluster randomized trial to explore the masking and the blocking hypotheses. *Vaccine*
421 2014;32(30):3759-64. doi: 10.1016/j.vaccine.2014.05.042

422 6. Tanner R, Kakalacheva K, Miller E, et al. Serum indoleamine 2,3-dioxygenase activity is associated with
423 reduced immunogenicity following vaccination with MVA85A. *BMC infectious diseases* 2014;14:660. doi:
424 10.1186/s12879-014-0660-7 [published Online First: 2014/12/04]

425 7. Sissoko MS, Healy SA, Katile A, et al. Safety and efficacy of PfSPZ Vaccine against Plasmodium falciparum via
426 direct venous inoculation in healthy malaria-exposed adults in Mali: a randomised, double-blind phase 1 trial.
427 *The Lancet Infectious diseases* 2017;17(5):498-509. doi: 10.1016/s1473-3099(17)30104-4 [published Online
428 First: 2017/02/22]

429 8. Venkatraman N, Ndiaye BP, Bowyer G, et al. Safety and Immunogenicity of a Heterologous Prime-Boost
430 Ebola Virus Vaccine Regimen in Healthy Adults in the United Kingdom and Senegal. *The Journal of infectious*
431 *diseases* 2018;219(8):1187-97. doi: 10.1093/infdis/jiy639

432 9. Barreto ML, Pereira SM, Pilger D, et al. Evidence of an effect of BCG revaccination on incidence of
433 tuberculosis in school-aged children in Brazil: second report of the BCG-REVAC cluster-randomised trial.
434 *Vaccine* 2011;29(31):4875-7. doi: 10.1016/j.vaccine.2011.05.023

435 10. van Riet E, Adegnika AA, Retra K, et al. Cellular and humoral responses to influenza in gabonese children
436 living in rural and semi-urban areas. *The Journal of infectious diseases* 2007;196(11):1671-8. doi:
437 10.1086/522010 [published Online First: 2007/11/17]

438 11. van Riet E, Retra K, Adegnika AA, et al. Cellular and humoral responses to tetanus vaccination in Gabonese
439 children. *Vaccine* 2008;26(29-30):3690-5. doi: 10.1016/j.vaccine.2008.04.067 [published Online First:
440 2008/06/10]

441 12. Venkatraman N, Ndiaye BP, Bowyer G, et al. Safety and Immunogenicity of a Heterologous Prime-Boost
442 Ebola Virus Vaccine Regimen in Healthy Adults in the United Kingdom and Senegal. *The Journal of infectious*
443 *diseases* 2019;219(8):1187-97. doi: 10.1093/infdis/jiy639

444 13. Brandt L, Feino Cunha J, Weinreich Olsen A, et al. Failure of the Mycobacterium bovis BCG vaccine: some
445 species of environmental mycobacteria block multiplication of BCG and induction of protective immunity to
446 tuberculosis. *Infection and immunity* 2002;70(2):672-8. [published Online First: 2002/01/18]

447 14. Flaherty DK, Vesosky B, Beamer GL, et al. Exposure to Mycobacterium avium can modulate established
448 immunity against Mycobacterium tuberculosis infection generated by Mycobacterium bovis BCG vaccination.

For peer review only - http://bmjopen.bmj.com/site/about/guidelines.xhtml

Journal of leukocyte biology 2006;80(6):1262-71. doi: 10.1189/jlb.0606407 [published Online First: 2006/09/14]

15. Maizels RM, McSorley HJ. Regulation of the host immune system by helminth parasites. *The Journal of allergy and clinical immunology* 2016;138(3):666-75. doi: 10.1016/j.jaci.2016.07.007 [published Online First: 2016/08/02]

16. Wammes LJ, Mpairwe H, Elliott AM, et al. Helminth therapy or elimination: epidemiological, immunological, and clinical considerations. *The Lancet Infectious diseases* 2014;14(11):1150-62. doi: 10.1016/S1473-3099(14)70771-6

17. Wammes LJ, Hamid F, Wiria AE, et al. Regulatory T cells in human geohelminth infection suppress immune responses to BCG and Plasmodium falciparum. *Eur J Immunol* 2010;40(2):437-42. doi: 10.1002/eji.200939699

18. Pfeiffer JK, Virgin HW. Viral immunity. Transkingdom control of viral infection and immunity in the mammalian intestine. *Science* 2016;351(6270) doi: 10.1126/science.aad5872

19. Onguru D, Liang Y, Griffith Q, et al. Human schistosomiasis is associated with endotoxemia and Toll-like receptor 2- and 4-bearing B cells. *The American journal of tropical medicine and hygiene* 2011;84(2):321-4. doi: 10.4269/ajtmh.2011.10-0397 [published Online First: 2011/02/05]

20. George PJ, Anuradha R, Kumar NP, et al. Evidence of microbial translocation associated with perturbations in T cell and antigen-presenting cell homeostasis in hookworm infections. *PLoS neglected tropical diseases* 2012;6(10):e1830. doi: 10.1371/journal.pntd.0001830 [published Online First: 2012/10/12]

21. Rajamanickam A, Munisankar S, Bhootra Y, et al. Microbial Translocation Associated with an Acute-Phase Response and Elevations in MMP-1, HO-1, and Proinflammatory Cytokines in Strongyloides stercoralis Infection. *Infection and immunity* 2017;85(1) doi: 10.1128/iai.00772-16 [published Online First: 2016/11/09]

22. Sanya RE, Nkurunungi G, Andia Biraro I, et al. A life without worms. *Transactions of the Royal Society of Tropical Medicine and Hygiene* 2017:1-9. doi: 10.1093/trstmh/trx010 [published Online First: 2017/03/25]

23. Elias D, Britton S, Aseffa A, et al. Poor immunogenicity of BCG in helminth infected population is associated with increased in vitro TGF-beta production. *Vaccine* 2008;26(31):3897-902. doi: S0264-410X(08)00540-9 [pii]
10.1016/j.vaccine.2008.04.083 [published Online First: 2008/06/17]

24. Elias D, Wolday D, Akuffo H, et al. Effect of deworming on human T cell responses to mycobacterial antigens in helminth-exposed individuals before and after bacille Calmette-Guerin (BCG) vaccination. *Clin Exp Immunol* 2001;123(2):219-25.

25. Cooper PJ, Chico ME, Losonsky G, et al. Albendazole treatment of children with ascariasis enhances the vibriocidal antibody response to the live attenuated oral cholera vaccine CVD 103-HgR. *The Journal of infectious diseases* 2000;182(4):1199-206. doi: 10.1086/315837 [published Online First: 2000/09/09]

26. Tweyongyere R, Nassanga BR, Muhwezi A, et al. Effect of Schistosoma mansoni infection and its treatment on antibody responses to measles catch-up immunisation in pre-school children: A randomised trial. *PLoS neglected tropical diseases* 2019;13(2):e0007157. doi: 10.1371/journal.pntd.0007157 [published Online First: 2019/02/15]

27. Chan AW, Tetzlaff JM, Altman DG, et al. SPIRIT 2013 statement: defining standard protocol items for clinical trials. *Ann Intern Med* 2013;158(3):200-7. doi: 10.7326/0003-4819-158-3-201302050-00583 [published Online First: 2013/01/09]

28. Sanya RE, Nkurunungi G, Hoek Spaans R, et al. The Impact of Intensive Versus Standard Anthelminthic Treatment on Allergy-related Outcomes, Helminth Infection Intensity, and Helminth-related Morbidity in Lake Victoria Fishing Communities, Uganda: Results From the LaVIISWA Cluster-randomized Trial. *Clinical*

For peer review only - http://bmjopen.bmj.com/site/about/guidelines.xhtml

infectious diseases : an official publication of the Infectious Diseases Society of America 2019;68(10):1665-74. doi: 10.1093/cid/ciy761 [published Online First: 2018/09/12]

29. WHO. Adolescent Health Research Priorities: Report of a Technical Consultation 2015. http://apps.who.int/iris/bitstream/10665/203564/1/WHO_FWC_MCA_15_07_eng.pdf?ua=1 (accessed 17th May 2019).

30. Webb EL, Nampijja M, Kaweesa J, et al. Helminths are positively associated with atopy and wheeze in Ugandan fishing communities: results from a cross-sectional survey. *Allergy* 2016 doi: 10.1111/all.12867

31. Montresor A, Odermatt P, Muth S, et al. The WHO dose pole for the administration of praziquantel is also accurate in non-African populations. *Transactions of the Royal Society of Tropical Medicine and Hygiene* 2005;99(1):78-81. doi: 10.1016/j.trstmh.2004.06.006

32. Plotkin SA. Correlates of protection induced by vaccination. *Clinical and vaccine immunology : CVI* 2010;17(7):1055-65. doi: 10.1128/cvi.00131-10 [published Online First: 2010/05/14]

33. Riner DK, Ndombi EM, Carter JM, et al. Schistosoma mansoni Infection Can Jeopardize the Duration of Protective Levels of Antibody Responses to Immunizations against Hepatitis B and Tetanus Toxoid. *PLoS neglected tropical diseases* 2016;10(12):e0005180. doi: 10.1371/journal.pntd.0005180

34. Corstjens PL, Nyakundi RK, de Dood CJ, et al. Improved sensitivity of the urine CAA lateral-flow assay for diagnosing active Schistosoma infections by using larger sample volumes. *Parasites & vectors* 2015;8:241. doi: 10.1186/s13071-015-0857-7 [published Online First: 2015/04/22]

35. Fletcher HA, Snowden MA, Landry B, et al. T-cell activation is an immune correlate of risk in BCG vaccinated infants. *Nat Commun* 2016;7:11290. doi: 10.1038/ncomms11290

36. Safaeian M, Porras C, Pan Y, et al. Durable antibody responses following one dose of the bivalent human papillomavirus L1 virus-like particle vaccine in the Costa Rica Vaccine Trial. *Cancer prevention research (Philadelphia, Pa)* 2013;6(11):1242-50. doi: 10.1158/1940-6207.capr-13-0203 [published Online First: 2013/11/06]

37. Harris PA, Taylor R, Minor BL, et al. The REDCap Consortium: Building an International Community of Software Platform Partners. *Journal of biomedical informatics* 2019:103208. doi: 10.1016/j.jbi.2019.103208 [published Online First: 2019/05/13]

38. Harris PA, Taylor R, Thielke R, et al. Research electronic data capture (REDCap)--a metadata-driven methodology and workflow process for providing translational research informatics support. *Journal of biomedical informatics* 2009;42(2):377-81. doi: 10.1016/j.jbi.2008.08.010 [published Online First: 2008/10/22]

39. Blok BA, Arts RJ, van Crevel R, et al. Trained innate immunity as underlying mechanism for the long-term, nonspecific effects of vaccines. *J Leukoc Biol* 2015;98(3):347-56. doi: 10.1189/jlb.5RI0315-096R

40. Watanabe K, Mwinzi PN, Black CL, et al. T regulatory cell levels decrease in people infected with Schistosoma mansoni on effective treatment. *The American journal of tropical medicine and hygiene* 2007;77(4):676-82. [published Online First: 2007/11/06]

41. Schmiedel Y, Mombo-Ngoma G, Labuda LA, et al. CD4+CD25hiFOXP3+ Regulatory T Cells and Cytokine Responses in Human Schistosomiasis before and after Treatment with Praziquantel. *PLoS neglected tropical diseases* 2015;9(8):e0003995. doi: 10.1371/journal.pntd.0003995 [published Online First: 2015/08/21]

42. van den Biggelaar AHJ, Borrmann S, Kremsner P, et al. Immune Responses Induced by Repeated Treatment Do Not Result in Protective Immunity to Schistosoma haematobium: Interleukin (IL)–5 and IL-10 Responses. *The Journal of infectious diseases* 2002;186(10):1474-82. doi: 10.1086/344352

For peer review only

For peer review only - http://bmjopen.bmj.com/site/about/guidelines.xhtml

534 43. Woolhouse MEJ, Hagan P. Seeking the ghost of worms past. *Nature Medicine* 1999;5(11):1225-27. doi: 10.1038/15169

536 44. Seledtsov VI, Goncharov AG, Seledtsova GV. Clinically feasible approaches to potentiating cancer cell-based immunotherapies. *Human vaccines & immunotherapeutics* 2015;11(4):851-69. doi: 10.1080/21645515.2015.1009814 [published Online First: 2015/05/02]

FIGURE LEGENDS

Figure 1. Outline of immunisations and anthelminthic intervention

[1]Primary endpoints will be at eight weeks post BCG and four weeks post Yellow fever (YF-17D), oral typhoid (Ty21a), Human Papilloma Virus (HPV) and Tetanus/diptheria (Td) vaccination.

[2]Primary endpoint for responses to Td given at 28 weeks.

For peer review only

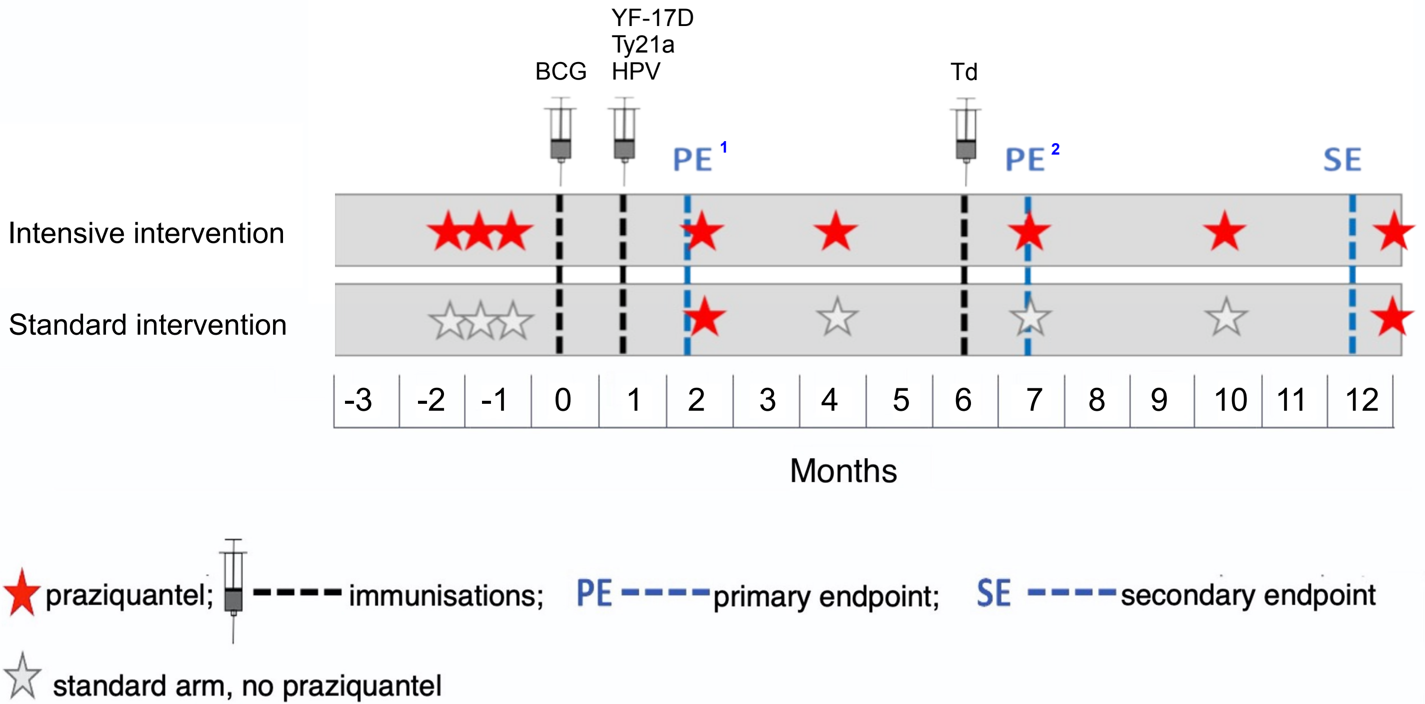
YF-17D
Ty21a
HPV
BCG
Td
PE 1
PE 2
SE
Intensive intervention
Standard intervention
-3
-2
-1
0
1
2
3
4
5
6
7
8
9
10
11
12
Months
praziquantel;
immunisations;
PE
primary endpoint;
SE
secondary endpoint
standard arm, no praziquantel

SUPPLEMENTARY INFORMATION

The effect of intensive treatment for schistosomiasis on immune responses to vaccines among rural Ugandan island adolescents: randomised controlled trial protocol A for the 'POPulation differences in VACcine responses' (POPVAC) programme

Gyaviira Nkurunungi[1,¶,*], Ludoviko Zirimenya[1,¶], Jacent Nassuuna[1,¶], Agnes Natukunda[1,¶], Prossy N Kabuubi[1], Emmanuel Niwagaba[1], Gloria Oduru[1], Grace Kabami[1], Rebecca Amongin[1], Alex Mutebe [1], Milly Namutebi[1], Christopher Zziwa[1], Susan Amongi[1], Caroline Ninsiima[1], Caroline Onen[1], Florence Akello[1], Moses Sewankambo[1], Samuel Kiwanuka[1], Robert Kizindo[1], James Kaweesa[2], Stephen Cose[1,3], Emily L Webb[4], Alison M Elliott[1,3] **for the POPVAC trial team**

[1]Immunomodulation and Vaccines Programme, Medical Research Council/Uganda Virus Research Institute and London School of Hygiene and Tropical Medicine (MRC/UVRI and LSHTM) Uganda Research Unit, Entebbe, Uganda

[2]Vector Control Division, Ministry of Health, Kampala, Uganda

[3]Department of Clinical Research, London School of Hygiene and Tropical Medicine, London, United Kingdom

[4]MRC Tropical Epidemiology Group, Department of Infectious Disease Epidemiology, London School of Hygiene and Tropical Medicine, London, United Kingdom

¶These authors contributed equally

***Correspondence:** Gyaviira Nkurunungi; Gyaviira.Nkurunungi@mrcuganda.org

For peer review only

Table S1. Schedule of visits and procedures

VISIT NUMBER	1	2 & 3	4	5[9]	5.2	6	7	8	9	10	11
WEEKS FROM 1ST IMMUNISATION	-8[1]	-6[10], -4, -2	0	4	4 weeks +4 days	8	20	28	32	44	52
	Screening	Treatment (Rx) only	Immunisations	Immunisations		Primary Endpoint (PE), Immunisations	Rx only	Immunisations	PE/Secondary Endpoint (SE)[2]	Rx only	SE, Immunisations
**RANDOMISED INTENSIVE VS STANDARD PRAZIQUANTEL INTERVENTION**											
PZQ intensive arm (x)		x				x[3]	x		x[3]	x	x[3]
PZQ standard arm						x[3]					x[3]
Albendazole						x[3]			x[3]		x[3]
**VACCINES**											
BCG			x								
YF-17D				x							
Ty21a				x							
HPV				x		[x][4]		x			
Td								x			[x][5]
**INVESTIGATIONS/PROCEDURES**											
Inclusion/exclusion criteria	x										
Informed consent	x										
Questionnaire	x		x	x	x	x		x	x		x
Examination	x		(x)	(x)	(x)	(x)		(x)	(x)		(x)
Urine β-HCG test (female only) 1mL	x		x	x				x			
Urine YF viral load					x						
Stool for PCR and storage	x							x			x
Stool for coproantibody and storage	x					x					
**BLOOD SAMPLES**											
Malaria PCR (1ml)			x	x				x			x
Serology for HIV, prior malaria and *S. mansoni* (0.5ml)	x										
Mansonella perstans (1ml)	x										
Serum/plasma CAA (1ml)	x		x	x		x		x	x		x
Hb[8] / Full blood count (0.5ml)	x		x	x				x			
Assessments of pre-immunisation responses, and/or vaccine response outcomes and/or exploratory immunology; storage[7] (10 – 20 mls)			x	x		x		x	x		x
Blood for gene expression (2mls)			x	x				x			
Blood vol (mL)	4		27	17		10-20		27	10		14
Cumulative blood vol (mL)[7]	4		31	48		68		95	105		119

PE: primary endpoint; **SE:** secondary endpoint; **Rx only**: treatment only
Immunisation days highlighted in green, primary end point days in red, days for treatment only in grey
(**x**) performed if clinically indicated

1. Screening and enrolment into Project A will take place about 8 weeks before immunisation 0 to allow initiation of the praziquantel intervention.
2. Week 32 is primary endpoint for responses to Td given at 28 weeks; secondary endpoint for HPV booster
3. Treatments given after sampling when schedules coincide
4. Week 8 HPV dose will be given for previously-unvaccinated girls aged ≥14 years
5. Week 52 Td booster dose will be provided as a service
6. Exploratory immunology blood volume will be guided by guidelines from Harvard Mass General, where a maximum of 3ml/kg body weight is taken at any one time point and not more than 3ml/kg is taken over any 8-week period (ref http://www.drgreene.com/21_1616.html.) These guidelines have been followed in a previous study vaccinating adolescents with investigational tuberculosis vaccine MVA85A (in Uganda).[1] The total blood volume planned is 68 ml over the initial intensive sampling period of 8 weeks. Revision of sample volumes based on weight will only be required for participants who weigh less than 22 kg; the average weight of children aged 9 years is expected to be 28kg (with 21kg the 3rd centile) with greater weights for older children.[2]
7. At baseline, it will only be Hb estimation by Haemocue
8. Oral typhoid vaccine doses will be administered on three alternate days namely visit 5, 5.1 and 5.2.
9. The first PZQ treatment at week -6 will be administered at the end of the screening visit

22

For peer review only

Further information on recruitment criteria

- Participants who are excluded from the trial because they have been discovered (during screening procedures) to be suffering from a previously undiagnosed condition thought to require further medical attention will be referred appropriately for further investigation and treatment.
- Participants discovered to have severe anaemia will be excluded from the trial and treated for anaemia
- Participants discovered to be HIV-positive will be counselled and offered a CD4 T-cell count and referred to a provider of antiretroviral treatment ("Test and Treat" – i.e. initiation of treatment regardless of CD4 count is recommended for these high-risk communities).
- Participants discovered to be pregnant will be counselled and referred to an antenatal clinic of their choice.

This trial proposes to recruit all participants within a short time-frame. It will not, therefore, be possible to reconsider enrolment of potential participants with temporary exclusion criteria after treatment and resolution of the condition.

Further rationale for the selection of vaccines

Bacillus Calmette–Guérin (BCG)

BCG is a live, replicating parenteral vaccine, and the only licensed vaccine against TB. The BCG vaccine for these studies will be obtained from the Serum Institute of India either directly, or through a supplier in Uganda. The Serum Institute of India provides much of the BCG vaccine used in Uganda.

Worldwide, TB is among the top 10 causes of death; Uganda has an estimated incidence of 202/100,000 people.[3] Infectious, sputum positive, pulmonary TB classically emerges in adolescence, driving the on-going epidemic.[4] Thus adolescent booster immunisation is a key TB control strategy.[5] However, BCG vaccine response and efficacy are often impaired in tropical and rural settings[6-8] and new TB vaccines are similarly affected.[9] In the past, the WHO has been hesitant to recommend BCG re-vaccination. However, in 2017 WHO's Strategic Advisory Group of Experts (SAGE) recommended: "Further research is warranted to explore whether certain sub-groups of age, geographic or *M. tuberculosis* exposure categories would benefit from re-vaccination."[10] Recent results suggest that, despite the variability of BCG efficacy between populations, BCG vaccination in adolescence offers benefit in some tropical settings, especially for individuals who are not yet infected with *Mycobacterium tuberculosis*, and may also be cost-effective.[7,11] Also, BCG vaccine is currently being used among adolescents in South Africa as a comparator in a trial of a novel TB vaccine (trial

For peer review only

registration NCT02075203). BCG efficacy in Ugandan adolescents, and differences in BCG vaccine responses between urban and rural Ugandan populations, have not been tested. Information obtained from this study is expected to further inform the use of BCG in adolescents, and also to inform the development of new vaccines for tuberculosis.

Yellow fever vaccine

Yellow fever vaccine YF-17D is a live replicating parenteral vaccine. The vaccine (Stamaril; Sanofi Pasteur) is available for purchase in Uganda. Yellow Fever (YF) causes outbreaks in Uganda and the wider region[12] and YF-17D is a candidate for Uganda's expanded programme on immunisation (EPI). Lower vaccine replication, lower neutralising antibody induction, and greater waning, are described in Uganda compared to Switzerland.[13] YF-17D is a potential vector for novel vaccine constructs,[14] adding relevance to vaccine development.

Typhoid vaccine Ty21a

Typhoid vaccine Ty21a is a live replicating oral vaccine and also a potential vector for new vaccine constructs.[15] The Ty21a vaccine will be purchased from PaxVax, Redwood City, California. Substantial, multi-year typhoid outbreaks occur in Uganda and immunisation campaigns have been advocated as cost effective.[16] Schistosomiasis has been associated with prolonged *S. typhi* infection[17] and impaired antibody responses to killed typhoid vaccines.[18]

Ty21a was developed in the 1970s. Although not routinely used in Uganda, it has been (and is currently) registered in many countries. It was first registered in the United States and United Kingdom in the 1980s, and is recommended by the WHO for both endemic and epidemic settings.[19] It has comparable efficacy to the parenteral Vi polysaccharide typhoid vaccine, good durability and minimal adverse effects.[19] It is proposed for use in this study to model effects of study exposures and intervention on the response to a live oral vaccine.

The Ty21a vaccine is given as a three-dose regimen on alternate days.

Human Papilloma Virus (HPV) vaccine

The Human Papilloma Virus (HPV) vaccine is a protein virus-like particle. The quadrivalent HPV Vaccine Gardasil (Merck) is available for purchase in Uganda and is the vaccine used by the national EPI programme. Studies after three vaccine doses have found somewhat enhanced responses in the presence of malaria, but no effect of helminths.[20] No study has previously investigated parasite effects on the priming response, but recent results for tetanus suggest that priming may be more susceptible than boosting to adverse effects.[21] This will be important if forthcoming trials support single-dose HPV immunisation (NCT02834637). HPV immunisation is being rolled out among girls to

For peer review only

For peer review only - http://bmjopen.bmj.com/site/about/guidelines.xhtml

prevent cervical neoplasia, the most common cancer among Ugandan women and we will coordinate provision with the national HPV immunisation programme.[22] HPV immunisation is also beneficial for boys since HPV infection is associated with anogenital warts, anal cancer and oropharyngeal cancers in both males and females, and with penile cancer in men,[23] and we will include boys in these studies.

Tetanus and diphtheria vaccines

Tetanus and diphtheria (Td) vaccines comprise inert toxoids. Schistosomiasis is associated with a Th2 biased response to tetanus toxoid[24] and with suppressed antibody responses among those with low pre-immunisation antibody levels.[21] Booster immunisation is recommended for young women to prevent maternal and neonatal tetanus. Recent evidence emphasises the need to protect young men also.[25]

Immunisation Postponement Criteria

If any one of the following is identified at the time scheduled for immunisation, the participant may be immunised at a later date, or withdrawn, at the discretion of the Investigator. The participant must be followed until resolution of the event as with any adverse event:

- Acute disease at the time of immunisation. Acute disease is defined as the presence of a moderate or severe illness with or without fever. All vaccines can be administered to persons with a minor illness such as diarrhoea or mild upper respiratory infection with or without low-grade fever, i.e. temperature of ≤37.5°C (99.5°F)
- Temperature of >37.5°C (99.5°F) at the time of immunisation
- Taking antibiotics or antimalarials currently, or within the past 7 days, of the date of Ty21a administration (ascertained verbally)

Vaccine storage and transport

In order to maintain a reliable vaccine cold chain, the vaccines and diluents to be used will be stored and transported within the recommended temperature range of +2°C to +8°C. Care will be taken to ensure that the vaccines are not frozen. BCG, being sensitive to light, will be kept in the dark (normally within its secondary packaging) for as long as possible to protect it during storage and transportation. All vaccines will be kept in appropriate refrigeration equipment with a temperature monitoring device to ensure temperatures remain between +2°C and +8°C. Cold boxes/vaccines carriers with temperature monitors will be used to transport vaccines and the diluents from the MRC/UVRI and LSHTM Uganda Research Unit (Entebbe) to Koome island and while transporting vaccines to immunisation sessions. Designated staff will be given responsibility for managing the

For peer review only - http://bmjopen.bmj.com/site/about/guidelines.xhtml

vaccine cold chain. All cold chain equipment including the temperature monitoring devices used for this project will comply with relevant technical specifications as defined by the EPI standards. Basic routine maintenance will be regularly carried out on all cold chain equipment.

Additional laboratory measurements

Additional assays will comprise HIV serology, pregnancy testing and full blood counts. HIV testing and pregnancy testing will be accompanied by appropriate counselling by trained staff.

- HIV serology will be done on blood samples using rapid tests and according to prevailing national algorithms.[26] This will be done at baseline.
- Pregnancy testing will be done using urine samples and standard operating procedures for assessment of urine β-human chorionic gonadotropin (βhCG). This will be done at baseline and before immunisation on each immunisation day.
- Full blood counts will be conducted using a haematology analyser. Mild, moderate and severe anaemia will be defined according to WHO guidelines, by age.[27] This will be done at baseline to test for anaemia as part of the eligibility assessment, and pre-immunisation as part of the assessment of immunological profile.

Individuals found to be HIV positive or pregnant will be referred to appropriate providers for further care. Individuals with severe anaemia (haemoglobin <82g/L) will be excluded from the randomised intervention (since the intervention might be beneficial in management of anaemia). They will be treated for anaemia.

Sample handling and archive

Blood and other samples will be processed according to local laboratory standard operating procedures (SOPs). All samples will reach the laboratory in anonymised form.

A sample archive will be developed. Although our current programme of work will address specific hypotheses regarding pathways of effects of parasites and interventions, the sample archive will provide a major asset for exploration of new leads arising from this work, or for an alternative, "systems biology" approach employing (for example) proteomic, genomic, epigenetic and transcriptomic analyses, and investigating the microbiome and virome. Information provided to participants, and consent forms, will include considerations of sample storage, and the possibility of sample analysis in laboratories within and outside Uganda. Participants will be able to decide if they will permit such future use of any leftover samples. We plan to store the samples for up to 20 years. If further storage is needed after that time, permission will be requested from the Uganda Virus Research Institute and London School of Hygiene and Tropical Medicine ethical review committees.

For peer review only

For peer review only - http://bmjopen.bmj.com/site/about/guidelines.xhtml

If they elect not to permit this, all of those leftover samples will be discarded after the completion of the work included in the current protocol.

Operational considerations

Programme governance

A Programme Steering Committee has been set up to guide progress across all projects. This comprises the following:

- An independent chair
- Representatives from the Ministry of Health programmes for immunisation and for vector borne disease control
- Representatives of district authorities (Mukono and Jinja districts)
- Community representatives
- Principal investigator and co-investigators
- Project leader and post-doctoral immunologist
- Trial statistician
- Laboratory manager
- Medical Research Council observer

Informed consent

Both written informed assent from the participants and written informed consent from a parent or guardian will be required for participation, although these may not necessarily be obtained at the same time. Information will be provided in both English and the appropriate local language. For individuals who cannot speak the languages used, or who cannot read or write, a witness who can read the information sheet and translate the information to the participant or parent/guardian will be used. Two different types of age specific assent forms will be used for the group of participants aged 9 – 12 years and for the group aged 13 – 17 years. Informed consent by emancipated or mature minors will be obtained using a designated consent form for these categories of participants.

The aims of the study, all tests, treatments and immunisations to be carried out and potential risks will be explained. The participant will be given the opportunity to ask about details of the trial, and will then have time to consider whether or not to participate. If they do decide to participate, they and their parent/guardian will sign and date two copies of the assent and consent forms, one for them to take away and keep, and one to be stored securely by the research team. Separate information and consent forms will be provided (i) for consent for storage of samples for future

For peer review only

For peer review only - http://bmjopen.bmj.com/site/about/guidelines.xhtml

studies and for anonymous sharing of data from this study and (ii) for possible genetic studies; the information sheet will explain that these data may be used in analyses related to this protocol.

Screening and Eligibility Assessment

Once the informed consent process has been completed, and consent (and assent) given, a baseline medical history (including concomitant medication) will be collected. Vital signs will be checked and a physical examination will be performed. Inclusion and exclusion criteria will be checked.

Participants will undergo pre- and post-test counselling for HIV and (for girls) pregnancy testing by a trained and experienced nurse- or clinician-counsellor. Blood, urine and stool samples will be obtained, for tests as specified in the schedule of procedures. These tests are to exclude the major, immunomodulating co-infection, HIV, and conditions that might impact safety (anaemia, pregnancy).

Enrolment

Participants who consent/assent, complete the screening processes, satisfy all the inclusion criteria and meet none of the exclusion criteria will be enrolled.

Discontinuation/withdrawal criteria

In accordance with the principles of the current revision of the Declaration of Helsinki and any other applicable regulations, a participant has the right to withdraw from the study at any time and for any reason, and is not obliged to give his or her reasons for doing so. The Investigator may withdraw the participant at any time in the interests of the participant's health and well-being. In addition, the participant may withdraw/be withdrawn for any of the following reasons:

- Ineligibility (either arising during the study or retrospectively, having been overlooked at screening)
- Administrative decision by the Investigator
- Significant protocol deviation
- Participant non-compliance with study requirements
- An adverse event which requires discontinuation of the study involvement or results in inability to continue to comply with study procedures.

Any participant who becomes pregnant during the trial will be followed up until the end of the pregnancy but no further immunisations will be given unless indicated during pregnancy (as is the case for tetanus toxoid). The trial allocation for this participant will be unblinded and the participant will only be given further treatment if clinically indicated. The babies will also be followed up and

For peer review only

examined for any adverse effects. We will not routinely perform venepuncture in a pregnant participant.

The reason for withdrawal will be recorded in the case report form (CRF). If withdrawal is due to an AE, appropriate follow-up visits or medical care will be arranged, with the agreement of the participant, until the AE has resolved, stabilised or a non-trial related causality has been assigned.

If a participant withdraws from the study samples collected before their withdrawal from the trial will be used/ stored unless the participant specifically requests otherwise.

Trial discontinuation

The trial will be discontinued in the event of new scientific information that renders continuation futile or unethical, or for any other reason, at the discretion of the Programme Steering Committee.

End of study definition

The trial will be completed when the last participant enrolled into the trial has completed their final follow up visit.

Safety assessments and oversight

No new investigational drug or product will be used in the proposed trial. However, standard approaches for monitoring safety and reporting of serious adverse events will be followed.

Monitoring

The trial will be monitored by both internal and external monitors according to a pre-defined monitoring plan which will include a site initiation visit, monitoring visits at least annually, and a close-out visit. The monitors will assess patient safety, data integrity, and adherence to the protocol and to Good Clinical Research Practice procedures.

Considerations regarding standard of care

S. mansoni infection status will be determined retrospectively through assays conducted in bulk on stored samples (plasma CAA). These results will not, therefore, be useful to determine management of individual participants.

Participants in the standard treatment arm will receive lower levels of anthelminthic treatment. However, all trial arms will receive a minimum of well-implemented national standard of care.

Standard of care will comprise annual praziquantel treatment. Our own results from the Lake Victoria Island Intervention Study on Worms and Allergy-related diseases (LaVIISWA),[28] which compared annual versus quarterly intervention for schistosomiasis at community level over three

For peer review only

244 years, showed no advantage of quarterly treatment for morbidity outcomes attributed to
245 schistosomiasis.

246 Schistosomiasis can cause anaemia. To manage the expected differential benefits of the
247 interventions for anaemia, a full blood count will be performed at baseline, as discussed above;
248 anaemic children will be managed appropriately and severely anaemic children excluded.

249 Albendazole will be provided twice a year to manage nematode infections (after collection of
250 primary and secondary endpoint samples).

251 ***Procedures to be followed in the event of abnormal findings***

252 Abnormal clinical findings from medical history, examination or blood tests will be assessed as to
253 their clinical significance throughout the trials. If an abnormal test result is deemed clinically
254 significant, it may be repeated. If a test remains clinically significant, the participant will be informed
255 and appropriate medical care arranged as appropriate and with the permission of the participant.
256 Specific details regarding findings, discussion with participants and resulting actions will be recorded
257 in the clinical records. Decisions to exclude the participant from enrolling in the trial or to withdraw
258 a participant from the trial will be at the discretion of the Investigator.

259 ***Data and Safety Monitoring Board (DSMB)***

260 A data and safety monitoring board (DSMB) has been appointed to provide real-time safety
261 oversight. The DSMB will be notified within 7 days of the Investigators' being aware of the
262 occurrence of SAEs. The DSMB may recommend the Investigators to place the trial on hold if
263 deemed necessary following an intervention-related SAE. The DSMB will be chaired by a clinician
264 experienced in clinical trials. There will be a minimum of two other appropriately qualified
265 committee members. In the case of events related to a blinded intervention, the DSMB can request
266 unblinding. Membership will include a statistician, and at least one Ugandan member. All
267 correspondence between Investigators and the DSMB will be conveyed by the Principal Investigator
268 to the trial Sponsor. The Chair of the DSMB will be contacted for advice and independent review by
269 the Investigator or trial Sponsor in the following situations:

270 • The occurrence of any SAE
271 • Any other situation where the Investigator or trial Sponsor feels independent advice or
272 review is important

273 ***Ethical and regulatory considerations***

274 *Further information regarding risks*

For peer review only - http://bmjopen.bmj.com/site/about/guidelines.xhtml

The immunisations to be given have recognised side effects which are usually mild and resolve spontaneously in a few days to one week. Parenteral vaccines are likely to result in pain and swelling at the site of injection and mild fever; very occasionally pain and swelling can be severe and associated with difficulty in moving the shoulder. Sometimes headache and tiredness occur. Rarely a vaccine may cause a severe allergic reaction. For most vaccines this is estimated at less than one in a million doses (but 1 in 55,000 for Yellow Fever vaccine).[29] Individuals with a history of a possible allergic reaction to drugs or vaccines, or to vaccine components including eggs or chicken proteins, will be excluded from the studies. The research team will be trained and prepared to manage severe allergic reactions.

Adverse reactions to Yellow Fever vaccine include severe nervous system reaction (about 1 person in 125,000) and severe, life-threatening illness with organ failure (about 1 person in 250,000). The mortality for this severe, life-threatening adverse effect is reported as about 50%.[29]

BCG immunisation is likely to induce a scar in many cases. This may develop over several weeks, starting as a small papule at the injection site which may become ulcerated and then heal over a period of 2 to 5 months; and lymphadenopathy may develop. Occasionally a more severe local reaction occurs (estimated at 1 per 1,000-10,000 doses): for example, an abscess develops and scars may develop into keloids. Rarely BCG can cause disseminated disease (1 per 230,000 to 640,000 doses), or disease in sites remote from the immunisation site. Disseminated BCG disease usually occurs in immunocompromised people: HIV positive people will be excluded from these studies.[30] BCG "pre-immunisation" may interfere with the response to the subsequent live vaccines; indeed our hypothesis, and published results, suggest that it may suppress replication of YF 17D vaccine.[31] However, this reduced replication has not been shown to correlate with, or result in, reduced levels of neutralising antibody titres (which are the desired protective outcome).[13 31]

Oral typhoid vaccine (Ty21a) may occasionally be associated with stomach pain, nausea, vomiting and (rarely) rash.[29]

Praziquantel has been in use for about 30 years. It has a well-recognised profile of side effects including dizziness, nausea, vomiting, abdominal pain, diarrhoea (sometimes with blood) and urticarial rash. The symptoms are considered to arise largely from the effects of killing worms and to be more severe in people with heavy infections. Symptoms are better tolerated when the drugs are given after food and we will provide treatment after a meal or snack. Simple medications, such as paracetamol and cetirizine, can alleviate symptoms and these will be available on treatment days.

For peer review only - http://bmjopen.bmj.com/site/about/guidelines.xhtml

References

1. Wajja A, Kizito D, Nassanga B, et al. The effect of current Schistosoma mansoni infection on the immunogenicity of a candidate TB vaccine, MVA85A, in BCG-vaccinated adolescents: An open-label trial. *PLoS neglected tropical diseases* 2017;11(5):e0005440. doi: 10.1371/journal.pntd.0005440 [published Online First: 2017/05/05]

2. WHO. Growth reference 5-19 years. 2007

3. WHO. Global tuberculosis report 2016 http://www.who.int/tb/publications/global_report/en/ (accessed 17 June 2017), 2016.

4. Alcais A, Fieschi C, Abel L, et al. Tuberculosis in children and adults: two distinct genetic diseases. *J Exp Med* 2005;202(12):1617-21. doi: 10.1084/jem.20052302

5. Weiner J, 3rd, Kaufmann SH. Recent advances towards tuberculosis control: vaccines and biomarkers. *J Intern Med* 2014;275(5):467-80. doi: 10.1111/joim.12212

6. Fine PE. Variation in protection by BCG: implications of and for heterologous immunity. *Lancet (London, England)* 1995;346(8986):1339-45.

7. Barreto ML, Pilger D, Pereira SM, et al. Causes of variation in BCG vaccine efficacy: examining evidence from the BCG REVAC cluster randomized trial to explore the masking and the blocking hypotheses. *Vaccine* 2014;32(30):3759-64. doi: 10.1016/j.vaccine.2014.05.042

8. Black GF, Weir RE, Floyd S, et al. BCG-induced increase in interferon-gamma response to mycobacterial antigens and efficacy of BCG vaccination in Malawi and the UK: two randomised controlled studies. *Lancet (London, England)* 2002;359(9315):1393-401. doi: 10.1016/S0140-6736(02)08353-8

9. Tanner R, Kakalacheva K, Miller E, et al. Serum indoleamine 2,3-dioxygenase activity is associated with reduced immunogenicity following vaccination with MVA85A. *BMC infectious diseases* 2014;14:660. doi: 10.1186/s12879-014-0660-7 [published Online First: 2014/12/04]

10. WHO. SAGE Evidence to recommendations framework. 2017. http://www.who.int/immunization/sage/meetings/2017/october/2_EvidencetoRecommendationFramework_BCG.pdf (accessed 16th March 2018).

11. Dye C. Making wider use of the world's most widely used vaccine: Bacille Calmette-Guerin revaccination reconsidered. *Journal of the Royal Society, Interface* 2013;10(87):20130365. doi: 10.1098/rsif.2013.0365 [published Online First: 2013/08/02]

12. Kraemer MU, Faria NR, Reiner RC, Jr., et al. Spread of yellow fever virus outbreak in Angola and the Democratic Republic of the Congo 2015-16: a modelling study. *The Lancet Infectious diseases* 2016 doi: 10.1016/s1473-3099(16)30513-8 [published Online First: 2016/12/27]

13. Muyanja E, Ssemaganda A, Ngauv P, et al. Immune activation alters cellular and humoral responses to yellow fever 17D vaccine. *The Journal of clinical investigation* 2014;124(7):3147-58. doi: 10.1172/jci75429 [published Online First: 2014/06/10]

14. Aguiar M, Stollenwerk N, Halstead SB. The Impact of the Newly Licensed Dengue Vaccine in Endemic Countries. *PLoS neglected tropical diseases* 2016;10(12):e0005179. doi: 10.1371/journal.pntd.0005179 [published Online First: 2016/12/22]

15. Dharmasena MN, Osorio M, Filipova S, et al. Stable expression of Shigella dysenteriae serotype 1 O-antigen genes integrated into the chromosome of live Salmonella oral vaccine vector Ty21a. *Pathogens and disease* 2016 doi: 10.1093/femspd/ftw098 [published Online First: 2016/09/23]

For peer review only

For peer review only - http://bmjopen.bmj.com/site/about/guidelines.xhtml

16. Carias C, Walters MS, Wefula E, et al. Economic evaluation of typhoid vaccination in a prolonged typhoid outbreak setting: the case of Kasese district in Uganda. *Vaccine* 2015;33(17):2079-85. doi: 10.1016/j.vaccine.2015.02.027 [published Online First: 2015/02/26]

17. Melhem RF, LoVerde PT. Mechanism of interaction of Salmonella and Schistosoma species. *Infection and immunity* 1984;44(2):274-81. [published Online First: 1984/05/01]

18. Muniz-Junqueira MI, Tavares-Neto J, Prata A, et al. Antibody response to Salmonella typhi in human schistosomiasis mansoni. *Revista da Sociedade Brasileira de Medicina Tropical* 1996;29(5):441-5. [published Online First: 1996/09/01]

19. WHO. Position Paper on Typhoid vaccines: WHO position paper – March 2018 2018

20. Brown J, Baisley K, Kavishe B, et al. Impact of malaria and helminth infections on immunogenicity of the human papillomavirus-16/18 AS04-adjuvanted vaccine in Tanzania. *Vaccine* 2014;32(5):611-7. doi: 10.1016/j.vaccine.2013.11.061

21. Riner DK, Ndombi EM, Carter JM, et al. Schistosoma mansoni Infection Can Jeopardize the Duration of Protective Levels of Antibody Responses to Immunizations against Hepatitis B and Tetanus Toxoid. *PLoS neglected tropical diseases* 2016;10(12):e0005180. doi: 10.1371/journal.pntd.0005180

22. Centre HI. HPV and related diseases report: Uganda. 2016. http://www.hpvcentre.net/statistics/reports/UGA.pdf (accessed 20.01.2017).

23. WHO. Human papillomavirus vaccines: WHO position paper, May 2017. *Releve epidemiologique hebdomadaire* 2017;92(19):241-68. [published Online First: 2017/05/23]

24. Sabin EA, Araujo MI, Carvalho EM, et al. Impairment of tetanus toxoid-specific Th1-like immune responses in humans infected with Schistosoma mansoni. *The Journal of infectious diseases* 1996;173(1):269-72. [published Online First: 1996/01/01]

25. Nanteza B, Galukande M, Aceng J, et al. The burden of tetanus in Uganda. *SpringerPlus* 2016;5(1):705. doi: 10.1186/s40064-016-2309-z [published Online First: 2016/06/29]

26. Ministry of Health–Uganda. Uganda Clinical Guidelines 2016. http://apps.who.int/medicinedocs/documents/s23532en/s23532en.pdf

27. WHO. Haemoglobin concentrations for the diagnosis of anaemia and assessment of severity. Vitamin and Mineral Nutrition Information System. Geneva, World Health Organization, 2011 (WHO/NMH/NHD/MNM/11.1).

28. Sanya RE, Nkurunungi G, Hoek Spaans R, et al. The Impact of Intensive Versus Standard Anthelminthic Treatment on Allergy-related Outcomes, Helminth Infection Intensity, and Helminth-related Morbidity in Lake Victoria Fishing Communities, Uganda: Results From the LaVIISWA Cluster-randomized Trial. *Clinical Infectious Diseases* 2018:ciy761-ciy61. doi: 10.1093/cid/ciy761

29. CDC. Centers for Disease Control and Prevention, vaccines and immunizations.

30. WHO. Information sheet observed rate of vaccine reactions Bacille Calmette-Guérin (BCG) vaccine. 2012

31. Arts RJW, Moorlag S, Novakovic B, et al. BCG Vaccination Protects against Experimental Viral Infection in Humans through the Induction of Cytokines Associated with Trained Immunity. *Cell host & microbe* 2018;23(1):89-100.e5. doi: 10.1016/j.chom.2017.12.010 [published Online First: 2018/01/13]

For peer review only - http://bmjopen.bmj.com/site/about/guidelines.xhtml

MRC/UVRI and LSHTM Uganda Research Unit

Medical Research Council — Uganda Virus Research Institute — LONDON SCHOOL of HYGIENE &TROPICAL MEDICINE

Population differences in vaccine responses: the effect of intensive treatment for schistosomiasis on response to vaccines among island adolescents

Short Title: POPVAC Project A

Information Sheet for participants 9 – 12 years

1. Why are we meeting with you?

We are inviting you to take part in a research which is being done by a team from Uganda Virus Research Institute (UVRI), working together with colleagues from the London School of Hygiene and Tropical Medicine, UK. We are meeting you because you are aged between 9 and 12 years and study in a primary school on Koome island. After we tell you about it, we will ask if you'd like to be in this study or not. Only If you agree to take part, will you sign the assent form to show us that you are happy to do so.

The people leading this research study are Professor Alison Elliott and Dr Ludoviko Zirimenya.

2. Why are we doing this research?

We want to **find out whether treating Bilharzia much more frequently (a total of 7 times in a year) can get rid of it completely and whether this will improve vaccine responses.**

In the whole study, there will be about 480 children from schools of Koome islands.

3. What is going to happen in this research study?

Only if you agree, a full health check-up that will involve taking stool, urine and blood samples will be done. If everything is okay, the following will be done:

You will be put into one of the two **Bilharzia treatment** groups by chance

- One group will be treated for Bilharzia using praziquantel (the recommended medicine for Bilharzia) seven times in the year, and an eighth time after the end of the study.
- The other group will be treated for Bilharzia using praziquantel once in the year, and a second time after the end of the study.

You will be immunised with **five (5) vaccines**. These are:

- BCG vaccine. This is intended to protect against tuberculosis.
- Yellow fever vaccine. This protects against Yellow Fever an infection carried by mosquitoes.
- HPV vaccine. Human Papilloma Virus (HPV) causes growths in private parts and cancer in girls and boys.
- Tetanus and diphtheria vaccines. Tetanus and diphtheria are bacterial infections that cause very serious disease.
- Typhoid vaccine. Typhoid is a serious form of fever which is spread through contaminated food.

These immunisations will be given on different times during the entire duration of the study.

UGANDA VIRUS RESEARCH INSTITUTE ENTEBBE
RESEARCH ETHICS COMMITTEE
APPROVED
0 5 SEP 2020
P. O. BOX 49, ENTEBBE
UGANDA

For peer review only - http://bmjopen.bmj.com/site/about/guidelines.xhtml

Medical Research Council MRC | Uganda Virus Research Institute | LONDON SCHOOL of HYGIENE &TROPICAL MEDICINE

Most of the vaccines will be injected into your upper arm (either right or left side). The typhoid vaccine is an oral vaccine: it is in the form of capsules which are swallowed. You will be given one capsule per day for three alternating days.

You will be asked to give **blood samples**

We will ask you to give blood samples before and after you are given the vaccines. The amount of blood that we will take at each visit is completely safe and will vary between 4 and 27 millilitres (1 teaspoon and 5 and a half teaspoons) at the different visits.

You will be asked to give **stool and urine samples**

At some visits we will ask you for additional stool and urine samples for testing to check whether the worm infections have responded to treatment or (for girls) whether you have become pregnant.

Some of you will be asked to take part in a special **sugar test** of absorption from the intestines

We think that worm infections may make our intestines a little bit leaky, and this might explain some of their effects. We can test this using a special sugar drink. We will ask about 200 people to do this additional test. If you are asked to do this, it will be done about 8 weeks after your first vaccine is given. You will be asked not to eat overnight or in the morning of the test day. Then you will be given a special drink that contains sugars early in the morning. Afterwards you will also be asked to drink a litre of water (equal to two Rwenzori bottles) over the following few hours. All the urine that you pass during the next five hours will be collected. The volume (amount) of urine will be measured and the amount of sugar in it will be tested, to find out how much of the sugar your body has absorbed.

4. What will the blood, stool and urine samples be used for?

The samples will be used to test for infections, including HIV, malaria and worms, to test for anaemia (the strength of your blood) and to test for pregnancy (among girls). The blood samples will also be used to test how your body responds to vaccines. For some participants, the sugar test will be used to test absorption from the intestines.

5. Will taking part in this study harm me?

We do not expect this to harm you, though you will experience the following:

- You will need to take time off classes during each visit by the study team.
- Taking blood samples is not expected to cause any problem for you, apart from the discomfort or pain, bruising or bleeding, redness at the place where the needle goes into the skin, but this will go away in a few days.
- During the sugar test, you will be asked not to eat overnight and during the morning of the test so you may get hungry (although water and a snack will be provided). This may make you feel a little sick and stools may become a bit loose.
- If you are a girl, even if you know you could not be pregnant, we will do a pregnancy test. We have to be quite sure. You must as well promise that you can avoid getting pregnant while taking part in this study.

UGANDA VIRUS RESEARCH INSTITUTE ENTEBBE
RESEARCH ETHICS COMMITTEE
APPROVED
05 SEP 2020
P. O. BOX 49, ENTEBBE
UGANDA

For peer review only - http://bmjopen.bmj.com/site/about/guidelines.xhtml

MRC/UVRI and LSHTM Uganda Research Unit

Medical Research Council MRC | Uganda Virus Research Institute | LONDON SCHOOL of HYGIENE &TROPICAL MEDICINE

- Treatment of Bilharzia with praziquantel may make you feel dizzy or sick, give you abdominal pain or diarrhoea, or occasionally cause an itchy rash. The research team will have medicines available to help you if you have a strong reaction to the treatment.
- All the study vaccines are known to be safe. However, even approved vaccines may very occasionally cause a serious reaction, such as an allergic reaction. The research doctors and nurses will be available to help if this happens.

6. <u>Will the study help me?</u>

The treatment for Bilharzia is likely to be good for you, whichever group you are put in. The vaccines are likely to help you by protecting you from infectious diseases. The research results may help all people in the world, because in the end we may get a better understanding whether treating Bilharzia much more frequently (a total of 7 times in a year) can get rid of it completely and whether this will improve vaccine responses.

7. <u>What is the cost of taking part in the trial?</u>

There is no cost to participate in this trial. You will receive a soft drink and the gift of a pen or other simple school material on days when blood samples are drawn, and the gift of a T-shirt at the end of the study when everything is done, in appreciation of your contribution to the work.

8. <u>What happens if I refuse to take part?</u>

It is very important for you to know that you do not have to take part in the research, the choice is yours. No-one will be upset if you decide not to take part. If you agree to take part and later decide that you do not want to take part anymore, that is also okay.

9. <u>What happens if something goes wrong?</u>

The researchers will make every effort to ensure your safety and well-being. MRC/UVRI and LSHTM has a specialist insurance policy in place which would help to pay for treatment if any harm came to you as a result of taking part in the research.

10. <u>Who will have access to your information and samples from this research?</u>

Only research staff trained to keep the information confidential will have access to the records. Your name will be removed from the records and samples, so no-one will be able to find out information about you from our records.

11. <u>Who has reviewed the trial?</u>

This trial has been reviewed by the Uganda National Council for Science and Technology, The Uganda National Drug Authority, the Uganda Virus Research Institute Research and Ethics Committee the ethics committee of the London School of Hygiene and Tropical Medicine in the United Kingdom.

You can find out more about the study at any time by asking one of the members of the research team. You may also contact Professor Alison Elliott (telephone: 0417 704000) who is in charge of the study, or the Chairperson of the Ethics Committee for the Uganda Virus Research Institute on 0414 321962.

UGANDA VIRUS RESEARCH INSTITUTE ENTEBBE
RESEARCH ETHICS COMMITTEE
APPROVED
05 SEP 2020
P.O. BOX 49, ENTEBBE
UGANDA

MRC/UVRI and LSHTM Uganda Research Unit

MRC Medical Research Council · Uganda Virus Research Institute · LONDON SCHOOL of HYGIENE &TROPICAL MEDICINE

Population differences in vaccine responses: the effect of intensive treatment for schistosomiasis on response to vaccines among island adolescents

Short Title: POPVAC Project A

Participant Assent

A copy of this form will be given to you. Please keep a copy of the form because it contains the information that was discussed with you and you may want to look at it again.

AGREEMENT TO TAKE PART:

I have read and understood (or been read to and understand) the information sheet for this study. My questions have been answered. I understand that taking part in the study is voluntary. I understand that at any time I may withdraw from this study without giving a reason. I agree to participate in this study.

Name: ______________________________ **PVA ID: |A|___|___|___|___|**
(please write your name in capital letters here if you agree)

Signature: ______________________________ **Date:**__________________
(please sign or write your name here if you agree; or use a thumbprint)

What if I have any questions?
If you have any questions about your participation in this study, please feel free to ask any member of the research team at any time. If you prefer, you may speak to the principal investigator for this study (Professor Alison Elliott, telephone 0417 704000).

What if we want to ask someone independent anything about this research, or have any questions about your rights as a research participant? You may speak with the Chairman of the Science and Ethics Committee at Uganda Virus Research Institute on 0414 321962.

UGANDA VIRUS RESEARCH INSTITUTE ENTEBBE
RESEARCH ETHICS COMMITTEE
APPROVED
05 SEP 2020
P. O. BOX 49, ENTEBBE
UGANDA

For peer review only - http://bmjopen.bmj.com/site/about/guidelines.xhtml

PVA ID: |A|___|___|___|___|

Witness:

I have read the participant information sheet and the assent statement above

to:.. (PRINT NAME OF PARTICIPANT) in a language which he/she understands. I believe that he/she gives assent to take part in the study.

....	....	....
**Witness name**	**Signature**	**Date**

Witness required only for those using a thumb print instead of the final signature, or unable to read the information and consent form, or if the person taking consent does not speak the participant's language. The witness must not be a member of the research staff or a study participant. The witness must be present for the whole consent process.

Person taking the assent:

....	....	....
**Researcher name**	**Signature**	**Date**

Comment:

UGANDA VIRUS RESEARCH INSTITUTE ENTEBBE
RESEARCH ETHICS COMMITTEE
APPROVED
VALID UNTIL
05 SEP 2020
P. O. BOX 49, ENTEBBE
UGANDA

For peer review only - http://bmjopen.bmj.com/site/about/guidelines.xhtml

MRC/UVRI and LSHTM Uganda Research Unit

MRC Medical Research Council | Uganda Virus Research Institute | LONDON SCHOOL of HYGIENE &TROPICAL MEDICINE

Population differences in vaccine responses: the effect of intensive treatment for schistosomiasis on response to vaccines among island adolescents

Short Title: POPVAC Project A

Information Sheet for participants aged 13 to 17 years

We are inviting you to join in some research which is being done by a team from Uganda Virus Research Institute (UVRI), working together with colleagues from the London School of Hygiene and Tropical Medicine, UK. This form will tell you about the research. If you have any questions, please ask the person who is telling you about this research. Then you can decide whether you want to take part or not. There is no need to take part unless you really wish to. If you agree to take part, you will need to sign the assent form to show us that you are happy to do so. If you decide you do not want to take part, do not sign the assent form.

The people leading this research study are Professor Alison Elliott and Dr Ludoviko Zirimenya.

1. What this research is about, and the reason for doing this research.

This research is about how worm infections like Bilharzia "switch off" the body's defence systems and how this affects vaccine responses. **We want to find out whether treating Bilharzia much more frequently (a total of 7 times in a year) can get rid of it completely and whether this will improve vaccine responses.**

2. Why have I been asked to take part in this study?

You have been asked because you are attending primary school in Koome islands.

3. What is going to happen in this research study?

Only if you agree, a comprehensive health check-up that will involve taking stool, urine and blood samples will be done. If everything is okay, the following will be done:

You will be put into one of the two **Bilharzia treatment** groups by luck

- One group will be treated for Bilharzia using praziquantel (the recommended medicine for Bilharzia) seven times in the year, and an eighth time after the end of the study.
- The other group will be treated for Bilharzia using praziquantel once in the year, and a second time after the end of the study.

You will be immunised with **several vaccines**. These are the vaccines:

- BCG vaccine. This is intended to protect against tuberculosis.
- Yellow fever vaccine. This protects against Yellow Fever virus carried by mosquitoes.
- HPV vaccine. Human Papilloma Virus (HPV) causes growths in private parts and cancer in girls and boys.
- Tetanus and diphtheria vaccines. Tetanus and diphtheria are bacterial infections that cause very serious disease.
- Typhoid vaccine. Typhoid is a serious form of fever which is spread through contaminated food.

These immunisations will be given on three different days.

UGANDA VIRUS RESEARCH INSTITUTE ENTEBBE
RESEARCH ETHICS COMMITTEE
APPROVED
VALID UNTIL
05 SEP 2020
P. O. BOX 49, ENTEBBE
UGANDA

For peer review only - http://bmjopen.bmj.com/site/about/guidelines.xhtml

MRC/UVRI and LSHTM Uganda Research Unit

Medical Research Council MRC | Uganda Virus Research Institute | LONDON SCHOOL of HYGIENE &TROPICAL MEDICINE

Most of the vaccines will be injected into your upper arm (either right or left side). This will be a bit painful, as for any injection of medicine that you may have had, and will feel a bit sore over the next week or so. The typhoid vaccine is an oral vaccine: it is in the form of capsules which are swallowed. You will be given one capsule per day for three alternating days.

You will be asked to give **blood samples**

We will ask you to give blood samples before and after you are given the vaccines. The amount of blood that we will take at each visit is completely safe and will vary between 4 and 27 millilitres (1 teaspoon and 5 and a half teaspoons) at the different visits

You will be asked to give **stool and urine samples**

At some visits we will ask you for additional stool and urine samples for testing to check whether the worm infections have responded to treatment or (for girls) whether you have become pregnant.

Some of you will be asked to take part in a special **sugar test** of absorption from the intestines

We think that worm infections may make our intestines a little bit leaky, and this might explain some of their effects. We can test this using a special sugar drink. We will ask about 200 people to do this additional test. If you are asked to do this, it will be done about 8 weeks after your first vaccine is given. You will be asked not to eat overnight or in the morning of the test day. Then you will be given a special drink that contains sugars early in the morning. Afterwards you will also be asked to drink a litre of water (equal to two Rwenzori bottles) over the following few hours. All the urine that you pass during the next five hours will be collected. The volume (amount) of urine will be measured and the amount of sugar in it will be tested, to find out how much of the sugar your body has absorbed.

4. What will the blood, stool and urine samples be used for?

The samples will be used to test for infections, including HIV, malaria and worms, to test for anaemia (the strength of your blood) and to test for pregnancy (among girls). The blood samples will also be used to test how your body responds to vaccines. For some participants, the sugar test will be used to test absorption from the intestines.

5. How many people will take part in the study, and how long will it last?

The whole study will enrol 480 people, and the study will last for about two years.

6. What are the risks of participating in this trial?

You will need to take time off classes during each visit by the study team.

Taking blood samples is not expected to cause any problem for you, apart from the discomfort or pain, bruising or bleeding, redness at the place where the needle goes into the skin, but this will go away in a few days.

During the sugar test you will be asked not to eat overnight and during the morning of the test so you may get hungry (although water and a snack will be provided). Most people have no problems with this test although a few may feel a little sick and stools may become a bit loose.

UGANDA VIRUS RESEARCH INSTITUTE ENTEBBE
RESEARCH ETHICS COMMITTEE
APPROVED
05 SEP 2020
P. O. BOX 49, ENTEBBE
UGANDA

For peer review only - http://bmjopen.bmj.com/site/about/guidelines.xhtml

MRC/UVRI and LSHTM Uganda Research Unit

MRC Medical Research Council · Uganda Virus Research Institute · LONDON SCHOOL of HYGIENE &TROPICAL MEDICINE

If you are a girl, even if you know you could not be pregnant. We have to be quite sure. We will test for pregnancy at the beginning of the study and on each immunisation day. You must as well promise that you can avoid getting pregnant while taking part in this study.

Treatment of Bilharzia with praziquantel may make you feel dizzy or sick, give you abdominal pain or diarrhoea, or occasionally cause an itchy rash. This is most likely caused by your body's response to the worms as they are being killed by the medicine. The research team will have medicines available to help you if you have a strong reaction to the treatment.

All the study vaccines are known to be safe though not often, may cause a serious reaction, such as an allergic reaction. The research doctors and nurses will be available to help if this happens.

7. What are the benefits of taking part in this trial?

The treatment for Bilharzia is likely to be good for you, whichever group you are put in. The vaccines are likely to help you by protecting you from infectious diseases.

Also, you will be helping us to find out why vaccines sometimes don't work so well in countries like Uganda, and whether vaccines work better if worms are treated first. This may help other people in the future.

8. What is the cost of taking part in the trial?

There is no cost to participate in this trial. You will receive a soft drink and the gift of a pen or other simple school material on days when blood samples are drawn, and the gift of a T-shirt at the end of the study when everything is done, in appreciation of your contribution to the work.

9. What happens if I refuse to take part?

It is very important for you to know that you do not have to take part in the research, the choice is yours. No-one will be upset if you decide not to take part. If you agree to take part and later decide that you do not want to take part anymore, that is also okay.

10. What happens if something goes wrong?

You will be making an important contribution to medical research. The researchers will make every effort to ensure your safety and well-being. MRC/UVRI and LSHTM has a specialist insurance policy in place which would help to pay for treatment if any harm came to you as a result of taking part in the research.

11. Who will have access to my information and samples from this research?

All our research records are stored securely in rooms with restricted access and on password protected computers. Only research staff trained to keep the information confidential will have access to the records. Your name will be removed from the records, so no-one will be able to find out information about you from our records.

12. Who has reviewed the trial?

This trial has been reviewed by the Uganda National Council for Science and Technology, Uganda National Drug Authority the Uganda Virus Research Institute Research and Ethics Committee the ethics committee of the London School of Hygiene and Tropical Medicine in the United Kingdom.

UGANDA VIRUS RESEARCH INSTITUTE
RESEARCH ETHICS COMMITTEE
APPROVED
VALID UNTIL
05 SEP 2020
P. O. BOX 49, ENTEBBE
UGANDA

For peer review only - http://bmjopen.bmj.com/site/about/guidelines.xhtml

Medical Research Council MRC
Uganda Virus Research Institute
LONDON SCHOOL of HYGIENE &TROPICAL MEDICINE

You can find out more about the study at any time by asking one of the members of the research team. You may also contact Professor Alison Elliott (telephone: 0417 704000) who is in charge of the study, or the Chairperson of the Ethics Committee for the Uganda Virus Research Institute on 0414 321962.

UGANDA VIRUS RESEARCH INSTITUTE ENTEBBE
RESEARCH ETHICS COMMITTEE
APPROVED
05 SEP 2020
P. O. BOX 49, ENTEBBE
UGANDA

For peer review only - http://bmjopen.bmj.com/site/about/guidelines.xhtml

MRC/UVRI and LSHTM Uganda Research Unit

MRC Medical Research Council | Uganda Virus Research Institute | LONDON SCHOOL of HYGIENE &TROPICAL MEDICINE

Population differences in vaccine responses: the effect of intensive treatment for schistosomiasis on response to vaccines among island adolescents

Short Title: POPVAC Project A

Participant Assent

A copy of this form will be given to you. Please keep a copy of the form because it contains the information that was discussed with you and you may want to look at it again.

AGREEMENT TO TAKE PART:

I have read and understood (or been read to and understand) the information sheet for this study. My questions have been answered. I understand that taking part in the study is voluntary. I understand that at any time I may withdraw from this study without giving a reason. I agree to participate in this study.

Name: ______________________________ **PVA ID:** |A|___|___|___|___|
(please write your name in capital letters here if you agree)

Signature: ______________________________ **Date:**________________
(please sign or write your name here if you agree; or use a thumbprint)

What if I have any questions?
If you have any questions about your participation in this study, please feel free to ask any member of the research team at any time. If you prefer, you may speak to the principal investigator for this study (Professor Alison Elliott, telephone 0417 704000).

What if we want to ask someone independent anything about this research, or have any questions about your rights as a research participant? You may speak with the Chairman of the Science and Ethics Committee at Uganda Virus Research Institute on 0414 321962.

UGANDA VIRUS RESEARCH INSTITUTE ENTEBBE
RESEARCH ETHICS COMMITTEE
APPROVED
05 SEP 2020
P. O. BOX 49, ENTEBBE
UGANDA

For peer review only - http://bmjopen.bmj.com/site/about/guidelines.xhtml

PVA ID: |A|___|___|___|___|

Witness:

I have read the participant information sheet and the assent statement above

to:.. (PRINT NAME OF PARTICIPANT) in a language which he/she understands. I believe that he/she gives assent to take part in the study.

....	....	....
**Witness name**	**Signature**	**Date**

Witness required only for those using a thumb print instead of the final signature, or unable to read the information and consent form, or if the person taking consent does not speak the participant's language. The witness must not be a member of the research staff or a study participant. The witness must be present for the whole consent process.

Person taking the assent:

....	....	....
**Researcher name**	**Signature**	**Date**

Comment:

UGANDA VIRUS RESEARCH INSTITUTE ENTEBBE
RESEARCH ETHICS COMMITTEE
APPROVED
05 SEP 2020
P. O. BOX 49, ENTEBBE
UGANDA

For peer review only - http://bmjopen.bmj.com/site/about/guidelines.xhtml

MRC/UVRI and LSHTM Uganda Research Unit

Medical Research Council MRC | Uganda Virus Research Institute | LONDON SCHOOL of HYGIENE &TROPICAL MEDICINE

Population differences in vaccine responses: the effect of intensive treatment for schistosomiasis on response to vaccines among island adolescents

Short Title: POPVAC Project A

Information Sheet for emancipated or mature minors

We are inviting you to join in some research which is being done by a team from Uganda Virus Research Institute (UVRI), working together with colleagues from the London School of Hygiene and Tropical Medicine, UK. This form will tell you about the research. If you have any questions, please ask the person who is telling you about this research. Then you can decide whether you want to take part or not. If you agree to take part, you will need to sign the consent form to show us that you are happy to do so. If you decide that you do not want to take part, do not sign the consent form.

The people leading this research study are Professor Alison Elliott and Dr Ludoviko Zirimenya.

1. <u>What is this research about?</u>

This research is about how worm infections like Bilharzia "switch off" the body's defence systems and how this affects vaccine responses. **We want to find out whether treating Bilharzia much more frequently (a total of 7 times in a year) can get rid of it completely and whether this will improve vaccine responses.**

2. <u>Why have I been asked to take part in this study?</u>

You have been asked because you are attending primary school in Koome islands.

3. <u>What is going to happen in this research study?</u>

Only if you agree, you will have a comprehensive health check-up that will involve taking stool, urine and blood samples. If everything is okay, the following will be done:

<u>You will be put into one of the two **Bilharzia treatment** groups by chance</u>

One group will be treated for Bilharzia using praziquantel (the recommended medicine for Bilharzia) <u>seven times</u> in the year, and an eighth time after the end of the study.

The other group will be treated for Bilharzia using praziquantel <u>once</u> in the year, and a second time after the end of the study.

UGANDA VIRUS RESEARCH INSTITUTE ENTEBBE
RESEARCH ETHICS COMMITTEE
APPROVED
03 SEP 2020
P. O. BOX 49, ENTEBBE
UGANDA

For peer review only - http://bmjopen.bmj.com/site/about/guidelines.xhtml

You will be immunised with **five (5) vaccines.** These are:

- **BCG vaccine.** This is intended to protect against tuberculosis.
- **Yellow fever vaccine.** This protects against Yellow Fever an infection carried by mosquitoes.
- **HPV vaccine.** Human Papilloma Virus (HPV) causes growths in private parts and cancer in girls and boys.
- **Tetanus and diphtheria vaccines.** Tetanus and diphtheria are bacterial infections that cause very serious disease.
- **Typhoid vaccine.** Typhoid is a serious form of fever which is spread through contaminated food.

These immunisations will be given on different times during the entire duration of the study.

Most of the vaccines will be injected into your upper arm (either right or left side). The typhoid vaccine is an oral vaccine: it is in the form of capsules which are swallowed. You will be given one capsule per day for three alternating days.

You will be asked to give **blood samples**

We will ask you to give blood samples before and after you are given the vaccines. The amount of blood that we will take at each visit is completely safe and will vary between 4 and 27 millilitres (1 teaspoon and 5 and a half teaspoons) at the different visits.

You will be asked to give **stool and urine samples**

At some visits we will ask you for additional stool and urine samples for testing to check whether the worm infections have responded to treatment or (for girls) whether you have become pregnant.

Some of you will be asked to take part in a special **sugar test** of absorption from the intestines

We think that worm infections may make our intestines a little bit leaky, and this might explain some of their effects. We can test this using a special sugar drink. We will ask about 200 people to do this additional test. If you are asked to do this, it will be done about 8 weeks after your first vaccine is given. You will be asked not to eat overnight or in the morning of the test day. Then you will be given a special drink that contains sugars early in the morning. Afterwards you will also be asked to drink a litre of water (equal to two Rwenzori bottles) over the following few hours. All the urine that you pass during the next five hours will be collected. The volume (amount) of urine will be measured and the amount of sugar in it will be tested, to find out how much of the sugar your body has absorbed.

UGANDA VIRUS RESEARCH INSTITUTE ENTEBBE
RESEARCH ETHICS COMMITTEE
APPROVED
05 SEP 2020
P. O. BOX 49, ENTEBBE
UGANDA

For peer review only - http://bmjopen.bmj.com/site/about/guidelines.xhtml

MRC/UVRI and LSHTM Uganda Research Unit

Medical Research Council MRC | Uganda Virus Research Institute | LONDON SCHOOL of HYGIENE & TROPICAL MEDICINE

4. What will the blood, stool and urine samples be used for?

The samples will be used to test for infections, including HIV, malaria and worms, to test for anaemia (the strength of your blood) and to test for pregnancy (among girls). The blood samples will also be used to test how your body responds to vaccines.

5. How many people will take part in the study, and how long will it last?

The whole study will enrol 480 people, and the study will last for about two years.

6. What are the risks of participating in this trial?

- You will need to take time off classes during each visit by the study team.
- Taking blood samples is not expected to cause any problem for you, apart from the discomfort or pain, bruising or bleeding, redness at the place where the needle goes into the skin, but this will go away in a few days.
- During the sugar test, you will be asked not to eat overnight and during the morning of the test so you may get hungry (although water and a snack will be provided). This may make you feel a little sick and stools may become a bit loose.
- If you are a girl, even if you know you could not be pregnant, we will do a pregnancy test. We have to be quite sure. You must as well promise that you can avoid getting pregnant while taking part in this study.
- Treatment of Bilharzia with praziquantel may make you feel dizzy or sick, give you abdominal pain or diarrhoea, or occasionally cause an itchy rash. The research team will have medicines available to help you if you have a strong reaction to the treatment.
- All the study vaccines are known to be safe. However, very occasionally even approved vaccines may cause a serious reaction, such as an allergic reaction. The research doctors and nurses will be available to help if this happens.

7. What are the benefits of taking part in this trial?

The treatment for Bilharzia is likely to be good for you, whichever group you are put in. The vaccines are likely to help you by protecting you from infectious diseases.

Also, you will be helping us to find out why vaccines sometimes don't work so well in countries like Uganda, and whether vaccines work better if worms are treated first. This may help other people in the future.

UGANDA VIRUS RESEARCH INSTITUTE ENTEBBE
RESEARCH ETHICS COMMITTEE
APPROVED
05 SEP 2020
P. O. BOX 49, ENTEBBE
UGANDA

For peer review only - http://bmjopen.bmj.com/site/about/guidelines.xhtml

8. What is the cost of taking part in the trial?

There is no cost to participate in this trial. You will receive a soft drink and the gift of a pen or other simple school material on days when blood samples are drawn, and the gift of a T-shirt at the end of the study when everything is done, in appreciation of your contribution to the work.

9. What happens if I refuse to take part?

It is very important for you to know that you do not have to take part in the research, the choice is yours. No-one will be upset if you decide not to take part. If you agree to take part and later decide that you do not want to take part anymore, that is also okay.

10. What happens if something goes wrong?

The researchers will make every effort to ensure your safety and well-being. MRC/UVRI and LSHTM has a specialist insurance policy in place which would help to pay for treatment if any harm came to you as a result of taking part in the research.

11. Who will have access to my information and samples from this research?

All our research records and samples are stored securely in rooms with restricted access and on password protected computers. Only research staff trained to keep the information confidential will have access to the them. Your name will be removed from the records, so no-one will be able to find out information about you from our records.

12. Who has reviewed the trial?

This trial has been reviewed by the Uganda National Council for Science and Technology, The Uganda National Drug Authority, the Uganda Virus Research Institute Research and Ethics Committee the ethics committee of the London School of Hygiene and Tropical Medicine in the United Kingdom.

You can find out more about the study at any time by asking one of the members of the research team. You may also contact Professor Alison Elliott (telephone: 0417 704000) who is in charge of the study, or the Chairperson of the Ethics Committee for the Uganda Virus Research Institute on 0414 321962.

UGANDA VIRUS RESEARCH INSTITUTE ENTEBBE
RESEARCH ETHICS COMMITTEE
APPROVED
05 SEP 2020
P. O. BOX 49, ENTEBBE
UGANDA

For peer review only - http://bmjopen.bmj.com/site/about/guidelines.xhtml

MRC/UVRI and LSHTM Uganda Research Unit

MRC Medical Research Council | Uganda Virus Research Institute | LONDON SCHOOL of HYGIENE &TROPICAL MEDICINE

Population differences in vaccine responses: the effect of intensive treatment for schistosomiasis on response to vaccines among island adolescents

Short Title: POPVAC Project A

Participant Consent

A copy of this form will be given to you. Please keep a copy of the form because it contains the information that was discussed with you and you may want to look at it again.

AGREEMENT TO TAKE PART:

I have read and understood (or been read to and understand) the information sheet for this study. My questions have been answered. I understand that taking part in the study is voluntary. I understand that at any time I may withdraw from this study without giving a reason. I agree to participate in this study.

Name: ______________________________ **PVA ID:**|A|___|___|___|___|

(please write your name in capital letters here if you agree)

Signature: ______________________________ **Date:**__________________

(please sign or write your name here if you agree; or use a thumbprint)

What if I have any questions?

If you have any questions about your participation in this study, please feel free to ask any member of the research team at any time. If you prefer, you may speak to the principal investigator for this study (Professor Alison Elliott, telephone 0417 704000).

What if we want to ask someone independent anything about this research, or have any questions about your rights as a research participant? You may speak with the Chairman of the Science and Ethics Committee at Uganda Virus Research Institute on 0414 321962.

UGANDA VIRUS RESEARCH INSTITUTE ENTEBBE
RESEARCH ETHICS COMMITTEE
APPROVED
05 SEP 2020
P. O. BOX 49, ENTEBBE
UGANDA

For peer review only - http://bmjopen.bmj.com/site/about/guidelines.xhtml

PVA ID|A|___|___|___|___|

Witness:

I have read the participant information sheet and the consent statement above

to:.. (PRINT NAME OF PARTICIPANT) in a language which he/she understands. I believe that he/she gives consent to take part in the study.

....	....	....
**Witness name**	**Signature**	**Date**

Witness required only for those using a thumb print instead of the final signature, or unable to read the information and consent form, or if the person taking consent does not speak the participant's language. The witness must not be a member of the research staff or a study participant. The witness must be present for the whole consent process.

Person taking the consent:

....	....	....
**Researcher name**	**Signature**	**Date**

Comment:

UGANDA VIRUS RESEARCH INSTITUTE ENTEBBE
RESEARCH ETHICS COMMITTEE
APPROVED
05 SEP 2020
P.O. BOX 49, ENTEBBE
UGANDA

For peer review only - http://bmjopen.bmj.com/site/about/guidelines.xhtml

MRC/UVRI and LSHTM Uganda Research Unit

Population differences in vaccine responses: the effect of intensive treatment for schistosomiasis on response to vaccines among island adolescents

Short Title: POPVAC Project A

Information Sheet for parents/guardians

We are inviting your child to join in some research which is being done by a team from Uganda Virus Research Institute (UVRI), working together with colleagues from the London School of Hygiene and Tropical Medicine, UK. This form will tell you about the research. If you have any questions, please ask the person who is telling you about this research. Then you can decide whether you want your child to take part or not. There is no need for your child to take part unless you really want him/her to do so. If you agree for your child to take part, you will need to sign the consent form to show us that you are happy for him/her to do so. If you decide that you do not want your child to take part, do not sign the consent form.

The people leading this research study are Professor Alison Elliott and Dr Ludoviko Zirimenya.

1. What this research is about, and the reason for doing this research?

Vaccines are a very important tool for preventing infectious diseases. They have saved many lives. Vaccines are usually made from a weakened or killed strain of the bacteria or viruses that cause infectious diseases, or from a part of the bacteria or viruses. Vaccines are designed to help our body's defence system to recognise infectious diseases before we actually meet them, so that we can defeat them more easily. **However, some vaccines seem to work less well in hot countries, near the equator (such as Uganda), than in cooler countries (such as the United Kingdom, the UK).** We want to find out why this is so.

Worm infections are much more common in warm countries (such as Uganda) than in cooler countries (such as the UK). Bilharzia (schistosomiasis) is a worm infection which is very common in Koome islands. Almost everyone in Koome has Bilharzia. Worms can live in our bodies for many years. To do this they have to be able to switch off some of our body's defence systems so that they will not be killed. **We think that this "switching off" of defence systems may also prevent some vaccines from working well.** If this is true then treating worms before giving vaccines might result in better responses to the vaccines.

UGANDA VIRUS RESEARCH INSTITUTE ENTEBBE RESEARCH ETHICS COMMITTEE
APPROVED
05 SEP 2020
P. O. BOX 49, ENTEBBE UGANDA

For peer review only - http://bmjopen.bmj.com/site/about/guidelines.xhtml

Light infections with Bilharzia may not be noticed but heavy infections can cause serious damage, especially to the liver. That is why the Ugandan government usually gives treatment for Bilharzia once a year in Ugandan schools. This usually reduces the number of Bilharzia worms but does not get rid of them. **We want to find out whether treating Bilharzia much more frequently (a total of 7 times in a year) can get rid of it completely and whether this will improve vaccine responses.**

2. Why has my child been asked to take part in this study?

Your child has been asked because he/she is attending primary school in Koome islands, in primary 1 to 6. Bilharzia infection is often heaviest in people of primary school age. Also, the government of Uganda offers vaccines to your child's age group that will help to protect them from infectious diseases later on. So it is important to know whether Bilharzia can affect these vaccines, and whether treating Bilharzia effectively is helpful.

3. What is going to happen in this research study?

Your child will have **a health check-up**

If you want your child to take part in this study, we will first check your child's health, and take stool samples, urine samples and some blood from a vein in your child's arm and do some tests. These will include tests for infections including HIV, malaria and worms, tests for anaemia (the strength of blood in your child's body), and tests for pregnancy if your child is a girl.

If everything is okay then we will enrol your child in the study. If something is not okay then we will either give your child the treatment that he/she needs, or tell you what to do.

Your child will be put into one of the two **Bilharzia treatment** groups

When your child is enrolled in the study we will put your child into one of two groups.

- One group will be treated for Bilharzia using praziquantel (the recommended medicine for Bilharzia) seven times in the year, and an eighth time after the end of the study.
- The other group will be treated for Bilharzia using praziquantel once in the year, and a second time after the end of the study.

The choice of groups will be done using a code generated by computer. This works so that your child is put into one group or the other by luck – this is like a lottery.

UGANDA VIRUS RESEARCH INSTITUTE ENTEBBE
RESEARCH ETHICS COMMITTEE
APPROVED
05 SEP 2020
P. O. BOX 49, ENTEBBE
UGANDA

For peer review only - http://bmjopen.bmj.com/site/about/guidelines.xhtml

<u>Your child will be immunised with **several vaccines**.</u> These are the vaccines:

- BCG vaccine. This is intended to protect against tuberculosis. Tuberculosis is very common in Uganda. Many people receive the BCG vaccine as a baby. BCG can be given again at school age and may help to protect against tuberculosis later on although the level of protection varies a lot between countries. We do not know how well it works in school children in Uganda.
- Yellow fever vaccine. This protects against Yellow Fever. Yellow fever is a virus carried by mosquitoes. Yellow fever disease affects the liver and causes fever and jaundice (yellow eyes). Outbreaks occur in Uganda and neighbouring countries from time to time – an outbreak is when a group of people falls sick around the same time.
- HPV vaccine. Human Papilloma Virus (HPV) causes warts. As well, some strains of HPV can cause cancer in the genital areas, especially on the cervix (the opening of the womb) in girls. HPV can also cause cancer of the penis in boys, and other cancers. The HPV vaccine reduces the risk of infection with dangerous HPV strains that cause cancer. In this way it reduces the risk of cancers. The Ugandan government recently started offering this vaccine to girls in primary 4. We will give this vaccine to you if you are a girl (in any class) and you have not received it already. We will also give this vaccine to boys because it can protect them against some cancers too.
- Tetanus and diphtheria vaccines. Tetanus and diphtheria are bacterial infections. The tetanus and diphtheria bacteria produce chemicals called toxins which cause diseases. Tetanus bacteria infect deep wounds and produce tetanus toxin which causes very serious muscle spasms. Tetanus can affect young babies if the wound where the umbilical cord is cut becomes infected. Immunising young women can protect their future babies too. Diphtheria causes a very serious throat infection. Tetanus and diphtheria immunisation is given to babies but booster immunisation is recommended by the Uganda government for school children also.
- Typhoid vaccine. Typhoid is a serious form of fever which is spread through contaminated food. Outbreaks occur in Uganda. Immunisation against typhoid can prevent this illness.

These immunisations will be given at three different time points. BCG will be given first. HPV vaccine, oral typhoid vaccine and yellow fever vaccine will be given four weeks later. A second dose of HPV vaccine and first dose of Tetanus/diphtheria vaccine will be given at 28 weeks after the BCG immunisation.

Most of the vaccines will be injected into your child's upper arm (either right or left). This will be a bit painful, as for any injection of medicine that you may have had, and will feel a bit sore over the next week or so. The BCG vaccine is likely to form a small swelling and then an ulcer

UGANDA VIRUS RESEARCH INSTITUTE ENTEBBE
RESEARCH ETHICS COMMITTEE
APPROVED
VALID UNTIL
05 SEP 2020
P.O. BOX 49, ENTEBBE
UGANDA

For peer review only - http://bmjopen.bmj.com/site/about/guidelines.xhtml

which may take quite some time (perhaps two to five months) to heal. The typhoid vaccine is an oral vaccine: it is in the form of capsules which are swallowed. Your child will be given one capsule per day for three alternating days.

Your child will be asked to give **blood samples**

To test your child's response to the vaccines we will ask your child to give blood samples before and after they are given the vaccines. The amount of blood that we will take at each visit is completely safe and will vary between 4 and 27 millilitres (1 teaspoon and 5 and a half teaspoons) at the different visits. In total we will ask your child to give blood samples at 7 different times during the study. The study will last for about two years.

Your child will be asked to give **stool and urine samples**

At some visits we will ask your child for additional stool and urine samples for testing to check whether the worm infections have responded to treatment or (for girls) whether your child has become pregnant.

Some of the children will be asked to take part in a special **sugar test** of absorption from the intestines

We think that worm infections may make our intestines a little bit leaky, and this might explain some of their effects. We can test this using a special sugar drink. We will ask about 200 children to do this additional test. If your child is asked to do this, it will be done about 8 weeks after the first vaccine is given. He/she will be asked not to eat overnight or in the morning of the test day. Then he/she will be given a special drink that contains sugars early in the morning. Afterwards he/she will also be asked to drink a litre of water (equal to two Rwenzori bottles) over the following few hours. All the urine that he/she passes during the next five hours will be collected. The volume (amount) of urine will be measured and the amount of sugar in it will be tested, to find out how much of the sugar his/her body has absorbed.

If you do not want your child to take part in the study, to have blood, urine and stool samples taken or to receive the treatment and vaccines, you can say no to your child's taking part in this study.

4. What will the blood, stool and urine samples be used for?

The samples will be used to test for infections, including HIV, malaria and worms, to test for anaemia (the strength of your child's blood) and to test for pregnancy (among girls). The blood samples will also be used to test how your child's body responds to vaccines.

UGANDA VIRUS RESEARCH INSTITUTE ENTEBBE
RESEARCH ETHICS COMMITTEE
APPROVED
VALID UNTIL
05 SEP 2020
P. O. BOX 49, ENTEBBE
UGANDA

For peer review only - http://bmjopen.bmj.com/site/about/guidelines.xhtml

5. How many people will take part in the study, and how long will it last?

The whole study will enrol 480 people, and the study will last for about two years.

6. What are the risks of participating in this trial?

Your child's time

Your child will need to take time off classes during each visit by the study team. There will be 12 visits altogether. Each visit will usually take about 30 minutes, but the first visit may take about two hours to give time for everything to be explained, and for a thorough check-up. The research team will work with teachers to avoid disturbing classes too much.

Blood samples

Taking blood samples is not expected to cause any problem for your child, apart from the discomfort or pain, bruising or bleeding, redness at the place where the needle goes into the skin, but this will go away in a few days. There is a very small chance that your child may get an infection or some swelling at this place – this almost never happens. Some people faint when their blood is taken. The person taking the blood will do all they can to prevent these things from happening.

Sugar test

During the sugar test your child will be asked not to eat overnight and during the morning of the test so he/she may get hungry (although water and a snack will be provided). Most people have no problems with this test although a few may feel a little sick and stools may become a bit loose.

Pregnancy

We do not know how some of these vaccines might affect a developing baby if they were given to someone who was pregnant. That is why we will do a pregnancy test, if your child is a girl, even if she knows she could not be pregnant. We have to be quite sure. We will test for pregnancy at the beginning of the study and on each immunisation day. We will not enrol your child in the study if she is pregnant at the start. We will not give your child the vaccines if she falls pregnant later.

Bilharzia treatment

Treatment of Bilharzia with praziquantel may make your child feel dizzy or sick, give your child abdominal pain or diarrhoea, or occasionally cause an itchy rash. This is most likely caused by

UGANDA VIRUS RESEARCH INSTITUTE ENTEBBE
RESEARCH ETHICS COMMITTEE
APPROVED
VALID UNTIL
05 SEP 2020
P.O. BOX 49, ENTEBBE
UGANDA

For peer review only - http://bmjopen.bmj.com/site/about/guidelines.xhtml

the body's response to the worms as they are being killed by the medicine. The research team will have medicines available to help your child if he/she has a strong reaction to the treatment.

Immunisations

All the vaccines that we will give have been used to protect very large numbers of people and are known to be safe. They are not expected to cause any major problems for your child. As mentioned, the BCG vaccine is expected to cause an ulcer and to heal slowly, leaving a scar. The injections will cause some pain at the time of injection and your child's arm will feel a bit sore for a day or two. Some people develop flu-like symptoms including headache and fever for a day or two. It is fine for your child to take painkillers like paracetamol (Panadol) for these symptoms. The typhoid vaccine, which your child will swallow as capsules, is not expected to cause your child any problem at all, although your child may experience some stomach pain, feeling sick, vomiting and (rarely) rash.

Very occasionally any vaccine can cause a serious reaction, such as an allergic reaction. The research doctors and nurses will be available to help if this happens.

7. What are the benefits of taking part in this trial?

The treatment for Bilharzia is likely to be good for your child, whichever group he or she is put in. The vaccines are likely to help your child by protecting him/her from infectious diseases.

Also, you and your child will be helping us to find out why vaccines sometimes don't work so well in countries like Uganda, and whether vaccines work better if worms are treated first. This may help other people in the future.

8. What is the cost of taking part in the trial?

There is no cost to participate in this trial. We will reimburse you (the parents) for the time you spend at meetings at school, 20,000/= (twenty thousand shillings) for each visit. Your child will receive a soft drink and the gift of a pen or other simple school material on days when blood samples are drawn, and the gift of a T-shirt at the end of the study when everything is done, in appreciation of his/her contribution to the work.

9. What happens if I refuse for my child to take part?

It is very important for you to know that your child does not have to take part in the research, the choice is yours and your child's. No-one will be upset if your child decides not to take part. The teachers will not be upset and the research team will not be upset. If you agree for your child to take part and later decide that you do not want him/her to take part anymore, that is also okay. Whatever happens, your child will still be able to receive the treatment for Bilharzia

UGANDA VIRUS RESEARCH INSTITUTE RESEARCH ETHICS COMMITTEE
APPROVED
VALID UNTIL
05 SEP 2020
P. O. BOX 49, ENTEBBE
UGANDA

For peer review only - http://bmjopen.bmj.com/site/about/guidelines.xhtml

and the immunisations for HPV and tetanus and diphtheria when they are provided by the government, if you wish.

10. Who will be able to see the results of tests done on my child's samples?

The research team will keep your child's results private. Only members of the research team will be able to see results and to know that they belong to your child. Your child's samples will be given a special code so that anyone who is working on them in research laboratories will not know they came from your child.

11. What happens if something goes wrong?

You and your child will be making an important contribution to medical research. The researchers will make every effort to ensure your child's safety and well-being. MRC/UVRI and LSHTM has a specialist insurance policy in place which would help to pay for treatment if any harm came to your child as a result of taking part in the research.

12. Who will have access to information from this research?

All our research records are stored securely in rooms with restricted access and on password protected computers. Only research staff trained to keep the information confidential will have access to the records. Your child's name will be removed from the records, so no-one will be able to find out information about your child from our records. The people who may review your child's records include Research Ethics Committees (Uganda Virus Research Institute Research Ethics Committee and the London School of Hygiene & Tropical Medicine Ethics Committee) the Uganda National Council Science and Technology, Study Monitors, Sponsor and the Uganda National Drug Authority. These organisations are there to ensure that your child's rights are protected and that the research is conducted properly and safely.

13. Who has reviewed the trial?

This trial has been reviewed by the Uganda National Council for Science and Technology, the Uganda Virus Research Institute Research and Ethics Committee, the ethics committee of the London School of Hygiene and Tropical Medicine in the United Kingdom. The Uganda National Drug Authority, which regulates the use of all medicines in Uganda, has granted permission to use the medicines and vaccines needed for this clinical trial.

You can find out more about the study at any time by asking one of the members of the research team. You may also contact Professor Alison Elliott (telephone: 0417 704000) who is in charge of the study, or the Chairperson of the Ethics Committee for the Uganda Virus Research Institute on 0414 321962.

UGANDA VIRUS RESEARCH INSTITUTE ENTEBBE
RESEARCH ETHICS COMMITTEE
APPROVED
VALID UNTIL
05 SEP 2020
P. O. BOX 49, ENTEBBE
UGANDA

MRC/UVRI and LSHTM Uganda Research Unit

MRC Medical Research Council | Uganda Virus Research Institute | LONDON SCHOOL of HYGIENE &TROPICAL MEDICINE

Population differences in vaccine responses: the effect of intensive treatment for schistosomiasis on response to vaccines among island adolescents

Short Title: POPVAC Project A

Parent/guardian consent

A copy of this form will be given to you. Please keep a copy of the form because it contains the information that was discussed with you and you may want to look at it again.

Participant (child) name: ______________________________ **PVA ID**|A|___|___|___|___|
(please write your child's name in capital letters here if you agree)

AGREEMENT BY PARENT OR GUARDIAN:

I have read and/or been fully explained the information sheet concerning my child's participation in this study and I understand what will be required if he or she takes part. I understand that my child's participation is voluntary. My questions concerning this study have been answered. I understand that at any time, I may withdraw my child from this study without giving a reason and without affecting his or her entitlement to government health care. I agree for my child to take part in this study.

Name: ______________________________________
(please write your name in capital letters here if you agree)

Signature: _________________________________ **Date:**________________
(please sign or write your name here if you agree; or use a thumbprint)

What if I have any questions?
If you have any questions about your participation in this study, please feel free to ask any member of the research team at any time. If you prefer, you may speak to the principal investigator for this study (Professor Alison Elliott, telephone 0417 704000).

What if we want to ask someone independent anything about this research, or have any questions about your rights as a research participant? You may speak with the Chairman of the Science and Ethics Committee at Uganda Virus Research Institute on 0414 321962.

UGANDA VIRUS RESEARCH INSTITUTE ENTEBBE
RESEARCH ETHICS COMMITTEE
APPROVED
05 SEP 2020
P. O. BOX 49, ENTEBBE
UGANDA

For peer review only - http://bmjopen.bmj.com/site/about/guidelines.xhtml

PVA ID | A | ___ | ___ | ___ | ___ |

Witness:

I have read the participant information sheet and the assent statement above

to:.. (PRINT NAME OF PARTICIPANT) in a language which he/she understands. I believe that he/she gives consent for his/her child to take part in the study.

....	....	....
**Witness name**	**Signature**	**Date**

Witness required only for those using a thumb print instead of the final signature, or unable to read the information and consent form, or if the person taking consent does not speak the participant's language. The witness must not be a member of the research staff or a study participant. The witness must be present for the whole consent process.

Person taking the consent:

....	....	....
**Researcher name**	**Signature**	**Date**

Comment:

UGANDA VIRUS RESEARCH INSTITUTE ENTEBBE
RESEARCH ETHICS COMMITTEE
APPROVED
VALID UNTIL
05 SEP 2020
P. O. BOX 49, ENTEBBE
UGANDA

For peer review only - http://bmjopen.bmj.com/site/about/guidelines.xhtml

UGANDA VIRUS RESEARCH INSTITUTE ENTEBBE
RESEARCH ETHICS COMMITTEE
APPROVED
05 SEP 2020
P. O. BOX 49, ENTEBBE
UGANDA

Reporting checklist for protocol of a clinical trial.

Based on the SPIRIT guidelines.

Instructions to authors

Complete this checklist by entering the page numbers from your manuscript where readers will find each of the items listed below.

Your article may not currently address all the items on the checklist. Please modify your text to include the missing information. If you are certain that an item does not apply, please write "n/a" and provide a short explanation.

Upload your completed checklist as an extra file when you submit to a journal.

In your methods section, say that you used the SPIRITreporting guidelines, and cite them as:

Chan A-W, Tetzlaff JM, Altman DG, Laupacis A, Gøtzsche PC, Krleža-Jerić K, Hróbjartsson A, Mann H, Dickersin K, Berlin J, Doré C, Parulekar W, Summerskill W, Groves T, Schulz K, Sox H, Rockhold FW, Rennie D, Moher D. SPIRIT 2013 Statement: Defining standard protocol items for clinical trials. Ann Intern Med. 2013;158(3):200-207

		Reporting Item	Page Number
**Administrative information**			
Title	#1	Descriptive title identifying the study design, population, interventions, and, if applicable, trial acronym	1
Trial registration	#2a	Trial identifier and registry name. If not yet registered, name of intended registry	2
Trial registration: data set	#2b	All items from the World Health Organization Trial Registration Data Set	n/a
Protocol version	#3	Date and version identifier	Information available at ISRCTN60517191
Funding	#4	Sources and types of financial, material, and other support	15
Roles and	#5a	Names, affiliations, and roles of protocol	15

For peer review only

responsibilities: contributorship		contributors	
Roles and responsibilities: sponsor contact information	#5b	Name and contact information for the trial sponsor	Information available at ISRCTN60517191
Roles and responsibilities: sponsor and funder	#5c	Role of study sponsor and funders, if any, in study design; collection, management, analysis, and interpretation of data; writing of the report; and the decision to submit the report for publication, including whether they will have ultimate authority over any of these activities	16
Roles and responsibilities: committees	#5d	Composition, roles, and responsibilities of the coordinating centre, steering committee, endpoint adjudication committee, data management team, and other individuals or groups overseeing the trial, if applicable (see Item 21a for data monitoring committee)	Information detailed in supplementary information file
**Introduction**			
Background and rationale	#6a	Description of research question and justification for undertaking the trial, including summary of relevant studies (published and unpublished) examining benefits and harms for each intervention	4
Background and rationale: choice of comparators	#6b	Explanation for choice of comparators	8
Objectives	#7	Specific objectives or hypotheses	6
Trial design	#8	Description of trial design including type of trial (eg, parallel group, crossover, factorial, single group), allocation ratio, and framework (eg, superiority, equivalence, non-inferiority, exploratory)	7
**Methods: Participants, interventions, and outcomes**			

For peer review only

Study setting	#9	Description of study settings (eg, community clinic, academic hospital) and list of countries where data will be collected. Reference to where list of study sites can be obtained	7
Eligibility criteria	#10	Inclusion and exclusion criteria for participants. If applicable, eligibility criteria for study centres and individuals who will perform the interventions (eg, surgeons, psychotherapists)	7
Interventions: description	#11a	Interventions for each group with sufficient detail to allow replication, including how and when they will be administered	8
Interventions: modifications	#11b	Criteria for discontinuing or modifying allocated interventions for a given trial participant (eg, drug dose change in response to harms, participant request, or improving / worsening disease)	Detailed in supplementary information file
Interventions: adherance	#11c	Strategies to improve adherence to intervention protocols, and any procedures for monitoring adherence (eg, drug tablet return; laboratory tests)	Detailed in supplementary information file
Interventions: concomitant care	#11d	Relevant concomitant care and interventions that are permitted or prohibited during the trial	Detailed in supplementary information file
Outcomes	#12	Primary, secondary, and other outcomes, including the specific measurement variable (eg, systolic blood pressure), analysis metric (eg, change from baseline, final value, time to event), method of aggregation (eg, median, proportion), and time point for each outcome. Explanation of the clinical relevance of chosen efficacy and harm outcomes is strongly recommended	10
Participant timeline	#13	Time schedule of enrolment, interventions (including any run-ins and washouts), assessments, and visits for participants. A schematic diagram is highly recommended (see Figure)	14<br>Also detailed in supplementary information file, Table S1

For peer review only

Sample size	#14	Estimated number of participants needed to achieve study objectives and how it was determined, including clinical and statistical assumptions supporting any sample size calculations	11
Recruitment	#15	Strategies for achieving adequate participant enrolment to reach target sample size	Detailed in supplementary information file
**Methods: Assignment of interventions (for controlled trials)**			
Allocation: sequence generation	#16a	Method of generating the allocation sequence (eg, computer-generated random numbers), and list of any factors for stratification. To reduce predictability of a random sequence, details of any planned restriction (eg, blocking) should be provided in a separate document that is unavailable to those who enrol participants or assign interventions	8
Allocation concealment mechanism	#16b	Mechanism of implementing the allocation sequence (eg, central telephone; sequentially numbered, opaque, sealed envelopes), describing any steps to conceal the sequence until interventions are assigned	9
Allocation: implementation	#16c	Who will generate the allocation sequence, who will enrol participants, and who will assign participants to interventions	8, 9
Blinding (masking)	#17a	Who will be blinded after assignment to interventions (eg, trial participants, care providers, outcome assessors, data analysts), and how	9
Blinding (masking): emergency unblinding	#17b	If blinded, circumstances under which unblinding is permissible, and procedure for revealing a participant's allocated intervention during the trial	Detailed in supplementary information file
**Methods: Data collection, management, and analysis**			

For peer review only

Data collection plan	#18a	Plans for assessment and collection of outcome, baseline, and other trial data, including any related processes to promote data quality (eg, duplicate measurements, training of assessors) and a description of study instruments (eg, questionnaires, laboratory tests) along with their reliability and validity, if known. Reference to where data collection forms can be found, if not in the protocol	10.<br><br>More details in supplementary information file
Data collection plan: retention	#18b	Plans to promote participant retention and complete follow-up, including list of any outcome data to be collected for participants who discontinue or deviate from intervention protocols	Detailed in supplementary information file
Data management	#19	Plans for data entry, coding, security, and storage, including any related processes to promote data quality (eg, double data entry; range checks for data values). Reference to where details of data management procedures can be found, if not in the protocol	13.<br><br>More information in the statistical analysis plan found at ISRCTN60517191
Statistics: outcomes	#20a	Statistical methods for analysing primary and secondary outcomes. Reference to where other details of the statistical analysis plan can be found, if not in the protocol	13.<br><br>More information in the statistical analysis plan found at ISRCTN60517191
Statistics: additional analyses	#20b	Methods for any additional analyses (eg, subgroup and adjusted analyses)	11.<br><br>More information in the statistical analysis plan found at ISRCTN60517191
Statistics: analysis population and missing data	#20c	Definition of analysis population relating to protocol non-adherence (eg, as randomised analysis), and any statistical methods to handle missing data (eg, multiple imputation)	Information in the statistical analysis plan found at ISRCTN60517191
**Methods: Monitoring**			

For peer review only

Data monitoring: formal committee	#21a	Composition of data monitoring committee (DMC); summary of its role and reporting structure; statement of whether it is independent from the sponsor and competing interests; and reference to where further details about its charter can be found, if not in the protocol. Alternatively, an explanation of why a DMC is not needed	Detailed in supplementary information file
Data monitoring: interim analysis	#21b	Description of any interim analyses and stopping guidelines, including who will have access to these interim results and make the final decision to terminate the trial	Detailed in supplementary information file
Harms	#22	Plans for collecting, assessing, reporting, and managing solicited and spontaneously reported adverse events and other unintended effects of trial interventions or trial conduct	Detailed in supplementary information file
Auditing	#23	Frequency and procedures for auditing trial conduct, if any, and whether the process will be independent from investigators and the sponsor	Detailed in supplementary information file
**Ethics and dissemination**			
Research ethics approval	#24	Plans for seeking research ethics committee / institutional review board (REC / IRB) approval	12
Protocol amendments	#25	Plans for communicating important protocol modifications (eg, changes to eligibility criteria, outcomes, analyses) to relevant parties (eg, investigators, REC / IRBs, trial participants, trial registries, journals, regulators)	11
Consent or assent	#26a	Who will obtain informed consent or assent from potential trial participants or authorised surrogates, and how (see Item 32)	12.<br>Also detailed in supplementary information file
Consent or assent: ancillary studies	#26b	Additional consent provisions for collection and use of participant data and biological specimens in ancillary studies, if applicable	Detailed in supplementary information file

For peer review only

Confidentiality	#27	How personal information about potential and enrolled participants will be collected, shared, and maintained in order to protect confidentiality before, during, and after the trial	Detailed in supplementary information file
Declaration of interests	#28	Financial and other competing interests for principal investigators for the overall trial and each study site	15
Data access	#29	Statement of who will have access to the final trial dataset, and disclosure of contractual agreements that limit such access for investigators	n/a
Ancillary and post trial care	#30	Provisions, if any, for ancillary and post-trial care, and for compensation to those who suffer harm from trial participation	Detailed in supplementary information file
Dissemination policy: trial results	#31a	Plans for investigators and sponsor to communicate trial results to participants, healthcare professionals, the public, and other relevant groups (eg, via publication, reporting in results databases, or other data sharing arrangements), including any publication restrictions	2, 13
Dissemination policy: authorship	#31b	Authorship eligibility guidelines and any intended use of professional writers	n/a
Dissemination policy: reproducible research	#31c	Plans, if any, for granting public access to the full protocol, participant-level dataset, and statistical code	13
**Appendices**			
Informed consent materials	#32	Model consent form and other related documentation given to participants and authorised surrogates	Supplementary files provided
Biological specimens	#33	Plans for collection, laboratory evaluation, and storage of biological specimens for genetic or molecular analysis in the current trial and for future use in ancillary studies, if applicable	n/a

None The SPIRIT checklist is distributed under the terms of the Creative Commons Attribution License CC-BY-ND 3.0. This checklist can be completed online using https://www.goodreports.org/, a tool made by the EQUATOR Network in collaboration with Penelope.ai

For peer review only

BMJ Open

The effect of intensive treatment for schistosomiasis on immune responses to vaccines among rural Ugandan island adolescents: randomised controlled trial protocol A for the 'POPulation differences in VACcine responses' (POPVAC) programme

Journal:	*BMJ Open*
Manuscript ID	bmjopen-2020-040426.R3
Article Type:	Protocol
Date Submitted by the Author:	09-Dec-2020

Complete List of Authors:	Nkurunungi, Gyaviira; MRC/UVRI and LSHTM Uganda Research Unit, Immunomodulation and Vaccines Programme<br>Zirimenya , Ludoviko ; MRC/UVRI and LSHTM Uganda Research Unit, Immunomodulation and Vaccines Programme<br>Nassuuna , Jacent; MRC/UVRI and LSHTM Uganda Research Unit, Immunomodulation and Vaccines Programme<br>Natukunda , Agnes ; MRC/UVRI and LSHTM Uganda Research Unit, Immunomodulation and Vaccines Programme<br>Kabuubi , Prossy ; MRC/UVRI and LSHTM Uganda Research Unit, Immunomodulation and Vaccines Programme<br>Niwagaba , Emmanuel ; MRC/UVRI and LSHTM Uganda Research Unit, Immunomodulation and Vaccines Programme<br>Oduru, Gloria; MRC/UVRI and LSHTM Uganda Research Unit, Immunomodulation and Vaccines Programme<br>Kabami , Grace ; MRC/UVRI and LSHTM Uganda Research Unit, Immunomodulation and Vaccines Programme<br>Amongin , Rebecca ; MRC/UVRI and LSHTM Uganda Research Unit, Immunomodulation and Vaccines Programme<br>Mutebe , Alex ; MRC/UVRI and LSHTM Uganda Research Unit, Immunomodulation and Vaccines Programme<br>Namutebi , Milly ; MRC/UVRI and LSHTM Uganda Research Unit, Immunomodulation and Vaccines Programme<br>Zziwa , Christopher ; MRC/UVRI and LSHTM Uganda Research Unit, Immunomodulation and Vaccines Programme<br>Amongi, Susan; MRC/UVRI and LSHTM Uganda Research Unit, Immunomodulation and Vaccines Programme<br>Ninsiima , Caroline ; MRC/UVRI and LSHTM Uganda Research Unit, Immunomodulation and Vaccines Programme<br>Onen, Caroline; MRC/UVRI and LSHTM Uganda Research Unit, Immunomodulation and Vaccines Programme<br>Akello , Florence ; MRC/UVRI and LSHTM Uganda Research Unit, Immunomodulation and Vaccines Programme<br>Sewankambo, Moses; MRC/UVRI and LSHTM Uganda Research Unit, Immunomodulation and Vaccines Programme<br>Kiwanuka, Samuel; MRC/UVRI and LSHTM Uganda Research Unit, Immunomodulation and Vaccines Programme<br>Kizindo , Robert ; MRC/UVRI and LSHTM Uganda Research Unit,

	Immunomodulation and Vaccines Programme<br>Kaweesa, James; Republic of Uganda Ministry of Health, Vector Control Division<br>Cose , Stephen ; MRC/UVRI and LSHTM Uganda Research Unit, Immunomodulation and Vaccines Programme; London School of Hygiene and Tropical Medicine Department of Clinical Research<br>Webb, Emily; London School of Hygiene & Tropical Medicine, MRC Tropical Epidemiology Group, Department of Infectious Disease Epidemiology<br>Elliott, Alison ; MRC/UVRI and LSHTM Uganda Research Unit, Immunomodulation and Vaccines Programme; London School of Hygiene and Tropical Medicine Department of Clinical Research

**Primary Subject Heading**:	Infectious diseases
Secondary Subject Heading:	Epidemiology, Immunology (including allergy), Public health
Keywords:	Infection control < INFECTIOUS DISEASES, PARASITOLOGY, Public health < INFECTIOUS DISEASES, Immunology < TROPICAL MEDICINE, Epidemiology < TROPICAL MEDICINE, Paediatric infectious disease & immunisation < PAEDIATRICS

SCHOLARONE™
Manuscripts

I, the Submitting Author has the right to grant and does grant on behalf of all authors of the Work (as defined in the below author licence), an exclusive licence and/or a non-exclusive licence for contributions from authors who are: i) UK Crown employees; ii) where BMJ has agreed a CC-BY licence shall apply, and/or iii) in accordance with the terms applicable for US Federal Government officers or employees acting as part of their official duties; on a worldwide, perpetual, irrevocable, royalty-free basis to BMJ Publishing Group Ltd ("BMJ") its licensees and where the relevant Journal is co-owned by BMJ to the co-owners of the Journal, to publish the Work in this journal and any other BMJ products and to exploit all rights, as set out in our licence.

The Submitting Author accepts and understands that any supply made under these terms is made by BMJ to the Submitting Author unless you are acting as an employee on behalf of your employer or a postgraduate student of an affiliated institution which is paying any applicable article publishing charge ("APC") for Open Access articles. Where the Submitting Author wishes to make the Work available on an Open Access basis (and intends to pay the relevant APC), the terms of reuse of such Open Access shall be governed by a Creative Commons licence – details of these licences and which Creative Commons licence will apply to this Work are set out in our licence referred to above.

Other than as permitted in any relevant BMJ Author's Self Archiving Policies, I confirm this Work has not been accepted for publication elsewhere, is not being considered for publication elsewhere and does not duplicate material already published. I confirm all authors consent to publication of this Work and authorise the granting of this licence.

For peer review only

The effect of intensive treatment for schistosomiasis on immune responses to vaccines among rural Ugandan island adolescents: randomised controlled trial protocol A for the 'POPulation differences in VACcine responses' (POPVAC) programme

Gyaviira Nkurunungi[1,¶,*], Ludoviko Zirimenya[1,¶], Jacent Nassuuna[1,¶], Agnes Natukunda[1,¶], Prossy N Kabuubi[1], Emmanuel Niwagaba[1], Gloria Oduru[1], Grace Kabami[1], Rebecca Amongin[1], Alex Mutebe [1], Milly Namutebi[1], Christopher Zziwa[1], Susan Amongi[1], Caroline Ninsiima[1], Caroline Onen[1], Florence Akello[1], Moses Sewankambo[1], Samuel Kiwanuka[1], Robert Kizindo[1], James Kaweesa[2], Stephen Cose[1,3], Emily L Webb[4], Alison M Elliott[1,3] **for the POPVAC trial team**

[1]Immunomodulation and Vaccines Programme, Medical Research Council/Uganda Virus Research Institute and London School of Hygiene and Tropical Medicine (MRC/UVRI and LSHTM) Uganda Research Unit, Entebbe, Uganda

[2]Vector Control Division, Ministry of Health, Kampala, Uganda

[3]Department of Clinical Research, London School of Hygiene and Tropical Medicine, London, United Kingdom

[4]MRC Tropical Epidemiology Group, Department of Infectious Disease Epidemiology, London School of Hygiene and Tropical Medicine, London, United Kingdom

¶These authors contributed equally

***Correspondence:** Gyaviira Nkurunungi; Gyaviira.Nkurunungi@mrcuganda.org

For peer review only

For peer review only - http://bmjopen.bmj.com/site/about/guidelines.xhtml

Abstract

Introduction

Several licensed and investigational vaccines have lower efficacy, and induce impaired immune responses, in low-income versus high-income countries and in rural, versus urban, settings. Understanding these population differences is essential to optimising vaccine effectiveness in the tropics. We suggest that repeated exposure to and immunomodulation by chronic helminth infections partly explains population differences in vaccine response.

Methods and analysis

We have designed an individually randomised, parallel group trial of intensive versus standard praziquantel (PZQ) intervention against schistosomiasis, to determine effects on vaccine response outcomes among school-going adolescents (9 to 17 years) from rural *Schistosoma mansoni* (*Sm*)-endemic Ugandan islands. Vaccines to be studied comprise BCG on day ‘zero’; yellow fever, oral typhoid and Human Papilloma Virus (HPV) vaccines at week 4; and HPV and Tetanus/diphtheria booster vaccine at week 28. The intensive arm will receive PZQ doses three times, each two weeks apart, before BCG immunisation, followed by a dose at week 8 and quarterly thereafter. The standard arm will receive PZQ at week 8 and week 52. We expect to enrol 480 participants, with 80% *Sm* infected at the outset.

Primary outcomes are BCG-specific IFN-γ ELISpot responses eight weeks after BCG immunisation and for other vaccines, antibody responses to key vaccine antigens at four weeks after immunisation. Secondary analyses will determine effects of intensive anthelminthic treatment on correlates of protective immunity, on vaccine response waning, on priming vs boosting immunisations, and on *Sm* infection status and intensity. Exploratory immunology assays using archived samples will enable assessment of mechanistic links between helminths and vaccine responses.

Ethics and dissemination

Ethics approval has been obtained from relevant Ugandan and UK ethics committees. Results will be shared with Uganda Ministry of Health, relevant district councils, community leaders and study participants. Further dissemination will be done through conference proceedings and publications.

Trial registration

Current Controlled Trials identifier: ISRCTN60517191.

For peer review only

For peer review only - http://bmjopen.bmj.com/site/about/guidelines.xhtml

Article summary

Strengths and limitations of this study

- This will be the first adequately powered intervention study to investigate effects of schistosomiasis treatment on vaccine responses in adolescents.
- Effects on both live-attenuated and inert vaccines will be studied.
- Our strong immunoepidemiological design and nested immunological studies will address specific hypotheses regarding pathways of effects.
- The sample archives developed will provide a major asset for exploration of new leads arising from this hypothesis-driven work, or for an alternative, “systems biology” approach investigating (for example) transcriptome, microbiome and virome.
- Even with intensive anthelminthic intervention, it may be difficult to “successfully” treat *Schistosoma* infection in our endemic setting due to re-infections; however, we still expect a substantial difference in intensity between the two trial arms.

Word count

3347

Keywords

Vaccine; Schistosomiasis; Praziquantel; Immunization

For peer review only

For peer review only - http://bmjopen.bmj.com/site/about/guidelines.xhtml

Introduction

Vaccine-specific immune responses are often impaired, and vaccine efficacy and effectiveness lower, in tropical low-income countries (LICs) compared to temperate high-income countries and in rural, compared to urban, LIC settings.[1-8] This has been recognised for both live vaccines (such as BCG,[2 3 5 9] polio,[1] yellow fever[4] vaccines) and non-live vaccines (such as influenza[10] and tetanus[11]). Investigational malaria[7] and viral-vectored tuberculosis[6] and Ebola[12] vaccines are also affected. Previous exposure to the target pathogen (or related organisms) may mask the benefit of the vaccine.[13 14] However, pre-vaccination exposure does not explain why Ebola trial vaccine-specific responses differ between healthy UK and Senegalese adults,[12] as the target organism is rare. Therefore, environmentally-dependent mechanisms may play an important role.[5]

A long-held hypothesis is that parasites, particularly helminths, modulate vaccine responses through profound pre- and post-immunisation bystander effects on immunological activation and regulation.[15-17] Helminths might also impact vaccines responses through interactions with the complex ecosystem of mammalian gut bacteria, fungi, protozoa and viruses (the"trans-kingdom" concept[18] detailed elsewhere in this journal [bmjopen-2020-040425]). Helminth-induced gut mucosa damage, the associated translocation of microbial products into the systemic circulation[19-21] and systemic immune activation or regulation mediated by microbial products might contribute to modulation of responses to vaccines and other infections.

Helminth-mediated modulation of vaccine responses has not been substantiated in human populations. No appropriately powered trials have been conducted to evaluate reversibility of their effects. In animal models, helminths generally impair priming and accelerate waning of vaccine responses, although effects vary with helminth species, vaccine type and the timing of infection and immunisation.[22] Most observational studies in humans also suggest suppressed or biased responses during helminth infection, especially during systemic infections, such as schistosomiasis and the filariases. There is modest evidence that treating geohelminths in humans improves responses to BCG[23 24] or oral cholera vaccine[25] and we found that schistosomiasis treatment improved the measles-booster response in pre-school children.[26] There is therefore a strong case for a comprehensive assessment of the effects of helminths and their treatment on vaccine responses.

The extent to which helminths and related "trans-kingdom" mediators causally and reversibly impact immunological characteristics associated with vaccine responses may best be determined by intervention studies. This trial protocol A of the 'Population differences in Vaccine responses" programme (POPVAC A; Current Controlled Trials identifier: ISRCTN60517191) has been designed to evaluate the effect of *Schistosoma mansoni* and its treatment on vaccine responses. This study is

For peer review only - http://bmjopen.bmj.com/site/about/guidelines.xhtml

101 one of three parallel trials whose designs and cross-cutting analyses are described separately in this journal (bmjopen-2020-040425, bmjopen-2020-040427 and bmjopen-2020-040430).

For peer review only

For peer review only - http://bmjopen.bmj.com/site/about/guidelines.xhtml

Hypothesis

The overarching goal of the POPVAC programme is to understand population differences in vaccine responses in Uganda, in order to identify strategies through which vaccine effectiveness can be optimised for the low-income, tropical settings where they are especially needed. For this Trial A, we focus on the hypothesis that *Schistosoma mansoni infection suppresses responses to unrelated vaccines; and that this effect can be reversed, at least in part, by intensive praziquantel treatment intervention.*

Objective

To determine whether there are reversible effects of chronic *Schistosoma mansoni* infection on vaccine response in adolescents, using an intervention study.

For peer review only - http://bmjopen.bmj.com/site/about/guidelines.xhtml

Methods and analysis

Setting and participants

SPIRIT reporting guidelines[27] have been used. We will conduct an individually randomised, parallel group trial of intensive versus standard intervention against schistosomiasis (described below) in the *S. mansoni*-endemic Koome islands of Lake Victoria, Mukono district, Uganda.[28] We aim to enroll 480 participants, randomising 240 to each intervention arm. The study cohort will recruit participants aged 9 to 17 years in primary school years 1 to 6. Adolescents[29] in this study setting bear a heavy parasite burden.[30] In addition, this age-group is a target group for vaccines against sexually transmitted infections (currently human papilloma virus [HPV] – in future, it is hoped, for vaccines against HIV) and for booster immunisations.

Recruitment criteria

Inclusion criteria

i. Attending the selected school and planning to continue to attend the school for the duration of the study
ii. Aged 9 to 17 years and enrolled in primary 1 to 6 (to avoid primary leaving examinations in late year 7, and loss to follow up of children leaving after primary 7)
iii. Written informed assent by participant and consent by parent or guardian
iv. Females agree to avoid pregnancy for the duration of the trial
v. Willing to provide locator information and to be contacted during the course of the trial
vi. Able and willing (in the investigator's opinion) to comply with all the study requirements

Exclusion criteria

i. Clinically significant history of immunodeficiency (including HIV), cancer, cardiovascular disease, gastrointestinal disease, liver disease, renal disease, endocrine disorder and neurological illness
ii. History of serious psychiatric condition or disorder
iii. Moderate or severe acute illness characterised by any of the following symptoms: fever, impaired consciousness, convulsions, difficulty in breathing, vomiting; or as otherwise determined by the attending project clinician.
iv. Concurrent oral or systemic steroid medication or the concurrent use of other immunosuppressive agents within 2 months prior to enrolment
v. History of allergic reaction to immunisation or any allergy likely to be exacerbated by any component of the study vaccines including egg or chicken proteins

For peer review only - http://bmjopen.bmj.com/site/about/guidelines.xhtml

vi. History of previous immunisation with Yellow Fever (YF), oral typhoid or Human Papillomavirus (HPV) vaccine; previous immunisation with BCG or Tetanus and diphtheria vaccine (Td) at age $\geq$5 years

vii. Tendency to develop keloid scars

viii. Haemoglobin less than 82g/L

ix. Positive HIV serology

x. Positive pregnancy test

xi. Female currently lactating, confirmed pregnancy or intention to become pregnant during the trial period

xii. Use of an investigational medicinal product or non-registered drug, live vaccine, or medical device other than the study vaccines for 30 days prior to dosing with the study vaccine, or planned use during the study period

xiii. Administration of immunoglobulins and/or any blood products within the three months preceding the planned trial immunisation date

Further information on recruitment criteria can be found in Supplementary information.

Interventions

We will individually randomise participants to intensive or standard praziquantel (PZQ) treatment, in a 1:1 ratio. The intensive arm will receive three doses of PZQ (approximately 40mg/kg, assessed by height pole[31]) each two weeks apart prior to the first immunisation (the last of these 2-3 weeks before immunisation), followed by PZQ at 8 weeks (after primary endpoint sampling) and thereafter quarterly PZQ (approximately; timings adjusted to accommodate school terms) during follow up. The standard arm will receive their first dose of PZQ at week 8 (after immunisation and after primary endpoint sampling) and a second dose at week 52 (to conform to Uganda Ministry of Health policy, which is annual treatment) (**Figure 1**). No placebo will be used in this trial because all participants will be treated (albeit at different frequencies) and participants are unlikely to seek additional treatments outside the trial schedule: praziquantel treatment is not popular because of the recognised (although temporary) adverse effects (described in Supplementary information).

Randomisation and allocation to treatment arm

A randomisation code will be generated by an independent statistician using a randomly permuted block size (sizes 4, 6, 8 and 10) and used to allocate participants to either receive quarterly PZQ (intensive arm) or annual PZQ (standard arm). A set of envelopes will be prepared, labelled sequentially with the randomisation numbers and containing a card indicating the corresponding allocation (to intensive or standard treatment). The randomisation code will be kept securely by the

For peer review only

trial statistician and made available only to those responsible for providing or preparing the trial interventions. A second copy will be held by a data manager or statistician not otherwise involved in the trial at the MRC/UVRI and LSHTM Uganda Research Unit. At enrolment, eligibility criteria will be checked and eligible participants will be allocated sequentially to the next randomisation number until the required sample size is achieved. Randomisation implementation will be done by a clinician using the sequentially numbered opaque sealed envelopes. When the next randomisation number in the sequence is allocated, the envelope bearing that number will be opened to reveal the allocation.

Blinding

Clinicians and participants will not be blinded to the treatment allocation since they will not participate in outcome ascertainment; only immunology laboratory staff who are assessing trial outcomes will be blinded.

Immunisations

We will study a portfolio of licensed vaccines (live and inert, oral and parental, priming and boosting) expected to be beneficial (in some cases, already given) to adolescents in Uganda. Our schedule (**Table 1,** supplementary **Table S1**) will comprise three main immunisation days (week 0, week 4 and week 28). Additional HPV immunisation will be provided for girls aged 14 years or above, and a second Tetanus/diphtheria boost will be given after completion of the study, to accord with the national Expanded Programme on Immunisation (EPI) routines, but the response to these will not specifically be assessed. Further rationale for the selection of vaccines is detailed in the Supplementary information.

Table 1. Immunisation schedule

	Immunisation week 0	Immunisation week 4	[Immunisation week 8]	Immunisation week 28	[Immunisation week 52]
**Live vaccines**	BCG vaccination / re-vaccination[1]	Yellow fever (YF-17D)<br>Oral typhoid (Ty21a)			
**Non-live vaccines**		HPV prime[2]	HPV boost for girls aged ≥14 years[3,4]	HPV boost[2] and Tetanus/ diphtheria (Td) boost	Tetanus/ diphtheria (Td) boost[4,5]

1. Prior BCG status may vary (data on history and documentation of prior BCG, and presence of a BCG scar, will be documented although these approaches have limitations for determining BCG status)
2. Both girls and boys will receive the HPV vaccine
3. The National EPI programme recommends three doses of HPV vaccine for older girls
4. These doses will be given to comply with guidelines but outcomes specifically relating to these doses will not be assessed
5. Priming by immunisation in infancy is assumed

For peer review only

For peer review only - http://bmjopen.bmj.com/site/about/guidelines.xhtml

Schedule of immunisation and sampling

The schedule of immunisation and sampling is outlined in **Figure 1** and **Table S1**. Pre-immunisation vaccine responses will be assessed in baseline samples. While optimal timings for outcome measures vary between vaccines, sampling at 8 weeks post BCG and 4 weeks post YF-17D, Ty21a, HPV and Td is proposed for the primary endpoints, targeting the establishment of memory responses and approximate peak of antibody responses. A secondary endpoint at one year will assess waning. Immunisation postponement criteria are detailed in Supplementary information.

Outcomes

Primary outcomes

These will be assessed in all participants.

i. **BCG:** BCG-specific IFN-γ ELISpot response eight weeks post BCG immunisation.
ii. **YF-17D:** neutralising antibody titres (plaque-reduction neutralisation test) at four weeks post YF immunisation.
iii. **Ty21a:** *Salmonella typhi* lipopolysaccharide (LPS)-specific immunoglobulin (Ig)G concentration at four weeks post Ty21a immunisation.
iv. **HPV:** IgG specific for L1-proteins of HPV-16/18 at four weeks post HPV priming immunisation.
v. **Td:** Tetanus and diphtheria toxoid-specific IgG concentration at four weeks post Td immunisation.

Secondary outcomes

These will be assessed in all participants and will further investigate estimates of protective immunity (for vaccines where these are available) and dynamics of the vaccine responses, as well as the impact of the interventions on parasite clearance.

i. **Protective immunity.** Proportions with protective neutralising antibody (YF); protective IgG levels (TT);[32] seroconversion rates (Ty21a) at four weeks post the corresponding immunisation.
ii. **Response waning.** Primary outcome measures (all vaccines) repeated at week 52, and area-under-the curve (AUC) analyses. Parasitic infection may accelerate,[33] and anti-parasitic interventions delay, waning.
iii. **Priming versus boosting.** Effects on priming versus boosting will be examined for HPV only, comparing outcomes four weeks after the first, and four weeks after the second vaccine dose.

For peer review only - http://bmjopen.bmj.com/site/about/guidelines.xhtml

For peer review only

iv. **Current *S. mansoni* infection status and intensity** will be determined by serum/plasma levels of circulating anodic antigen (CAA). The method is quantitative, highly specific for *Schistosoma* infection, and much more sensitive than the conventional Kato Katz method.[34] CAA will be assessed retrospectively on stored samples collected at baseline, on immunisation days, and on primary and secondary endpoint days.

Furthermore, our sample collection will offer opportunities for an array of exploratory immunological evaluations on stored samples, focusing mainly on vaccine antigen specific outcomes. Exploratory assays will provide further detail on the role of immunological profiles and trans-kingdom effects in mediating helminth modulation of vaccine-specific responses.

Evaluation of parasite infection exposure

The following measures will also be assessed in all participants, and will be used to describe the general infection-exposure experience of the study participants.

i. **Prior exposure to schistosomiasis** will be evaluated by ELISA for IgG to schistosome egg antigen using stored blood samples collected at baseline.
ii. **The presence of other helminth infections** will be determined retrospectively using stool PCR of samples collected at baseline and at weeks 28 and 52.[30] In accordance with national guidelines, all participants will be treated with albendazole or mebendazole after collection of samples for primary endpoints at week 8 and 28, and after collection of samples for secondary endpoints at week 52.
iii. **Current malaria infection status and intensity** will be assessed retrospectively by PCR on stored samples collected on immunisation days and at week 52. Individuals presenting with fever will be investigated using rapid diagnostic tests for malaria and treated based on the results and according to prevailing national guidelines.
iv. **Prior malaria exposure** will be evaluated by ELISA for IgG to malaria antigen using stored samples collected at baseline.

Sample size considerations

Based on the literature[4 35 36] and preliminary data, we anticipate that, following log to base 10 transformations that will be applied to normalise primary outcome measures, standard deviations (SDs) of primary outcome measures will lie between 0.3 and 0.6 on this log scale, and that effective treatment may increase responses by approximately 0.2 on the log scale (based on Tweyongyere *et al.*[26]). We have therefore powered our study to detect differences of this magnitude (0.2 on the log scale) or (in some cases) smaller (**Table 2**). We assume *S. mansoni* prevalence of $\geq$80%.

For peer review only - http://bmjopen.bmj.com/site/about/guidelines.xhtml

Based on these assumptions and a two independent samples t-test, we plan to include 480 participants in total (240 quarterly PZQ, 240 annual PZQ); of whom 384 are expected to be *S. mansoni* infected,[28] giving 192 participants in each trial arm who are infected at baseline.

Table 2 shows power estimates, for 5% type-1 error rate for each primary outcome measure and assuming 20% loss to follow-up.

Table 2. Power estimates (5% type-1 error rate for each primary outcome measure)

Standard deviation (log_{10})	Difference in mean log_{10} transformed outcome, between trial arms						
	**0.08**	**0.10**	**0.12**	**0.14**	**0.16**	**0.18**	**0.20**
192 intensive PZQ vs 192 standard PZQ (*S. mansoni* infected only)							
0.3	65%	83%	94%	98%	>99%	>99%	>99%
0.4	42%	59%	75%	87%	94%	98%	99%
0.5	29%	42%	56%	69%	80%	88%	94%
0.6	21%	31%	42%	53%	65%	75%	83%

Cells highlighted in grey correspond to >80% power; differences in mean log10 transformed outcome of 0.08, 0.10, 0.12, 0.14, 0.16, 0.18 and 0.20 are equivalent to geometric mean ratios for untransformed outcomes of 1.20, 1.26, 1.32, 1.38, 1.45, 1.51 and 1.59, respectively.

Ethics and dissemination

Ethical approval has been granted from the Research Ethics Committee of the Uganda Virus Research Institute (UVRI REC, reference: GC/127/19/05/664) and the London School of Hygiene and Tropical Medicine (LSHTM, reference: 16032), and from the Uganda National Council for Science and Technology (UNCST, reference: HS2486) and the Uganda National Drug Authority (NDA, reference: CTA0093). Any protocol amendments will be submitted to ethics committees and regulatory bodies for approval before implementation.

Participants are adolescents and therefore a vulnerable human population. Care will be taken to provide adequate, age and education-status appropriate information and to ensure that it is understood; and to emphasise that participation is voluntary. Participants will be enrolled only when they have given their own assent and when consent has been given by the parent or guardian. Model consent and assent forms are shown in Supplementary file 2. No major risks to the participants are anticipated since all the treatments and vaccines to be given are licensed and known to be safe. The main risk to participants will be time lost from school work: we will work with teachers and parents to minimise disruption to classes, and will avoid enrolment of primary 7 students since these classes are involved in national examinations. Further risks are discussed in Supplementary information.

For peer review only

For peer review only - http://bmjopen.bmj.com/site/about/guidelines.xhtml

Study findings will be published through open access peer-reviewed journals, presentations at local, national and international conferences and to the local community through community meetings. Anonymised participant level datasets generated will be available upon request.

Patient and public involvement

Concepts involved in this work have been discussed with colleagues at the Vector Control Division and Expanded Programme on Immunisation in the Ministry of Health (Uganda), with the Mukono District Council and with community leaders and Village Health Teams from Koome subcounty. We also have held meetings to explain the proposed work to teachers, parents, participants and village members, and to address their questions about issues such as study length, the study's ethical approval status, why adults were excluded from the study, and to explain to them why boys will also receive the HPV vaccine. Study findings will be shared with these stakeholders and with participants.

Data management and analysis

Socio-demographic information and clinical and laboratory measurements will be recorded and managed using Research Electronic Data Capture (REDCap) tools,[37 38] with paper-based forms as back-up. All data will be recorded under a unique study ID number. When paper forms must be used, data will be double entered in a study-specific database, with standard checks for discrepancies. All data for analysis will be anonymised and stored on a secure and password-protected server, with access limited to essential research personnel.

Baseline characteristics including age, sex, school, location of birth, prior vaccination status, helminth infection and prior exposure status and malaria infection and prior exposure status will be summarised by trial arm. The effect of intensive (compared to standard) praziquantel treatment on the outcomes will be analysed. Information on infection status will only be available after randomisation. The primary analysis will be done on individuals identified as infected at baseline (through randomisation, these will be balanced between treatment arms); this will test the hypothesis that treating the infection (and subsequent reinfections) reverses the parasite's effects on vaccine responses. If treating *S. mansoni* reverses adverse parasite effects on vaccine responses, this may be a beneficial public health intervention. However, routine screening for parasite infection before immunisation would be laborious. Secondary analyses will include all randomised individuals; this will provide insight into the broader benefit of the interventions as public health measures. The effect of intensive versus standard praziquantel treatment on primary outcomes will be assessed using unpaired t-tests, with results presented as a mean difference in vaccine response measure together with 95% confidence interval and p-value. For all outcomes, we will investigate adjusting for corresponding baseline vaccine responses as this may improve the precision of effect estimates;

For peer review only - http://bmjopen.bmj.com/site/about/guidelines.xhtml

this will be done using multivariable regression. We anticipate that outcomes will be positively skewed, and will apply log transformations to normalise distributions before analysis if required. The detailed analytical plan is available on the online trial registration site (http://www.isrctn.com/ISRCTN60517191).

For peer review only

For peer review only - http://bmjopen.bmj.com/site/about/guidelines.xhtml

Discussion

This will be the first adequately powered intervention study to investigate the effect of schistosomiasis treatment on vaccine responses in adolescents. This study will determine whether *S. mansoni* infection has a causal, reversible, impact on the response to live or inert vaccines, including effects on vaccine replication, immune response profile, priming, boosting and waning. The results will add to understanding of population differences in vaccine responses and on interventions that may enhance responses. If treating helminths improves vaccine responses in adolescents, combined parasite-control/immunisation programmes offer an attractive, practical public health intervention for schools and communities.

There are risks associated with our approach to addressing the trial objective. **First**, there is a risk of failure to clear *S. mansoni* infections, and repeated reinfection during the trial. This issue can be challenging because of incomplete cure or maturation of immature worms after treatment, and lifestyles in endemic communities that result in repeated exposure. To mitigate this, we will administer three PZQ treatments over a six-week period before the first immunisations, and continuing quarterly treatment in the intensive arm. **Second,** there is a risk that *S. mansoni* infection has long-term effects, not removed by treatment, mediated, for example, by epigenetic change.[39] However, studies show that parasite treatment results in immunological changes,[40 41] and our data suggest at least partial recovery of the measles vaccine response among young children treated for schistosomiasis.[26] By initiating intervention six to eight weeks before the first immunisations, and providing repeated intervention in the intensive arms, we hope to achieve significant resolution of *S. mansoni* effects.

We are interested in the effects of removing *S. mansoni*. Treating parasites can induce acute immunological change due to release of previously hidden antigens.[42 43] To minimise such effects, immunisations will be given at least 2 weeks after PZQ (the longest practicable interval; **Figure 1**).

Laboratory analyses will also highlight immune parameters and cellular populations that link environmental exposures to vaccine responses. Identifying processes associated with poor or good outcomes will inform strategies in vaccine design (for example, the genetic modification of vaccines, or innovative use of adjuvants, to counter any adverse immunological milieu, currently an area of intense research for cancer vaccines[44]); ultimately supporting the development of effective vaccines tailored to the low-income settings that most need them.

Study timeline

For peer review only

For peer review only - http://bmjopen.bmj.com/site/about/guidelines.xhtml

355 Applications for ethical approval were submitted in May 2018, with approval received in September
356 2018 (Uganda Virus Research Institute Research Ethics Committee), May 2019 (National Drug
357 Authority and Uganda National Council for Science and Technology), June 2019 (London School of
358 Hygiene and Tropical Medicine). Collaborator/investigator/trial steering committee meetings were
359 also held during the initial 12-month planning period. The study began recruitment in July 2019.
360 Intervention will be up to 12 months, with completion of the project scheduled for September 2020.

361

362 **Competing interests**

363 Alison Elliott reports a grant from the Medical research Council, UK (POPVAC programme funding).
364 The rest of the authors declare that they have no conflicts of interest.

365 **Author contributions**

366 AME conceived the study. AME, GN, ELW, AN, JN, SC, LZ and JK contributed to study design. LZ, GO,
367 PNK, EN, GK, RA, CN, CO, MN, CZ, SA and FA are site clinicians/nurses/clinical laboratory technicians
368 providing valuable input on clinical considerations of the intervention. MS, SK and RK are field
369 workers handling the organisational integration of the intervention. AN, AM and ELW are involved in
370 organisation of the databases, trial randomization, treatment allocation and drawing up of analytical
371 plans. GN, LZ, JN, AN, SC, ELW and AME drafted the manuscript. All authors reviewed the
372 manuscript, contributed to it and approved the final version.

373 **Acknowledgements**

374 We thank the Uganda National Expanded Programme for Immunisation, Sanofi Pasteur and PaxVax
375 for providing the HPV, yellow fever and oral typhoid vaccines, respectively. The BCG and tetanus-
376 diphtheria vaccines were kind donations from the Serum Institute of India. We thank the Vector
377 Control Division of the Ministry of Health and the Mukono district local government for their
378 support. We also thank members of the POPVAC programme steering committee (chaired by Prof.
379 Richard Hayes) and the Data and Safety Monitoring Board (Dr David Meya, Prof Andrew Prendergast
380 and Dr Elizabeth George).

381 **Funding**

382 The POPVAC programme of work is supported by the Medical Research Council of the United
383 Kingdom (grant number MR/R02118X/1). SC and JN are supported in part by the Makerere
384 University – Uganda Virus Research Institute Centre of Excellence for Infection and Immunity
385 Research and Training (MUII-plus). MUII-plus is funded under the DELTAS Africa Initiative. The
386 DELTAS Africa Initiative is an independent funding scheme of the African Academy of Sciences (AAS),

For peer review only

Alliance for Accelerating Excellence in Science in Africa (AESA) and supported by the New Partnership for Africa's Development Planning and Coordinating Agency (NEPAD Agency) with funding from the Wellcome Trust (grant 107743) and the UK Government. The MRC/UVRI and LSHTM Uganda Research Unit is jointly funded by the UK Medical Research Council (MRC) and the UK Department for International Development (DFID) under the MRC/DFID Concordat agreement and is also part of the EDCTP2 programme supported by the European Union.

The study sponsor (London School of Hygiene and Tropical Medicine) and funders had no role in study design; collection, management, analysis, and interpretation of data; writing of the protocol; and the decision to submit the protocol for publication.

POPVAC trial team

Principal investigator**:* Alison Elliott; ***Project leader: Ludoviko Zirimenya; ***laboratory staff**:* Gyaviira Nkurunungi, Stephen Cose, Rebecca Amongin, Beatrice Nassanga, Jacent Nassuuna, Irene Nambuya, Prossy Kabuubi, Emmanuel Niwagaba, Gloria Oduru, Grace Kabami; ***statisticians and data managers**:* Emily Webb, Agnes Natukunda, Helen Akurut, Alex Mutebe; ***clinicians**:* Anne Wajja, Milly Namutebi, Christopher Zziwa, Joel Serubanja; ***nurses**:* Caroline Onen, Esther Nakazibwe, Josephine Tumusiime, Caroline Ninsiima, Susan Amongi, Florence Akello; ***internal monitor**:* Mirriam Akello; ***field workers**:* Robert Kizindo, Moses Sewankambo, Denis Nsubuga, Samuel Kiwanuka, Fred Kiwudhu; ***boatman**:* David Abiriga; ***administrative management**:* Moses Kizza, Samsi Nansukusa; ***internal and external collaborators:*** Pontiano Kaleebu, Hermelijn Smits, Maria Yazdanbakhsh, Govert van Dam, Paul Corstjens, Sarah Staedke, Henry Luzze, James Kaweesa, Edridah Tukahebwa, Elly Tumushabe, Moses Muwanga

For peer review only

For peer review only - http://bmjopen.bmj.com/site/about/guidelines.xhtml

References

1. Taniuchi M, Platts-Mills JA, Begum S, et al. Impact of enterovirus and other enteric pathogens on oral polio and rotavirus vaccine performance in Bangladeshi infants. *Vaccine* 2016;34(27):3068-75. doi: 10.1016/j.vaccine.2016.04.080 [published Online First: 2016/05/08]

2. Fine PE. Variation in protection by BCG: implications of and for heterologous immunity. *Lancet* 1995;346(8986):1339-45.

3. Black GF, Weir RE, Floyd S, et al. BCG-induced increase in interferon-gamma response to mycobacterial antigens and efficacy of BCG vaccination in Malawi and the UK: two randomised controlled studies. *Lancet* 2002;359(9315):1393-401. doi: 10.1016/S0140-6736(02)08353-8

4. Muyanja E, Ssemaganda A, Ngauv P, et al. Immune activation alters cellular and humoral responses to yellow fever 17D vaccine. *The Journal of clinical investigation* 2014;124(7):3147-58. doi: 10.1172/jci75429 [published Online First: 2014/06/10]

5. Barreto ML, Pilger D, Pereira SM, et al. Causes of variation in BCG vaccine efficacy: examining evidence from the BCG REVAC cluster randomized trial to explore the masking and the blocking hypotheses. *Vaccine* 2014;32(30):3759-64. doi: 10.1016/j.vaccine.2014.05.042

6. Tanner R, Kakalacheva K, Miller E, et al. Serum indoleamine 2,3-dioxygenase activity is associated with reduced immunogenicity following vaccination with MVA85A. *BMC infectious diseases* 2014;14:660. doi: 10.1186/s12879-014-0660-7 [published Online First: 2014/12/04]

7. Sissoko MS, Healy SA, Katile A, et al. Safety and efficacy of PfSPZ Vaccine against Plasmodium falciparum via direct venous inoculation in healthy malaria-exposed adults in Mali: a randomised, double-blind phase 1 trial. *The Lancet Infectious diseases* 2017;17(5):498-509. doi: 10.1016/s1473-3099(17)30104-4 [published Online First: 2017/02/22]

8. Venkatraman N, Ndiaye BP, Bowyer G, et al. Safety and Immunogenicity of a Heterologous Prime-Boost Ebola Virus Vaccine Regimen in Healthy Adults in the United Kingdom and Senegal. *The Journal of infectious diseases* 2018;219(8):1187-97. doi: 10.1093/infdis/jiy639

9. Barreto ML, Pereira SM, Pilger D, et al. Evidence of an effect of BCG revaccination on incidence of tuberculosis in school-aged children in Brazil: second report of the BCG-REVAC cluster-randomised trial. *Vaccine* 2011;29(31):4875-7. doi: 10.1016/j.vaccine.2011.05.023

10. van Riet E, Adegnika AA, Retra K, et al. Cellular and humoral responses to influenza in gabonese children living in rural and semi-urban areas. *The Journal of infectious diseases* 2007;196(11):1671-8. doi: 10.1086/522010 [published Online First: 2007/11/17]

11. van Riet E, Retra K, Adegnika AA, et al. Cellular and humoral responses to tetanus vaccination in Gabonese children. *Vaccine* 2008;26(29-30):3690-5. doi: 10.1016/j.vaccine.2008.04.067 [published Online First: 2008/06/10]

12. Venkatraman N, Ndiaye BP, Bowyer G, et al. Safety and Immunogenicity of a Heterologous Prime-Boost Ebola Virus Vaccine Regimen in Healthy Adults in the United Kingdom and Senegal. *The Journal of infectious diseases* 2019;219(8):1187-97. doi: 10.1093/infdis/jiy639

13. Brandt L, Feino Cunha J, Weinreich Olsen A, et al. Failure of the Mycobacterium bovis BCG vaccine: some species of environmental mycobacteria block multiplication of BCG and induction of protective immunity to tuberculosis. *Infection and immunity* 2002;70(2):672-8. [published Online First: 2002/01/18]

14. Flaherty DK, Vesosky B, Beamer GL, et al. Exposure to Mycobacterium avium can modulate established immunity against Mycobacterium tuberculosis infection generated by Mycobacterium bovis BCG vaccination.

For peer review only - http://bmjopen.bmj.com/site/about/guidelines.xhtml

Journal of leukocyte biology 2006;80(6):1262-71. doi: 10.1189/jlb.0606407 [published Online First: 2006/09/14]

15. Maizels RM, McSorley HJ. Regulation of the host immune system by helminth parasites. *The Journal of allergy and clinical immunology* 2016;138(3):666-75. doi: 10.1016/j.jaci.2016.07.007 [published Online First: 2016/08/02]

16. Wammes LJ, Mpairwe H, Elliott AM, et al. Helminth therapy or elimination: epidemiological, immunological, and clinical considerations. *The Lancet Infectious diseases* 2014;14(11):1150-62. doi: 10.1016/S1473-3099(14)70771-6

17. Wammes LJ, Hamid F, Wiria AE, et al. Regulatory T cells in human geohelminth infection suppress immune responses to BCG and Plasmodium falciparum. *Eur J Immunol* 2010;40(2):437-42. doi: 10.1002/eji.200939699

18. Pfeiffer JK, Virgin HW. Viral immunity. Transkingdom control of viral infection and immunity in the mammalian intestine. *Science* 2016;351(6270) doi: 10.1126/science.aad5872

19. Onguru D, Liang Y, Griffith Q, et al. Human schistosomiasis is associated with endotoxemia and Toll-like receptor 2- and 4-bearing B cells. *The American journal of tropical medicine and hygiene* 2011;84(2):321-4. doi: 10.4269/ajtmh.2011.10-0397 [published Online First: 2011/02/05]

20. George PJ, Anuradha R, Kumar NP, et al. Evidence of microbial translocation associated with perturbations in T cell and antigen-presenting cell homeostasis in hookworm infections. *PLoS neglected tropical diseases* 2012;6(10):e1830. doi: 10.1371/journal.pntd.0001830 [published Online First: 2012/10/12]

21. Rajamanickam A, Munisankar S, Bhootra Y, et al. Microbial Translocation Associated with an Acute-Phase Response and Elevations in MMP-1, HO-1, and Proinflammatory Cytokines in Strongyloides stercoralis Infection. *Infection and immunity* 2017;85(1) doi: 10.1128/iai.00772-16 [published Online First: 2016/11/09]

22. Sanya RE, Nkurunungi G, Andia Biraro I, et al. A life without worms. *Transactions of the Royal Society of Tropical Medicine and Hygiene* 2017:1-9. doi: 10.1093/trstmh/trx010 [published Online First: 2017/03/25]

23. Elias D, Britton S, Aseffa A, et al. Poor immunogenicity of BCG in helminth infected population is associated with increased in vitro TGF-beta production. *Vaccine* 2008;26(31):3897-902. doi: S0264-410X(08)00540-9 [pii]
10.1016/j.vaccine.2008.04.083 [published Online First: 2008/06/17]

24. Elias D, Wolday D, Akuffo H, et al. Effect of deworming on human T cell responses to mycobacterial antigens in helminth-exposed individuals before and after bacille Calmette-Guerin (BCG) vaccination. *Clin Exp Immunol* 2001;123(2):219-25.

25. Cooper PJ, Chico ME, Losonsky G, et al. Albendazole treatment of children with ascariasis enhances the vibriocidal antibody response to the live attenuated oral cholera vaccine CVD 103-HgR. *The Journal of infectious diseases* 2000;182(4):1199-206. doi: 10.1086/315837 [published Online First: 2000/09/09]

26. Tweyongyere R, Nassanga BR, Muhwezi A, et al. Effect of Schistosoma mansoni infection and its treatment on antibody responses to measles catch-up immunisation in pre-school children: A randomised trial. *PLoS neglected tropical diseases* 2019;13(2):e0007157. doi: 10.1371/journal.pntd.0007157 [published Online First: 2019/02/15]

27. Chan AW, Tetzlaff JM, Altman DG, et al. SPIRIT 2013 statement: defining standard protocol items for clinical trials. *Ann Intern Med* 2013;158(3):200-7. doi: 10.7326/0003-4819-158-3-201302050-00583 [published Online First: 2013/01/09]

28. Sanya RE, Nkurunungi G, Hoek Spaans R, et al. The Impact of Intensive Versus Standard Anthelminthic Treatment on Allergy-related Outcomes, Helminth Infection Intensity, and Helminth-related Morbidity in Lake Victoria Fishing Communities, Uganda: Results From the LaVIISWA Cluster-randomized Trial. *Clinical*

For peer review only - http://bmjopen.bmj.com/site/about/guidelines.xhtml

infectious diseases : an official publication of the Infectious Diseases Society of America 2019;68(10):1665-74. doi: 10.1093/cid/ciy761 [published Online First: 2018/09/12]

29. WHO. Adolescent Health Research Priorities: Report of a Technical Consultation 2015. http://apps.who.int/iris/bitstream/10665/203564/1/WHO_FWC_MCA_15_07_eng.pdf?ua=1 (accessed 17th May 2019).

30. Webb EL, Nampijja M, Kaweesa J, et al. Helminths are positively associated with atopy and wheeze in Ugandan fishing communities: results from a cross-sectional survey. *Allergy* 2016 doi: 10.1111/all.12867

31. Montresor A, Odermatt P, Muth S, et al. The WHO dose pole for the administration of praziquantel is also accurate in non-African populations. *Transactions of the Royal Society of Tropical Medicine and Hygiene* 2005;99(1):78-81. doi: 10.1016/j.trstmh.2004.06.006

32. Plotkin SA. Correlates of protection induced by vaccination. *Clinical and vaccine immunology : CVI* 2010;17(7):1055-65. doi: 10.1128/cvi.00131-10 [published Online First: 2010/05/14]

33. Riner DK, Ndombi EM, Carter JM, et al. Schistosoma mansoni Infection Can Jeopardize the Duration of Protective Levels of Antibody Responses to Immunizations against Hepatitis B and Tetanus Toxoid. *PLoS neglected tropical diseases* 2016;10(12):e0005180. doi: 10.1371/journal.pntd.0005180

34. Corstjens PL, Nyakundi RK, de Dood CJ, et al. Improved sensitivity of the urine CAA lateral-flow assay for diagnosing active Schistosoma infections by using larger sample volumes. *Parasites & vectors* 2015;8:241. doi: 10.1186/s13071-015-0857-7 [published Online First: 2015/04/22]

35. Fletcher HA, Snowden MA, Landry B, et al. T-cell activation is an immune correlate of risk in BCG vaccinated infants. *Nat Commun* 2016;7:11290. doi: 10.1038/ncomms11290

36. Safaeian M, Porras C, Pan Y, et al. Durable antibody responses following one dose of the bivalent human papillomavirus L1 virus-like particle vaccine in the Costa Rica Vaccine Trial. *Cancer prevention research (Philadelphia, Pa)* 2013;6(11):1242-50. doi: 10.1158/1940-6207.capr-13-0203 [published Online First: 2013/11/06]

37. Harris PA, Taylor R, Minor BL, et al. The REDCap Consortium: Building an International Community of Software Platform Partners. *Journal of biomedical informatics* 2019:103208. doi: 10.1016/j.jbi.2019.103208 [published Online First: 2019/05/13]

38. Harris PA, Taylor R, Thielke R, et al. Research electronic data capture (REDCap)--a metadata-driven methodology and workflow process for providing translational research informatics support. *Journal of biomedical informatics* 2009;42(2):377-81. doi: 10.1016/j.jbi.2008.08.010 [published Online First: 2008/10/22]

39. Blok BA, Arts RJ, van Crevel R, et al. Trained innate immunity as underlying mechanism for the long-term, nonspecific effects of vaccines. *J Leukoc Biol* 2015;98(3):347-56. doi: 10.1189/jlb.5RI0315-096R

40. Watanabe K, Mwinzi PN, Black CL, et al. T regulatory cell levels decrease in people infected with Schistosoma mansoni on effective treatment. *The American journal of tropical medicine and hygiene* 2007;77(4):676-82. [published Online First: 2007/11/06]

41. Schmiedel Y, Mombo-Ngoma G, Labuda LA, et al. CD4+CD25hiFOXP3+ Regulatory T Cells and Cytokine Responses in Human Schistosomiasis before and after Treatment with Praziquantel. *PLoS neglected tropical diseases* 2015;9(8):e0003995. doi: 10.1371/journal.pntd.0003995 [published Online First: 2015/08/21]

42. van den Biggelaar AHJ, Borrmann S, Kremsner P, et al. Immune Responses Induced by Repeated Treatment Do Not Result in Protective Immunity to Schistosoma haematobium: Interleukin (IL)–5 and IL-10 Responses. *The Journal of infectious diseases* 2002;186(10):1474-82. doi: 10.1086/344352

For peer review only - http://bmjopen.bmj.com/site/about/guidelines.xhtml

535 43. Woolhouse MEJ, Hagan P. Seeking the ghost of worms past. *Nature Medicine* 1999;5(11):1225-27. doi:
536 10.1038/15169

537 44. Seledtsov VI, Goncharov AG, Seledtsova GV. Clinically feasible approaches to potentiating cancer cell-based
538 immunotherapies. *Human vaccines & immunotherapeutics* 2015;11(4):851-69. doi:
539 10.1080/21645515.2015.1009814 [published Online First: 2015/05/02]

540

541

542 **FIGURE LEGENDS**

543 **Figure 1. Outline of immunisations and anthelminthic intervention**

544 [1]Primary endpoints will be at eight weeks post BCG and four weeks post Yellow fever (YF-17D), oral
545 typhoid (Ty21a), Human Papilloma Virus (HPV) and Tetanus/diptheria (Td) vaccination.

546 [2]Primary endpoint for responses to Td given at 28 weeks.

547

For peer review only

For peer review only - http://bmjopen.bmj.com/site/about/guidelines.xhtml

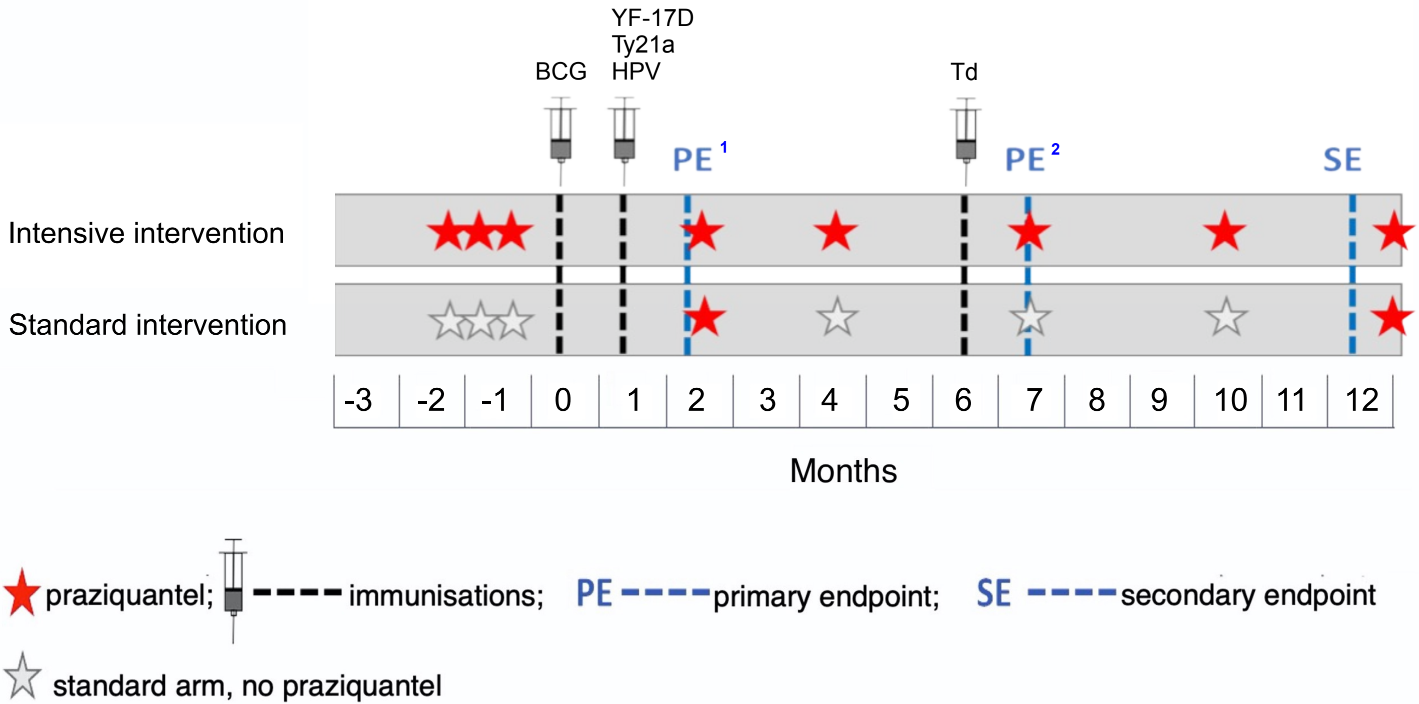
YF-17D
Ty21a
HPV
BCG
Td
PE 1
PE 2
SE
Intensive intervention
Standard intervention
-3
-2
-1
0
1
2
3
4
5
6
7
8
9
10
11
12
Months
praziquantel;
immunisations;
PE
primary endpoint;
SE
secondary endpoint
standard arm, no praziquantel

SUPPLEMENTARY INFORMATION

The effect of intensive treatment for schistosomiasis on immune responses to vaccines among rural Ugandan island adolescents: randomised controlled trial protocol A for the 'POPulation differences in VACcine responses' (POPVAC) programme

Gyaviira Nkurunungi[1,¶,*], Ludoviko Zirimenya[1,¶], Jacent Nassuuna[1,¶], Agnes Natukunda[1,¶], Prossy N Kabuubi[1], Emmanuel Niwagaba[1], Gloria Oduru[1], Grace Kabami[1], Rebecca Amongin[1], Alex Mutebe [1], Milly Namutebi[1], Christopher Zziwa[1], Susan Amongi[1], Caroline Ninsiima[1], Caroline Onen[1], Florence Akello[1], Moses Sewankambo[1], Samuel Kiwanuka[1], Robert Kizindo[1], James Kaweesa[2], Stephen Cose[1,3], Emily L Webb[4], Alison M Elliott[1,3] **for the POPVAC trial team**

[1]Immunomodulation and Vaccines Programme, Medical Research Council/Uganda Virus Research Institute and London School of Hygiene and Tropical Medicine (MRC/UVRI and LSHTM) Uganda Research Unit, Entebbe, Uganda

[2]Vector Control Division, Ministry of Health, Kampala, Uganda

[3]Department of Clinical Research, London School of Hygiene and Tropical Medicine, London, United Kingdom

[4]MRC Tropical Epidemiology Group, Department of Infectious Disease Epidemiology, London School of Hygiene and Tropical Medicine, London, United Kingdom

[¶]These authors contributed equally

***Correspondence:** Gyaviira Nkurunungi; Gyaviira.Nkurunungi@mrcuganda.org

For peer review only

Table S1. Schedule of visits and procedures

**VISIT NUMBER**	**1**	**2 & 3**	**4**	**5**[9]	**5.2**	**6**	**7**	**8**	**9**	**10**	**11**
**WEEKS FROM 1ST IMMUNISATION**	**-8**[1]	**-6**[10]**, -4, -2**	**0**	**4**	**4 weeks +4 days**	**8**	**20**	**28**	**32**	**44**	**52**
	**Screening**	**Treatment (Rx) only**	**Immunisations**	**Immunisations**		**Primary Endpoint (PE), Immunisations**	**Rx only**	**Immunisations**	**PE/Secondary Endpoint (SE)**[2]	**Rx only**	**SE, Immunisations**
**RANDOMISED INTENSIVE VS STANDARD PRAZIQUANTEL INTERVENTION**											
PZQ intensive arm (x)		x				x[3]	x		x[3]	x	x[3]
PZQ standard arm						x[3]					x[3]
Albendazole						x[3]			x[3]		x[3]
**VACCINES**											
BCG			x								
YF-17D				x							
Ty21a				x							
HPV				x		[x][4]		x			
Td								x			[x][5]
**INVESTIGATIONS/PROCEDURES**											
Inclusion/exclusion criteria	x										
Informed consent	x										
Questionnaire	x		x	x	x	x		x	x		x
Examination	x		(x)	(x)	(x)	(x)		(x)	(x)		(x)
Urine β-HCG test (female only) 1mL	x		x	x				x			
Urine YF viral load					x						
Stool for PCR and storage	x							x			x
Stool for coproantibody and storage	x					x					
**BLOOD SAMPLES**											
Malaria PCR (1ml)			x	x				x			x
Serology for HIV, prior malaria and *S. mansoni* (0.5ml)	x										
Mansonella perstans (1ml)	x										
Serum/plasma CAA (1ml)	x		x	x		x		x	x		x
Hb[8] / Full blood count (0.5ml)	x		x	x				x			
Assessments of pre-immunisation responses, and/or vaccine response outcomes and/or exploratory immunology; storage[7] (10 – 20 mls)			x	x		x		x	x		x
Blood for gene expression (2mls)			x	x				x			
Blood vol (mL)	4		27	17		10-20		27	10		14
Cumulative blood vol (mL)[7]	4		31	48		68		95	105		119

For peer review only - http://bmjopen.bmj.com/site/about/guidelines.xhtml

PE: primary endpoint; **SE:** secondary endpoint; **Rx only**: treatment only
Immunisation days highlighted in green, primary end point days in red, days for treatment only in grey
(**x**) performed if clinically indicated

1. Screening and enrolment into Project A will take place about 8 weeks before immunisation 0 to allow initiation of the praziquantel intervention.
2. Week 32 is primary endpoint for responses to Td given at 28 weeks; secondary endpoint for HPV booster
3. Treatments given after sampling when schedules coincide
4. Week 8 HPV dose will be given for previously-unvaccinated girls aged ≥14 years
5. Week 52 Td booster dose will be provided as a service
6. Exploratory immunology blood volume will be guided by guidelines from Harvard Mass General, where a maximum of 3ml/kg body weight is taken at any one time point and not more than 3ml/kg is taken over any 8-week period (ref http://www.drgreene.com/21_1616.html.) These guidelines have been followed in a previous study vaccinating adolescents with investigational tuberculosis vaccine MVA85A (in Uganda).[1] The total blood volume planned is 68 ml over the initial intensive sampling period of 8 weeks. Revision of sample volumes based on weight will only be required for participants who weigh less than 22 kg; the average weight of children aged 9 years is expected to be 28kg (with 21kg the 3rd centile) with greater weights for older children.[2]
7. At baseline, it will only be Hb estimation by Haemocue
8. Oral typhoid vaccine doses will be administered on three alternate days namely visit 5, 5.1 and 5.2.
9. The first PZQ treatment at week -6 will be administered at the end of the screening visit

22

For peer review only

Further information on recruitment criteria

- Participants who are excluded from the trial because they have been discovered (during screening procedures) to be suffering from a previously undiagnosed condition thought to require further medical attention will be referred appropriately for further investigation and treatment.
- Participants discovered to have severe anaemia will be excluded from the trial and treated for anaemia
- Participants discovered to be HIV-positive will be counselled and offered a CD4 T-cell count and referred to a provider of antiretroviral treatment ("Test and Treat" – i.e. initiation of treatment regardless of CD4 count is recommended for these high-risk communities).
- Participants discovered to be pregnant will be counselled and referred to an antenatal clinic of their choice.

This trial proposes to recruit all participants within a short time-frame. It will not, therefore, be possible to reconsider enrolment of potential participants with temporary exclusion criteria after treatment and resolution of the condition.

Further rationale for the selection of vaccines

Bacillus Calmette–Guérin (BCG)

BCG is a live, replicating parenteral vaccine, and the only licensed vaccine against TB. The BCG vaccine for these studies will be obtained from the Serum Institute of India either directly, or through a supplier in Uganda. The Serum Institute of India provides much of the BCG vaccine used in Uganda.

Worldwide, TB is among the top 10 causes of death; Uganda has an estimated incidence of 202/100,000 people.[3] Infectious, sputum positive, pulmonary TB classically emerges in adolescence, driving the on-going epidemic.[4] Thus adolescent booster immunisation is a key TB control strategy.[5] However, BCG vaccine response and efficacy are often impaired in tropical and rural settings[6-8] and new TB vaccines are similarly affected.[9] In the past, the WHO has been hesitant to recommend BCG re-vaccination. However, in 2017 WHO's Strategic Advisory Group of Experts (SAGE) recommended: "Further research is warranted to explore whether certain sub-groups of age, geographic or *M. tuberculosis* exposure categories would benefit from re-vaccination."[10] Recent results suggest that, despite the variability of BCG efficacy between populations, BCG vaccination in adolescence offers benefit in some tropical settings, especially for individuals who are not yet infected with *Mycobacterium tuberculosis*, and may also be cost-effective.[7 11] Also, BCG vaccine is currently being used among adolescents in South Africa as a comparator in a trial of a novel TB vaccine (trial

For peer review only

registration NCT02075203). BCG efficacy in Ugandan adolescents, and differences in BCG vaccine responses between urban and rural Ugandan populations, have not been tested. Information obtained from this study is expected to further inform the use of BCG in adolescents, and also to inform the development of new vaccines for tuberculosis.

Yellow fever vaccine

Yellow fever vaccine YF-17D is a live replicating parenteral vaccine. The vaccine (Stamaril; Sanofi Pasteur) is available for purchase in Uganda. Yellow Fever (YF) causes outbreaks in Uganda and the wider region[12] and YF-17D is a candidate for Uganda's expanded programme on immunisation (EPI). Lower vaccine replication, lower neutralising antibody induction, and greater waning, are described in Uganda compared to Switzerland.[13] YF-17D is a potential vector for novel vaccine constructs,[14] adding relevance to vaccine development.

Typhoid vaccine Ty21a

Typhoid vaccine Ty21a is a live replicating oral vaccine and also a potential vector for new vaccine constructs.[15] The Ty21a vaccine will be purchased from PaxVax, Redwood City, California. Substantial, multi-year typhoid outbreaks occur in Uganda and immunisation campaigns have been advocated as cost effective.[16] Schistosomiasis has been associated with prolonged *S. typhi* infection[17] and impaired antibody responses to killed typhoid vaccines.[18]

Ty21a was developed in the 1970s. Although not routinely used in Uganda, it has been (and is currently) registered in many countries. It was first registered in the United States and United Kingdom in the 1980s, and is recommended by the WHO for both endemic and epidemic settings.[19] It has comparable efficacy to the parenteral Vi polysaccharide typhoid vaccine, good durability and minimal adverse effects.[19] It is proposed for use in this study to model effects of study exposures and intervention on the response to a live oral vaccine.

The Ty21a vaccine is given as a three-dose regimen on alternate days.

Human Papilloma Virus (HPV) vaccine

The Human Papilloma Virus (HPV) vaccine is a protein virus-like particle. The quadrivalent HPV Vaccine Gardasil (Merck) is available for purchase in Uganda and is the vaccine used by the national EPI programme. Studies after three vaccine doses have found somewhat enhanced responses in the presence of malaria, but no effect of helminths.[20] No study has previously investigated parasite effects on the priming response, but recent results for tetanus suggest that priming may be more susceptible than boosting to adverse effects.[21] This will be important if forthcoming trials support single-dose HPV immunisation (NCT02834637). HPV immunisation is being rolled out among girls to

For peer review only - http://bmjopen.bmj.com/site/about/guidelines.xhtml

prevent cervical neoplasia, the most common cancer among Ugandan women and we will coordinate provision with the national HPV immunisation programme.[22] HPV immunisation is also beneficial for boys since HPV infection is associated with anogenital warts, anal cancer and oropharyngeal cancers in both males and females, and with penile cancer in men,[23] and we will include boys in these studies.

Tetanus and diphtheria vaccines

Tetanus and diphtheria (Td) vaccines comprise inert toxoids. Schistosomiasis is associated with a Th2 biased response to tetanus toxoid[24] and with suppressed antibody responses among those with low pre-immunisation antibody levels.[21] Booster immunisation is recommended for young women to prevent maternal and neonatal tetanus. Recent evidence emphasises the need to protect young men also.[25]

Immunisation Postponement Criteria

If any one of the following is identified at the time scheduled for immunisation, the participant may be immunised at a later date, or withdrawn, at the discretion of the Investigator. The participant must be followed until resolution of the event as with any adverse event:

- Acute disease at the time of immunisation. Acute disease is defined as the presence of a moderate or severe illness with or without fever. All vaccines can be administered to persons with a minor illness such as diarrhoea or mild upper respiratory infection with or without low-grade fever, i.e. temperature of ≤37.5°C (99.5°F)
- Temperature of >37.5°C (99.5°F) at the time of immunisation
- Taking antibiotics or antimalarials currently, or within the past 7 days, of the date of Ty21a administration (ascertained verbally)

Vaccine storage and transport

In order to maintain a reliable vaccine cold chain, the vaccines and diluents to be used will be stored and transported within the recommended temperature range of +2°C to +8°C. Care will be taken to ensure that the vaccines are not frozen. BCG, being sensitive to light, will be kept in the dark (normally within its secondary packaging) for as long as possible to protect it during storage and transportation. All vaccines will be kept in appropriate refrigeration equipment with a temperature monitoring device to ensure temperatures remain between +2°C and +8°C. Cold boxes/vaccines carriers with temperature monitors will be used to transport vaccines and the diluents from the MRC/UVRI and LSHTM Uganda Research Unit (Entebbe) to Koome island and while transporting vaccines to immunisation sessions. Designated staff will be given responsibility for managing the

For peer review only

For peer review only - http://bmjopen.bmj.com/site/about/guidelines.xhtml

vaccine cold chain. All cold chain equipment including the temperature monitoring devices used for this project will comply with relevant technical specifications as defined by the EPI standards. Basic routine maintenance will be regularly carried out on all cold chain equipment.

Additional laboratory measurements

Additional assays will comprise HIV serology, pregnancy testing and full blood counts. HIV testing and pregnancy testing will be accompanied by appropriate counselling by trained staff.

- HIV serology will be done on blood samples using rapid tests and according to prevailing national algorithms.[26] This will be done at baseline.
- Pregnancy testing will be done using urine samples and standard operating procedures for assessment of urine β-human chorionic gonadotropin (βhCG). This will be done at baseline and before immunisation on each immunisation day.
- Full blood counts will be conducted using a haematology analyser. Mild, moderate and severe anaemia will be defined according to WHO guidelines, by age.[27] This will be done at baseline to test for anaemia as part of the eligibility assessment, and pre-immunisation as part of the assessment of immunological profile.

Individuals found to be HIV positive or pregnant will be referred to appropriate providers for further care. Individuals with severe anaemia (haemoglobin <82g/L) will be excluded from the randomised intervention (since the intervention might be beneficial in management of anaemia). They will be treated for anaemia.

Sample handling and archive

Blood and other samples will be processed according to local laboratory standard operating procedures (SOPs). All samples will reach the laboratory in anonymised form.

A sample archive will be developed. Although our current programme of work will address specific hypotheses regarding pathways of effects of parasites and interventions, the sample archive will provide a major asset for exploration of new leads arising from this work, or for an alternative, "systems biology" approach employing (for example) proteomic, genomic, epigenetic and transcriptomic analyses, and investigating the microbiome and virome. Information provided to participants, and consent forms, will include considerations of sample storage, and the possibility of sample analysis in laboratories within and outside Uganda. Participants will be able to decide if they will permit such future use of any leftover samples. We plan to store the samples for up to 20 years. If further storage is needed after that time, permission will be requested from the Uganda Virus Research Institute and London School of Hygiene and Tropical Medicine ethical review committees.

For peer review only - http://bmjopen.bmj.com/site/about/guidelines.xhtml

If they elect not to permit this, all of those leftover samples will be discarded after the completion of the work included in the current protocol.

Operational considerations

Programme governance

A Programme Steering Committee has been set up to guide progress across all projects. This comprises the following:

- An independent chair
- Representatives from the Ministry of Health programmes for immunisation and for vector borne disease control
- Representatives of district authorities (Mukono and Jinja districts)
- Community representatives
- Principal investigator and co-investigators
- Project leader and post-doctoral immunologist
- Trial statistician
- Laboratory manager
- Medical Research Council observer

Informed consent

Both written informed assent from the participants and written informed consent from a parent or guardian will be required for participation, although these may not necessarily be obtained at the same time. Information will be provided in both English and the appropriate local language. For individuals who cannot speak the languages used, or who cannot read or write, a witness who can read the information sheet and translate the information to the participant or parent/guardian will be used. Two different types of age specific assent forms will be used for the group of participants aged 9 – 12 years and for the group aged 13 – 17 years. Informed consent by emancipated or mature minors will be obtained using a designated consent form for these categories of participants.

The aims of the study, all tests, treatments and immunisations to be carried out and potential risks will be explained. The participant will be given the opportunity to ask about details of the trial, and will then have time to consider whether or not to participate. If they do decide to participate, they and their parent/guardian will sign and date two copies of the assent and consent forms, one for them to take away and keep, and one to be stored securely by the research team. Separate information and consent forms will be provided (i) for consent for storage of samples for future

For peer review only

For peer review only - http://bmjopen.bmj.com/site/about/guidelines.xhtml

studies and for anonymous sharing of data from this study and (ii) for possible genetic studies; the information sheet will explain that these data may be used in analyses related to this protocol.

Screening and Eligibility Assessment

Once the informed consent process has been completed, and consent (and assent) given, a baseline medical history (including concomitant medication) will be collected. Vital signs will be checked and a physical examination will be performed. Inclusion and exclusion criteria will be checked.

Participants will undergo pre- and post-test counselling for HIV and (for girls) pregnancy testing by a trained and experienced nurse- or clinician-counsellor. Blood, urine and stool samples will be obtained, for tests as specified in the schedule of procedures. These tests are to exclude the major, immunomodulating co-infection, HIV, and conditions that might impact safety (anaemia, pregnancy).

Enrolment

Participants who consent/assent, complete the screening processes, satisfy all the inclusion criteria and meet none of the exclusion criteria will be enrolled.

Discontinuation/withdrawal criteria

In accordance with the principles of the current revision of the Declaration of Helsinki and any other applicable regulations, a participant has the right to withdraw from the study at any time and for any reason, and is not obliged to give his or her reasons for doing so. The Investigator may withdraw the participant at any time in the interests of the participant's health and well-being. In addition, the participant may withdraw/be withdrawn for any of the following reasons:

- Ineligibility (either arising during the study or retrospectively, having been overlooked at screening)
- Administrative decision by the Investigator
- Significant protocol deviation
- Participant non-compliance with study requirements
- An adverse event which requires discontinuation of the study involvement or results in inability to continue to comply with study procedures.

Any participant who becomes pregnant during the trial will be followed up until the end of the pregnancy but no further immunisations will be given unless indicated during pregnancy (as is the case for tetanus toxoid). The trial allocation for this participant will be unblinded and the participant will only be given further treatment if clinically indicated. The babies will also be followed up and

For peer review only - http://bmjopen.bmj.com/site/about/guidelines.xhtml

examined for any adverse effects. We will not routinely perform venepuncture in a pregnant participant.

The reason for withdrawal will be recorded in the case report form (CRF). If withdrawal is due to an AE, appropriate follow-up visits or medical care will be arranged, with the agreement of the participant, until the AE has resolved, stabilised or a non-trial related causality has been assigned.

If a participant withdraws from the study samples collected before their withdrawal from the trial will be used/ stored unless the participant specifically requests otherwise.

Trial discontinuation

The trial will be discontinued in the event of new scientific information that renders continuation futile or unethical, or for any other reason, at the discretion of the Programme Steering Committee.

End of study definition

The trial will be completed when the last participant enrolled into the trial has completed their final follow up visit.

Safety assessments and oversight

No new investigational drug or product will be used in the proposed trial. However, standard approaches for monitoring safety and reporting of serious adverse events will be followed.

Monitoring

The trial will be monitored by both internal and external monitors according to a pre-defined monitoring plan which will include a site initiation visit, monitoring visits at least annually, and a close-out visit. The monitors will assess patient safety, data integrity, and adherence to the protocol and to Good Clinical Research Practice procedures.

Considerations regarding standard of care

S. mansoni infection status will be determined retrospectively through assays conducted in bulk on stored samples (plasma CAA). These results will not, therefore, be useful to determine management of individual participants.

Participants in the standard treatment arm will receive lower levels of anthelminthic treatment. However, all trial arms will receive a minimum of well-implemented national standard of care.

Standard of care will comprise annual praziquantel treatment. Our own results from the Lake Victoria Island Intervention Study on Worms and Allergy-related diseases (LaVIISWA),[28] which compared annual versus quarterly intervention for schistosomiasis at community level over three

For peer review only - http://bmjopen.bmj.com/site/about/guidelines.xhtml

years, showed no advantage of quarterly treatment for morbidity outcomes attributed to schistosomiasis.

Schistosomiasis can cause anaemia. To manage the expected differential benefits of the interventions for anaemia, a full blood count will be performed at baseline, as discussed above; anaemic children will be managed appropriately and severely anaemic children excluded.

Albendazole will be provided twice a year to manage nematode infections (after collection of primary and secondary endpoint samples).

Procedures to be followed in the event of abnormal findings

Abnormal clinical findings from medical history, examination or blood tests will be assessed as to their clinical significance throughout the trials. If an abnormal test result is deemed clinically significant, it may be repeated. If a test remains clinically significant, the participant will be informed and appropriate medical care arranged as appropriate and with the permission of the participant. Specific details regarding findings, discussion with participants and resulting actions will be recorded in the clinical records. Decisions to exclude the participant from enrolling in the trial or to withdraw a participant from the trial will be at the discretion of the Investigator.

Data and Safety Monitoring Board (DSMB)

A data and safety monitoring board (DSMB) has been appointed to provide real-time safety oversight. The DSMB will be notified within 7 days of the Investigators' being aware of the occurrence of SAEs. The DSMB may recommend the Investigators to place the trial on hold if deemed necessary following an intervention-related SAE. The DSMB will be chaired by a clinician experienced in clinical trials. There will be a minimum of two other appropriately qualified committee members. In the case of events related to a blinded intervention, the DSMB can request unblinding. Membership will include a statistician, and at least one Ugandan member. All correspondence between Investigators and the DSMB will be conveyed by the Principal Investigator to the trial Sponsor. The Chair of the DSMB will be contacted for advice and independent review by the Investigator or trial Sponsor in the following situations:

- The occurrence of any SAE
- Any other situation where the Investigator or trial Sponsor feels independent advice or review is important

Ethical and regulatory considerations

Further information regarding risks

For peer review only

The immunisations to be given have recognised side effects which are usually mild and resolve spontaneously in a few days to one week. Parenteral vaccines are likely to result in pain and swelling at the site of injection and mild fever; very occasionally pain and swelling can be severe and associated with difficulty in moving the shoulder. Sometimes headache and tiredness occur. Rarely a vaccine may cause a severe allergic reaction. For most vaccines this is estimated at less than one in a million doses (but 1 in 55,000 for Yellow Fever vaccine).[29] Individuals with a history of a possible allergic reaction to drugs or vaccines, or to vaccine components including eggs or chicken proteins, will be excluded from the studies. The research team will be trained and prepared to manage severe allergic reactions.

Adverse reactions to Yellow Fever vaccine include severe nervous system reaction (about 1 person in 125,000) and severe, life-threatening illness with organ failure (about 1 person in 250,000). The mortality for this severe, life-threatening adverse effect is reported as about 50%.[29]

BCG immunisation is likely to induce a scar in many cases. This may develop over several weeks, starting as a small papule at the injection site which may become ulcerated and then heal over a period of 2 to 5 months; and lymphadenopathy may develop. Occasionally a more severe local reaction occurs (estimated at 1 per 1,000-10,000 doses): for example, an abscess develops and scars may develop into keloids. Rarely BCG can cause disseminated disease (1 per 230,000 to 640,000 doses), or disease in sites remote from the immunisation site. Disseminated BCG disease usually occurs in immunocompromised people: HIV positive people will be excluded from these studies.[30] BCG "pre-immunisation" may interfere with the response to the subsequent live vaccines; indeed our hypothesis, and published results, suggest that it may suppress replication of YF 17D vaccine.[31] However, this reduced replication has not been shown to correlate with, or result in, reduced levels of neutralising antibody titres (which are the desired protective outcome).[13 31]

Oral typhoid vaccine (Ty21a) may occasionally be associated with stomach pain, nausea, vomiting and (rarely) rash.[29]

Praziquantel has been in use for about 30 years. It has a well-recognised profile of side effects including dizziness, nausea, vomiting, abdominal pain, diarrhoea (sometimes with blood) and urticarial rash. The symptoms are considered to arise largely from the effects of killing worms and to be more severe in people with heavy infections. Symptoms are better tolerated when the drugs are given after food and we will provide treatment after a meal or snack. Simple medications, such as paracetamol and cetirizine, can alleviate symptoms and these will be available on treatment days.

For peer review only - http://bmjopen.bmj.com/site/about/guidelines.xhtml

References

1. Wajja A, Kizito D, Nassanga B, et al. The effect of current Schistosoma mansoni infection on the immunogenicity of a candidate TB vaccine, MVA85A, in BCG-vaccinated adolescents: An open-label trial. *PLoS neglected tropical diseases* 2017;11(5):e0005440. doi: 10.1371/journal.pntd.0005440 [published Online First: 2017/05/05]

2. WHO. Growth reference 5-19 years. 2007

3. WHO. Global tuberculosis report 2016 http://www.who.int/tb/publications/global_report/en/ (accessed 17 June 2017), 2016.

4. Alcais A, Fieschi C, Abel L, et al. Tuberculosis in children and adults: two distinct genetic diseases. *J Exp Med* 2005;202(12):1617-21. doi: 10.1084/jem.20052302

5. Weiner J, 3rd, Kaufmann SH. Recent advances towards tuberculosis control: vaccines and biomarkers. *J Intern Med* 2014;275(5):467-80. doi: 10.1111/joim.12212

6. Fine PE. Variation in protection by BCG: implications of and for heterologous immunity. *Lancet (London, England)* 1995;346(8986):1339-45.

7. Barreto ML, Pilger D, Pereira SM, et al. Causes of variation in BCG vaccine efficacy: examining evidence from the BCG REVAC cluster randomized trial to explore the masking and the blocking hypotheses. *Vaccine* 2014;32(30):3759-64. doi: 10.1016/j.vaccine.2014.05.042

8. Black GF, Weir RE, Floyd S, et al. BCG-induced increase in interferon-gamma response to mycobacterial antigens and efficacy of BCG vaccination in Malawi and the UK: two randomised controlled studies. *Lancet (London, England)* 2002;359(9315):1393-401. doi: 10.1016/S0140-6736(02)08353-8

9. Tanner R, Kakalacheva K, Miller E, et al. Serum indoleamine 2,3-dioxygenase activity is associated with reduced immunogenicity following vaccination with MVA85A. *BMC infectious diseases* 2014;14:660. doi: 10.1186/s12879-014-0660-7 [published Online First: 2014/12/04]

10. WHO. SAGE Evidence to recommendations framework. 2017. http://www.who.int/immunization/sage/meetings/2017/october/2_EvidencetoRecommendationFramework_BCG.pdf (accessed 16th March 2018).

11. Dye C. Making wider use of the world's most widely used vaccine: Bacille Calmette-Guerin revaccination reconsidered. *Journal of the Royal Society, Interface* 2013;10(87):20130365. doi: 10.1098/rsif.2013.0365 [published Online First: 2013/08/02]

12. Kraemer MU, Faria NR, Reiner RC, Jr., et al. Spread of yellow fever virus outbreak in Angola and the Democratic Republic of the Congo 2015-16: a modelling study. *The Lancet Infectious diseases* 2016 doi: 10.1016/s1473-3099(16)30513-8 [published Online First: 2016/12/27]

13. Muyanja E, Ssemaganda A, Ngauv P, et al. Immune activation alters cellular and humoral responses to yellow fever 17D vaccine. *The Journal of clinical investigation* 2014;124(7):3147-58. doi: 10.1172/jci75429 [published Online First: 2014/06/10]

14. Aguiar M, Stollenwerk N, Halstead SB. The Impact of the Newly Licensed Dengue Vaccine in Endemic Countries. *PLoS neglected tropical diseases* 2016;10(12):e0005179. doi: 10.1371/journal.pntd.0005179 [published Online First: 2016/12/22]

15. Dharmasena MN, Osorio M, Filipova S, et al. Stable expression of Shigella dysenteriae serotype 1 O-antigen genes integrated into the chromosome of live Salmonella oral vaccine vector Ty21a. *Pathogens and disease* 2016 doi: 10.1093/femspd/ftw098 [published Online First: 2016/09/23]

For peer review only - http://bmjopen.bmj.com/site/about/guidelines.xhtml

16. Carias C, Walters MS, Wefula E, et al. Economic evaluation of typhoid vaccination in a prolonged typhoid outbreak setting: the case of Kasese district in Uganda. *Vaccine* 2015;33(17):2079-85. doi: 10.1016/j.vaccine.2015.02.027 [published Online First: 2015/02/26]

17. Melhem RF, LoVerde PT. Mechanism of interaction of Salmonella and Schistosoma species. *Infection and immunity* 1984;44(2):274-81. [published Online First: 1984/05/01]

18. Muniz-Junqueira MI, Tavares-Neto J, Prata A, et al. Antibody response to Salmonella typhi in human schistosomiasis mansoni. *Revista da Sociedade Brasileira de Medicina Tropical* 1996;29(5):441-5. [published Online First: 1996/09/01]

19. WHO. Position Paper on Typhoid vaccines: WHO position paper – March 2018 2018

20. Brown J, Baisley K, Kavishe B, et al. Impact of malaria and helminth infections on immunogenicity of the human papillomavirus-16/18 AS04-adjuvanted vaccine in Tanzania. *Vaccine* 2014;32(5):611-7. doi: 10.1016/j.vaccine.2013.11.061

21. Riner DK, Ndombi EM, Carter JM, et al. Schistosoma mansoni Infection Can Jeopardize the Duration of Protective Levels of Antibody Responses to Immunizations against Hepatitis B and Tetanus Toxoid. *PLoS neglected tropical diseases* 2016;10(12):e0005180. doi: 10.1371/journal.pntd.0005180

22. Centre HI. HPV and related diseases report: Uganda. 2016. http://www.hpvcentre.net/statistics/reports/UGA.pdf (accessed 20.01.2017).

23. WHO. Human papillomavirus vaccines: WHO position paper, May 2017. *Releve epidemiologique hebdomadaire* 2017;92(19):241-68. [published Online First: 2017/05/23]

24. Sabin EA, Araujo MI, Carvalho EM, et al. Impairment of tetanus toxoid-specific Th1-like immune responses in humans infected with Schistosoma mansoni. *The Journal of infectious diseases* 1996;173(1):269-72. [published Online First: 1996/01/01]

25. Nanteza B, Galukande M, Aceng J, et al. The burden of tetanus in Uganda. *SpringerPlus* 2016;5(1):705. doi: 10.1186/s40064-016-2309-z [published Online First: 2016/06/29]

26. Ministry of Health–Uganda. Uganda Clinical Guidelines 2016. http://apps.who.int/medicinedocs/documents/s23532en/s23532en.pdf

27. WHO. Haemoglobin concentrations for the diagnosis of anaemia and assessment of severity. Vitamin and Mineral Nutrition Information System. Geneva, World Health Organization, 2011 (WHO/NMH/NHD/MNM/11.1).

28. Sanya RE, Nkurunungi G, Hoek Spaans R, et al. The Impact of Intensive Versus Standard Anthelminthic Treatment on Allergy-related Outcomes, Helminth Infection Intensity, and Helminth-related Morbidity in Lake Victoria Fishing Communities, Uganda: Results From the LaVIISWA Cluster-randomized Trial. *Clinical Infectious Diseases* 2018:ciy761-ciy61. doi: 10.1093/cid/ciy761

29. CDC. Centers for Disease Control and Prevention, vaccines and immunizations.

30. WHO. Information sheet observed rate of vaccine reactions Bacille Calmette-Guérin (BCG) vaccine. 2012

31. Arts RJW, Moorlag S, Novakovic B, et al. BCG Vaccination Protects against Experimental Viral Infection in Humans through the Induction of Cytokines Associated with Trained Immunity. *Cell host & microbe* 2018;23(1):89-100.e5. doi: 10.1016/j.chom.2017.12.010 [published Online First: 2018/01/13]

For peer review only

For peer review only - http://bmjopen.bmj.com/site/about/guidelines.xhtml

MRC/UVRI and LSHTM Uganda Research Unit

MRC Medical Research Council | Uganda Virus Research Institute | LONDON SCHOOL of HYGIENE &TROPICAL MEDICINE

Population differences in vaccine responses: the effect of intensive treatment for schistosomiasis on response to vaccines among island adolescents

Short Title: POPVAC Project A

Information Sheet for participants 9 – 12 years

1. Why are we meeting with you?

We are inviting you to take part in a research which is being done by a team from Uganda Virus Research Institute (UVRI), working together with colleagues from the London School of Hygiene and Tropical Medicine, UK. We are meeting you because you are aged between 9 and 12 years and study in a primary school on Koome island. After we tell you about it, we will ask if you'd like to be in this study or not. Only If you agree to take part, will you sign the assent form to show us that you are happy to do so.

The people leading this research study are Professor Alison Elliott and Dr Ludoviko Zirimenya.

2. Why are we doing this research?

We want to **find out whether treating Bilharzia much more frequently (a total of 7 times in a year) can get rid of it completely and whether this will improve vaccine responses.**

In the whole study, there will be about 480 children from schools of Koome islands.

3. What is going to happen in this research study?

Only if you agree, a full health check-up that will involve taking stool, urine and blood samples will be done. If everything is okay, the following will be done:

You will be put into one of the two **Bilharzia treatment** groups by chance

- One group will be treated for Bilharzia using praziquantel (the recommended medicine for Bilharzia) seven times in the year, and an eighth time after the end of the study.
- The other group will be treated for Bilharzia using praziquantel once in the year, and a second time after the end of the study.

You will be immunised with **five (5) vaccines**. These are:

- BCG vaccine. This is intended to protect against tuberculosis.
- Yellow fever vaccine. This protects against Yellow Fever an infection carried by mosquitoes.
- HPV vaccine. Human Papilloma Virus (HPV) causes growths in private parts and cancer in girls and boys.
- Tetanus and diphtheria vaccines. Tetanus and diphtheria are bacterial infections that cause very serious disease.
- Typhoid vaccine. Typhoid is a serious form of fever which is spread through contaminated food.

These immunisations will be given on different times during the entire duration of the study.

UGANDA VIRUS RESEARCH INSTITUTE ENTEBBE
RESEARCH ETHICS COMMITTEE
APPROVED
0 5 SEP 2020
P. O. BOX 49, ENTEBBE
UGANDA

For peer review only - http://bmjopen.bmj.com/site/about/guidelines.xhtml

Most of the vaccines will be injected into your upper arm (either right or left side). The typhoid vaccine is an oral vaccine: it is in the form of capsules which are swallowed. You will be given one capsule per day for three alternating days.

You will be asked to give **blood samples**

We will ask you to give blood samples before and after you are given the vaccines. The amount of blood that we will take at each visit is completely safe and will vary between 4 and 27 millilitres (1 teaspoon and 5 and a half teaspoons) at the different visits.

You will be asked to give **stool and urine samples**

At some visits we will ask you for additional stool and urine samples for testing to check whether the worm infections have responded to treatment or (for girls) whether you have become pregnant.

Some of you will be asked to take part in a special **sugar test** of absorption from the intestines

We think that worm infections may make our intestines a little bit leaky, and this might explain some of their effects. We can test this using a special sugar drink. We will ask about 200 people to do this additional test. If you are asked to do this, it will be done about 8 weeks after your first vaccine is given. You will be asked not to eat overnight or in the morning of the test day. Then you will be given a special drink that contains sugars early in the morning. Afterwards you will also be asked to drink a litre of water (equal to two Rwenzori bottles) over the following few hours. All the urine that you pass during the next five hours will be collected. The volume (amount) of urine will be measured and the amount of sugar in it will be tested, to find out how much of the sugar your body has absorbed.

4. What will the blood, stool and urine samples be used for?

The samples will be used to test for infections, including HIV, malaria and worms, to test for anaemia (the strength of your blood) and to test for pregnancy (among girls). The blood samples will also be used to test how your body responds to vaccines. For some participants, the sugar test will be used to test absorption from the intestines.

5. Will taking part in this study harm me?

We do not expect this to harm you, though you will experience the following:

- You will need to take time off classes during each visit by the study team.
- Taking blood samples is not expected to cause any problem for you, apart from the discomfort or pain, bruising or bleeding, redness at the place where the needle goes into the skin, but this will go away in a few days.
- During the sugar test, you will be asked not to eat overnight and during the morning of the test so you may get hungry (although water and a snack will be provided). This may make you feel a little sick and stools may become a bit loose.
- If you are a girl, even if you know you could not be pregnant, we will do a pregnancy test. We have to be quite sure. You must as well promise that you can avoid getting pregnant while taking part in this study.

UGANDA VIRUS RESEARCH INSTITUTE ENTEBBE
RESEARCH ETHICS COMMITTEE
APPROVED
05 SEP 2020
P. O. BOX 49, ENTEBBE
UGANDA

For peer review only - http://bmjopen.bmj.com/site/about/guidelines.xhtml

MRC/UVRI and LSHTM Uganda Research Unit

MRC Medical Research Council | Uganda Virus Research Institute | LONDON SCHOOL of HYGIENE &TROPICAL MEDICINE

- Treatment of Bilharzia with praziquantel may make you feel dizzy or sick, give you abdominal pain or diarrhoea, or occasionally cause an itchy rash. The research team will have medicines available to help you if you have a strong reaction to the treatment.
- All the study vaccines are known to be safe. However, even approved vaccines may very occasionally cause a serious reaction, such as an allergic reaction. The research doctors and nurses will be available to help if this happens.

6. Will the study help me?

The treatment for Bilharzia is likely to be good for you, whichever group you are put in. The vaccines are likely to help you by protecting you from infectious diseases. The research results may help all people in the world, because in the end we may get a better understanding whether treating Bilharzia much more frequently (a total of 7 times in a year) can get rid of it completely and whether this will improve vaccine responses.

7. What is the cost of taking part in the trial?

There is no cost to participate in this trial. You will receive a soft drink and the gift of a pen or other simple school material on days when blood samples are drawn, and the gift of a T-shirt at the end of the study when everything is done, in appreciation of your contribution to the work.

8. What happens if I refuse to take part?

It is very important for you to know that you do not have to take part in the research, the choice is yours. No-one will be upset if you decide not to take part. If you agree to take part and later decide that you do not want to take part anymore, that is also okay.

9. What happens if something goes wrong?

The researchers will make every effort to ensure your safety and well-being. MRC/UVRI and LSHTM has a specialist insurance policy in place which would help to pay for treatment if any harm came to you as a result of taking part in the research.

10. Who will have access to your information and samples from this research?

Only research staff trained to keep the information confidential will have access to the records. Your name will be removed from the records and samples, so no-one will be able to find out information about you from our records.

11. Who has reviewed the trial?

This trial has been reviewed by the Uganda National Council for Science and Technology, The Uganda National Drug Authority, the Uganda Virus Research Institute Research and Ethics Committee the ethics committee of the London School of Hygiene and Tropical Medicine in the United Kingdom.

You can find out more about the study at any time by asking one of the members of the research team. You may also contact Professor Alison Elliott (telephone: 0417 704000) who is in charge of the study, or the Chairperson of the Ethics Committee for the Uganda Virus Research Institute on 0414 321962.

UGANDA VIRUS RESEARCH INSTITUTE ENTEBBE
RESEARCH ETHICS COMMITTEE
APPROVED
05 SEP 2020
P.O. BOX 49, ENTEBBE
UGANDA

For peer review only - http://bmjopen.bmj.com/site/about/guidelines.xhtml

MRC/UVRI and LSHTM Uganda Research Unit

MRC Medical Research Council

Uganda Virus Research Institute

LONDON SCHOOL of HYGIENE &TROPICAL MEDICINE

Population differences in vaccine responses: the effect of intensive treatment for schistosomiasis on response to vaccines among island adolescents

Short Title: POPVAC Project A

Participant Assent

A copy of this form will be given to you. Please keep a copy of the form because it contains the information that was discussed with you and you may want to look at it again.

AGREEMENT TO TAKE PART:

I have read and understood (or been read to and understand) the information sheet for this study. My questions have been answered. I understand that taking part in the study is voluntary. I understand that at any time I may withdraw from this study without giving a reason. I agree to participate in this study.

Name: ______________________________ **PVA ID: |A|___|___|___|___|**
(please write your name in capital letters here if you agree)

Signature: ______________________________ **Date:**________________
(please sign or write your name here if you agree; or use a thumbprint)

What if I have any questions?
If you have any questions about your participation in this study, please feel free to ask any member of the research team at any time. If you prefer, you may speak to the principal investigator for this study (Professor Alison Elliott, telephone 0417 704000).

What if we want to ask someone independent anything about this research, or have any questions about your rights as a research participant? You may speak with the Chairman of the Science and Ethics Committee at Uganda Virus Research Institute on 0414 321962.

UGANDA VIRUS RESEARCH INSTITUTE ENTEBBE
RESEARCH ETHICS COMMITTEE
APPROVED
05 SEP 2020
P. O. BOX 49, ENTEBBE
UGANDA

For peer review only - http://bmjopen.bmj.com/site/about/guidelines.xhtml

PVA ID: |A|___|___|___|___|

Witness:

I have read the participant information sheet and the assent statement above

to:.. (PRINT NAME OF PARTICIPANT) in a language which he/she understands. I believe that he/she gives assent to take part in the study.

.... Witness name	.... Signature	.... Date

Witness required only for those using a thumb print instead of the final signature, or unable to read the information and consent form, or if the person taking consent does not speak the participant's language. The witness must not be a member of the research staff or a study participant. The witness must be present for the whole consent process.

Person taking the assent:

.... Researcher name	.... Signature	.... Date

**Comment:**

UGANDA VIRUS RESEARCH INSTITUTE ENTEBBE
RESEARCH ETHICS COMMITTEE
APPROVED
VALID UNTIL
05 SEP 2020
P. O. BOX 49, ENTEBBE
UGANDA

For peer review only - http://bmjopen.bmj.com/site/about/guidelines.xhtml

MRC/UVRI and LSHTM Uganda Research Unit

MRC Medical Research Council | Uganda Virus Research Institute | LONDON SCHOOL of HYGIENE &TROPICAL MEDICINE

Population differences in vaccine responses: the effect of intensive treatment for schistosomiasis on response to vaccines among island adolescents

Short Title: POPVAC Project A

Information Sheet for participants aged 13 to 17 years

We are inviting you to join in some research which is being done by a team from Uganda Virus Research Institute (UVRI), working together with colleagues from the London School of Hygiene and Tropical Medicine, UK. This form will tell you about the research. If you have any questions, please ask the person who is telling you about this research. Then you can decide whether you want to take part or not. There is no need to take part unless you really wish to. If you agree to take part, you will need to sign the assent form to show us that you are happy to do so. If you decide you do not want to take part, do not sign the assent form.

The people leading this research study are Professor Alison Elliott and Dr Ludoviko Zirimenya.

1. What this research is about, and the reason for doing this research.

This research is about how worm infections like Bilharzia "switch off" the body's defence systems and how this affects vaccine responses. **We want to find out whether treating Bilharzia much more frequently (a total of 7 times in a year) can get rid of it completely and whether this will improve vaccine responses.**

2. Why have I been asked to take part in this study?

You have been asked because you are attending primary school in Koome islands.

3. What is going to happen in this research study?

Only if you agree, a comprehensive health check-up that will involve taking stool, urine and blood samples will be done. If everything is okay, the following will be done:

You will be put into one of the two **Bilharzia treatment** groups by luck

- One group will be treated for Bilharzia using praziquantel (the recommended medicine for Bilharzia) seven times in the year, and an eighth time after the end of the study.
- The other group will be treated for Bilharzia using praziquantel once in the year, and a second time after the end of the study.

You will be immunised with **several vaccines**. These are the vaccines:

- BCG vaccine. This is intended to protect against tuberculosis.
- Yellow fever vaccine. This protects against Yellow Fever virus carried by mosquitoes.
- HPV vaccine. Human Papilloma Virus (HPV) causes growths in private parts and cancer in girls and boys.
- Tetanus and diphtheria vaccines. Tetanus and diphtheria are bacterial infections that cause very serious disease.
- Typhoid vaccine. Typhoid is a serious form of fever which is spread through contaminated food.

These immunisations will be given on three different days.

UGANDA VIRUS RESEARCH INSTITUTE ENTEBBE RESEARCH ETHICS COMMITTEE APPROVED VALID UNTIL 05 SEP 2020 P. O. BOX 49, ENTEBBE UGANDA

For peer review only - http://bmjopen.bmj.com/site/about/guidelines.xhtml

Most of the vaccines will be injected into your upper arm (either right or left side). This will be a bit painful, as for any injection of medicine that you may have had, and will feel a bit sore over the next week or so. The typhoid vaccine is an oral vaccine: it is in the form of capsules which are swallowed. You will be given one capsule per day for three alternating days.

You will be asked to give **blood samples**

We will ask you to give blood samples before and after you are given the vaccines. The amount of blood that we will take at each visit is completely safe and will vary between 4 and 27 millilitres (1 teaspoon and 5 and a half teaspoons) at the different visits

You will be asked to give **stool and urine samples**

At some visits we will ask you for additional stool and urine samples for testing to check whether the worm infections have responded to treatment or (for girls) whether you have become pregnant.

Some of you will be asked to take part in a special **sugar test** of absorption from the intestines

We think that worm infections may make our intestines a little bit leaky, and this might explain some of their effects. We can test this using a special sugar drink. We will ask about 200 people to do this additional test. If you are asked to do this, it will be done about 8 weeks after your first vaccine is given. You will be asked not to eat overnight or in the morning of the test day. Then you will be given a special drink that contains sugars early in the morning. Afterwards you will also be asked to drink a litre of water (equal to two Rwenzori bottles) over the following few hours. All the urine that you pass during the next five hours will be collected. The volume (amount) of urine will be measured and the amount of sugar in it will be tested, to find out how much of the sugar your body has absorbed.

4. What will the blood, stool and urine samples be used for?

The samples will be used to test for infections, including HIV, malaria and worms, to test for anaemia (the strength of your blood) and to test for pregnancy (among girls). The blood samples will also be used to test how your body responds to vaccines. For some participants, the sugar test will be used to test absorption from the intestines.

5. How many people will take part in the study, and how long will it last?

The whole study will enrol 480 people, and the study will last for about two years.

6. What are the risks of participating in this trial?

You will need to take time off classes during each visit by the study team.

Taking blood samples is not expected to cause any problem for you, apart from the discomfort or pain, bruising or bleeding, redness at the place where the needle goes into the skin, but this will go away in a few days.

During the sugar test you will be asked not to eat overnight and during the morning of the test so you may get hungry (although water and a snack will be provided). Most people have no problems with this test although a few may feel a little sick and stools may become a bit loose.

UGANDA VIRUS RESEARCH INSTITUTE ENTEBBE
RESEARCH ETHICS COMMITTEE
APPROVED
05 SEP 2020
P. O. BOX 49, ENTEBBE
UGANDA

For peer review only - http://bmjopen.bmj.com/site/about/guidelines.xhtml

MRC/UVRI and LSHTM Uganda Research Unit

MRC Medical Research Council · Uganda Virus Research Institute · LONDON SCHOOL of HYGIENE &TROPICAL MEDICINE

If you are a girl, even if you know you could not be pregnant. We have to be quite sure. We will test for pregnancy at the beginning of the study and on each immunisation day. You must as well promise that you can avoid getting pregnant while taking part in this study.

Treatment of Bilharzia with praziquantel may make you feel dizzy or sick, give you abdominal pain or diarrhoea, or occasionally cause an itchy rash. This is most likely caused by your body's response to the worms as they are being killed by the medicine. The research team will have medicines available to help you if you have a strong reaction to the treatment.

All the study vaccines are known to be safe though not often, may cause a serious reaction, such as an allergic reaction. The research doctors and nurses will be available to help if this happens.

7. What are the benefits of taking part in this trial?

The treatment for Bilharzia is likely to be good for you, whichever group you are put in. The vaccines are likely to help you by protecting you from infectious diseases.

Also, you will be helping us to find out why vaccines sometimes don't work so well in countries like Uganda, and whether vaccines work better if worms are treated first. This may help other people in the future.

8. What is the cost of taking part in the trial?

There is no cost to participate in this trial. You will receive a soft drink and the gift of a pen or other simple school material on days when blood samples are drawn, and the gift of a T-shirt at the end of the study when everything is done, in appreciation of your contribution to the work.

9. What happens if I refuse to take part?

It is very important for you to know that you do not have to take part in the research, the choice is yours. No-one will be upset if you decide not to take part. If you agree to take part and later decide that you do not want to take part anymore, that is also okay.

10. What happens if something goes wrong?

You will be making an important contribution to medical research. The researchers will make every effort to ensure your safety and well-being. MRC/UVRI and LSHTM has a specialist insurance policy in place which would help to pay for treatment if any harm came to you as a result of taking part in the research.

11. Who will have access to my information and samples from this research?

All our research records are stored securely in rooms with restricted access and on password protected computers. Only research staff trained to keep the information confidential will have access to the records. Your name will be removed from the records, so no-one will be able to find out information about you from our records.

12. Who has reviewed the trial?

This trial has been reviewed by the Uganda National Council for Science and Technology, Uganda National Drug Authority the Uganda Virus Research Institute Research and Ethics Committee the ethics committee of the London School of Hygiene and Tropical Medicine in the United Kingdom.

UGANDA VIRUS RESEARCH INSTITUTE ENTEBBE
RESEARCH ETHICS COMMITTEE
APPROVED
VALID UNTIL
05 SEP 2020
P. O. BOX 49, ENTEBBE
UGANDA

Medical Research Council MRC | Uganda Virus Research Institute | LONDON SCHOOL of HYGIENE &TROPICAL MEDICINE

You can find out more about the study at any time by asking one of the members of the research team. You may also contact Professor Alison Elliott (telephone: 0417 704000) who is in charge of the study, or the Chairperson of the Ethics Committee for the Uganda Virus Research Institute on 0414 321962.

UGANDA VIRUS RESEARCH INSTITUTE ENTEBBE
RESEARCH ETHICS COMMITTEE
APPROVED
05 SEP 2020
P. O. BOX 49, ENTEBBE
UGANDA

For peer review only - http://bmjopen.bmj.com/site/about/guidelines.xhtml

MRC/UVRI and LSHTM Uganda Research Unit

MRC Medical Research Council | Uganda Virus Research Institute | LONDON SCHOOL of HYGIENE &TROPICAL MEDICINE

Population differences in vaccine responses: the effect of intensive treatment for schistosomiasis on response to vaccines among island adolescents

Short Title: POPVAC Project A

Participant Assent

A copy of this form will be given to you. Please keep a copy of the form because it contains the information that was discussed with you and you may want to look at it again.

AGREEMENT TO TAKE PART:

I have read and understood (or been read to and understand) the information sheet for this study. My questions have been answered. I understand that taking part in the study is voluntary. I understand that at any time I may withdraw from this study without giving a reason. I agree to participate in this study.

Name: ______________________________ **PVA ID:** |A|___|___|___|___|
(please write your name in capital letters here if you agree)

Signature: ______________________________ **Date:**________________
(please sign or write your name here if you agree; or use a thumbprint)

What if I have any questions?
If you have any questions about your participation in this study, please feel free to ask any member of the research team at any time. If you prefer, you may speak to the principal investigator for this study (Professor Alison Elliott, telephone 0417 704000).

What if we want to ask someone independent anything about this research, or have any questions about your rights as a research participant? You may speak with the Chairman of the Science and Ethics Committee at Uganda Virus Research Institute on 0414 321962.

UGANDA VIRUS RESEARCH INSTITUTE ENTEBBE
RESEARCH ETHICS COMMITTEE
APPROVED
05 SEP 2020
P. O. BOX 49, ENTEBBE
UGANDA

For peer review only - http://bmjopen.bmj.com/site/about/guidelines.xhtml

PVA ID: |A|___|___|___|___|

Witness:

I have read the participant information sheet and the assent statement above

to:.. (PRINT NAME OF PARTICIPANT) in a language which he/she understands. I believe that he/she gives assent to take part in the study.

....	....	....
**Witness name**	**Signature**	**Date**

Witness required only for those using a thumb print instead of the final signature, or unable to read the information and consent form, or if the person taking consent does not speak the participant's language. The witness must be present for the whole consent process.

Person taking the assent:

....	....	....
**Researcher name**	**Signature**	**Date**

Comment:

UGANDA VIRUS RESEARCH INSTITUTE ENTEBBE
RESEARCH ETHICS COMMITTEE
APPROVED
05 SEP 2020
P. O. BOX 49, ENTEBBE
UGANDA

For peer review only - http://bmjopen.bmj.com/site/about/guidelines.xhtml

MRC/UVRI and LSHTM Uganda Research Unit

MRC Medical Research Council | Uganda Virus Research Institute | LONDON SCHOOL of HYGIENE &TROPICAL MEDICINE

Population differences in vaccine responses: the effect of intensive treatment for schistosomiasis on response to vaccines among island adolescents

Short Title: POPVAC Project A

Information Sheet for emancipated or mature minors

We are inviting you to join in some research which is being done by a team from Uganda Virus Research Institute (UVRI), working together with colleagues from the London School of Hygiene and Tropical Medicine, UK. This form will tell you about the research. If you have any questions, please ask the person who is telling you about this research. Then you can decide whether you want to take part or not. If you agree to take part, you will need to sign the consent form to show us that you are happy to do so. If you decide that you do not want to take part, do not sign the consent form.

The people leading this research study are Professor Alison Elliott and Dr Ludoviko Zirimenya.

1. What is this research about?

This research is about how worm infections like Bilharzia "switch off" the body's defence systems and how this affects vaccine responses. **We want to find out whether treating Bilharzia much more frequently (a total of 7 times in a year) can get rid of it completely and whether this will improve vaccine responses.**

2. Why have I been asked to take part in this study?

You have been asked because you are attending primary school in Koome islands.

3. What is going to happen in this research study?

Only if you agree, you will have a comprehensive health check-up that will involve taking stool, urine and blood samples. If everything is okay, the following will be done:

You will be put into one of the two **Bilharzia treatment** groups by chance

One group will be treated for Bilharzia using praziquantel (the recommended medicine for Bilharzia) seven times in the year, and an eighth time after the end of the study.

The other group will be treated for Bilharzia using praziquantel once in the year, and a second time after the end of the study.

UGANDA VIRUS RESEARCH INSTITUTE ENTEBBE
RESEARCH ETHICS COMMITTEE
APPROVED
05 SEP 2020
P. O. BOX 49, ENTEBBE
UGANDA

For peer review only - http://bmjopen.bmj.com/site/about/guidelines.xhtml

<u>You will be immunised with **five (5) vaccines.**</u> These are:

- **BCG vaccine.** This is intended to protect against tuberculosis.
- **Yellow fever vaccine.** This protects against Yellow Fever an infection carried by mosquitoes.
- **HPV vaccine.** Human Papilloma Virus (HPV) causes growths in private parts and cancer in girls and boys.
- **Tetanus and diphtheria vaccines.** Tetanus and diphtheria are bacterial infections that cause very serious disease.
- **Typhoid vaccine.** Typhoid is a serious form of fever which is spread through contaminated food.

These immunisations will be given on different times during the entire duration of the study.

Most of the vaccines will be injected into your upper arm (either right or left side). The typhoid vaccine is an oral vaccine: it is in the form of capsules which are swallowed. You will be given one capsule per day for three alternating days.

<u>You will be asked to give **blood samples**</u>

We will ask you to give blood samples before and after you are given the vaccines. The amount of blood that we will take at each visit is completely safe and will vary between 4 and 27 millilitres (1 teaspoon and 5 and a half teaspoons) at the different visits.

<u>You will be asked to give **stool and urine samples**</u>

At some visits we will ask you for additional stool and urine samples for testing to check whether the worm infections have responded to treatment or (for girls) whether you have become pregnant.

<u>Some of you will be asked to take part in a special **sugar test** of absorption from the intestines</u>

We think that worm infections may make our intestines a little bit leaky, and this might explain some of their effects. We can test this using a special sugar drink. We will ask about 200 people to do this additional test. If you are asked to do this, it will be done about 8 weeks after your first vaccine is given. You will be asked not to eat overnight or in the morning of the test day. Then you will be given a special drink that contains sugars early in the morning. Afterwards you will also be asked to drink a litre of water (equal to two Rwenzori bottles) over the following few hours. All the urine that you pass during the next five hours will be collected. The volume (amount) of urine will be measured and the amount of sugar in it will be tested, to find out how much of the sugar your body has absorbed.

UGANDA VIRUS RESEARCH INSTITUTE ENTEBBE
RESEARCH ETHICS COMMITTEE
APPROVED
05 SEP 2020
P. O. BOX 49, ENTEBBE
UGANDA

For peer review only - http://bmjopen.bmj.com/site/about/guidelines.xhtml

MRC/UVRI and LSHTM Uganda Research Unit

MRC Medical Research Council | Uganda Virus Research Institute | LONDON SCHOOL of HYGIENE &TROPICAL MEDICINE

4. What will the blood, stool and urine samples be used for?

The samples will be used to test for infections, including HIV, malaria and worms, to test for anaemia (the strength of your blood) and to test for pregnancy (among girls). The blood samples will also be used to test how your body responds to vaccines.

5. How many people will take part in the study, and how long will it last?

The whole study will enrol 480 people, and the study will last for about two years.

6. What are the risks of participating in this trial?

- You will need to take time off classes during each visit by the study team.
- Taking blood samples is not expected to cause any problem for you, apart from the discomfort or pain, bruising or bleeding, redness at the place where the needle goes into the skin, but this will go away in a few days.
- During the sugar test, you will be asked not to eat overnight and during the morning of the test so you may get hungry (although water and a snack will be provided). This may make you feel a little sick and stools may become a bit loose.
- If you are a girl, even if you know you could not be pregnant, we will do a pregnancy test. We have to be quite sure. You must as well promise that you can avoid getting pregnant while taking part in this study.
- Treatment of Bilharzia with praziquantel may make you feel dizzy or sick, give you abdominal pain or diarrhoea, or occasionally cause an itchy rash. The research team will have medicines available to help you if you have a strong reaction to the treatment.
- All the study vaccines are known to be safe. However, very occasionally even approved vaccines may cause a serious reaction, such as an allergic reaction. The research doctors and nurses will be available to help if this happens.

7. What are the benefits of taking part in this trial?

The treatment for Bilharzia is likely to be good for you, whichever group you are put in. The vaccines are likely to help you by protecting you from infectious diseases.

Also, you will be helping us to find out why vaccines sometimes don't work so well in countries like Uganda, and whether vaccines work better if worms are treated first. This may help other people in the future.

UGANDA VIRUS RESEARCH INSTITUTE ENTEBBE
RESEARCH ETHICS COMMITTEE
APPROVED
05 SEP 2020
P. O. BOX 49, ENTEBBE
UGANDA

For peer review only - http://bmjopen.bmj.com/site/about/guidelines.xhtml

MRC/UVRI and LSHTM Uganda Research Unit

MRC Medical Research Council | Uganda Virus Research Institute | LONDON SCHOOL of HYGIENE &TROPICAL MEDICINE

8. What is the cost of taking part in the trial?

There is no cost to participate in this trial. You will receive a soft drink and the gift of a pen or other simple school material on days when blood samples are drawn, and the gift of a T-shirt at the end of the study when everything is done, in appreciation of your contribution to the work.

9. What happens if I refuse to take part?

It is very important for you to know that you do not have to take part in the research, the choice is yours. No-one will be upset if you decide not to take part. If you agree to take part and later decide that you do not want to take part anymore, that is also okay.

10. What happens if something goes wrong?

The researchers will make every effort to ensure your safety and well-being. MRC/UVRI and LSHTM has a specialist insurance policy in place which would help to pay for treatment if any harm came to you as a result of taking part in the research.

11. Who will have access to my information and samples from this research?

All our research records and samples are stored securely in rooms with restricted access and on password protected computers. Only research staff trained to keep the information confidential will have access to the them. Your name will be removed from the records, so no-one will be able to find out information about you from our records.

12. Who has reviewed the trial?

This trial has been reviewed by the Uganda National Council for Science and Technology, The Uganda National Drug Authority, the Uganda Virus Research Institute Research and Ethics Committee the ethics committee of the London School of Hygiene and Tropical Medicine in the United Kingdom.

You can find out more about the study at any time by asking one of the members of the research team. You may also contact Professor Alison Elliott (telephone: 0417 704000) who is in charge of the study, or the Chairperson of the Ethics Committee for the Uganda Virus Research Institute on 0414 321962.

UGANDA VIRUS RESEARCH INSTITUTE ENTEBBE
RESEARCH ETHICS COMMITTEE
APPROVED
05 SEP 2020
P. O. BOX 49, ENTEBBE
UGANDA

For peer review only - http://bmjopen.bmj.com/site/about/guidelines.xhtml

MRC/UVRI and LSHTM Uganda Research Unit

MRC Medical Research Council | Uganda Virus Research Institute | LONDON SCHOOL of HYGIENE &TROPICAL MEDICINE

Population differences in vaccine responses: the effect of intensive treatment for schistosomiasis on response to vaccines among island adolescents

Short Title: POPVAC Project A

Participant Consent

A copy of this form will be given to you. Please keep a copy of the form because it contains the information that was discussed with you and you may want to look at it again.

AGREEMENT TO TAKE PART:

I have read and understood (or been read to and understand) the information sheet for this study. My questions have been answered. I understand that taking part in the study is voluntary. I understand that at any time I may withdraw from this study without giving a reason. I agree to participate in this study.

Name: ______________________________ **PVA ID:** |A|___|___|___|___|

(please write your name in capital letters here if you agree)

Signature: ______________________________ **Date:**__________________

(please sign or write your name here if you agree; or use a thumbprint)

What if I have any questions?

If you have any questions about your participation in this study, please feel free to ask any member of the research team at any time. If you prefer, you may speak to the principal investigator for this study (Professor Alison Elliott, telephone 0417 704000).

What if we want to ask someone independent anything about this research, or have any questions about your rights as a research participant? You may speak with the Chairman of the Science and Ethics Committee at Uganda Virus Research Institute on 0414 321962.

UGANDA VIRUS RESEARCH INSTITUTE ENTEBBE
RESEARCH ETHICS COMMITTEE
APPROVED
[illegible]
05 SEP 2020
P. O. BOX 49, ENTEBBE
UGANDA

For peer review only - http://bmjopen.bmj.com/site/about/guidelines.xhtml

PVA ID | A | ___ | ___ | ___ | ___ |

Witness:

I have read the participant information sheet and the consent statement above

to:.. (PRINT NAME OF PARTICIPANT) in a language which he/she understands. I believe that he/she gives consent to take part in the study.

....	....	....
**Witness name**	**Signature**	**Date**

Witness required only for those using a thumb print instead of the final signature, or unable to read the information and consent form, or if the person taking consent does not speak the participant's language. The witness must not be a member of the research staff or a study participant. The witness must be present for the whole consent process.

Person taking the consent:

....	....	....
**Researcher name**	**Signature**	**Date**

**Comment:**

UGANDA VIRUS RESEARCH INSTITUTE ENTEBBE
RESEARCH ETHICS COMMITTEE
APPROVED
05 SEP 2020
P.O. BOX 49, ENTEBBE
UGANDA

For peer review only - http://bmjopen.bmj.com/site/about/guidelines.xhtml

MRC/UVRI and LSHTM Uganda Research Unit

MRC Medical Research Council | Uganda Virus Research Institute | LONDON SCHOOL of HYGIENE &TROPICAL MEDICINE

Population differences in vaccine responses: the effect of intensive treatment for schistosomiasis on response to vaccines among island adolescents

Short Title: POPVAC Project A

Information Sheet for parents/guardians

We are inviting your child to join in some research which is being done by a team from Uganda Virus Research Institute (UVRI), working together with colleagues from the London School of Hygiene and Tropical Medicine, UK. This form will tell you about the research. If you have any questions, please ask the person who is telling you about this research. Then you can decide whether you want your child to take part or not. There is no need for your child to take part unless you really want him/her to do so. If you agree for your child to take part, you will need to sign the consent form to show us that you are happy for him/her to do so. If you decide that you do not want your child to take part, do not sign the consent form.

The people leading this research study are Professor Alison Elliott and Dr Ludoviko Zirimenya.

1. <u>What this research is about, and the reason for doing this research?</u>

Vaccines are a very important tool for preventing infectious diseases. They have saved many lives. Vaccines are usually made from a weakened or killed strain of the bacteria or viruses that cause infectious diseases, or from a part of the bacteria or viruses. Vaccines are designed to help our body's defence system to recognise infectious diseases before we actually meet them, so that we can defeat them more easily. **However, some vaccines seem to work less well in hot countries, near the equator (such as Uganda), than in cooler countries (such as the United Kingdom, the UK).** We want to find out why this is so.

Worm infections are much more common in warm countries (such as Uganda) than in cooler countries (such as the UK). Bilharzia (schistosomiasis) is a worm infection which is very common in Koome islands. Almost everyone in Koome has Bilharzia. Worms can live in our bodies for many years. To do this they have to be able to switch off some of our body's defence systems so that they will not be killed. **We think that this "switching off" of defence systems may also prevent some vaccines from working well.** If this is true then treating worms before giving vaccines might result in better responses to the vaccines.

UGANDA VIRUS RESEARCH INSTITUTE ENTEBBE
RESEARCH ETHICS COMMITTEE
APPROVED
05 SEP 2020
P. O. BOX 49, ENTEBBE
UGANDA

For peer review only - http://bmjopen.bmj.com/site/about/guidelines.xhtml

Light infections with Bilharzia may not be noticed but heavy infections can cause serious damage, especially to the liver. That is why the Ugandan government usually gives treatment for Bilharzia once a year in Ugandan schools. This usually reduces the number of Bilharzia worms but does not get rid of them. **We want to find out whether treating Bilharzia much more frequently (a total of 7 times in a year) can get rid of it completely and whether this will improve vaccine responses.**

2. Why has my child been asked to take part in this study?

Your child has been asked because he/she is attending primary school in Koome islands, in primary 1 to 6. Bilharzia infection is often heaviest in people of primary school age. Also, the government of Uganda offers vaccines to your child's age group that will help to protect them from infectious diseases later on. So it is important to know whether Bilharzia can affect these vaccines, and whether treating Bilharzia effectively is helpful.

3. What is going to happen in this research study?

Your child will have **a health check-up**

If you want your child to take part in this study, we will first check your child's health, and take stool samples, urine samples and some blood from a vein in your child's arm and do some tests. These will include tests for infections including HIV, malaria and worms, tests for anaemia (the strength of blood in your child's body), and tests for pregnancy if your child is a girl.

If everything is okay then we will enrol your child in the study. If something is not okay then we will either give your child the treatment that he/she needs, or tell you what to do.

Your child will be put into one of the two **Bilharzia treatment** groups

When your child is enrolled in the study we will put your child into one of two groups.

- One group will be treated for Bilharzia using praziquantel (the recommended medicine for Bilharzia) seven times in the year, and an eighth time after the end of the study.
- The other group will be treated for Bilharzia using praziquantel once in the year, and a second time after the end of the study.

The choice of groups will be done using a code generated by computer. This works so that your child is put into one group or the other by luck – this is like a lottery.

UGANDA VIRUS RESEARCH INSTITUTE ENTEBBE
RESEARCH ETHICS COMMITTEE
APPROVED
05 SEP 2020
P. O. BOX 49, ENTEBBE
UGANDA

For peer review only - http://bmjopen.bmj.com/site/about/guidelines.xhtml

Your child will be immunised with **several vaccines.** These are the vaccines:

- BCG vaccine. This is intended to protect against tuberculosis. Tuberculosis is very common in Uganda. Many people receive the BCG vaccine as a baby. BCG can be given again at school age and may help to protect against tuberculosis later on although the level of protection varies a lot between countries. We do not know how well it works in school children in Uganda.
- Yellow fever vaccine. This protects against Yellow Fever. Yellow fever is a virus carried by mosquitoes. Yellow fever disease affects the liver and causes fever and jaundice (yellow eyes). Outbreaks occur in Uganda and neighbouring countries from time to time – an outbreak is when a group of people falls sick around the same time.
- HPV vaccine. Human Papilloma Virus (HPV) causes warts. As well, some strains of HPV can cause cancer in the genital areas, especially on the cervix (the opening of the womb) in girls. HPV can also cause cancer of the penis in boys, and other cancers. The HPV vaccine reduces the risk of infection with dangerous HPV strains that cause cancer. In this way it reduces the risk of cancers. The Ugandan government recently started offering this vaccine to girls in primary 4. We will give this vaccine to you if you are a girl (in any class) and you have not received it already. We will also give this vaccine to boys because it can protect them against some cancers too.
- Tetanus and diphtheria vaccines. Tetanus and diphtheria are bacterial infections. The tetanus and diphtheria bacteria produce chemicals called toxins which cause diseases. Tetanus bacteria infect deep wounds and produce tetanus toxin which causes very serious muscle spasms. Tetanus can affect young babies if the wound where the umbilical cord is cut becomes infected. Immunising young women can protect their future babies too. Diphtheria causes a very serious throat infection. Tetanus and diphtheria immunisation is given to babies but booster immunisation is recommended by the Uganda government for school children also.
- Typhoid vaccine. Typhoid is a serious form of fever which is spread through contaminated food. Outbreaks occur in Uganda. Immunisation against typhoid can prevent this illness.

These immunisations will be given at three different time points. BCG will be given first. HPV vaccine, oral typhoid vaccine and yellow fever vaccine will be given four weeks later. A second dose of HPV vaccine and first dose of Tetanus/diphtheria vaccine will be given at 28 weeks after the BCG immunisation.

Most of the vaccines will be injected into your child's upper arm (either right or left). This will be a bit painful, as for any injection of medicine that you may have had, and will feel a bit sore over the next week or so. The BCG vaccine is likely to form a small swelling and then an ulcer

UGANDA VIRUS RESEARCH INSTITUTE ENTEBBE
RESEARCH ETHICS COMMITTEE
APPROVED
VALID UNTIL
05 SEP 2020
P.O. BOX 49, ENTEBBE
UGANDA

For peer review only - http://bmjopen.bmj.com/site/about/guidelines.xhtml

which may take quite some time (perhaps two to five months) to heal. The typhoid vaccine is an oral vaccine: it is in the form of capsules which are swallowed. Your child will be given one capsule per day for three alternating days.

Your child will be asked to give **blood samples**

To test your child's response to the vaccines we will ask your child to give blood samples before and after they are given the vaccines. The amount of blood that we will take at each visit is completely safe and will vary between 4 and 27 millilitres (1 teaspoon and 5 and a half teaspoons) at the different visits. In total we will ask your child to give blood samples at 7 different times during the study. The study will last for about two years.

Your child will be asked to give **stool and urine samples**

At some visits we will ask your child for additional stool and urine samples for testing to check whether the worm infections have responded to treatment or (for girls) whether your child has become pregnant.

Some of the children will be asked to take part in a special **sugar test** of absorption from the intestines

We think that worm infections may make our intestines a little bit leaky, and this might explain some of their effects. We can test this using a special sugar drink. We will ask about 200 children to do this additional test. If your child is asked to do this, it will be done about 8 weeks after the first vaccine is given. He/she will be asked not to eat overnight or in the morning of the test day. Then he/she will be given a special drink that contains sugars early in the morning. Afterwards he/she will also be asked to drink a litre of water (equal to two Rwenzori bottles) over the following few hours. All the urine that he/she passes during the next five hours will be collected. The volume (amount) of urine will be measured and the amount of sugar in it will be tested, to find out how much of the sugar his/her body has absorbed.

If you do not want your child to take part in the study, to have blood, urine and stool samples taken or to receive the treatment and vaccines, you can say no to your child's taking part in this study.

4. What will the blood, stool and urine samples be used for?

The samples will be used to test for infections, including HIV, malaria and worms, to test for anaemia (the strength of your child's blood) and to test for pregnancy (among girls). The blood samples will also be used to test how your child's body responds to vaccines.

UGANDA VIRUS [illegible] INSTITUTE ENTEBBE
RESEARCH ETHICS COMMITTEE
[illegible]
05 SEP 2020
P. O. BOX 49, ENTEBBE
UGANDA

For peer review only - http://bmjopen.bmj.com/site/about/guidelines.xhtml

5. How many people will take part in the study, and how long will it last?

The whole study will enrol 480 people, and the study will last for about two years.

6. What are the risks of participating in this trial?

Your child's time

Your child will need to take time off classes during each visit by the study team. There will be 12 visits altogether. Each visit will usually take about 30 minutes, but the first visit may take about two hours to give time for everything to be explained, and for a thorough check-up. The research team will work with teachers to avoid disturbing classes too much.

Blood samples

Taking blood samples is not expected to cause any problem for your child, apart from the discomfort or pain, bruising or bleeding, redness at the place where the needle goes into the skin, but this will go away in a few days. There is a very small chance that your child may get an infection or some swelling at this place – this almost never happens. Some people faint when their blood is taken. The person taking the blood will do all they can to prevent these things from happening.

Sugar test

During the sugar test your child will be asked not to eat overnight and during the morning of the test so he/she may get hungry (although water and a snack will be provided). Most people have no problems with this test although a few may feel a little sick and stools may become a bit loose.

Pregnancy

We do not know how some of these vaccines might affect a developing baby if they were given to someone who was pregnant. That is why we will do a pregnancy test, if your child is a girl, even if she knows she could not be pregnant. We have to be quite sure. We will test for pregnancy at the beginning of the study and on each immunisation day. We will not enrol your child in the study if she is pregnant at the start. We will not give your child the vaccines if she falls pregnant later.

Bilharzia treatment

Treatment of Bilharzia with praziquantel may make your child feel dizzy or sick, give your child abdominal pain or diarrhoea, or occasionally cause an itchy rash. This is most likely caused by

UGANDA VIRUS RESEARCH INSTITUTE ENTEBBE
RESEARCH ETHICS COMMITTEE
APPROVED
VALID UNTIL
05 SEP 2020
P.O. BOX 49, ENTEBBE
UGANDA

For peer review only - http://bmjopen.bmj.com/site/about/guidelines.xhtml

the body's response to the worms as they are being killed by the medicine. The research team will have medicines available to help your child if he/she has a strong reaction to the treatment.

Immunisations

All the vaccines that we will give have been used to protect very large numbers of people and are known to be safe. They are not expected to cause any major problems for your child. As mentioned, the BCG vaccine is expected to cause an ulcer and to heal slowly, leaving a scar. The injections will cause some pain at the time of injection and your child's arm will feel a bit sore for a day or two. Some people develop flu-like symptoms including headache and fever for a day or two. It is fine for your child to take painkillers like paracetamol (Panadol) for these symptoms. The typhoid vaccine, which your child will swallow as capsules, is not expected to cause your child any problem at all, although your child may experience some stomach pain, feeling sick, vomiting and (rarely) rash.

Very occasionally any vaccine can cause a serious reaction, such as an allergic reaction. The research doctors and nurses will be available to help if this happens.

7. What are the benefits of taking part in this trial?

The treatment for Bilharzia is likely to be good for your child, whichever group he or she is put in. The vaccines are likely to help your child by protecting him/her from infectious diseases.

Also, you and your child will be helping us to find out why vaccines sometimes don't work so well in countries like Uganda, and whether vaccines work better if worms are treated first. This may help other people in the future.

8. What is the cost of taking part in the trial?

There is no cost to participate in this trial. We will reimburse you (the parents) for the time you spend at meetings at school, 20,000/= (twenty thousand shillings) for each visit. Your child will receive a soft drink and the gift of a pen or other simple school material on days when blood samples are drawn, and the gift of a T-shirt at the end of the study when everything is done, in appreciation of his/her contribution to the work.

9. What happens if I refuse for my child to take part?

It is very important for you to know that your child does not have to take part in the research, the choice is yours and your child's. No-one will be upset if your child decides not to take part. The teachers will not be upset and the research team will not be upset. If you agree for your child to take part and later decide that you do not want him/her to take part anymore, that is also okay. Whatever happens, your child will still be able to receive the treatment for Bilharzia

UGANDA VIRUS RESEARCH INSTITUTE
RESEARCH ETHICS COMMITTEE
APPROVED
VALID UNTIL
05 SEP 2020
P. O. BOX 49, ENTEBBE
UGANDA

For peer review only - http://bmjopen.bmj.com/site/about/guidelines.xhtml

and the immunisations for HPV and tetanus and diphtheria when they are provided by the government, if you wish.

10. Who will be able to see the results of tests done on my child's samples?

The research team will keep your child's results private. Only members of the research team will be able to see results and to know that they belong to your child. Your child's samples will be given a special code so that anyone who is working on them in research laboratories will not know they came from your child.

11. What happens if something goes wrong?

You and your child will be making an important contribution to medical research. The researchers will make every effort to ensure your child's safety and well-being. MRC/UVRI and LSHTM has a specialist insurance policy in place which would help to pay for treatment if any harm came to your child as a result of taking part in the research.

12. Who will have access to information from this research?

All our research records are stored securely in rooms with restricted access and on password protected computers. Only research staff trained to keep the information confidential will have access to the records. Your child's name will be removed from the records, so no-one will be able to find out information about your child from our records. The people who may review your child's records include Research Ethics Committees (Uganda Virus Research Institute Research Ethics Committee and the London School of Hygiene & Tropical Medicine Ethics Committee) the Uganda National Council Science and Technology, Study Monitors, Sponsor and the Uganda National Drug Authority. These organisations are there to ensure that your child's rights are protected and that the research is conducted properly and safely.

13. Who has reviewed the trial?

This trial has been reviewed by the Uganda National Council for Science and Technology, the Uganda Virus Research Institute Research and Ethics Committee, the ethics committee of the London School of Hygiene and Tropical Medicine in the United Kingdom. The Uganda National Drug Authority, which regulates the use of all medicines in Uganda, has granted permission to use the medicines and vaccines needed for this clinical trial.

You can find out more about the study at any time by asking one of the members of the research team. You may also contact Professor Alison Elliott (telephone: 0417 704000) who is in charge of the study, or the Chairperson of the Ethics Committee for the Uganda Virus Research Institute on 0414 321962.

UGANDA VIRUS RESEARCH INSTITUTE ENTEBBE
RESEARCH ETHICS COMMITTEE
APPROVED
VALID UNTIL
05 SEP 2020
P. O. BOX 49, ENTEBBE
UGANDA

For peer review only - http://bmjopen.bmj.com/site/about/guidelines.xhtml

MRC/UVRI and LSHTM Uganda Research Unit

MRC Medical Research Council | Uganda Virus Research Institute | LONDON SCHOOL of HYGIENE &TROPICAL MEDICINE

Population differences in vaccine responses: the effect of intensive treatment for schistosomiasis on response to vaccines among island adolescents

Short Title: POPVAC Project A

Parent/guardian consent

A copy of this form will be given to you. Please keep a copy of the form because it contains the information that was discussed with you and you may want to look at it again.

Participant (child) name: ______________________________ **PVA ID|A|___|___|___|___|**
(please write your child's name in capital letters here if you agree)

AGREEMENT BY PARENT OR GUARDIAN:

I have read and/or been fully explained the information sheet concerning my child's participation in this study and I understand what will be required if he or she takes part. I understand that my child's participation is voluntary. My questions concerning this study have been answered. I understand that at any time, I may withdraw my child from this study without giving a reason and without affecting his or her entitlement to government health care. I agree for my child to take part in this study.

Name: ______________________________________
(please write your name in capital letters here if you agree)

Signature: __________________________________ **Date:**________________
(please sign or write your name here if you agree; or use a thumbprint)

What if I have any questions?
If you have any questions about your participation in this study, please feel free to ask any member of the research team at any time. If you prefer, you may speak to the principal investigator for this study (Professor Alison Elliott, telephone 0417 704000).

What if we want to ask someone independent anything about this research, or have any questions about your rights as a research participant? You may speak with the Chairman of the Science and Ethics Committee at Uganda Virus Research Institute on 0414 321962.

UGANDA VIRUS RESEARCH INSTITUTE ENTEBBE
RESEARCH ETHICS COMMITTEE
APPROVED
05 SEP 2020
P. O. BOX 49, ENTEBBE
UGANDA

For peer review only - http://bmjopen.bmj.com/site/about/guidelines.xhtml

PVA ID|A|___|___|___|___|

Witness:

I have read the participant information sheet and the assent statement above

to:.. (PRINT NAME OF PARTICIPANT) in a language which he/she understands. I believe that he/she gives consent for his/her child to take part in the study.

....	....	....
**Witness name**	**Signature**	**Date**

Witness required only for those using a thumb print instead of the final signature, or unable to read the information and consent form, or if the person taking consent does not speak the participant's language. The witness must not be a member of the research staff or a study participant. The witness must be present for the whole consent process.

Person taking the consent:

....	....	....
**Researcher name**	**Signature**	**Date**

Comment:

UGANDA VIRUS RESEARCH INSTITUTE ENTEBBE
RESEARCH ETHICS COMMITTEE
APPROVED
05 SEP 2020
P. O. BOX 49, ENTEBBE
UGANDA

For peer review only - http://bmjopen.bmj.com/site/about/guidelines.xhtml

UGANDA VIRUS RESEARCH INSTITUTE ENTEBBE
RESEARCH ETHICS COMMITTEE
APPROVED
05 SEP 2020
P. O. BOX 49, ENTEBBE
UGANDA

Reporting checklist for protocol of a clinical trial.

Based on the SPIRIT guidelines.

Instructions to authors

Complete this checklist by entering the page numbers from your manuscript where readers will find each of the items listed below.

Your article may not currently address all the items on the checklist. Please modify your text to include the missing information. If you are certain that an item does not apply, please write "n/a" and provide a short explanation.

Upload your completed checklist as an extra file when you submit to a journal.

In your methods section, say that you used the SPIRITreporting guidelines, and cite them as:

Chan A-W, Tetzlaff JM, Altman DG, Laupacis A, Gøtzsche PC, Krleža-Jerić K, Hróbjartsson A, Mann H, Dickersin K, Berlin J, Doré C, Parulekar W, Summerskill W, Groves T, Schulz K, Sox H, Rockhold FW, Rennie D, Moher D. SPIRIT 2013 Statement: Defining standard protocol items for clinical trials. Ann Intern Med. 2013;158(3):200-207

		Reporting Item	Page Number
**Administrative information**			
Title	#1	Descriptive title identifying the study design, population, interventions, and, if applicable, trial acronym	1
Trial registration	#2a	Trial identifier and registry name. If not yet registered, name of intended registry	2
Trial registration: data set	#2b	All items from the World Health Organization Trial Registration Data Set	n/a
Protocol version	#3	Date and version identifier	Information available at ISRCTN60517191
Funding	#4	Sources and types of financial, material, and other support	15
Roles and	#5a	Names, affiliations, and roles of protocol	15

For peer review only

responsibilities: contributorship		contributors	
Roles and responsibilities: sponsor contact information	#5b	Name and contact information for the trial sponsor	Information available at ISRCTN60517191
Roles and responsibilities: sponsor and funder	#5c	Role of study sponsor and funders, if any, in study design; collection, management, analysis, and interpretation of data; writing of the report; and the decision to submit the report for publication, including whether they will have ultimate authority over any of these activities	16
Roles and responsibilities: committees	#5d	Composition, roles, and responsibilities of the coordinating centre, steering committee, endpoint adjudication committee, data management team, and other individuals or groups overseeing the trial, if applicable (see Item 21a for data monitoring committee)	Information detailed in supplementary information file
**Introduction**			
Background and rationale	#6a	Description of research question and justification for undertaking the trial, including summary of relevant studies (published and unpublished) examining benefits and harms for each intervention	4
Background and rationale: choice of comparators	#6b	Explanation for choice of comparators	8
Objectives	#7	Specific objectives or hypotheses	6
Trial design	#8	Description of trial design including type of trial (eg, parallel group, crossover, factorial, single group), allocation ratio, and framework (eg, superiority, equivalence, non-inferiority, exploratory)	7
**Methods: Participants, interventions, and outcomes**			

For peer review only

Study setting	#9	Description of study settings (eg, community clinic, academic hospital) and list of countries where data will be collected. Reference to where list of study sites can be obtained	7
Eligibility criteria	#10	Inclusion and exclusion criteria for participants. If applicable, eligibility criteria for study centres and individuals who will perform the interventions (eg, surgeons, psychotherapists)	7
Interventions: description	#11a	Interventions for each group with sufficient detail to allow replication, including how and when they will be administered	8
Interventions: modifications	#11b	Criteria for discontinuing or modifying allocated interventions for a given trial participant (eg, drug dose change in response to harms, participant request, or improving / worsening disease)	Detailed in supplementary information file
Interventions: adherance	#11c	Strategies to improve adherence to intervention protocols, and any procedures for monitoring adherence (eg, drug tablet return; laboratory tests)	Detailed in supplementary information file
Interventions: concomitant care	#11d	Relevant concomitant care and interventions that are permitted or prohibited during the trial	Detailed in supplementary information file
Outcomes	#12	Primary, secondary, and other outcomes, including the specific measurement variable (eg, systolic blood pressure), analysis metric (eg, change from baseline, final value, time to event), method of aggregation (eg, median, proportion), and time point for each outcome. Explanation of the clinical relevance of chosen efficacy and harm outcomes is strongly recommended	10
Participant timeline	#13	Time schedule of enrolment, interventions (including any run-ins and washouts), assessments, and visits for participants. A schematic diagram is highly recommended (see Figure)	14<br><br>Also detailed in supplementary information file, Table S1

For peer review only

Sample size	#14	Estimated number of participants needed to achieve study objectives and how it was determined, including clinical and statistical assumptions supporting any sample size calculations	11
Recruitment	#15	Strategies for achieving adequate participant enrolment to reach target sample size	Detailed in supplementary information file
**Methods: Assignment of interventions (for controlled trials)**			
Allocation: sequence generation	#16a	Method of generating the allocation sequence (eg, computer-generated random numbers), and list of any factors for stratification. To reduce predictability of a random sequence, details of any planned restriction (eg, blocking) should be provided in a separate document that is unavailable to those who enrol participants or assign interventions	8
Allocation concealment mechanism	#16b	Mechanism of implementing the allocation sequence (eg, central telephone; sequentially numbered, opaque, sealed envelopes), describing any steps to conceal the sequence until interventions are assigned	9
Allocation: implementation	#16c	Who will generate the allocation sequence, who will enrol participants, and who will assign participants to interventions	8, 9
Blinding (masking)	#17a	Who will be blinded after assignment to interventions (eg, trial participants, care providers, outcome assessors, data analysts), and how	9
Blinding (masking): emergency unblinding	#17b	If blinded, circumstances under which unblinding is permissible, and procedure for revealing a participant's allocated intervention during the trial	Detailed in supplementary information file
**Methods: Data collection, management, and analysis**			

For peer review only

Data collection plan	#18a	Plans for assessment and collection of outcome, baseline, and other trial data, including any related processes to promote data quality (eg, duplicate measurements, training of assessors) and a description of study instruments (eg, questionnaires, laboratory tests) along with their reliability and validity, if known. Reference to where data collection forms can be found, if not in the protocol	10.<br><br>More details in supplementary information file
Data collection plan: retention	#18b	Plans to promote participant retention and complete follow-up, including list of any outcome data to be collected for participants who discontinue or deviate from intervention protocols	Detailed in supplementary information file
Data management	#19	Plans for data entry, coding, security, and storage, including any related processes to promote data quality (eg, double data entry; range checks for data values). Reference to where details of data management procedures can be found, if not in the protocol	13.<br><br>More information in the statistical analysis plan found at ISRCTN60517191
Statistics: outcomes	#20a	Statistical methods for analysing primary and secondary outcomes. Reference to where other details of the statistical analysis plan can be found, if not in the protocol	13.<br><br>More information in the statistical analysis plan found at ISRCTN60517191
Statistics: additional analyses	#20b	Methods for any additional analyses (eg, subgroup and adjusted analyses)	11.<br><br>More information in the statistical analysis plan found at ISRCTN60517191
Statistics: analysis population and missing data	#20c	Definition of analysis population relating to protocol non-adherence (eg, as randomised analysis), and any statistical methods to handle missing data (eg, multiple imputation)	Information in the statistical analysis plan found at ISRCTN60517191
**Methods: Monitoring**			

For peer review only

Data monitoring: formal committee	#21a	Composition of data monitoring committee (DMC); summary of its role and reporting structure; statement of whether it is independent from the sponsor and competing interests; and reference to where further details about its charter can be found, if not in the protocol. Alternatively, an explanation of why a DMC is not needed	Detailed in supplementary information file
Data monitoring: interim analysis	#21b	Description of any interim analyses and stopping guidelines, including who will have access to these interim results and make the final decision to terminate the trial	Detailed in supplementary information file
Harms	#22	Plans for collecting, assessing, reporting, and managing solicited and spontaneously reported adverse events and other unintended effects of trial interventions or trial conduct	Detailed in supplementary information file
Auditing	#23	Frequency and procedures for auditing trial conduct, if any, and whether the process will be independent from investigators and the sponsor	Detailed in supplementary information file
**Ethics and dissemination**			
Research ethics approval	#24	Plans for seeking research ethics committee / institutional review board (REC / IRB) approval	12
Protocol amendments	#25	Plans for communicating important protocol modifications (eg, changes to eligibility criteria, outcomes, analyses) to relevant parties (eg, investigators, REC / IRBs, trial participants, trial registries, journals, regulators)	11
Consent or assent	#26a	Who will obtain informed consent or assent from potential trial participants or authorised surrogates, and how (see Item 32)	12.<br>Also detailed in supplementary information file
Consent or assent: ancillary studies	#26b	Additional consent provisions for collection and use of participant data and biological specimens in ancillary studies, if applicable	Detailed in supplementary information file

For peer review only

Confidentiality	#27	How personal information about potential and enrolled participants will be collected, shared, and maintained in order to protect confidentiality before, during, and after the trial	Detailed in supplementary information file
Declaration of interests	#28	Financial and other competing interests for principal investigators for the overall trial and each study site	15
Data access	#29	Statement of who will have access to the final trial dataset, and disclosure of contractual agreements that limit such access for investigators	n/a
Ancillary and post trial care	#30	Provisions, if any, for ancillary and post-trial care, and for compensation to those who suffer harm from trial participation	Detailed in supplementary information file
Dissemination policy: trial results	#31a	Plans for investigators and sponsor to communicate trial results to participants, healthcare professionals, the public, and other relevant groups (eg, via publication, reporting in results databases, or other data sharing arrangements), including any publication restrictions	2, 13
Dissemination policy: authorship	#31b	Authorship eligibility guidelines and any intended use of professional writers	n/a
Dissemination policy: reproducible research	#31c	Plans, if any, for granting public access to the full protocol, participant-level dataset, and statistical code	13
**Appendices**			
Informed consent materials	#32	Model consent form and other related documentation given to participants and authorised surrogates	Supplementary files provided
Biological specimens	#33	Plans for collection, laboratory evaluation, and storage of biological specimens for genetic or molecular analysis in the current trial and for future use in ancillary studies, if applicable	n/a

None The SPIRIT checklist is distributed under the terms of the Creative Commons Attribution License CC-BY-ND 3.0. This checklist can be completed online using https://www.goodreports.org/, a tool made by the EQUATOR Network in collaboration with Penelope.ai

For peer review only
